# Supplementary material for: Chiral aldehyde-nickel dual catalysis enables asymmetric α−propargylation of amino acids and stereodivergent synthesis of NP25302
Source: Nat Commun. 2022 Nov 26;13:7290. doi: 10.1038/s41467-022-35062-2 (PMC9701212; doi:10.1038/s41467-022-35062-2)
Supplement: Supplementary file 1 — Supplementary Information [file 41467_2022_35062_MOESM1_ESM.pdf]

Supplementary Information

for

Chiral aldehyde-nickel dual catalysis enables asymmetric α–propargylation of amino acids and stereodivergent synthesis of NP25302

Fang Zhu, Chao-Xing Li, Zhu-Lian Wu, Tian Cai, Wei Wen*, Qi-Xiang Guo*

Key Laboratory of Applied Chemistry of Chongqing Municipality, and Chongqing Key Laboratory of Soft-Matter Material Chemistry and Function Manufacturing, School of Chemistry and Chemical Engineering, Southwest University, Chongqing, 400715, China.

wenwei1989@swu.edu.cn; qxguo@swu.edu.cn

Table of Contents

1. Supplementary Methods S2

1.1 General data S2

1.2 Reaction condition optimization S2

1.3 General procedure and analytic data S5

1.4 Determination of the absolute configuration of 4a S29

1.5 The stereodivergent synthesis of NP25302 .... S29

1.6 Reaction mechanism investigation S37

1.7 Copies of NMR spectra S42

2. Supplementary References S92

1. Supplementary Methods

1.1 General data

Solvents for reactions were dried appropriately before use: toluene, THF and Et_2O were dried by refluxing with sodium and benzophenone as indicator, CH_2Cl_2 and $CHCl_3$ were dried by refluxing with CaH_2. All other reagents were directly used as purchased from Aladdin, Adamas-beta® and Energy Chemical. ^{1}H NMR (600 MHz) and ^{13}C NMR (150 MHz) spectra were recorded on Bruker Avance 600 MHz spectrometer. Chemical shifts (δ) are reported in ppm from tetramethylsilane (TMS) with the solvent resonance as the internal standard. Proton signal multiplicities are given as s(singlet), d (doublet), t (triplet), q (quartet), m (multiplet), br (broad) or a combination of them. *J*-values are in Hz. HRMS (ESI-Q-TOF) spectra were recorded on Bruker Impact-II. Enantiomer ratios were determined by HPLC (Chiralpak AD-H, IA-H, OD-H, ID-H, IE-H, IF-H columns were purchased from Daicel Chemical Industries, LTD. Optical rotations were determined at λ = 589 nm (sodium D line) by using a Rudolph-API automatic polarimeter. The *tert*-butyl amino acid esters[1], chiral aldehydes[2] and propargylic alcohol acetates[3] were prepared according to literatures.

1.2 Reaction condition optimization

Supplementary Table 1: Base screening

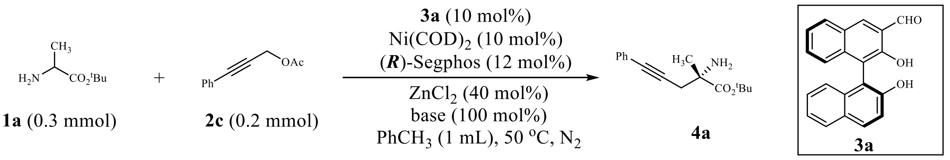

Entry	Base	T (h)	Yield (%)^b	ee (%)^c
1	Et_3N	24	trace	N.D.^{d}
2	Cs_2CO_3	24	16	70
3	^{t}BuOK	24	39	36
4	^{t}BuONa	24	16	73
5	BTMG^e	24	31	79
6	DBU	24	15	83
7	DBN^f	24	7	97
8	TBD^g	24	14	67
9	MTBD^h	24	43	85
10	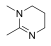	24	14	86
11	TDMAIP^i	24	17	87
12	TMG^g	24	49	94

^{a} Unless noted otherwise, reactions were performed with **1a** (0.30 mmol), **2c** (0.20 mmol), catalyst **3a** (0.02 mmol), (***R***)-Segphos (0.024 mmol), $Ni(COD)_2$ (0.02 mmol), TMG (0.20 mmol), and $ZnCl_2$ (0.08 mmol) in toluene (1.0 mL) at 50 °C. ^{b}Isolated yield. ^{c}Determined by chiral HPLC analysis. ^{d} N.D. = Not determined. ^{e}2-tert-butyl-1,1,3,3-tetramethylguanidine. ^{f}1,5-diazabicyclo [4.3.0] non-5-ene. ^{g}1,5,7-triazabicyclo [4.4.0] dec-5-ene. ^{h}1,3,4,6,7,8-hexahydro-1-methyl-2H-pyrimido [1,2-a] pyrimidine. ^{i}lmino-tnis(dimethytamino)phosphorane.

[g]1,1,3,3-tetramethylguanidine.

Supplementary Table 2: Base equivalent screening

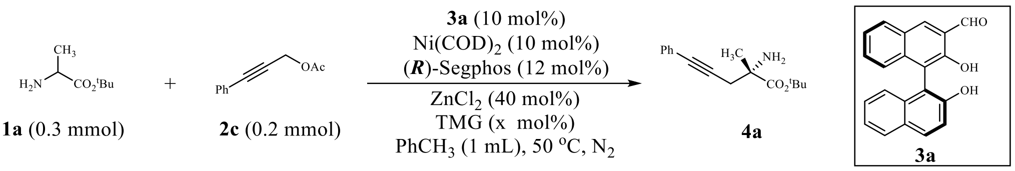

Entry	x (mmol)	T (h)	Yield (%)[b]	ee (%)[c]
**1**	70	24	40	90
**2**	100	24	49	94
**3**	130	24	65	92
**4**	**160**	**24**	**71**	**93**
**5**	190	24	28	90
**6**	220	24	15	77

[a] Unless noted otherwise, reactions were performed with **1a** (0.30 mmol), **2c** (0.20 mmol), catalyst **3a** (0.02 mmol), (***R***)-Segphos (0.024 mmol), Ni(COD)$_2$ (0.02 mmol), TMG (x mmol), and $ZnCl_2$ (0.08 mmol) in toluene (1.0 mL) at 50 °C. [b]Isolated yield. [c]Determined by chiral HPLC analysis.

Supplementary Table 3: Ligand screening

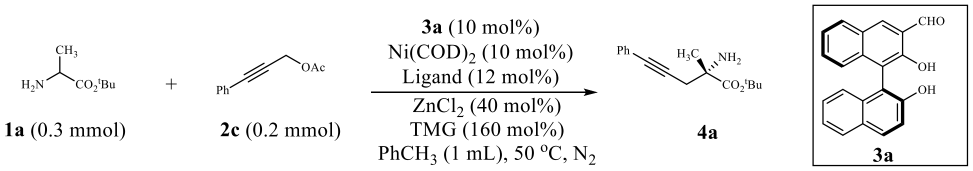

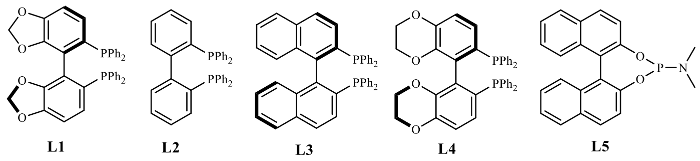

Entry	**Ligand**	T (h)	Yield (%)[b]	ee (%)[c]
1	**dppp**	24	N.R.	N.D.[d]
2	**dppf**	24	N.R.	N.D.[d]
3	**L1**	24	71	93
4	**L2**	24	6	22
5	**L3**	24	10	87
6	**L4**	24	80	91
7	**L5**	24	N.R.	N.D.[d]

[a] Unless noted otherwise, reactions were performed with **1a** (0.30 mmol), **2c** (0.20 mmol), catalyst **3a** (0.02 mmol), Ligand (0.024 mmol), Ni(COD)$_2$ (0.02 mmol), TMG (0.32 mmol), and $ZnCl_2$ (0.08 mmol) in toluene (1.0 mL) at 50 °C. [b]Isolated yield. [c]Determined by chiral HPLC analysis.

Supplementary Table 4: Lewis acid screening

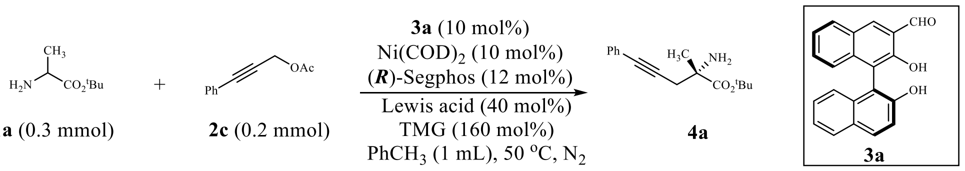

Entry	Lewis acid	T (h)	Yield (%)[b]	ee (%)[c]
1	/	24	17	95
2	$MgBr_2$	24	trace	N.D.[d]
3	$LiCl_2$	24	trace	N.D.[d]
4	$CuCl_2$	24	trace	N.D.[d]
5	$ZnCl_2$	24	71	93
6	$ZnBr_2$	24	32	84
7	ZnF_2	24	16	92
8	$Zn(OTf)_2$	24	17	74
9	$Zn(OAc)_2$	24	trace	N.D.
10	$Zn(BF_4)_2$•XH_2O	24	28	92
11	$Zn(ClO_4)_2$•$6H_2O$	24	8	78

[a] Unless noted otherwise, reactions were performed with **1a** (0.30 mmol), **2c** (0.20 mmol), catalyst **3a** (0.02 mmol), (***R***)-Segphos (0.024 mmol), $Ni(COD)_2$ (0.02 mmol), TMG (0.32 mmol), and Lewis acid (0.08 mmol) in toluene (1.0 mL) at 50 °C. [b]Isolated yield. [c]Determined by chiral HPLC analysis. [d]N.D. = Not determined.

Supplementary Table 5: Lewis acid equivalent screening

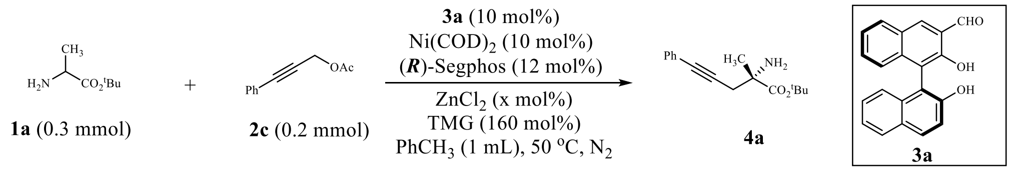

Entry	$ZnCl_2$ (x mol)	T (h)	Yield (%)[b]	ee (%)[c]
1	20	24	13	81
2	40	24	71	93
3	60	24	40	89
4	80	24	53	93
5	100	24	9	81

[a] Unless noted otherwise, reactions were performed with **1a** (0.30 mmol), **2c** (0.20 mmol), catalyst **3a** (0.02 mmol), (***R***)-Segphos (0.024 mmol), $Ni(COD)_2$ (0.02 mmol), TMG (0.32 mmol), and $ZnCl_2$ (x mmol) in toluene (1.0 mL) at 50 °C. [b]Isolated yield. [c]Determined by chiral HPLC analysis. [d]N.D. = Not determined.

Supplementary Table 6: Screening of the alkoxyl group of amino acid ester

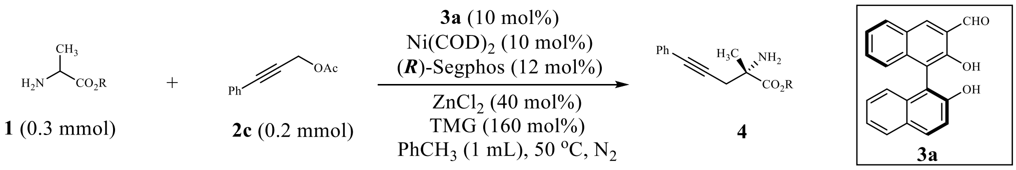

Entry	R	T (h)	Yield (%)[b]	ee (%)[c]
1	Me	24	trace	N.D.[d]
2	Et	24	23	85
3	^{i}Pr	24	50	85
4	^{t}Bu	24	71	93

[a] Unless noted otherwise, reactions were performed with **1** (0.30 mmol), **2c** (0.20 mmol), catalyst **3a** (0.02 mmol), (***R***)-Segphos (0.024 mmol), $Ni(COD)_2$ (0.02 mmol), TMG (0.32 mmol), and $ZnCl_2$ (0.08 mmol) in toluene (1.0 mL) at 50 °C. [b]Isolated yield. [c]Determined by chiral HPLC analysis. [d]N.D. = Not determined.

Supplementary Table 7: Solvent screening

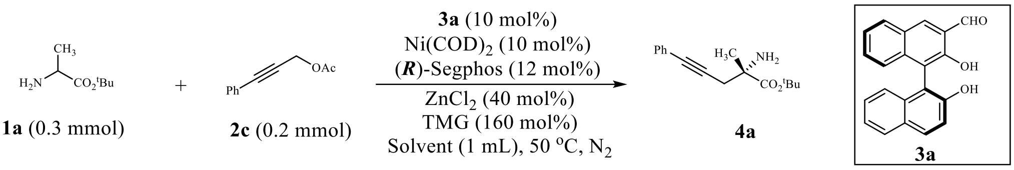

Entry	Solvent	T(h)	Yield (%)[b]	ee (%)[c]
1	DME	24	38	82
2	THF	24	24	84
3	dioxane	24	14	93
4	$PhCH_3$	24	71	93
5	*o*-xylenes	24	4	89
6	mesitylene	24	4	87
7	PhCl	24	14	93
8	CH_3CN	24	7	75
9	EA	24	41	85
10	DCE	24	trace	N.D.[d]

[a] Unless noted otherwise, reactions were performed with **1a** (0.30 mmol), **2c** (0.20 mmol), catalyst **3a** (0.02 mmol), (***R***)-Segphos (0.024 mmol), $Ni(COD)_2$ (0.02 mmol), TMG (0.32 mmol), and $ZnCl_2$ (0.08 mmol) in Solvent (1.0 mL) at 50 °C. [b]Isolated yield. [c]Determined by chiral HPLC analysis. [d] N.D. = Not determined.

1.3 General procedure and analytic data

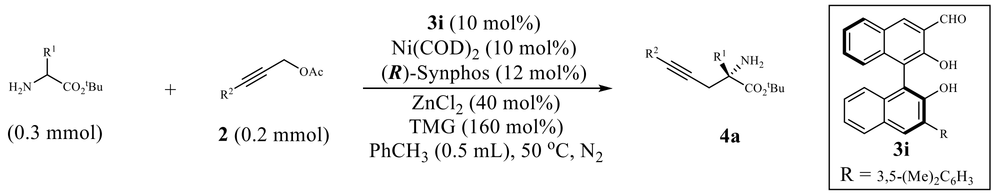

General procedure: In a nitrogen-filled glove box, an oven-dried 10 mL screw-cap reaction tube equipped with a stir bar was charged with $Ni(COD)_2$ (5.5 mg, 0.02 mmol), (*R*)-Synphos (15.3 mg, 0.024 mmol) and stirred in toluene (0.5 mL) at r.t. for about 5 min. Then, *tert*-butyl amino acid ester **1** (0.3 mmol), propargylic acetate ester **2** (0.2 mmol), chiral aldehyde **3i** (8.2 mg, 0.02 mmol), $ZnCl_2$ (10.9 mg, 0.08 mmol) and TMG (36.8 mg, 0.32 mmol) were added. The mixture was continuously stirred at 50 °C under nitrogen atmosphere. After the reaction completed, the solvent was removed by rotary evaporation, and the residue was purified by flash chromatography separation on silica gel column (eluent: petroleum ether/ ethyl acetate/ triethylamine =250/100/2).

tert-Butyl (S)-2-amino-2-methyl-5-phenylpent-4-ynoate (4a):

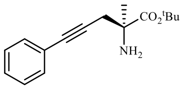

Colorless oil (48.6 mg, 92%); R_f = 0.26 (petroleum ether/ ethyl acetate = 2.5:1); the enantiomeric excess was determined to be 96% by HPLC analysis on Daicel Chirapak OD-H column (hexane/isopropanol =95/5, flow rate 0.6 mL/min, T = 30 °C), UV 254 nm,

t_R (major) 9.866 min, t_R (minor) 9.126 min; $[\alpha]_D^{20}$ = -20.45 (c = 0.92, CH_2Cl_2); **^{1}H NMR (600 MHz, $CDCl_3$)** δ 7.38-7.37 (m, 2H), 7.28-7.27 (m, 3H), 2.84 (d, J = 18.0Hz, 1H), 2.62 (d, J = 18.0 Hz, 1H), 1.87 (s, 2H), 1.49 (s, 9H), 1.39 (s, 3H). **^{13}C NMR (151 MHz, $CDCl_3$)** δ 175.38, 131.62, 128.20, 127.87, 123.40, 85.53, 83.23, 81.26, 57.97, 32.09, 27.94, 25.9. **HRMS(ESI)** m/z: $[M+H]^+$ Calculated for $C_{16}H_{22}NO_2^+$ 260.1645 found 260.1645.

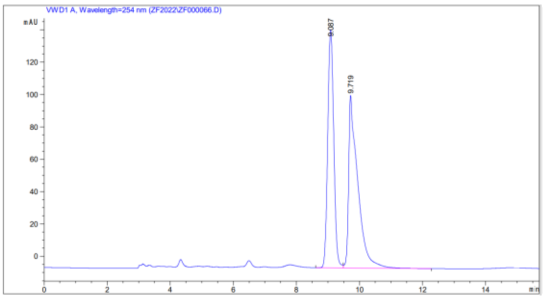

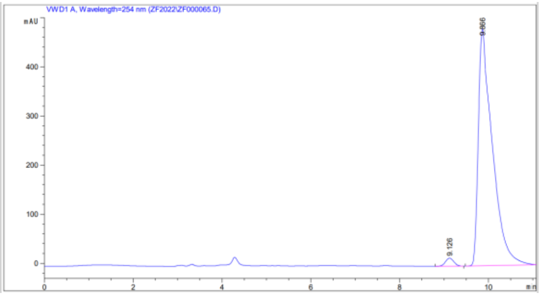

Peak #	RetTime [min]	Type	Width [min]	Area [mAU*s]	Height [mAU]	Area %
1	9.087	BV	0.2074	1951.15076	146.94365	47.5285
2	9.719	VB	0.2589	2154.07495	106.67879	52.4715

Peak #	RetTime [min]	Type	Width [min]	Area [mAU*s]	Height [mAU]	Area %
1	9.126	BB	0.2051	213.59351	16.32781	1.9800
2	9.866	BBA	0.3002	1.05739e4	480.96964	98.0200

tert-Butyl (S)-2-amino-5-(2-fluorophenyl)-2-methylpent-4-ynoate (4b):

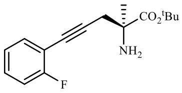

Yellow oil (32.7 mg, 59 %); R_f = 0.23 (petroleum ether/ ethyl acetate = 2.5:1); the enantiomeric excess was determined to be 94% by HPLC analysis on Daicel Chirapak OD-H column (hexane/isopropanol =95/5, flow rate 0.6 mL/min, T = 30 °C), UV 254 nm, t_R (major) 11.598 min, t_R (minor) 12.391 min; $[\alpha]_D^{20}$ = -27.43 (c = 0.63, CH_2Cl_2); **^{1}H NMR (600 MHz, $CDCl_3$)** δ 7.37 (t, J = 6.9 Hz, 1H), 7.27-7.24 (m, 1H), 7.08 – 7.00 (m, 2H), 2.89 (d, J = 18.0 Hz, 1H), 2.67 (d, J = 18.0 Hz, 1H), 1.88 (s, 2H), 1.48 (s, 9H), 1.40 (s, 3H). **^{13}C NMR (151 MHz, $CDCl_3$)** δ 175.25, 163.75, 162.09, 133.51, 129.55, 129.50, 123.80, 123.78, 115.44, 115.30, 111.94, 111.84, 91.10, 81.36, 76.57, 57.89, 32.21, 27.89, 25.97. **^{19}F NMR (565 MHz, $CDCl_3$)** δ -110.42. **HRMS(ESI)** m/z: $[M+H]^+$ Calculated for $C_{16}H_{21}FNO_2^+$ 278.1551 found 278.1551.

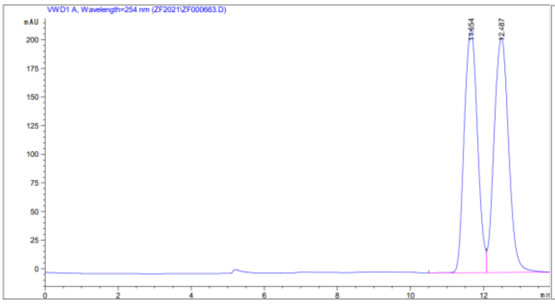

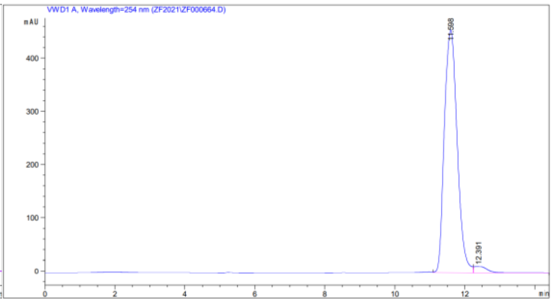

Peak #	RetTime [min]	Type	Width [min]	Area [mAU*s]	Height [mAU]	Area %
1	11.654	BV	0.4072	5330.35449	211.88858	48.4518
2	12.487	VBA	0.4418	5670.99414	205.27905	51.5482

Peak #	RetTime [min]	Type	Width [min]	Area [mAU*s]	Height [mAU]	Area %
1	11.598	VV	0.4121	1.16113e4	456.20041	97.2637
2	12.391	VBA	0.4102	326.66382	12.08332	2.7363

tert-Butyl (S)-2-amino-5-(2-chlorophenyl)-2-methylpent-4-ynoate (4c):

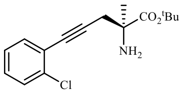

Yellow oil (25.6 mg, 44 %); R_f = 0.25 (petroleum ether/ ethyl acetate = 2.5:1); the enantiomeric excess was determined to be 92% by HPLC analysis on Daicel Chirapak OD-H column (hexane/isopropanol = 90/10, flow rate 1.0 mL/min, T = 30 ^{o}C), UV 254 nm, t_R (major) 5.734 min, t_R (minor) 6.812 min; $[\alpha]_D^{20}$ = -23.55 (c = 0.53, CH_2Cl_2); **^{1}H NMR (600 MHz, $CDCl_3$)** δ 7.47 – 7.33 (m, 2H), 7.25 – 7.13 (m, 2H), 2.91 (d, J = 18.0 Hz, 1H), 2.70 (d, J = 18.0 Hz, 1H), 1.87 (s, 2H), 1.48 (s, 9H), 1.42 (s, 3H). **^{13}C NMR (151 MHz, $CDCl_3$)** δ 175.32, 136.01, 133.40, 129.24, 128.99, 126.44, 123.35, 91.40, 81.45, 80.23, 58.03, 32.25, 28.03, 26.18. **HRMS(ESI)** m/z: $[M+H]^+$ Calculated for $C_{16}H_{21}ClNO_2^+$ 260.1645 found 260.1645.

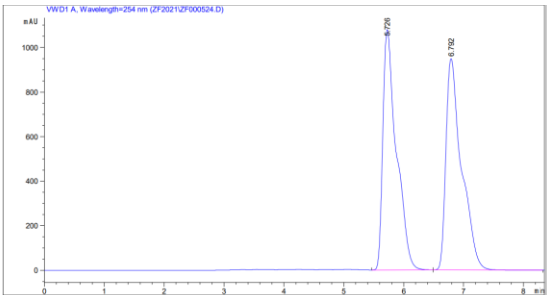

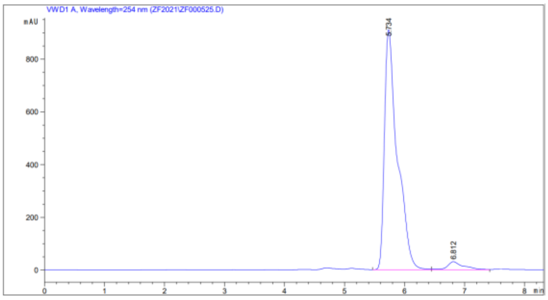

Peak #	RetTime [min]	Type	Width [min]	Area [mAU*s]	Height [mAU]	Area %
1	5.726	BB	0.2272	1.69536e4	1078.22937	49.7145
2	6.792	BBA	0.2619	1.71483e4	948.78607	50.2855

Peak #	RetTime [min]	Type	Width [min]	Area [mAU*s]	Height [mAU]	Area %
1	5.734	BV	0.2147	1.35471e4	908.87762	95.8968
2	6.812	VV	0.2652	579.65405	30.33332	4.1032

tert-Butyl (S)-2-amino-2-methyl-5-(o-tolyl)pent-4-ynoate (4d):

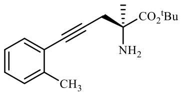

Yellow oil (16.9 mg, 31 %); R_f = 0.23 (petroleum ether/ ethyl acetate = 2.5:1); the enantiomeric excess was determined to be 94% by HPLC analysis on Daicel Chirapak OD-H column (hexane/isopropanol = 90/10, flow rate 1.0 mL/min, T = 30 ^{o}C), UV 254 nm, t_R (major) 9.213 min, t_R (minor) 11.239 min; $[\alpha]_D^{20}$ = -19.46 (c = 0.44, CH_2Cl_2); **^{1}H NMR (600 MHz, $CDCl_3$)** δ 7.35 (d, J = 7.6 Hz, 1H), 7.19-7.17 (m, 2H), 7.12 – 7.06 (m, 1H), 2.90 (d, J = 16.4 Hz, 1H), 2.70 (d, J = 16.4 Hz, 1H), 2.40 (s, 3H), 2.02 (s, 2H), 1.48 (s, 9H), 1.41 (s, 3H). **^{13}C NMR (151 MHz, $CDCl_3$)** δ 175.18, 140.00, 132.02, 129.30, 127.86, 125.42, 123.17, 89.23, 82.23, 81.32, 57.99, 32.12, 27.94, 25.88, 20.75. **HRMS(ESI)** m/z: $[M+H]^+$ Calculated for $C_{17}H_{24}NO_2^+$ 274.1802 found 2274.1801.

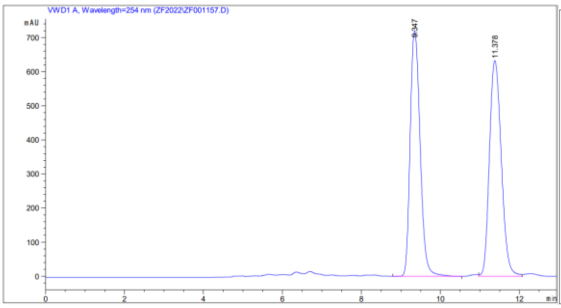

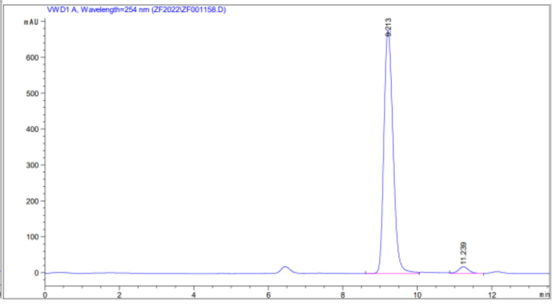

Peak #	RetTime [min]	Type	Width [min]	Area [mAU*s]	Height [mAU]	Area %
1	9.347	BB	0.2777	1.26318e4	719.69812	48.9235
2	11.378	VV	0.3333	1.31877e4	633.26050	51.0765

Peak #	RetTime [min]	Type	Width [min]	Area [mAU*s]	Height [mAU]	Area %
1	9.213	BV	0.2689	1.15600e4	677.89630	97.0645
2	11.239	VB	0.2910	349.60904	18.45647	2.9355

tert-Butyl (S)-2-amino-5-(3-chlorophenyl)-2-methylpent-4-ynoate (4e):

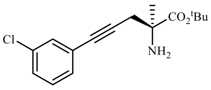

Yellow oil (39.1 mg, 67 %); R_f = 0.17 (petroleum ether/ ethyl acetate = 2.5:1); the enantiomeric excess was determined to be 92% by HPLC analysis on Daicel Chirapak OD-H column (hexane/isopropanol = 95/5, flow rate 0.8 mL/min, T = 30 °C), UV 254 nm, t_R (major) 11.254 min, t_R (minor) 10.285 min; $[\alpha]_D^{20}$ = -18.11 (c = 0.55, CH_2Cl_2); **^{1}H NMR (600 MHz, $CDCl_3$)** δ 7.36 (s, 1H), 7.29 – 7.22 (m, 2H), 7.20 (t, *J* = 7.8 Hz, 1H), 2.83 (d, *J* = 18.0 Hz, 1H), 2.62 (d, *J* = 18.0 Hz, 1H), 1.81 (s, 2H), 1.49 (s, 9H), 1.39 (s, 3H). **^{13}C NMR (151 MHz, $CDCl_3$)** δ 175.27, 134.05, 131.51, 129.77, 129.44, 128.21, 125.09, 87.05, 81.90, 81.37, 57.93, 32.03, 27.94, 25.93. **HRMS(ESI)** m/z: $[M+H]^+$ Calculated for $C_{16}H_{21}ClNO_2^+$ 294.1255 found 294.1255.

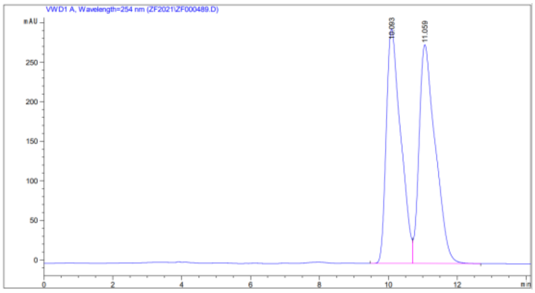

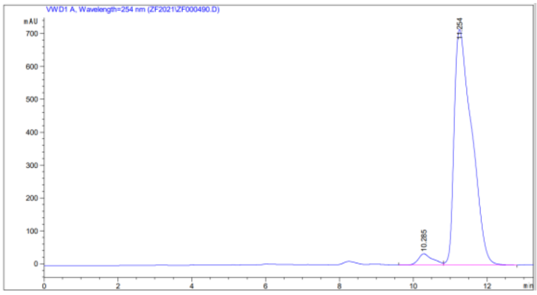

Peak #	RetTime [min]	Type	Width [min]	Area [mAU*s]	Height [mAU]	Area %
1	10.093	BV	0.4241	8618.12305	296.12140	48.8144
2	11.059	VB	0.4716	9036.75391	276.52036	51.1856

Peak #	RetTime [min]	Type	Width [min]	Area [mAU*s]	Height [mAU]	Area %
1	10.285	BV	0.3774	918.02344	34.19299	3.7297
2	11.254	VB	0.4734	2.36961e4	716.24127	96.2703

tert-Butyl (S)-2-amino-2-methyl-5-(m-tolyl)pent-4-ynoate (4f):

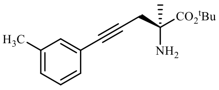

Yellow oil (50.5 mg, 92 %); R_f = 0.18 (petroleum ether/ ethyl acetate = 3:1); the enantiomeric excess was determined to be 92% by HPLC analysis on Daicel Chirapak OD-H column (hexane/isopropanol = 90/10, flow rate 1.0 mL/min, T = 30 °C), UV 254 nm, t_R (major) 9.829 min, t_R (minor) 8.748 min; $[\alpha]_D^{20}$ = -23.96 (c = 0.98, CH_2Cl_2); **^{1}H NMR (600 MHz, $CDCl_3$)** δ 7.23 – 7.13 (m, 3H), 7.08 (d, *J* = 7.2 Hz, 1H), 2.84 (d, *J* = 18.0 Hz, 1H), 2.62

(d, J = 18.0 Hz, 1H), 2.30 (s, 3H), 1.96 (s, 2H), 1.49 (s, 9H), 1.39 (s, 3H). **^{13}C NMR (151 MHz, $CDCl_3$)** δ 175.34, 137.84, 132.23, 128.77, 128.69, 128.10, 123.19, 85.07, 83.42, 81.25, 57.98, 32.07, 27.94, 25.89, 21.16. **HRMS(ESI)** m/z: $[M+H]^+$ Calculated for $C_{17}H_{24}NO_2^+$ 274.1802 found 274.1802.

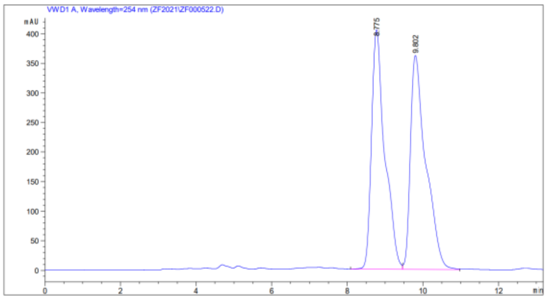

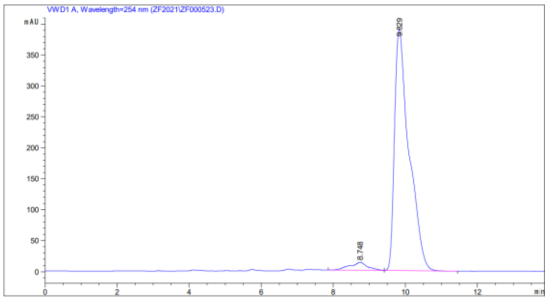

Peak #	RetTime [min]	Type	Width [min]	Area [mAU*s]	Height [mAU]	Area %
1	8.775	BV	0.3540	9849.19629	404.60397	49.0232
2	9.802	VV	0.4091	1.02417e4	361.71030	50.9768

Peak #	RetTime [min]	Type	Width [min]	Area [mAU*s]	Height [mAU]	Area %
1	8.748	BV	0.4572	442.65982	12.94578	3.8995
2	9.829	VB	0.4059	1.09091e4	388.94629	96.1005

tert-Butyl (S)-2-amino-2-methyl-5-(4-(trifluoromethyl)phenyl)pent-4-ynoate (4g):

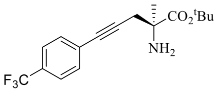

Yellow oil (26.0 mg, 40 %); R_f = 0.28 (petroleum ether/ ethyl acetate = 2:1); the enantiomeric excess was determined to be 96% by HPLC analysis on Daicel Chirapak AD-H column (hexane/isopropanol = 95/5, flow rate 1.0 mL/min, T = 30 °C), UV 254 nm, t_R (major) 7.213 min, t_R (minor) 8.089 min; $[\alpha]_D^{20}$ = -21.55 (c = 0.56, CH_2Cl_2); **^{1}H NMR (600 MHz, $CDCl_3$)** δ 7.50 (dd, J = 38.4, 8.1 Hz, 4H), 2.86 (d, J = 16.5 Hz, 1H), 2.65 (d, J = 16.5 Hz, 1H), 1.82 (s, 2H), 1.49 (s, 9H), 1.40 (s, 3H). **^{13}C NMR (151 MHz, $CDCl_3$)** δ 175.20, 131.84, 129.84, 129.62, 127.23, 125.19, 125.16, 125.14, 125.11, 124.83, 123.03, 88.50, 82.00, 81.38, 57.93, 32.09, 27.92, 25.92. **^{19}F NMR (565 MHz, $CDCl_3$)** δ -62.86. **HRMS(ESI)** m/z: $[M+H]^+$ Calculated for $C_{17}H_{21}F_3NO_2^+$ 328.1519 found 328.1518.

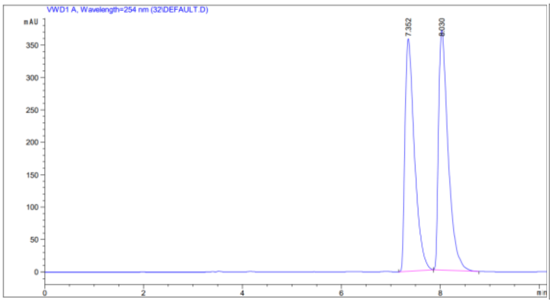

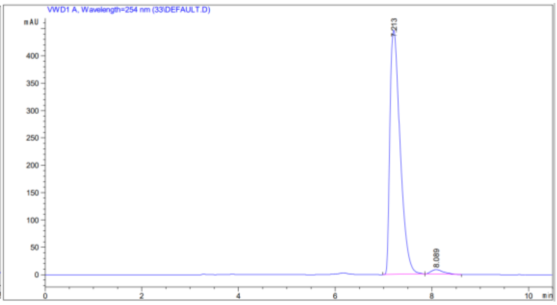

Peak #	RetTime [min]	Type	Width [min]	Area [mAU*s]	Height [mAU]	Area %
1	7.352	BB	0.2021	4778.92236	358.66745	48.9808
2	8.030	BB	0.2035	4977.79834	370.23245	51.0192

Peak #	RetTime [min]	Type	Width [min]	Area [mAU*s]	Height [mAU]	Area %
1	7.213	BB	0.2269	6563.94189	446.40399	97.8734
2	8.089	BB	0.2587	142.61838	8.12676	2.1266

Methyl (S)-4-(4-amino-5-(tert-butoxy)-4-methyl-5-oxopent-1-yn-1-yl)benzoate (4h):

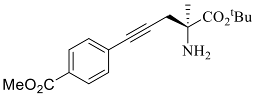

Yellow oil (46.9 mg, 74 %); R_f = 0.21 (petroleum ether/ ethyl acetate = 2:1); the enantiomeric excess was determined to be 95% by HPLC analysis on Daicel Chirapak AD-H column (hexane/isopropanol = 93/7, flow rate 1.0 mL/min, T = 30 °C), UV 254 nm, t_R (major) 10.677 min, t_R (minor) 11.399 min; $[\alpha]_D^{20}$ = -22.67 (c = 0.73, CH_2Cl_2); **1H NMR (600 MHz, CDCl3)** δ 7.95 (d, J = 8.2 Hz, 2H), 7.43 (d, J = 8.2 Hz, 2H), 3.91 (s, 3H), 2.86 (d, J = 16.5 Hz, 1H), 2.65 (d, J = 16.5 Hz, 1H), 1.81 (s, 2H), 1.49 (s, 9H), 1.40 (s, 3H). **13C NMR (151 MHz, CDCl3)** δ 175.23, 166.53, 131.53, 129.41, 129.30, 128.13, 89.02, 82.61, 81.37, 57.95, 52.12, 32.17, 27.93, 25.94. **HRMS(ESI)** m/z: $[M+H]^+$ Calculated for $C_{18}H_{24}NO_4{}^+$ 318.1700 found 318.1699.

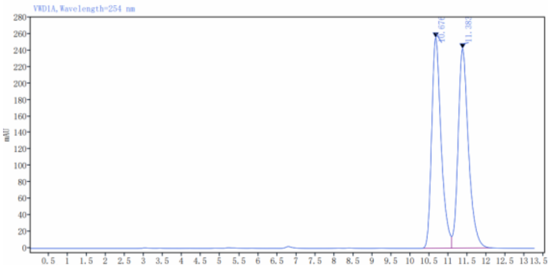

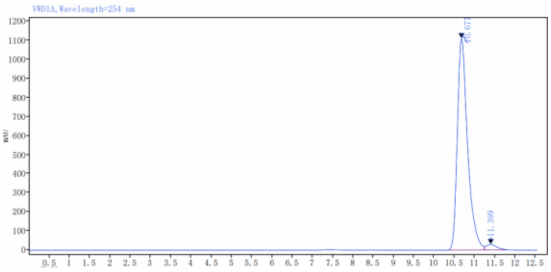

RetTime [min]	Type	Width[min]	Area[mAU^s]	Height[mAU]	Area%
10.676	BV	0.7778	4434.4260	255.8103	49.1008
11.383	VBA	1.0655	4596.8485	242.3980	50.8992

RetTime [min]	Type	Width[min]	Area[mAU^s]	Height[mAU]	Area%
10.677	BV	0.9294	19534.2258	1108.4396	97.3070
11.399	VBA	0.6339	540.6230	29.1927	2.6930

tert-Butyl (S)-2-amino-5-(4-bromophenyl)-2-methylpent-4-ynoate (4i):

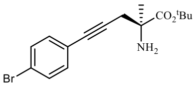

Yellow oil (50.9 mg, 75 %); R_f = 0.25 (petroleum ether/ ethyl acetate = 2:1); the enantiomeric excess was determined to be 97% by HPLC analysis on Daicel Chirapak AD-H column (hexane/isopropanol = 90/10, flow rate 1.0 mL/min, T = 30 °C), UV 254 nm, t_R (major) 6.074 min, t_R (minor) 6.766 min; $[\alpha]_D^{20}$ = -22.13 (c = 1.0, CH_2Cl_2); **^{1}H NMR (600 MHz, CDCl$_3$)** δ 7.41 (d, *J* = 8.4 Hz, 2H), 7.23 (d, *J* = 8.4 Hz, 2H), 2.82 (d, *J* = 18.0 Hz, 1H), 2.60 (d, *J* = 18.0 Hz, 1H), 1.85 (s, 2H), 1.48 (s, 9H), 1.38 (s, 3H). **^{13}C NMR (151 MHz, CDCl$_3$)** δ 175.30, 133.07, 131.47, 122.34, 122.05, 86.91, 82.19, 81.32, 57.92, 32.10, 27.93, 25.92. **HRMS(ESI)** m/z: $[M+H]^+$ Calculated for $C_{16}H_{21}BrNO^+$ 338.0750 found 338.0750.

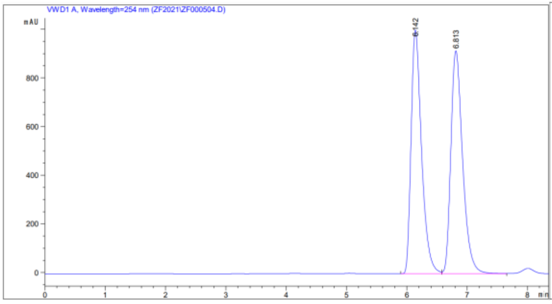

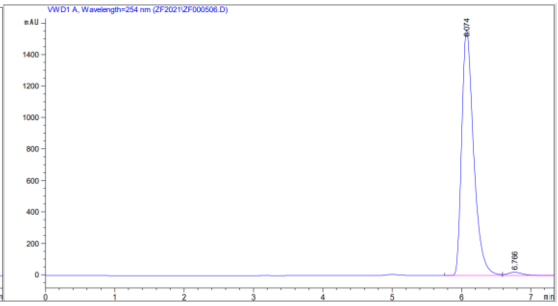

Peak #	RetTime [min]	Type	Width [min]	Area [mAU*s]	Height [mAU]	Area %
1	6.142	BV	0.1810	1.20262e4	1000.75604	49.1768
2	6.813	VV	0.2063	1.24289e4	916.51459	50.8232

Peak #	RetTime [min]	Type	Width [min]	Area [mAU*s]	Height [mAU]	Area %
1	6.074	BV	0.1813	1.85714e4	1557.97571	98.3441
2	6.766	VBA	0.2214	312.69989	21.05198	1.6559

tert-Butyl (S)-5-([1,1'-biphenyl]-4-yl)-2-amino-2-methylpent-4-ynoate (4j):

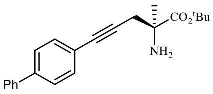

Yellow solid (56.9 mg, 85 %); m.p. = 44-47 °C; R_f = 0.25 (petroleum ether/ ethyl acetate = 2:1); the enantiomeric excess was determined to be 97% by HPLC analysis on Daicel Chirapak OD-H column (hexane/isopropanol = 90/10, flow rate 1.0 mL/min, T = 30 °C), UV 254 nm, t_R (major) 7.557 min, t_R (minor) 6.303 min; $[\alpha]_D^{20}$ = -28.96 (c = 1.1, CH_2Cl_2); **^{1}H NMR (600 MHz, $CDCl_3$)** δ 7.56 (d, *J* = 7.4 Hz, 2H), 7.51 (d, *J* = 8.2 Hz, 2H), 7.43 (dd, *J* = 16.9, 8.1 Hz, 4H), 7.33 (t, *J* = 7.4 Hz, 1H), 2.87 (d, *J* = 18.0 Hz, 1H), 2.64 (d, *J* = 18.0 Hz, 1H), 1.88 (s, 2H), 1.50 (s, 9H), 1.40 (s, 3H). **^{13}C NMR (151 MHz, $CDCl_3$)** δ 175.40, 140.68, 140.42, 132.05, 128.83, 127.55, 126.99, 126.92, 122.34, 86.27, 83.11, 81.28, 58.02, 32.21, 27.98, 25.97. **HRMS(ESI)** m/z: $[M+H]^+$ Calculated for $C_{22}H_{26}NO_2^+$ 336.1958 found 336.1960.

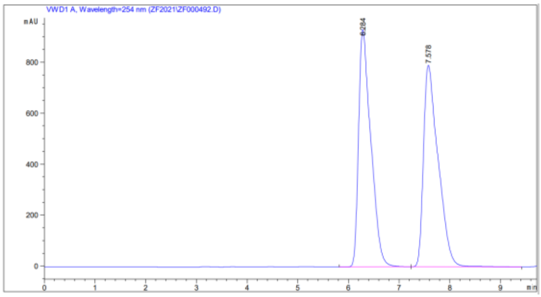

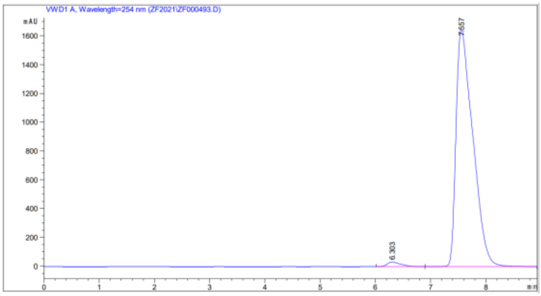

Peak #	RetTime [min]	Type	Width [min]	Area [mAU*s]	Height [mAU]	Area %
1	6.284	BB	0.2404	1.55714e4	930.54773	49.5225
2	7.578	BB	0.2898	1.58717e4	790.04590	50.4775

Peak #	RetTime [min]	Type	Width [min]	Area [mAU*s]	Height [mAU]	Area %
1	6.303	BB	0.2373	523.59851	31.79008	1.4867
2	7.557	BBA	0.3041	3.46952e4	1646.88794	98.5133

tert-Butyl (S)-2-amino-5-(4-(tert-butyl)phenyl)-2-methylpent-4-ynoate (4k):

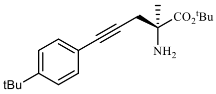

Yellow oil (59.8 mg, 95 %); R_f = 0.18 (petroleum ether/ ethyl acetate = 2.5:1); the enantiomeric excess was determined to be 97% by HPLC analysis on Daicel Chirapak IE-H column (hexane/isopropanol = 95/5, flow rate 0.7 mL/min, T = 30 °C), UV 254 nm, t_R (major) 14.545 min, t_R (minor) 14.031 min; $[\alpha]_D^{20}$ = -22.10 (c = 1.2, CH_2Cl_2); **^{1}H NMR**

(600 MHz, CDCl$_3$) δ 7.30 (q, *J* = 8.4 Hz, 4H), 2.83 (d, *J* = 18.0 Hz, 1H), 2.61 (d, *J* = 18.0 Hz, 1H), 1.85 (s, 2H), 1.49 (s, 9H), 1.38 (s, 3H), 1.29 (s, 9H). **^{13}C NMR (151 MHz, CDCl$_3$)** δ 175.40, 151.09, 131.34, 125.18, 120.39, 84.72, 83.28, 81.19, 57.96, 34.68, 32.14, 31.16, 27.96, 25.95. **HRMS(ESI)** m/z: [M+H]$^+$ Calculated for $C_{20}H_{30}NO_2{}^+$ 316.2271 found 316.2273.

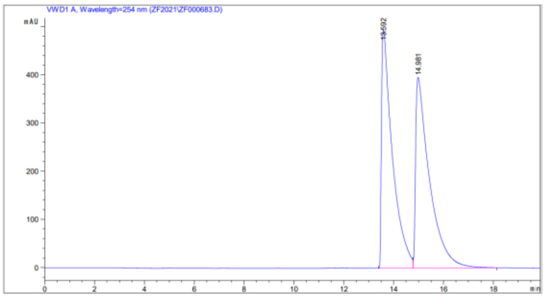

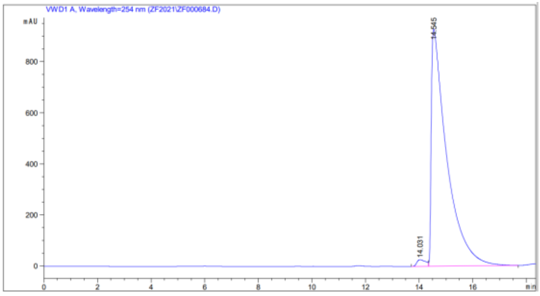

Peak #	RetTime [min]	Type	Width [min]	Area [mAU*s]	Height [mAU]	Area %
1	13.592	BV	0.4196	1.49506e4	494.81204	49.4738
2	14.981	VB	0.5333	1.52686e4	395.65051	50.5262

Peak #	RetTime [min]	Type	Width [min]	Area [mAU*s]	Height [mAU]	Area %
1	14.031	BV	0.3319	592.43164	26.10034	1.5714
2	14.545	VB	0.5527	3.71077e4	927.58350	98.4286

tert-Butyl (S)-2-amino-5-(4-methoxyphenyl)-2-methylpent-4-ynoate (4l):

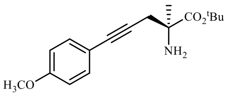

Yellow oil (52.3 mg, 90 %); R$_f$ = 0.24 (petroleum ether/ ethyl acetate = 2:1); the enantiomeric excess was determined to be 98% by HPLC analysis on Daicel Chirapak OD-H column (hexane/isopropanol = 90/10, flow rate 1.0 mL/min, T = 30 ^{o}C), UV 254 nm, t$_R$ (major) 12.959 min, t$_R$ (minor) 11.138 min; $[\alpha]_D^{20}$ = -22.74 (c = 1.0, CH_2Cl_2); **^{1}H NMR (600 MHz, CDCl$_3$)** δ 7.31 (d, *J* = 8.6 Hz, 2H), 6.80 (d, *J* = 8.6 Hz, 2H), 3.79 (s, 3H), 2.83 (d, *J* = 18.0 Hz, 1H), 2.60 (d, *J* = 18.0 Hz, 1H), 1.88 (s, 2H), 1.48 (s, 9H), 1.38 (s, 3H). **^{13}C NMR (151 MHz, CDCl$_3$)** δ 175.45, 159.30, 132.97, 115.56, 113.85, 83.88, 83.02, 81.18, 57.99, 55.23, 32.12, 27.94, 25.90. **HRMS(ESI)** m/z: [M+H]$^+$ Calculated for $C_{17}H_{24}NO_3{}^+$ 290.1751 found 290.1751.

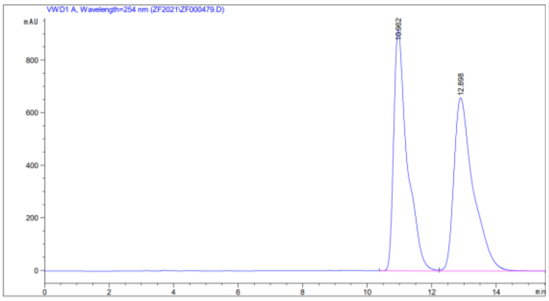

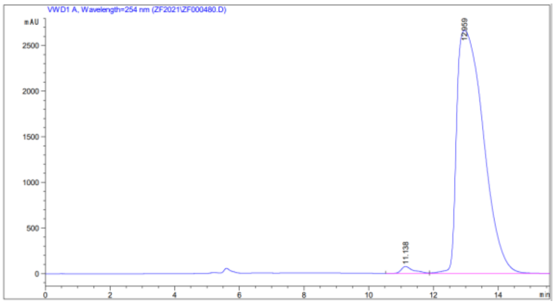

Peak #	RetTime [min]	Type	Width [min]	Area [mAU*s]	Height [mAU]	Area %
1	10.962	BV	0.4232	2.66982e4	915.93243	49.3748
2	12.898	VBA	0.6077	2.73743e4	658.09906	50.6252

Peak #	RetTime [min]	Type	Width [min]	Area [mAU*s]	Height [mAU]	Area %
1	11.138	BV	0.3768	2001.97742	76.11020	1.2952
2	12.959	VBA	0.9201	1.52567e5	2664.96753	98.7048

tert-Butyl (S)-2-amino-2-methyl-5-(4-(methylthio)phenyl)pent-4-ynoate (4m):

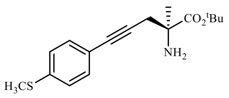

Yellow oil (56.7 mg, 93 %); R_f = 0.24 (petroleum ether/ ethyl acetate = 2:1); the enantiomeric excess was determined to be 97% by HPLC analysis on Daicel Chirapak IA-H column (hexane/isopropanol = 95/5, flow rate 1.0 mL/min, T = 30 °C), UV 254 nm, t_R (major) 10.656 min, t_R (minor) 9.686 min; $[\alpha]_D^{20}$ = -29.44 (c = 1.1, CH_2Cl_2); **^{1}H NMR (600 MHz, $CDCl_3$)** δ 7.28 (d, *J* = 8.2 Hz, 2H), 7.14 (d, *J* = 8.1 Hz, 2H), 2.83 (d, *J* = 18.0 Hz, 1H), 2.61 (d, *J* = 18.0 Hz, 1H), 2.46 (s, 3H), 1.85 (s, 2H), 1.48 (s, 9H), 1.38 (s, 3H). **^{13}C NMR (151 MHz, $CDCl_3$)** δ 175.37, 138.75, 131.91, 126.01, 119.83, 85.62, 82.91, 81.24, 57.97, 32.17, 27.94, 25.92, 15.51. **HRMS(ESI)** m/z: $[M+H]^+$ Calculated for $C_{17}H_{24}NO_2S^+$ 306.1522 found 306.1523.

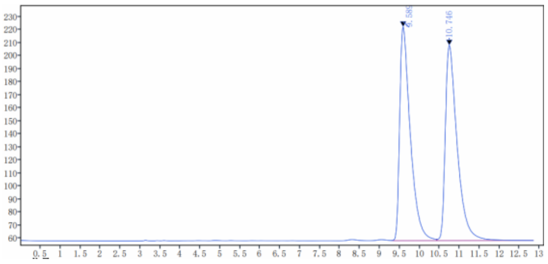

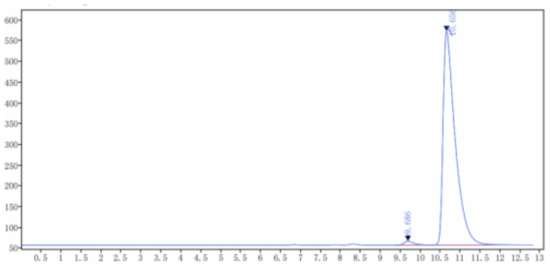

RetTime [min]	Type	Width[min]	Area[mAU*s]	Height[mAU]	Area%
9.589	VV	1.1424	3042.9480	163.8775	49.6501
10.746	VBA	2.4240	3085.8361	149.7230	50.3499

RetTime [min]	Type	Width[min]	Area[mAU*s]	Height[mAU]	Area%
9.686	BB	0.9357	162.1188	9.3742	1.4679
10.656	BB	2.1050	10882.1022	514.8735	98.5321

tert-Butyl (S)-2-amino-5-(3,4-dimethylphenyl)-2-methylpent-4-ynoate (4n):

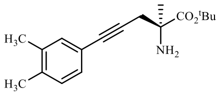

Yellow solid (53.2 mg, 93 %); m.p. = 36-38 °C; R_f = 0.24 (petroleum ether/ ethyl acetate = 2:1); the enantiomeric excess was determined to be 97% by HPLC analysis on Daicel Chirapak OD-H column (hexane/isopropanol = 90/10, flow rate 1.0 mL/min, T = 30 °C), UV 254 nm, t_R (major) 11.078 min, t_R (minor) 9.970 min; $[\alpha]_D^{20}$ = -26.65 (c = 1.1, CH_2Cl_2); **^{1}H NMR (600 MHz, $CDCl_3$)** δ 7.16 (s, 1H), 7.11 (d, *J* = 7.7 Hz, 1H), 7.03 (d, *J* = 7.7 Hz, 1H), 2.83 (d, *J* = 18.0 Hz, 1H), 2.60 (d, *J* = 18.0 Hz, 1H), 2.23 (s, 3H), 2.21 (s, 3H), 1.85 (s, 2H), 1.48 (s, 9H), 1.38 (s, 3H). **^{13}C NMR (151 MHz, $CDCl_3$)** δ 175.45, 136.67, 136.45, 132.69, 129.51, 129.03, 120.64, 84.39, 83.42, 81.19, 57.98, 32.14, 27.94, 25.94, 19.63, 19.50. **HRMS(ESI)** m/z: $[M+H]^+$ Calculated for $C_{18}H_{26}NO_2^+$ 288.1958 found 288.1959.

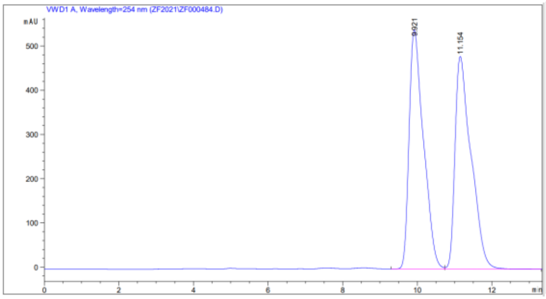

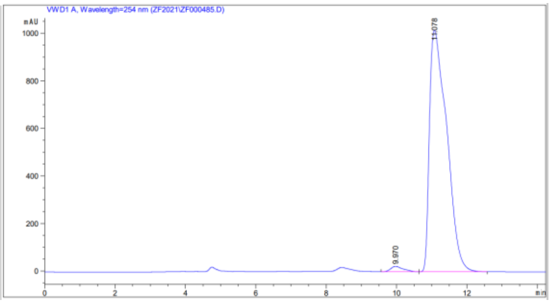

Peak #	RetTime [min]	Type	Width [min]	Area [mAU*s]	Height [mAU]	Area %
1	9.921	BV	0.3889	1.44429e4	540.65228	50.1804
2	11.154	VBA	0.4284	1.43390e4	480.45325	49.8196

Peak #	RetTime [min]	Type	Width [min]	Area [mAU*s]	Height [mAU]	Area %
1	9.970	BB	0.3541	554.13611	22.75414	1.5790
2	11.078	BB	0.4839	3.45399e4	1016.53900	98.4210

tert-Butyl (S)-2-amino-5-(benzo[d][1,3]dioxol-5-yl)-2-methylpent-4-ynoate (4o):

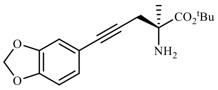

Yellow oil (38.2 mg, 63 %); R_f = 0.27 (petroleum ether/ ethyl acetate = 1:1); the enantiomeric excess was determined to be 95% by HPLC analysis on Daicel Chirapak OD-H column (hexane/isopropanol = 95/5, flow rate 1.0 mL/min, T = 30 °C), UV 254 nm, t_R (major) 14.118 min, t_R (minor) 13.301 min; $[\alpha]_D^{20}$ = -26.40 (c = 0.76, CH_2Cl_2); **^{1}H NMR (600 MHz, $CDCl_3$)** δ 6.89 (d, *J* = 8.0 Hz, 1H), 6.82 (s, 1H), 6.71 (d, *J* = 8.0 Hz, 1H), 5.94 (s, 2H), 2.81 (d, *J* = 18.0 Hz, 1H), 2.58 (d, *J* = 18.0 Hz, 1H), 1.83 (s, 2H), 1.48 (s, 9H), 1.37 (s, 3H). **^{13}C NMR (151 MHz, $CDCl_3$)** δ 175.43, 147.58, 147.35, 126.10, 116.72, 111.68, 108.34, 101.21, 83.78, 83.03, 81.24, 58.00, 32.11, 27.98, 25.93. **HRMS(ESI)** m/z: $[M+H]^+$ Calculated for $C_{17}H_{22}NO_4$ 304.1543 found 304.1544.

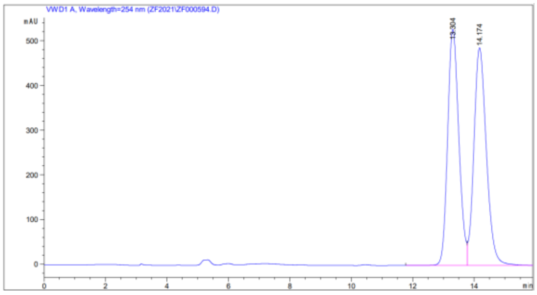

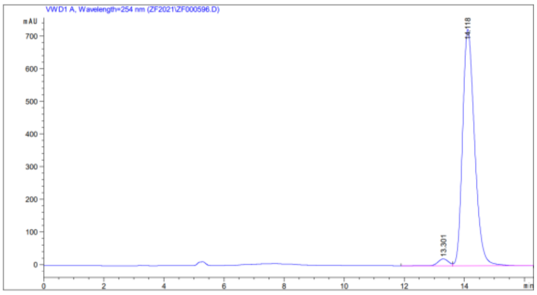

Peak #	RetTime [min]	Type	Width [min]	Area [mAU*s]	Height [mAU]	Area %
1	13.304	BV	0.3924	1.33414e4	528.17267	49.2154
2	14.174	VBA	0.4363	1.37667e4	486.90533	50.7846

Peak #	RetTime [min]	Type	Width [min]	Area [mAU*s]	Height [mAU]	Area %
1	13.301	BV	0.3764	508.72000	21.06913	2.4788
2	14.118	VBA	0.4263	2.00139e4	723.33673	97.5212

tert-Butyl (S)-2-amino-5-(3-fluoro-4-methylphenyl)-2-methylpent-4-ynoate (4p):

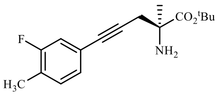

Yellow solid (39.6 mg, 68 %); R_f = 0.24 (petroleum ether/ ethyl acetate = 2:1); the enantiomeric excess was determined to be 96% by HPLC analysis on Daicel Chirapak OD-H column (hexane/isopropanol = 95/5, flow rate 1.0 mL/min, T = 30 °C), UV 254 nm, t_R (major) 8.094 min, t_R (minor) 7.591 min; $[\alpha]_D^{20}$ = -26.40 (c = 0.76, CH_2Cl_2); **^{1}H NMR**

(600 MHz, $CDCl_3$) δ 7.10 – 7.03 (m, 2H), 7.01 (d, *J* = 10.3 Hz, 1H), 2.83 (d, *J* = 18.0 Hz, 1H), 2.60 (d, *J* = 18.0 Hz, 1H), 2.25 (s, 3H), 1.85 (s, 2H), 1.48 (s, 9H), 1.38 (s, 3H). **^{13}C NMR (151 MHz, $CDCl_3$)** δ 175.33, 161.52, 159.90, 131.24, 131.20, 127.25, 127.23, 125.24, 125.12, 122.36, 122.30, 118.06, 117.90, 85.72, 82.19, 81.30, 57.94, 32.04, 27.93, 25.92, 14.48, 14.45. **^{19}F NMR (565 MHz, $CDCl_3$)** δ -117.50. **HRMS(ESI)** m/z: $[M+H]^+$ Calculated for $C_{17}H_{23}FNO_2$ 292.1707 found 292.1709.

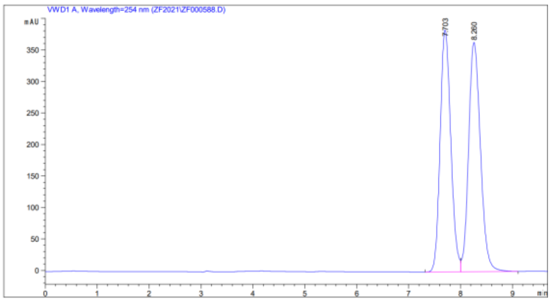

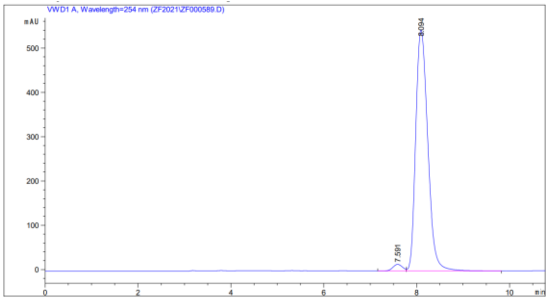

Peak #	RetTime [min]	Type	Width [min]	Area [mAU*s]	Height [mAU]	Area %
1	7.703	BV	0.2250	5485.74365	383.88849	48.7517
2	8.260	VB	0.2474	5766.67432	364.16974	51.2483

Peak #	RetTime [min]	Type	Width [min]	Area [mAU*s]	Height [mAU]	Area %
1	7.591	BV	0.2207	208.35359	14.83847	2.0968
2	8.094	VB	0.2816	9728.35156	543.89111	97.9032

tert-Butyl (S)-2-amino-2-methyl-5-(naphthalen-2-yl)pent-4-ynoate (4q):

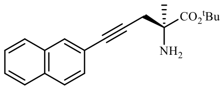

Yellow solid (55.8 mg, 90 %); R_f = 0.27 (petroleum ether/ ethyl acetate = 2:1); the enantiomeric excess was determined to be 94% by HPLC analysis on Daicel Chirapak AD-H column (hexane/isopropanol = 90/10, flow rate 1.0 mL/min, T = 30 °C), UV 254 nm, t_R (major) 7.452 min, t_R (minor) 8.873 min; $[\alpha]_D^{20}$ = -29.79 (c = 1.1, CH_2Cl_2); **^{1}H NMR (600 MHz, $CDCl_3$)** δ 7.89 (s, 1H), 7.79-7.73 (m, 3H), 7.48 – 7.41 (m, 3H), 2.89 (d, *J* = 18.0 Hz, 1H), 2.67 (d, *J* = 18.0 Hz, 1H), 1.89 (s, 2H), 1.50 (s, 9H), 1.42 (s, 3H). **^{13}C NMR (151 MHz, $CDCl_3$)** δ 175.43, 133.00, 132.65, 131.30, 128.59, 127.86, 127.71, 127.63, 126.44, 120.72, 85.95, 83.59, 81.30, 58.04, 32.24, 27.97, 25.98. **HRMS(ESI)** m/z: $[M+H]^+$ Calculated for $C_{20}H_{24}NO_2$ 310.1802 found 310.1802.

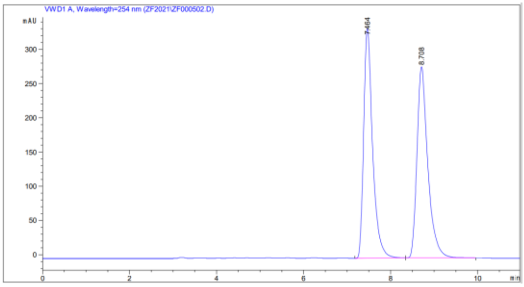

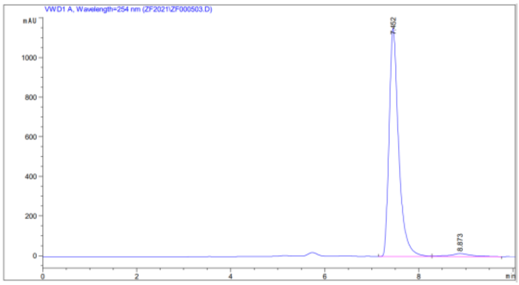

Peak #	RetTime [min]	Type	Width [min]	Area [mAU*s]	Height [mAU]	Area %
1	7.464	BB	0.2114	4777.07275	335.33356	49.6800
2	8.708	BB	0.2561	4838.61328	279.22552	50.3200

Peak #	RetTime [min]	Type	Width [min]	Area [mAU*s]	Height [mAU]	Area %
1	7.452	BV	0.2096	1.62175e4	1150.76782	97.0876
2	8.873	VB	0.4373	486.49283	15.38860	2.9124

tert-Butyl (S)-2-amino-2-methyl-5-(naphthalen-1-yl)pent-4-ynoate (4r):

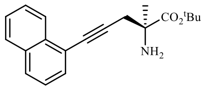

Yellow oil (36.1 mg, 58 %); R_f = 0.20 (petroleum ether/ ethyl acetate = 2:1); the enantiomeric excess was determined to be 98% by HPLC analysis on Daicel Chirapak ID-H column (hexane/isopropanol = 95/5, flow rate 0.8 mL/min, T = 30 °C), UV 254 nm, t_R (major) 18.733 min, t_R (minor) 18.170 min; $[\alpha]_D^{20}$ = -19.21 (c = 0.72, CH_2Cl_2); **^{1}H NMR (600 MHz, CDCl$_3$)** δ 8.31 (d, *J* = 8.2 Hz, 1H), 7.82 (d, *J* = 8.0 Hz, 1H), 7.78 (d, *J* = 8.2 Hz, 1H), 7.61 (d, *J* = 7.0 Hz, 1H), 7.54 (t, *J* = 7.4 Hz, 1H), 7.49 (t, *J* = 7.4 Hz, 1H), 7.38 (t, *J* = 7.7 Hz, 1H), 3.01 (d, *J* = 18.0 Hz, 1H), 2.79 (d, *J* = 18.0 Hz, 1H), 1.90 (s, 2H), 1.49 (s, 9H), 1.46 (s, 3H). **^{13}C NMR (151 MHz, CDCl$_3$)** δ 175.48, 133.46, 133.18, 130.35, 128.32, 128.19, 126.61, 126.29, 126.28, 125.16, 121.11, 90.51, 81.37, 81.32, 58.08, 32.41, 27.99, 26.13. **HRMS(ESI)** m/z: $[M+H]^+$ Calculated for $C_{20}H_{24}NO_2$ 310.1802 found 310.1802.

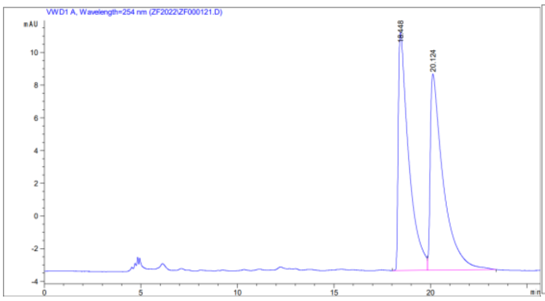

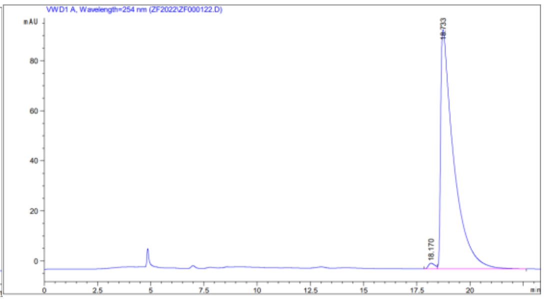

Peak #	RetTime [min]	Type	Width [min]	Area [mAU*s]	Height [mAU]	Area %
1	18.448	BV	0.5242	545.18726	14.56301	49.2021
2	20.124	VB	0.6495	562.87073	11.99556	50.7979

Peak #	RetTime [min]	Type	Width [min]	Area [mAU*s]	Height [mAU]	Area %
1	18.170	BV	0.3324	47.35405	2.22943	1.1521
2	18.733	VB	0.5781	4062.79956	95.47116	98.8479

tert-Butyl (S)-2-amino-5-(9H-fluoren-2-yl)-2-methylpent-4-ynoate (4s):

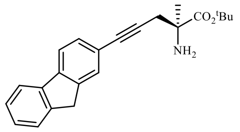

Yellow solid (63.4 mg, 91 %); m.p. = 87-90 °C; R_f = 0.34 (petroleum ether/ ethyl acetate = 1:1); the enantiomeric excess was determined to be 96% by HPLC analysis on Daicel Chirapak AD-H column (hexane/isopropanol = 90/10, flow rate 1.0 mL/min, T = 30 °C), UV 254 nm, t_R (major) 7.629 min, t_R (minor) 8.396 min; $[\alpha]_D^{20}$ = -29.71 (c = 1.3, CH_2Cl_2); **^{1}H NMR (600 MHz, CDCl$_3$)** δ 7.73 (d, *J* = 7.6 Hz, 1H), 7.67 (d, *J* = 7.9 Hz, 1H), 7.54 (s, 1H), 7.51 (d, *J* = 7.4 Hz, 1H), 7.39 (d, *J* = 7.8 Hz, 1H), 7.35 (t, *J* = 7.4 Hz, 1H), 7.32 – 7.26 (m, 1H), 3.84 (s, 2H), 2.87 (d, *J* = 18.0 Hz, 1H), 2.65 (d, *J* = 18.0 Hz, 1H), 1.88 (s, 2H), 1.50 (s, 9H), 1.40 (s, 3H). **^{13}C NMR (151 MHz, CDCl$_3$)** δ 175.46, 143.48, 143.10, 141.52, 141.12, 130.48, 128.21, 127.04, 126.88, 125.05, 121.47, 120.09, 119.65, 85.42, 83.92, 81.26, 58.05, 36.70, 32.26, 27.99, 25.97. **HRMS(ESI)** m/z: $[M+H]^+$ Calculated for $C_{23}H_{26}NO_2$ 348.1958 found

348.1959.

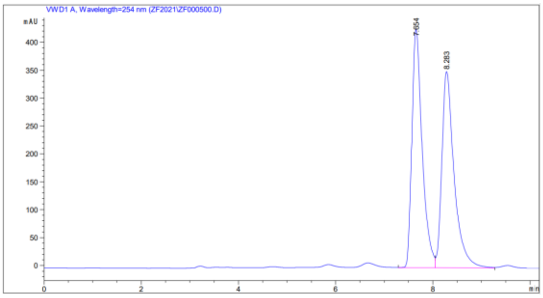

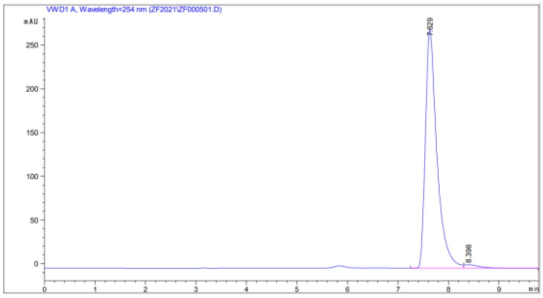

Peak #	RetTime [min]	Type	Width [min]	Area [mAU*s]	Height [mAU]	Area %
1	7.654	VV	0.2188	6298.38623	426.77557	50.5515
2	8.283	VV	0.2555	6160.95557	351.58682	49.4485

Peak #	RetTime [min]	Type	Width [min]	Area [mAU*s]	Height [mAU]	Area %
1	7.629	BV	0.2313	4320.87549	272.99789	98.0920
2	8.396	VBA	0.3085	84.04588	3.87352	1.9080

tert-Butyl (S)-2-amino-2-methyl-5-(thiophen-3-yl)pent-4-ynoate (4t):

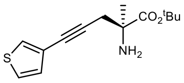

Yellow oil (40.1 mg, 76 %); R_f = 0.20 (petroleum ether/ ethyl acetate = 3:1); the enantiomeric excess was determined to be 95% by HPLC analysis on Daicel Chirapak AD-H column (hexane/isopropanol = 90/10, flow rate 1.0 mL/min, T = 30 °C), UV 254 nm, t_R (major) 5.906 min, t_R (minor) 6.455 min; $[\alpha]_D^{20}$ = -24.94 (c = 0.79, CH_2Cl_2); **^{1}H NMR (600 MHz, $CDCl_3$)** δ 7.34 (d, *J* = 1.5 Hz, 1H), 7.22 (dd, *J* = 4.5, 2.9 Hz, 1H), 7.04 (d, *J* = 4.8 Hz, 1H), 2.82 (d, *J* = 18.0 Hz, 1H), 2.60 (d, *J* = 18.0 Hz, 1H), 1.85 (s, 2H), 1.49 (s, 9H), 1.38 (s, 3H). **^{13}C NMR (151 MHz, $CDCl_3$)** δ 175.39, 129.96, 128.15, 125.07, 122.42, 85.11, 81.28, 78.28, 57.96, 32.13, 27.96, 25.95. **HRMS(ESI)** m/z: $[M+H]^+$ Calculated for $C_{14}H_{20}NO_2S$ 266.1209 found 266.1210.

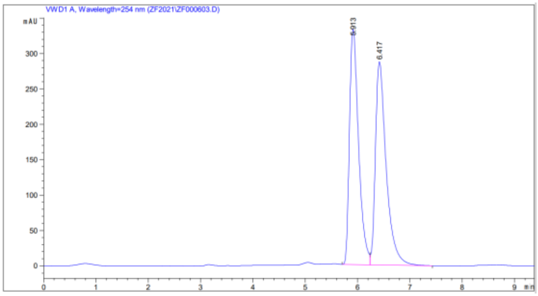

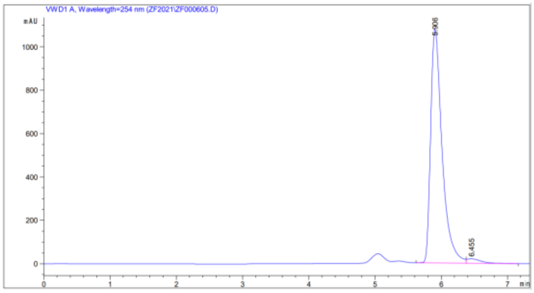

Peak #	RetTime [min]	Type	Width [min]	Area [mAU*s]	Height [mAU]	Area %
1	5.913	BV	0.1832	4014.44019	332.24207	49.4066
2	6.417	VB	0.2136	4110.87939	287.19714	50.5934

Peak #	RetTime [min]	Type	Width [min]	Area [mAU*s]	Height [mAU]	Area %
1	5.906	BV	0.1805	1.28955e4	1076.96106	97.6885
2	6.455	VBA	0.2177	305.12897	20.46399	2.3115

tert-Butyl (S)-2-amino-5-cyclopropyl-2-methylpent-4-ynoate (4u):

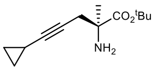

Yellow oil (40.5 mg, 91 %); R_f = 0.20 (petroleum ether/ ethyl acetate = 2.5:1); the enantiomeric excess was determined to be 96% by HPLC analysis on Daicel Chirapak ID-H column (hexane/isopropanol = 90/10, flow rate 1.0 mL/min, T = 30 °C), UV 254 nm,

t_R (major) 11.536 min, t_R (minor) 12.928 min; $[\alpha]_D^{20}$ = -15.44 (c = 0.68, CH_2Cl_2); **^{1}H NMR (600 MHz, $CDCl_3$)** δ 2.55 (d, *J* = 18.0 Hz, 1H), 2.31 (d, *J* = 18.0 Hz, 1H), 1.83 (s, 2H), 1.47 (s, 9H), 1.29 (s, 3H), 1.29-1.18 (m, 1H), 0.75 – 0.66 (m, 2H), 0.62 – 0.57 (m, 2H). **^{13}C NMR (151 MHz, $CDCl_3$)** δ 175.59, 86.36, 81.02, 70.85, 57.88, 31.51, 27.93, 25.79, 8.04, -0.51. **HRMS(ESI)** m/z: $[M+H]^+$ Calculated for $C_{13}H_{22}NO_2$ 224.1645 found 224.1646.

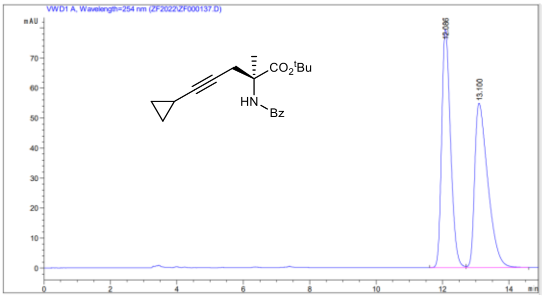

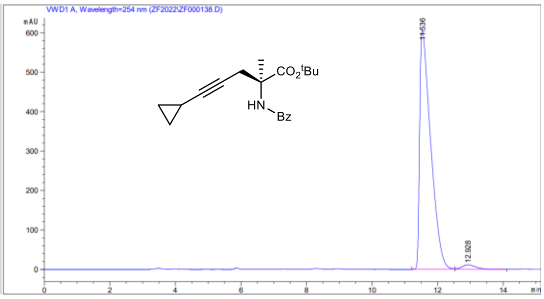

Peak #	RetTime [min]	Type	Width [min]	Area [mAU*s]	Height [mAU]	Area %
1	12.086	BV	0.2856	1467.44690	79.40241	49.3702
2	13.100	VB	0.4168	1504.88733	54.76521	50.6298

Peak #	RetTime [min]	Type	Width [min]	Area [mAU*s]	Height [mAU]	Area %
1	11.536	BV	0.3453	1.41759e4	607.05640	97.7595
2	12.928	VB	0.4438	324.89572	11.28508	2.2405

tert-Butyl (S)-2-amino-2-methylhept-4-ynoate (4v):

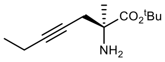

Colorless oil (38.1 mg, 90 %); R_f = 0.33 (petroleum ether/ ethyl acetate = 2.5:1); the enantiomeric excess was determined to be 91% by HPLC analysis on Daicel Chirapak ID-H column (hexane/isopropanol = 95/5, flow rate 1.0 mL/min, T = 30 °C), UV 220 nm, t_R (major) 7.384 min, t_R (minor) 6.976 min; $[\alpha]_D^{20}$ = -8.71 (c = 0.44, CH_2Cl_2); **^{1}H NMR (600 MHz, $CDCl_3$)** δ 2.58 (d, *J* = 18.0 Hz, 1H), 2.34 (d, *J* = 18.0 Hz, 1H), 2.15 (q, *J* = 9.0 Hz, 2H), 1.82 (s, 2H), 1.47 (s, 9H), 1.31 (s, 3H), 1.10 (t, *J* = 7.5 Hz, 3H). **^{13}C NMR (151 MHz, $CDCl_3$)** δ 175.65, 84.62, 80.99, 74.82, 57.80, 31.42, 27.90, 25.78, 14.16, 12.33. **HRMS(ESI)** m/z: $[M+H]^+$ Calculated for $C_{12}H_{22}NO_2$ 212.1645 found 212.1644.

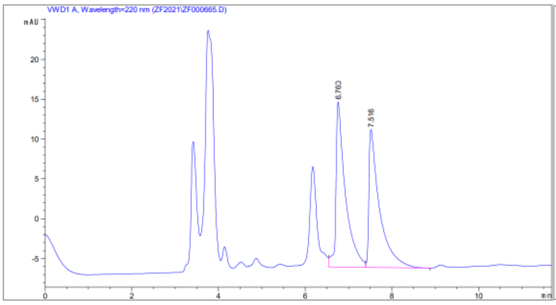

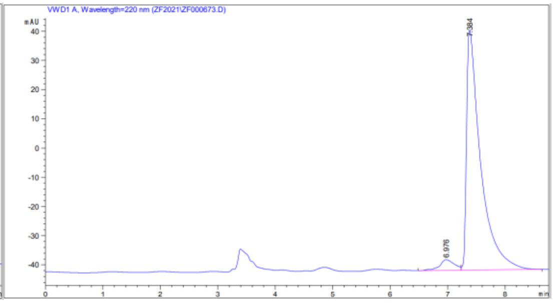

Peak #	RetTime [min]	Type	Width [min]	Area [mAU*s]	Height [mAU]	Area %
1	6.760	VV	0.2063	312.22739	20.72888	51.4536
2	7.516	VB	0.2322	294.58606	17.29504	48.5464

Peak #	RetTime [min]	Type	Width [min]	Area [mAU*s]	Height [mAU]	Area %
1	6.976	BV	0.2635	65.57275	3.62546	4.4898
2	7.384	VB	0.2403	1394.91016	82.19255	95.5102

tert-Butyl (S)-2-amino-2-methyloct-4-ynoate (4w):

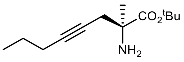

Yellow oil (42.4 mg, 94 %); R_f = 0.33 (petroleum ether/ ethyl acetate = 2:1); the enantiomeric excess was determined to be 91% by HPLC analysis on Daicel Chirapak ID-H column (hexane/isopropanol = 95/5, flow rate 1.0 mL/min, T = 30 °C), UV 220 nm, t_R (major) 6.818 min, t_R (minor) 6.398 min; $[\alpha]_D^{20}$ = -11.55 (c = 0.85, CH_2Cl_2); **^{1}H NMR (600 MHz, $CDCl_3$)** δ 2.59 (d, *J* = 18.0 Hz, 1H), 2.36 (d, *J* = 18.0 Hz, 1H), 2.12 (d, *J* = 6.1 Hz, 2H), 1.82 (s, 2H), 1.52 – 1.48 (m, 2H), 1.47 (s, 9H), 1.31 (s, 3H), 0.96 (t, *J* = 7.3 Hz, 3H). **^{13}C NMR (151 MHz, $CDCl_3$)** δ 175.79, 83.10, 80.97, 75.58, 57.78, 31.44, 27.89, 25.85, 22.33, 20.68, 13.44. **HRMS(ESI)** m/z: $[M+H]^+$ Calculated for $C_{13}H_{24}NO_2$ 226.1802 found 226.1801.

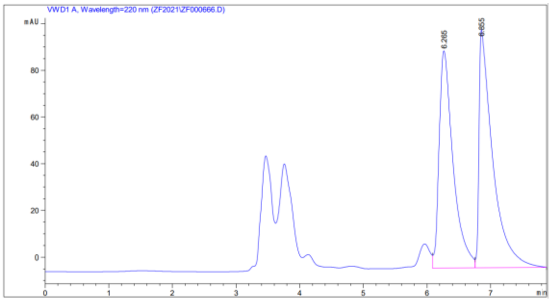

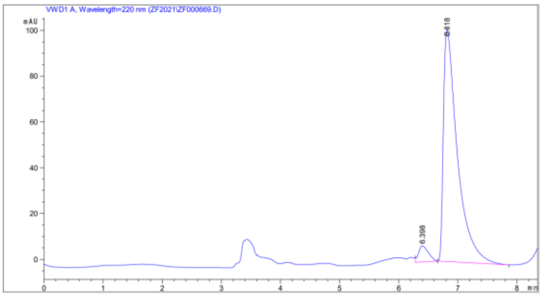

Peak #	RetTime [min]	Type	Width [min]	Area [mAU*s]	Height [mAU]	Area %
1	6.265	VV	0.2305	1419.19653	92.98288	48.4237
2	6.855	VBA	0.2155	1511.59131	101.79702	51.5763

Peak #	RetTime [min]	Type	Width [min]	Area [mAU*s]	Height [mAU]	Area %
1	6.398	VB	0.1768	79.36359	6.95673	4.5690
2	6.818	BB	0.2391	1657.65039	101.25554	95.4310

tert-Butyl (S)-2-amino-2-methyldec-4-ynoate (4x):

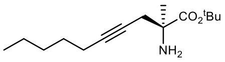

Yellow oil (48.2 mg, 95 %); R_f = 0.38 (petroleum ether/ ethyl acetate = 2.5:1); the enantiomeric excess was determined to be 92% by HPLC analysis on Daicel Chirapak ID-H column (hexane/isopropanol = 95/5, flow rate 1.0 mL/min, T = 30 °C), UV 220 nm, t_R (major) 7.738 min, t_R (minor) 7.256 min; $[\alpha]_D^{20}$ = -15.25 (c = 96, CH_2Cl_2); **^{1}H NMR (600 MHz, $CDCl_3$)** δ 2.59 (d, *J* = 12.0 Hz, 1H), 2.35 (d, *J* = 12.0 Hz, 1H), 2.13 (t, *J* = 6.6 Hz, 2H), 1.87 (s, 2H), 1.51 – 1.43 (m, 11H), 1.39 – 1.28 (m, 7H), 0.89 (t, *J* = 6.9 Hz, 3H). **^{13}C NMR (151 MHz, $CDCl_3$)** δ 175.61, 83.31, 80.96, 75.38, 57.79, 31.41, 31.01, 28.62, 27.89, 25.80, 22.15, 18.62, 13.91. **HRMS(ESI)** m/z: $[M+H]^+$ Calculated for $C_{15}H_{28}NO_2$ 254.2115 found 254.2115.

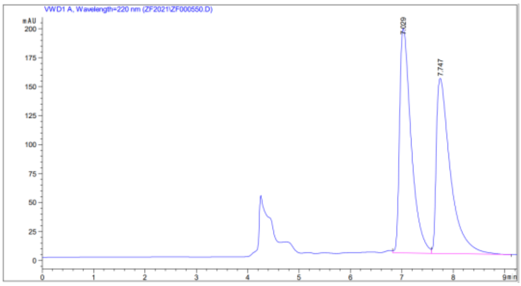

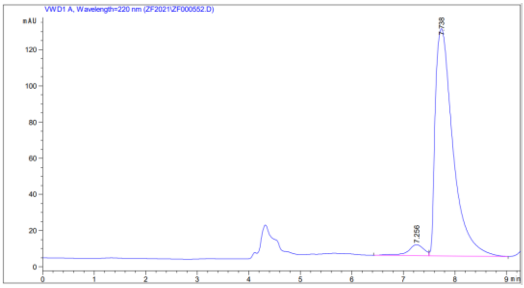

Peak #	RetTime [min]	Type	Width [min]	Area [mAU*s]	Height [mAU]	Area %
1	7.029	VV	0.2353	3033.21973	193.55099	51.3460
2	7.747	VB	0.2803	2874.19385	151.06850	48.6540

Peak #	RetTime [min]	Type	Width [min]	Area [mAU*s]	Height [mAU]	Area %
1	7.256	BV	0.3264	130.89221	5.98949	4.1959
2	7.738	VB	0.3653	2988.61157	125.47501	95.8041

(*S*)-3,7-dimethyloct-6-en-1-yl 4-((S)-4-amino-5-(tert-butoxy)-4-methyl-5-oxopent-1-yn-1-yl) Benzoate (4y):

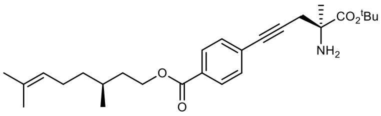

Yellow oil (37.2 mg, 44 %); R_f = 0.47 (petroleum ether/ ethyl acetate = 1:1); $[\alpha]_D^{20}$ = -17.74 (c = 62, CH_2Cl_2); 20:1 dr (dr is measured by^{1}H NMR), **^{1}H NMR (600 MHz, CDCl$_3$)** δ 7.94 (d, *J* = 7.9 Hz, 2H), 7.42 (d, *J* = 7.9 Hz, 2H), 5.09 (t, *J* = 6.7 Hz, 1H), 4.40 – 4.26 (m, 2H), 2.86 (d, *J* = 18.0 Hz, 1H), 2.65 (d, *J* = 18.0 Hz, 1H), 1.98-2.03 (m, 2H), 1.82 (s, 2H), 1.82 – 1.77 (m, 1H), 1.67 (s, 3H), 1.66 – 1.62 (m, 1H), 1.60 (s, 3H), 1.59 – 1.54 (m, 1H), 1.49 (s, 9H), 1.40 (s,3H), 1.38 (d, *J* = 5.5 Hz, 1H), 1.28 – 1.19 (m, 1H), 0.97 (d, *J* = 6.6 Hz, 3H). **^{13}C NMR (151 MHz, CDCl$_3$)** δ 175.24, 166.10, 131.50, 131.37, 129.67, 129.37, 127.99, 124.54, 88.91, 82.65, 81.37, 63.63, 57.96, 36.97, 35.50, 32.17, 29.56, 27.93, 25.94, 25.67, 25.38, 19.50, 17.63. **HRMS(ESI)** m/z: $[M+H]^+$ Calculated for $C_{27}H_{40}NO_4$ 442.2952 found 442.2950.

2-((1S,5R)-6,6-dimethylbicyclo [3.1.1] hept-2-en-2-yl) ethyl 4-((S)-4-amino-5-(tert-butoxy)-4-methyl-5-oxopent-1-yn-1-yl) benzoate (4z):

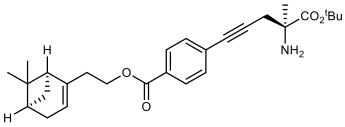

Yellow oil (43.1 mg, 48 %); R_f = 0.3 (petroleum ether/ ethyl acetate = 2:1); $[\alpha]_D^{20}$ = -30.28 (c = 26, CH_2Cl_2); 20:1 dr (dr is measured by^{1}H NMR), **^{1}H NMR (600 MHz,** CDCl$_3$) δ 7.94 (d, *J* = 8.2 Hz, 2H), 7.42 (d, *J* = 8.2 Hz, 2H), 5.35 (s, 1H), 4.36 – 4.28 (m, 2H), 2.86 (d, *J* = 12.0 Hz, 1H), 2.65 (d, *J* = 12.0 Hz, 1H), 2.45 – 2.34 (m, 3H), 2.23 (q, *J* = 17.6 Hz, 2H), 2.11-2.09 (m, 2H), 1.84 (s, 2H), 1.49 (s, 9H), 1.40 (s, 3H), 1.27 (s, 3H), 1.17 (d, *J* = 8.6 Hz, 1H), 0.83 (s, 3H). **^{13}C NMR (151 MHz, CDCl$_3$)** δ 175.23, 165.99, 144.19, 131.50, 129.59, 129.38, 128.00, 118.96, 88.91, 82.66, 81.37, 63.44, 57.95, 45.81, 40.76, 38.01, 36.03, 32.16, 31.68, 31.38, 27.93, 26.25, 25.93, 21.13. **HRMS(ESI)** m/z: $[M+H]^+$ Calculated for $C_{28}H_{38}NO_4$ 452.2795 found 452.2798.

tert-Butyl (R)-2-amino-2,5-diphenylpent-4-ynoate (5a):

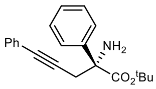

White solid (56.7 mg, 88 %); R_f = 0.43 (petroleum ether/ ethyl acetate = 4:1); the enantiomeric excess was determined to be 86% by HPLC analysis on Daicel Chirapak AD-H column (hexane/isopropanol = 90/10, flow rate 1.0 mL/min, T = 30 °C), UV 254 nm, t_R (major) 7.135 min, t_R (minor) 7.796 min; $[\alpha]_D^{20}$ = -5.42 (c = 1.1, CH_2Cl_2); **^{1}H NMR (600 MHz, $CDCl_3$)** δ 7.59 (d, *J* = 7.4 Hz, 2H), 7.37-7.35 (m, 4H), 7.31 – 7.23 (m, 4H), 3.31 (d, *J* = 18.0 Hz, 1H), 2.92 (d, *J* = 18.0 Hz, 1H), 2.20 (s, 2H), 1.48 (s, 9H). **^{13}C NMR (151 MHz, $CDCl_3$)** δ 173.64, 142.23, 131.66, 128.40, 128.24, 127.96, 127.69, 125.45, 123.33, 85.72, 83.27, 82.04, 63.86, 31.94, 27.90. **HRMS(ESI)** m/z: $[M+H]^+$ Calculated for $C_{21}H_{24}NO_2$ 322.1802 found 322.1803.

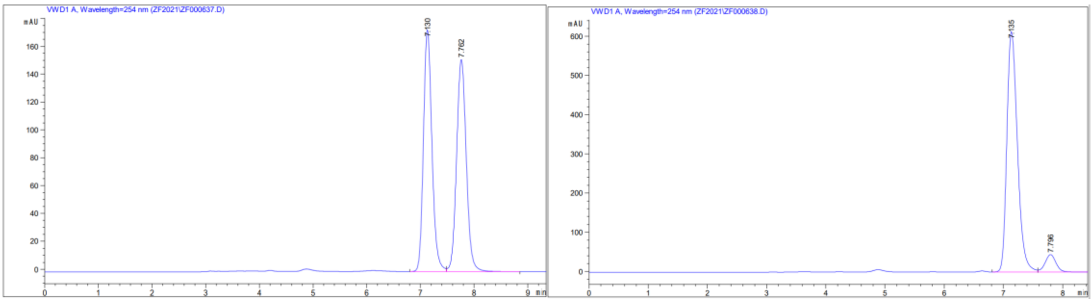

Peak #	RetTime [min]	Type	Width [min]	Area [mAU*s]	Height [mAU]	Area %
1	7.130	BV	0.1694	1909.62024	173.32849	49.9265
2	7.762	VB	0.1964	1915.24414	152.12140	50.0735

Peak #	RetTime [min]	Type	Width [min]	Area [mAU*s]	Height [mAU]	Area %
1	7.135	VV	0.1929	7612.41357	613.19397	93.0397
2	7.796	VBA	0.1955	569.48163	44.64599	6.9603

tert-Butyl (R)-2-amino-5-phenyl-2-(p-tolyl)pent-4-ynoate (5b):

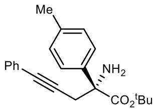

White solid (55.5 mg, 83 %); m.p. = 45-47 °C; R_f = 0.25 (petroleum ether/ ethyl acetate = 5:1); the enantiomeric excess was determined to be 89% by HPLC analysis on Daicel Chirapak AD-H column (hexane/isopropanol = 95/5, flow rate 1.0 mL/min, T = 30 °C), UV 254 nm, t_R (major) 10.113 min, t_R (minor) 10.912 min; $[\alpha]_D^{20}$ = -0.81 (c = 0.98, CH_2Cl_2); **^{1}H NMR (600 MHz, $CDCl_3$)** δ 7.46 (d, *J* = 8.2 Hz, 2H), 7.37-7.36 (m, 2H), 7.27-7.26 (m, 3H), 7.16 (d, *J* = 8.0 Hz, 2H), 3.30 (d, *J* = 18.0 Hz, 1H), 2.89 (d, *J* = 18.0 Hz, 1H), 2.34 (s, 3H), 2.23 (s, 2H), 1.48 (s, 9H). **^{13}C NMR (151 MHz, $CDCl_3$)** δ 173.79, 139.30, 137.36, 131.67, 129.10, 128.23, 127.93, 125.33, 123.37, 85.85, 83.20, 81.92, 63.64, 31.97, 27.91, 21.00. **HRMS(ESI)** m/z: $[M+H]^+$ Calculated for $C_{22}H_{26}NO_2$ 336.1958 found 336.1958.

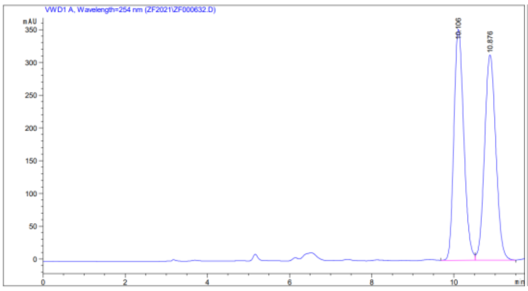

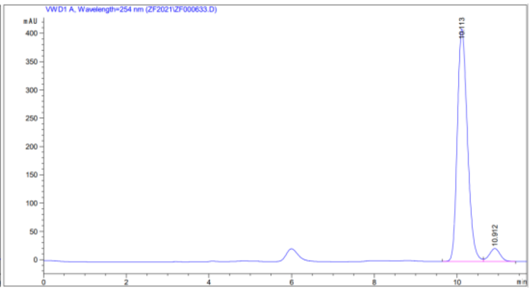

Peak #	RetTime [min]	Type	Width [min]	Area [mAU*s]	Height [mAU]	Area %
1	10.106	BV	0.2611	5862.01025	352.39053	49.5989
2	10.876	VB	0.2991	5956.83252	313.21805	50.4011

Peak #	RetTime [min]	Type	Width [min]	Area [mAU*s]	Height [mAU]	Area %
1	10.113	BV	0.2672	6991.79053	410.56519	94.4344
2	10.912	VB	0.2802	412.06827	22.71413	5.5656

tert-Butyl (R)-2-amino-2-(4-fluorophenyl)-5-phenylpent-4-ynoate (5c):

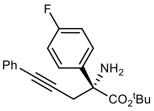

Yellow oil (60.3 mg, 89 %); R_f = 0.42 (petroleum ether/ ethyl acetate = 3:1); the enantiomeric excess was determined to be 80% by HPLC analysis on Daicel Chirapak AD-H column (hexane/isopropanol = 95/5, flow rate 1.0 mL/min, T = 30 °C), UV 254 nm, t_R (major) 9.578 min, t_R (minor) 10.916 min; $[\alpha]_D^{20}$ = -6.76 (c =1.2, CH_2Cl_2); **^{1}H NMR (600 MHz, $CDCl_3$)** δ 7.59-7.57 (m, 2H), 7.38 – 7.32 (m, 2H), 7.27 (dd, *J* = 9.1, 6.0 Hz, 3H), 7.04 (t, *J* = 8.6 Hz, 2H), 3.27 (d, *J* = 16.4 Hz, 1H), 2.89 (d, *J* = 16.4 Hz, 1H), 2.23 (s, 2H), 1.48 (s, 9H). **^{13}C NMR (151 MHz, $CDCl_3$)** δ 173.41, 163.07, 161.44, 138.00, 131.65, 128.26, 128.04, 127.40, 127.34, 123.19, 115.21, 115.07, 85.39, 83.44, 82.23, 63.41, 32.17, 27.87. **^{19}F NMR (565 MHz, $CDCl_3$)** δ -115.04. **HRMS(ESI)** m/z: $[M+H]^+$ Calculated for $C_{21}H_{23}FNO_2$ 340.1707 found 340.1707.

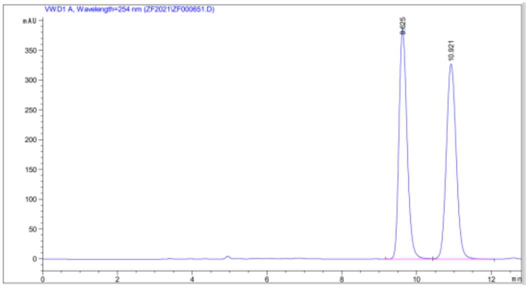

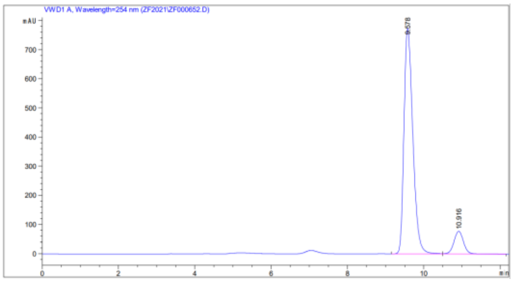

Peak #	RetTime [min]	Type	Width [min]	Area [mAU*s]	Height [mAU]	Area %
1	9.625	BV	0.2356	5833.77637	387.05475	49.3906
2	10.921	VB	0.2855	5977.74121	327.96832	50.6094

Peak #	RetTime [min]	Type	Width [min]	Area [mAU*s]	Height [mAU]	Area %
1	9.578	VV	0.2509	1.24690e4	772.86102	90.2108
2	10.916	VB	0.2727	1353.07397	77.29877	9.7892

tert-Butyl (R)-2-amino-2-(4-chlorophenyl)-5-phenylpent-4-ynoate (5d):

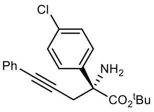

Yellow solid (66.5 mg, 93 %); R_f = 0.32 (petroleum ether/ ethyl acetate = 5:1); the enantiomeric excess was determined to be 79% by HPLC analysis on Daicel Chirapak AD-H column (hexane/isopropanol = 90/10, flow rate 1.0 mL/min, T = 30 °C), UV 254 nm, t_R (major) 7.430 min, t_R (minor) 8.218 min; $[\alpha]_D^{20}$ = +5.25 (c =1.3, CH_2Cl_2); **^{1}H NMR (600 MHz, $CDCl_3$)** δ 7.55 (d, *J* = 8.6 Hz, 2H), 7.36 (dd, *J* = 6.6, 3.0 Hz, 2H), 7.33 (d, *J* = 8.6 Hz, 2H),

7.29 – 7.26 (m, 3H), 3.27 (d, *J* = 18.0 Hz, 1H), 2.88 (d, *J* = 18.0 Hz, 1H), 2.23 (s, 2H), 1.47 (s, 9H). **^{13}C NMR (151 MHz, $CDCl_3$)** δ 173.18, 140.79, 133.62, 131.66, 128.50, 128.27, 128.08, 127.10, 123.15, 85.25, 83.52, 82.33, 63.48, 32.06, 27.87. **HRMS(ESI)** m/z: $[M+H]^+$ Calculated for $C_{21}H_{23}ClNO_2$ 356.1412 found 356.1412.

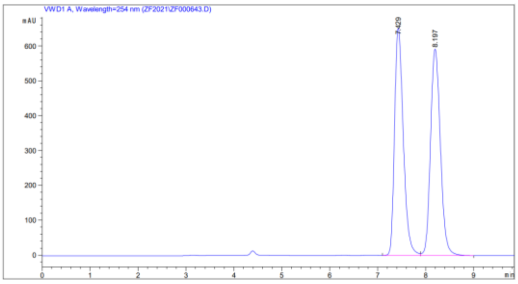

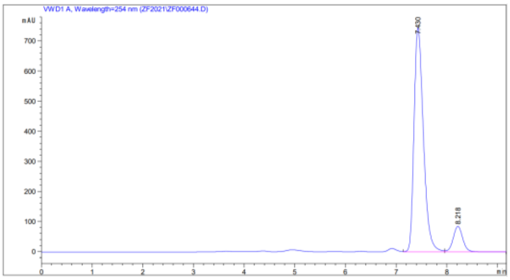

Peak #	RetTime [min]	Type	Width [min]	Area [mAU*s]	Height [mAU]	Area %
1	7.429	BV	0.1975	8251.41309	650.94342	49.4097
2	8.197	VB	0.2249	8448.57910	591.59155	50.5903

Peak #	RetTime [min]	Type	Width [min]	Area [mAU*s]	Height [mAU]	Area %
1	7.430	VV	0.2003	9618.90723	744.46570	89.6131
2	8.218	VBA	0.2026	1114.91052	84.99237	10.3869

tert-Butyl (S)-2-amino-2-benzyl-5-phenylpent-4-ynoate (5e):

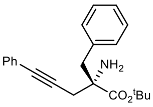

Yellow oil (50.0 mg, 74 %); R_f = 0.40 (petroleum ether/ ethyl acetate = 3:1); the enantiomeric excess was determined to be 96% by HPLC analysis on Daicel Chirapak AD-H column (hexane/isopropanol = 95/5, flow rate 1.0 mL/min, T = 30 °C), UV 254 nm, t_R (major) 7.841 min, t_R (minor) 10.137 min; $[\alpha]_D^{20}$ **=** -15.38 (c =0.96, CH_2Cl_2); **^{1}H NMR (600 MHz, $CDCl_3$)** δ 7.37 (m, 2H), 7.30 – 7.22 (m, 8H), 3.16 (d, *J* = 13.2 Hz, 1H), 2.95 (d, *J*= 18.0 Hz, 1H), 2.88 (d, *J* = 13.2 Hz, 1H), 2.65 (d, *J* = 18.0Hz, 1H), 1.80 (s, 2H), 1.47 (s, 9H). **^{13}C NMR (151 MHz, $CDCl_3$)** δ 174.17, 136.20, 131.64, 130.23, 128.28, 128.23, 127.92, 127.02, 123.40, 85.26, 83.66, 81.67, 61.78, 45.02, 31.32, 28.03. $[M+H]^+$ Calculated for $C_{22}H_{26}NO_2$ 336.1958 found 336.1957.

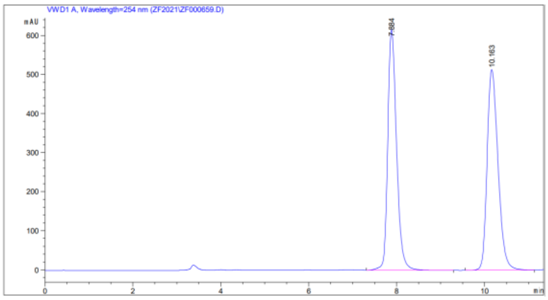

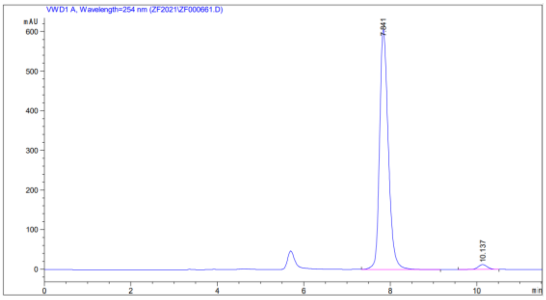

Peak #	RetTime [min]	Type	Width [min]	Area [mAU*s]	Height [mAU]	Area %
1	7.884	BB	0.2130	8539.79004	615.08344	49.1361
2	10.163	BB	0.2694	8840.09082	513.39276	50.8639

Peak #	RetTime [min]	Type	Width [min]	Area [mAU*s]	Height [mAU]	Area %
1	7.841	VB	0.2158	8473.17871	605.13367	97.9029
2	10.137	BB	0.2273	181.49718	12.31585	2.0971

tert-Butyl (R)-2-amino-2-(2-fluorobenzyl)-5-phenylpent-4-ynoate (5f):

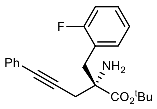

Yellow oil (59.7 mg, 85 %); R_f = 0.32 (petroleum ether/ ethyl acetate = 5:1); the enantiomeric excess was determined to be 94% by HPLC analysis on Daicel Chirapak AD-H column (hexane/isopropanol = 90/10, flow rate 1.0 mL/min, T = 30 °C), UV 254 nm, t_R (major) 6.017 min, t_R (minor) 9.652 min; $[\alpha]_D^{20}$ = -20.85 (c = 0.69, CH_2Cl_2); **^{1}H NMR (600 MHz, $CDCl_3$)** δ 7.37 (dd, *J* = 6.5, 3.0 Hz, 2H), 7.33 – 7.20 (m, 5H), 7.02-7.08 (m, 2H), 3.13 (d, *J* = 13.6 Hz, 1H), 3.04 (d, *J* = 13.6 Hz, 1H), 2.97 (d, *J* = 18.0 Hz, 1H), 2.66 (d, *J* = 18.0 Hz, 1H), 1.84 (s, 2H), 1.46 (s, 9H). **^{13}C NMR (151 MHz, $CDCl_3$)** δ 174.07, 162.33, 160.70, 132.57, 132.54, 131.63, 128.82, 128.77, 128.22, 127.91, 123.84, 123.82, 123.37, 123.28, 115.45, 115.29, 85.28, 83.56, 81.77, 61.59, 37.58, 30.97, 27.90. **^{19}F NMR (565 MHz, $CDCl_3$)** δ -115.11. $[M+H]^+$ Calculated for $C_{22}H_{25}FNO_2$ 354.1864 found 354.1863.

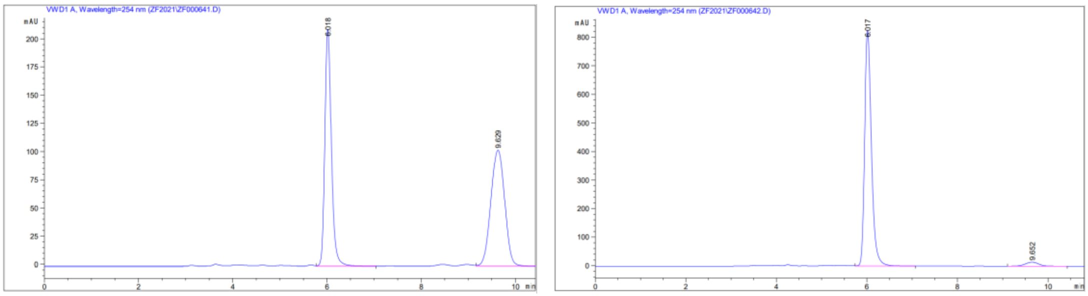

Peak #	RetTime [min]	Type	Width [min]	Area [mAU*s]	Height [mAU]	Area %
1	6.018	VB	0.1477	2028.18616	209.85353	49.1818
2	9.629	VBA	0.3240	2095.66553	102.72674	50.8182

Peak #	RetTime [min]	Type	Width [min]	Area [mAU*s]	Height [mAU]	Area %
1	6.017	VB	0.1615	8627.22559	823.77942	96.8770
2	9.652	BB	0.3228	278.11423	13.78922	3.1230

tert-Butyl (S)-2-amino-2-(3-methylbenzyl)-5-phenylpent-4-ynoate (5g):

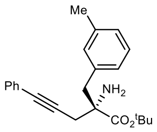

Yellow oil (42.4 mg, 61 %); R_f = 0.28 (petroleum ether/ ethyl acetate = 3:1); the enantiomeric excess was determined to be 96% by HPLC analysis on Daicel Chirapak AD-H column (hexane/isopropanol = 90/10, flow rate 1.0 mL/min, T = 30 °C), UV 254 nm, t_R (major) 5.324 min, t_R (minor) 6.062 min; $[\alpha]_D^{20}$ = -21.78 (c = 0.80, CH_2Cl_2); **^{1}H NMR (600 MHz, $CDCl_3$)** δ 7.40 – 7.35 (m, 2H), 7.27-7.25 (m, 3H), 7.17 (dd, *J* = 10.2, 5.2 Hz, 1H), 7.05 (t, *J* = 6.9 Hz, 3H), 3.14 (d, *J* = 12.0 Hz, 1H), 2.96 (d, *J* = 18.0 Hz, 1H), 2.83 (d, *J* = 12.0 Hz, 1H), 2.65 (d, *J* = 18.0 Hz, 1H), 2.32 (s, 3H), 1.79 (s, 2H), 1.48 (s, 9H). **^{13}C NMR (151 MHz, $CDCl_3$)** δ 174.23, 137.80, 136.03, 131.64, 130.95, 128.22, 128.20, 127.92, 127.78, 127.20, 123.40, 85.26, 83.61, 81.63, 61.79, 44.94, 31.40, 28.03, 21.35. $[M+H]^+$ Calculated for $C_{23}H_{28}NO_2$ 350.2115 found 350.2116.

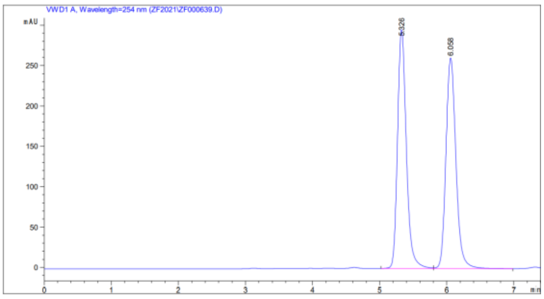

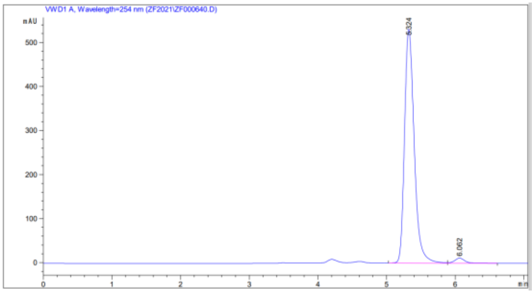

Peak #	RetTime [min]	Type	Width [min]	Area [mAU*s]	Height [mAU]	Area %
1	5.326	VV	0.1400	2696.29248	295.56659	50.0603
2	6.058	VB	0.1611	2689.79272	260.84467	49.9397

Peak #	RetTime [min]	Type	Width [min]	Area [mAU*s]	Height [mAU]	Area %
1	5.324	VV	0.1519	5190.75928	531.18329	97.7293
2	6.062	VB	0.1602	120.60282	11.37288	2.2707

tert-Butyl (S)-2-amino-2-(naphthalen-2-ylmethyl)-5-phenylpent-4-ynoate (5h):

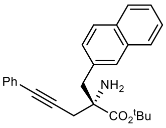

Yellow solid (50.7 mg, 66 %); R_f = 0.32 (petroleum ether/ ethyl acetate = 3:1); the enantiomeric excess was determined to be 96% by HPLC analysis on Daicel Chirapak AD-H column (hexane/isopropanol = 95/5, flow rate 1.0 mL/min, T = 30 °C), UV 254 nm, t_R (major) 12.600 min, t_R (minor) 11.712 min; $[\alpha]_D^{20}$ = -4.5 (c = 1.0, CH_2Cl_2); **^{1}H NMR (600 MHz, CDCl$_3$)** δ 7.81 (d, J = 7.5 Hz, 1H), 7.77 (d, J = 8.1 Hz, 2H), 7.71 (s, 1H), 7.49 – 7.41 (m, 2H), 7.39 (d, J = 8.7 Hz, 3H), 7.27 (s, 3H), 3.35 (d, J = 12.0 Hz, 1H), 3.04 (d, J = 12.0 Hz, 1H), 3.01 (d, J = 18.0 Hz, 1H), 2.71 (d, J = 18.0 Hz, 1H), 1.79 (s, 2H), 1.48 (s, 9H). **^{13}C NMR (151 MHz, CDCl$_3$)** δ 174.22, 133.80, 133.37, 132.55, 131.66, 128.94, 128.46, 128.25, 127.96, 127.85, 127.64, 126.06, 125.67, 123.37, 85.19, 83.75, 81.77, 62.01, 45.13, 31.47, 28.06. $[M+H]^+$ Calculated for $C_{26}H_{28}NO_2$ 386.2115 found 386.2112.

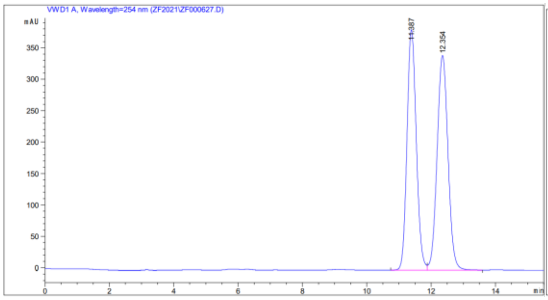

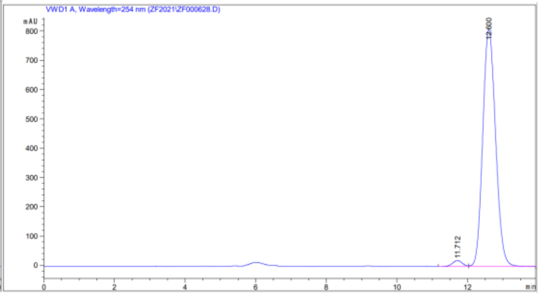

Peak #	RetTime [min]	Type	Width [min]	Area [mAU*s]	Height [mAU]	Area %
1	11.387	BV	0.3125	7662.63721	382.34729	48.8005
2	12.354	VB	0.3694	8039.32910	341.52319	51.1995

Peak #	RetTime [min]	Type	Width [min]	Area [mAU*s]	Height [mAU]	Area %
1	11.712	BV	0.2991	390.30960	20.26223	1.8744
2	12.600	VBA	0.3967	2.04327e4	809.01147	98.1256

tert-Butyl (S)-2-amino-2-phenethyl-5-phenylpent-4-ynoate (5i):

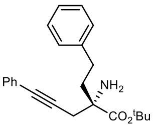

Yellow oil (56.3 mg, 81 %); R_f = 0.29 (petroleum ether/ ethyl acetate = 3:1); the enantiomeric excess was determined to be 86% by HPLC analysis on Daicel Chirapak AD-H column (hexane/isopropanol = 90/10, flow rate 1.0 mL/min, T =

30 °C), UV 254 nm, t_R (major) 6.638 min, t_R (minor) 7.720 min; $[\alpha]_D^{20}$ = -26.63 (c = 0.94, CH_2Cl_2); **^{1}H NMR (600 MHz, $CDCl_3$)** δ 7.38 (dd, *J* = 6.4, 3.0 Hz, 2H), 7.30 – 7.25 (m, 5H), 7.19 (t, *J* = 5.7 Hz, 3H), 2.89 (d, *J* = 18.0 Hz, 1H), 2.74-2.91 (m, 1H), 2.67 (d, *J* = 18.0 Hz, 1H), 2.57-2.52 (m, 1H), 2.08-2.07(m, 1H), 1.94-1.93 (m, 1H), 1.90 (s, 2H), 1.52 (s, 9H). **^{13}C NMR (151 MHz, $CDCl_3$)** δ 174.63, 141.64, 131.67, 128.52, 128.34, 128.25, 127.96, 126.04, 123.34, 85.13, 83.50, 81.55, 61.12, 41.59, 31.26, 30.72, 28.08. $[M+H]^+$ Calculated for $C_{23}H_{28}NO_2$ 350.2115 found 350.2115.

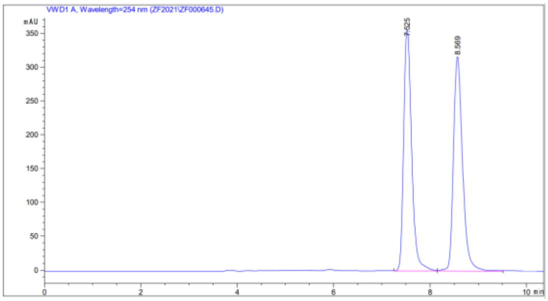

Peak #	RetTime [min]	Type	Width [min]	Area [mAU*s]	Height [mAU]	Area %
1	7.525	VV	0.1786	4120.13672	356.34445	49.7218
2	8.569	VV	0.2030	4166.24072	316.73987	50.2782

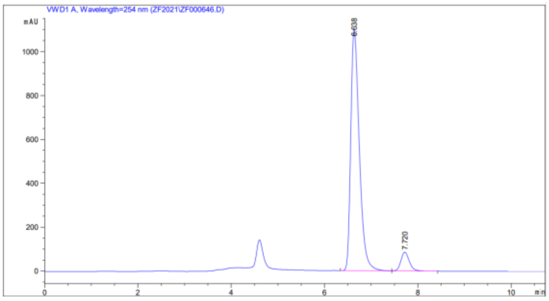

Peak #	RetTime [min]	Type	Width [min]	Area [mAU*s]	Height [mAU]	Area %
1	6.638	BV	0.1949	1.39381e4	1097.17896	92.8735
2	7.720	VB	0.1922	1069.51208	85.69890	7.1265

tert-Butyl (S)-2-amino-2-ethyl-5-phenylpent-4-ynoate (5j):

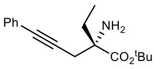

Yellow oil (29.4 mg, 54 %); R_f = 0.30 (petroleum ether/ ethyl acetate = 2:1); the enantiomeric excess was determined to be 94% by HPLC analysis on Daicel Chirapak OD-H column (hexane/isopropanol = 95/5, flow rate 1.0 mL/min, T = 30 °C), UV 254 nm, t_R (major) 14.928 min, t_R (minor) 17.799 min; $[\alpha]_D^{20}$ = -32.63 (c = 0.61, CH_2Cl_2); **^{1}H NMR (600 MHz, $CDCl_3$)** δ 7.38-7.37 (m, 2H), 7.30 – 7.25 (m, 3H), 2.86 (d, *J* = 18.0 Hz, 1H), 2.60 (d, *J* = 18.0 Hz, 1H), 1.84 (s, 2H), 1.84-1.82 (m, 1H), 1.70 – 1.59 (m, 1H), 1.49 (s, 9H), 0.92 (t, *J* = 7.5 Hz, 3H). **^{13}C NMR (151 MHz, $CDCl_3$)** δ 174.84, 131.63, 128.20, 127.86, 123.42, 85.46, 83.18, 81.26, 61.41, 32.45, 30.82, 28.00, 8.39. $[M+H]^+$ Calculated for $C_{17}H_{24}NO_2$ 274.1802 found 274.1802.

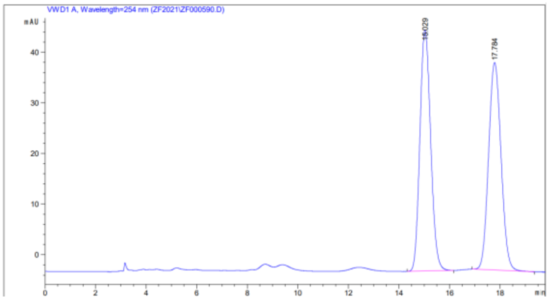

Peak #	RetTime [min]	Type	Width [min]	Area [mAU*s]	Height [mAU]	Area %
1	15.029	BB	0.4625	1405.61548	47.60720	50.1141
2	17.784	BB	0.5365	1399.21411	41.01195	49.8859

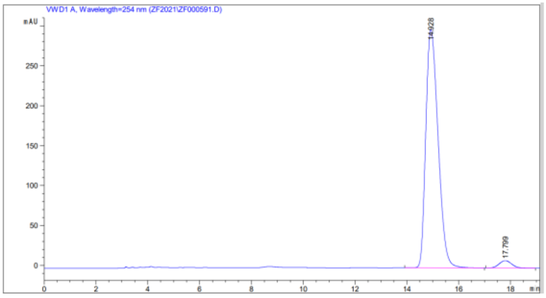

Peak #	RetTime [min]	Type	Width [min]	Area [mAU*s]	Height [mAU]	Area %
1	14.928	BB	0.5201	9793.11035	298.25101	96.9325
2	17.799	BB	0.5224	309.91028	9.27602	3.0675

tert-Butyl (S)-2-amino-2-isobutyl-5-phenylpent-4-ynoate (5k):

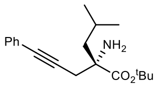

Yellow oil (40.6 mg, 67 %); R_f = 0.34 (petroleum ether/ ethyl acetate = 4:1); the enantiomeric excess was determined to be 90% by HPLC analysis on Daicel Chirapak AD-H column (hexane/isopropanol = 95/5, flow rate 1.0 mL/min, T = 30 °C), UV 254 nm, t_R (major) 5.953 min, t_R (minor) 7.120 min; $[\alpha]_D^{20}$ = -49.27 (c = 0.83, CH_2Cl_2); **^{1}H NMR (600 MHz, $CDCl_3$)** δ 7.38-7.36 (m, 2H), 7.27-7.26 (m, 3H), 2.84 (d, *J* = 16.4 Hz, 1H), 2.57 (d, *J* = 16.4 Hz, 1H), 1.88 (s, 2H), 1.82 – 1.72 (m, 2H), 1.58-1.56 (m, 1H), 1.49 (s, 9H), 0.97 (d, *J* = 6.4 Hz, 3H), 0.92 (d, *J* = 6.4 Hz, 3H). **^{13}C NMR (151 MHz, $CDCl_3$)** δ 175.38, 131.62, 128.20, 127.87, 123.40, 85.29, 83.42, 81.35, 60.87, 47.89, 32.60, 27.97, 24.58, 24.55, 23.27.$[M+H]^+$ Calculated for $C_{19}H_{28}NO_2$ 302.2115 found 302.2115.

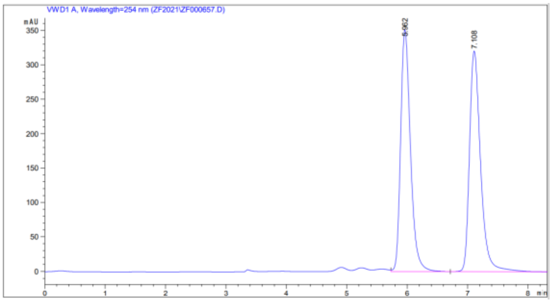
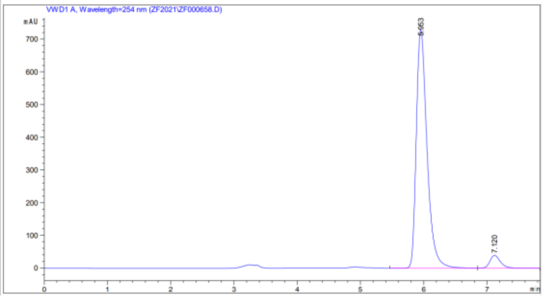

Peak #	RetTime [min]	Type	Width [min]	Area [mAU*s]	Height [mAU]	Area %
1	5.962	VB	0.1744	3976.22998	351.05264	49.3715
2	7.108	BBA	0.1936	4077.47290	320.46634	50.6285

Peak #	RetTime [min]	Type	Width [min]	Area [mAU*s]	Height [mAU]	Area %
1	5.953	BV	0.1799	8609.15137	729.67761	94.9410
2	7.120	VBA	0.1768	458.74515	39.34615	5.0590

tert-Butyl (S)-2-amino-2-(3-phenylprop-2-yn-1-yl)pent-4-enoate (5l):

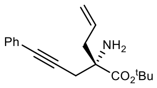

Yellow soild (44.0 mg, 77 %); m.p. = 64-66 °C; R_f = 0.35 (petroleum ether/ ethyl acetate = 3:1); the enantiomeric excess was determined to be 92% by HPLC analysis on Daicel Chirapak AD-H column (hexane/isopropanol = 95/5, flow rate 1.0 mL/min, T = 30 °C), UV 254 nm, t_R (major) 6.417 min, t_R (minor) 7.742 min; $[\alpha]_D^{20}$ = -27.84 (c = 0.56, CH_2Cl_2); **^{1}H NMR (600 MHz, $CDCl_3$)** δ 7.37 (dd, *J* = 6.2, 2.8 Hz, 2H), 7.28 – 7.25 (m,3H), 5.79-5.72 (m, 1H), 5.17 (t, *J* = 12.9 Hz, 2H), 2.86 (d, *J* = 18.0 Hz, 1H), 2.62 (d, *J* = 18.0 Hz, 1H), 2.57 (dd, *J* = 13.5, 6.7 Hz, 1H), 2.34 (dd, *J* = 13.4, 8.1 Hz, 1H), 1.86 (s, 2H), 1.49 (s, 9H). **^{13}C NMR (151 MHz, $CDCl_3$)** δ 174.34, 132.46, 131.63, 128.20, 127.90, 123.38, 119.48, 85.15, 83.45, 81.49, 60.62, 43.68, 30.90, 28.02. $[M+H]^+$ Calculated for $C_{18}H_{24}NO_2$ 286.1802 found 286.1803.

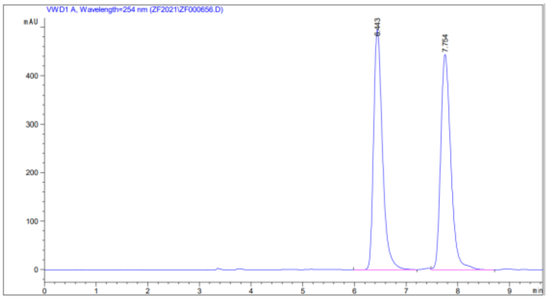
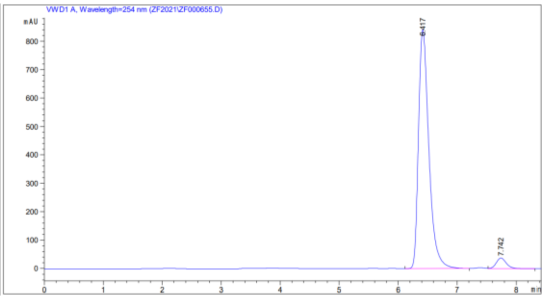

Peak #	RetTime [min]	Type	Width [min]	Area [mAU*s]	Height [mAU]	Area %
1	6.443	BV	0.1847	5918.99121	495.03534	49.5248
2	7.754	VB	0.2110	6032.57227	443.91034	50.4752

Peak #	RetTime [min]	Type	Width [min]	Area [mAU*s]	Height [mAU]	Area %
1	6.417	BB	0.1861	1.02581e4	840.91260	95.8763
2	7.742	VB	0.1818	441.20828	37.29353	4.1237

tert-Butyl (R)-2-amino-2-(2-(methylthio)ethyl)-5-phenylpent-4-ynoate (5m):

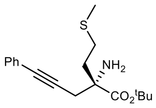

Yellow oil (53.8 mg, 84 %); R_f = 0.24 (petroleum ether/ ethyl acetate = 2.5:1); the enantiomeric excess was determined to be 94% by HPLC analysis on Daicel Chirapak AD-H column (hexane/isopropanol = 95/5, flow rate 0.8 mL/min, T = 30 °C), UV 254 nm, t_R (major) 11.793 min, t_R (minor) 14.045 min; $[\alpha]_D^{20}$ = -29.94 (c =1.1, CH_2Cl_2); **^{1}H NMR (600 MHz, CDCl$_3$)** δ 7.37 (dd, J = 6.6, 3.0 Hz, 2H), 7.27 (dd, J = 4.9, 1.6 Hz, 3H), 2.85 (d, J = 18.0 Hz, 1H), 2.65 (d, J = 18.0 Hz, 1H), 2.60 -2.57 (m, 1H), 2.47-2.42 (m, 1H), 2.12 (s, 3H), 2.11 – 2.05 (m, 1H), 1.92-1.85 (m, 1H), 1.85 (s, 2H), 1.49 (s, 9H). **^{13}C NMR (151 MHz, CDCl$_3$)** δ 174.08, 131.62, 128.22, 127.97, 123.24, 84.77, 83.68, 81.72, 60.89, 38.95, 31.29, 28.91, 27.98, 15.54. $[M+H]^+$ Calculated for $C_{18}H_{26}NO_2S$ 320.1679 found 320.1678.

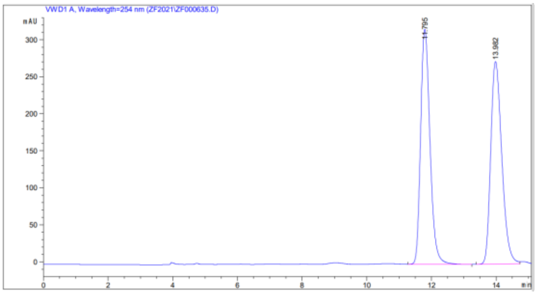
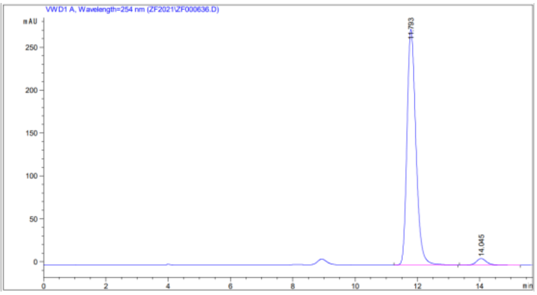

Peak #	RetTime [min]	Type	Width [min]	Area [mAU*s]	Height [mAU]	Area %
1	11.795	BB	0.3114	6373.41602	317.54141	49.4653
2	13.982	BV	0.3709	6511.21191	273.65045	50.5347

Peak #	RetTime [min]	Type	Width [min]	Area [mAU*s]	Height [mAU]	Area %
1	11.793	BB	0.3068	5439.97021	274.77191	96.9045
2	14.045	BB	0.3479	173.77415	7.61503	3.0955

di-tert-Butyl (S)-2-amino-2-(3-phenylprop-2-yn-1-yl)pentanedioate (5n):

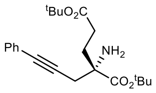

Yellow oil (42.1 mg, 56 %); R_f = 0.39 (petroleum ether/ ethyl acetate = 2:1); the enantiomeric excess was determined to be 82% by HPLC analysis on Daicel Chirapak AD-H column (hexane/isopropanol = 95/5, flow rate 1.0 mL/min, T = 30 °C), UV 254 nm, t_R (major) 8.325 min, t_R (minor) 8.890 min; $[\alpha]_D^{20}$ = -13.67 (c =0.39, CH_2Cl_2); **^{1}H NMR (600 MHz,**

$CDCl_3$) δ 7.38 – 7.35 (m, 2H), 7.28 – 7.26 (m, 3H), 2.85 (d, *J* = 18.0 Hz, 1H), 2.61 (d, *J* = 18.0 Hz, 1H), 2.40-2.35 (m, 1H), 2.23-2.20 (m, 1H), 2.09 – 2.02 (m, 1H), 1.97 – 1.90 (m, 1H), 1.78 (s, 2H), 1.49 (s, 9H), 1.44 (s,9H).**^{13}C NMR (151 MHz, $CDCl_3$)** δ 174.42, 172.59, 131.75, 128.33, 128.06, 123.40, 85.11, 83.64, 81.78, 80.54, 60.63, 34.29, 31.16, 30.60, 28.21, 28.09. $[M+H]^+$ Calculated for $C_{22}H_{32}NO_4$ 374.2326 found 374.2327.

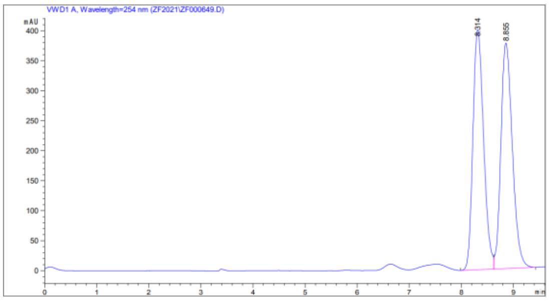

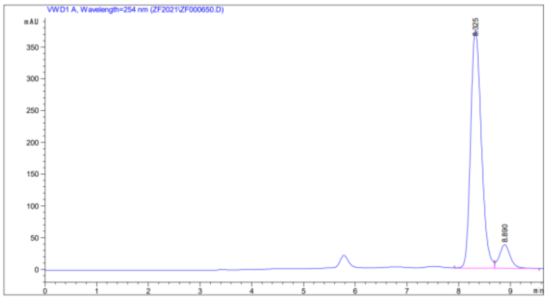

Peak #	RetTime [min]	Type	Width [min]	Area [mAU*s]	Height [mAU]	Area %
1	8.314	BV	0.2234	5705.13232	399.70541	49.8071
2	8.855	VB	0.2382	5749.31641	375.83401	50.1929

Peak #	RetTime [min]	Type	Width [min]	Area [mAU*s]	Height [mAU]	Area %
1	8.325	VV	0.2241	5376.86133	375.03836	90.9503
2	8.890	VBA	0.2190	535.00519	37.16198	9.0497

1.4 Determination of the absolute configuration of 4a

The absolute configuration of compound **4a** was established by comparing its optical rotation value with the literature data:

(*S*)-product (**4a**) in this work	(*S*)-product in literature[4]
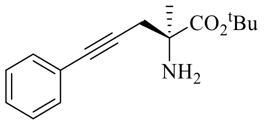 *tert*-butyl (*S*)-2-amino-2-methyl-5-phenylpent-4-ynoate	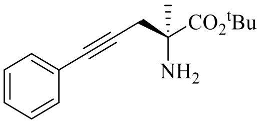 *tert*-butyl (*S*)-2-amino-2-methyl-5-phenylpent-4-ynoate
$[\alpha]_D^{20}$ = -20.45 (c = 0.92, CH_2Cl_2)	$[\alpha]_D^{20}$ = -19.70 (c = 0.73, CH_2Cl_2)

1.5 The stereodivergent synthesis of NP25302

1.5.1 The synthesis of compounds 6

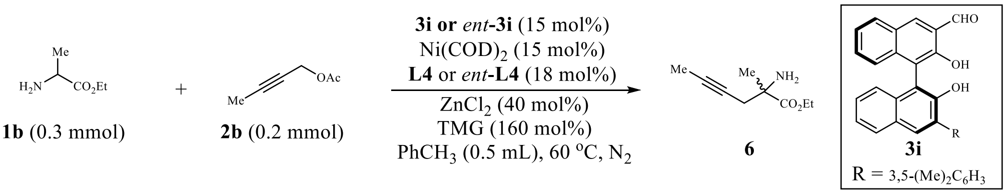

In a nitrogen-filled glove box, an oven-dried 10 mL screw-cap reaction tube equipped with a stir bar was charged with $Ni(COD)_2$ (8.2 mg, 0.03 mmol), **L4** (23.0 mg, 0.036 mmol) and stirred in toluene (0.5 mL) at r.t. for about 5 min. Then, ethyl amino acid ester **1b** (0.3 mmol, 35.4 mg), propargylic acetate ester **2b** (0.2 mmol, 22.4 mg), chiral aldehyde **3i** (12.5 mg, 0.03 mmol), $ZnCl_2$

(10.9 mg, 0.08 mmol) and TMG (36.8 mg, 0.32 mmol) were added. The mixture was continuously stirred at indicated reaction temperature under nitrogen atmosphere. After the reaction completed, the solvent was removed by rotary evaporation, and the residue was purified by flash chromatography separation on silica gel column (eluent: petroleum ether/ ethyl acetate/ triethylamine = 200/100/2) to afford (*S*)-**6** (18.9 mg, 56%). (*R*)-**6** was prepared according to this procedure under the promotion of chiral aldehyde *ent*-**3i** and ligand *ent*-**L4**.

Ethyl (S)-2-amino-2-methylhex-4-ynoate (6):

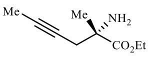

Colorless oil (18.9 mg, 56 %); R_f = 0.33 (petroleum ether/ ethyl acetate = 2:1); the enantiomeric excess was determined to be 90% by HPLC analysis on Daicel Chirapak ID-H column (hexane/isopropanol = 97/3, flow rate 0.7 mL/min, T = 30 °C), UV 254 nm, t_R(major) 20.640 min, t_R(minor) 19.942 min; $[\alpha]_D^{20}$ = -6.91 (c =1.2, CH_2Cl_2); **^{1}H NMR (600 MHz, $CDCl_3$)** δ 4.21-4.18 (m, 2H), 2.61 (d, *J* = 16.2 Hz, 1H), 2.39 (d, *J* = 16.2 Hz, 1H), 1.90 (s, 2H), 1.78 (s, 3H), 1.35 (s, 3H), 1.28 (t, *J* = 6.9 Hz, 3H). **^{13}C NMR (151 MHz, $CDCl_3$)** δ 176.33, 78.69, 74.30, 61.16, 57.53, 31.37, 25.77, 14.15, 3.40. $[M+H]^+$ Calculated for $C_9H_{16}NO_2$ 170.1176 found 170.1175.

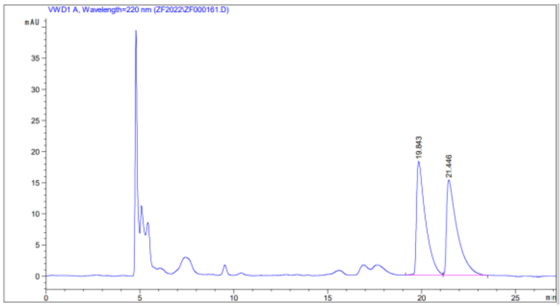

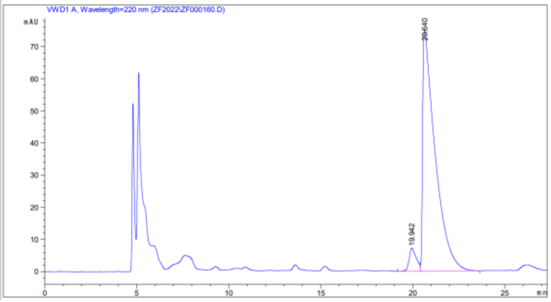

Peak #	RetTime [min]	Type	Width [min]	Area [mAU*s]	Height [mAU]	Area %
1	19.843	BV	0.4731	605.45148	18.24751	50.3459
2	21.446	VB	0.5461	597.13184	15.28650	49.6541

Peak #	RetTime [min]	Type	Width [min]	Area [mAU*s]	Height [mAU]	Area %
1	19.942	BV	0.3864	193.41840	7.16533	5.2092
2	20.640	VB	0.6497	3519.61597	74.97895	94.7908

Ethyl (R)-2-amino-2-methylhex-4-ynoate ((***R)*-6**):

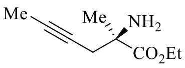

Colorless oil (16.8 mg, 50 %); R_f = 0.33 (petroleum ether/ ethyl acetate = 2:1); the enantiomeric excess was determined to be 89% by HPLC analysis on Daicel Chirapak ID-H column (hexane/isopropanol = 95/5, flow rate 0.7 mL/min, T = 30 °C), UV 254 nm, t_R(major) 15.729 min, t_R(minor) 17.278 min; $[\alpha]_D^{20}$ = +7.92 (c =1.4, CH_2Cl_2).

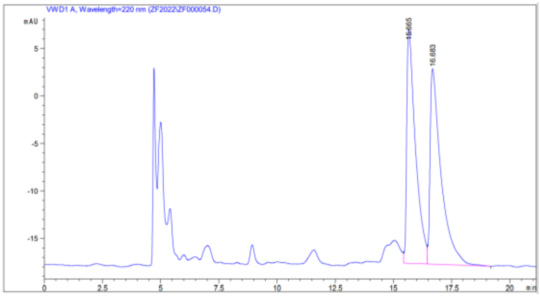

Peak #	RetTime [min]	Type	Width [min]	Area [mAU*s]	Height [mAU]	Area %
1	15.665	VV	0.3798	650.53723	24.61258	49.0435
2	16.683	VB	0.4646	675.91138	20.58851	50.9565

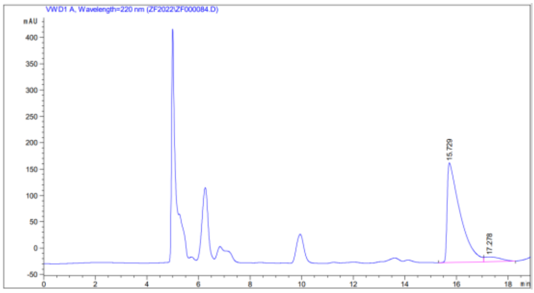

Peak #	RetTime [min]	Type	Width [min]	Area [mAU*s]	Height [mAU]	Area %
1	15.729	BV	0.4861	6611.47949	189.33780	94.5575
2	17.278	VB	0.6634	380.53754	8.85115	5.4425

1.5.2 The synthesis of compounds 7

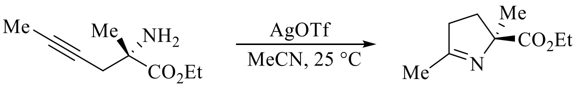

(*S*)-6 **(*S*)-7**

(*S*)-**6** (246.9 mg, 1.4 mmol) was dissolved in dry acetonitrile (3 mL), AgOTf (72.0 mg, 0.01 mmol) was added, and the mixture was stirred at 25 °C for 24 h. The solvent was removed in vacuo. The residue was purified by column chromatography to give (*S*)-**7** (eluent: petroleum ether/ ethyl acetate =100/200). $[\alpha]_D^{22}$ = +62.44 (c = 1.2, CH_2Cl_2); **^{1}H NMR (600 MHz, $CDCl_3$)** δ 4.20-4.16 (m, 2H), 2.65-2.60 (m, 2H), 2.35-2.34 (m, 1H), 2.07 (s, 3H), 1.79-1.77 (m, 1H), 1.45 (s, 3H), 1.27 (t, *J* = 7.1 Hz, 3H). **^{13}C NMR (151 MHz, $CDCl_3$)** δ 175.93, 174.75, 79.30, 60.86, 39.21, 34.07, 24.92, 19.60, 14.05. $[M+H]^+$ Calculated for $C_9H_{16}NO_2$ 170.1176 found 170.1175.

(*R*)-**7** was prepared according this procedure from (*R*)-**6**. $[\alpha]_D^{22}$ = -44.11 (c = 1.4, CH_2Cl_2).

1.5.3 The synthesis of compounds 8

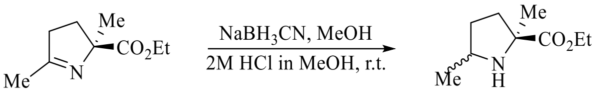

(*S*)-7 **(*S, R*)-8** **(*S, S*)-8**

Compound (*S*)-**7** (142.0 mg, 0.84 mmol) was dissolved in MeOH (2 mL), followed by $NaBH_3CN$ (158.8 mg, 2.52 mmol) was added, and additional 2 M HCl was added periodically to maintain the acidic pH of the reaction mixture. The progress of the reaction was monitored by TLC and added saturated $NaHCO_3$ after 60 min to maintain the alkaline pH of the reaction mixture. The mixture was extracted with CH_2Cl_2 (5 mL × 3). The combined organic extracts were dried over Na_2SO_4, concentrated and purified by flash column chromatography separation (eluent: petroleum ether/ ethyl acetate =4/1) to afford the diastereoisomers (*S, R*)-**8** and (*S, S*)-**8**.

The other two diastereoisomers (*R, S*)-**8** and (*R, R*)-**8** were prepared according to this procedure

with the utilization of (*R*)-**7** as starting material.

1.5.4 The synthesis of compounds 9

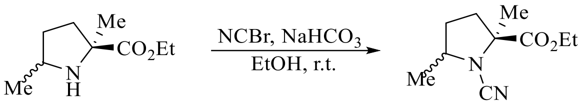

(*S*, *S*)-8 **(*S*, *S*)-9**
(*S*, *R*)-8 **(*S*, *R*)-9**

General procedure: $NaHCO_3$ (138.6 mg, 1.7 mmol) and NCBr (70.0 mg, 0.66 mmol) were added to a solution of **8** (95.4 mg, 0.55 mmol) in EtOH (2 mL), and the resulting mixture was stirred for 2 h at 25 °C. H_2O (3 mL) was added, and the solution was stirred for 15 min and extracted with CH_2Cl_2 (3 mL × 3). The combined organic extracts were dried over $MgSO_4$, concentrated and purified by flash column chromatography separation (eluent: petroleum ether/ ethyl acetate =4/1) to afford products **9**. The (*R*, *R*)- and (*R*, *S*)-**9** were prepared analogously from (*R*, *R*)- and (*R*, *S*)**-8**.

Ethyl (2*S*, 5*S*)-1-cyano-2,5-dimethylpyrrolidine-2-carboxylate ((*S*, *S*)-9):

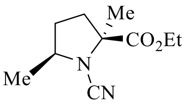

Colorless oil (51.0 mg, 35%); R_f = 0.24 (petroleum ether/ ethyl acetate = 2:1); the enantiomeric excess was determined to be 86% by HPLC analysis on Daicel Chirapak ID-H column (hexane/isopropanol = 80/20, flow rate 0.8 mL/min, T = 30 °C), UV 220 nm, t_R(major) 11.680 min, t_R(minor) 10.943; $[\alpha]_D^{22}$ = -30.58 (c =1.1, MeOH); **^{1}H NMR (600 MHz, CDCl$_3$)** δ 4.27 – 4.15 (m, 2H), 3.83-3.82 (m, 1H), 2.43-2.41 (m, 1H), 2.07-2.04 (m, 1H), 1.79-1.75 (m, 1H), 1.60 (s, 3H), 1.55-1.53 (m, 1H), 1.38 (d, *J* = 6.2 Hz, 3H), 1.33 – 1.28 (m, 3H). **^{13}C NMR (151 MHz, CDCl$_3$)** δ 172.96, 114.46, 68.16, 61.90, 58.50, 37.27, 32.11, 23.07, 19.58, 14.14. $[M+H]^+$ Calculated for $C_{10}H_{17}N_2O_2$ 197.1285 found 197.1284.

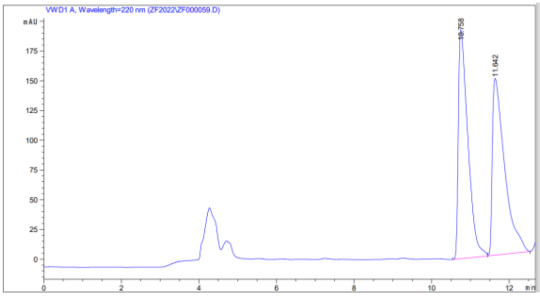

Peak #	RetTime [min]	Type	Width [min]	Area [mAU*s]	Height [mAU]	Area %
1	10.758	BV	0.2573	3275.35767	192.00891	50.0364
2	11.642	VB	0.3220	3270.59204	147.98445	49.9636

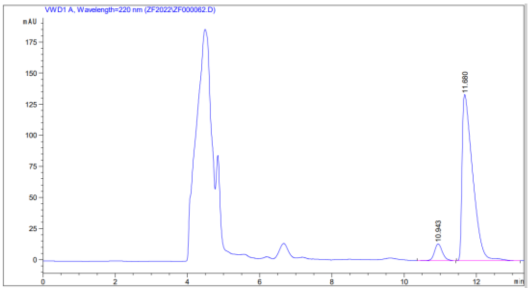

Peak #	RetTime [min]	Type	Width [min]	Area [mAU*s]	Height [mAU]	Area %
1	10.943	BB	0.2374	206.35875	13.44393	7.0101
2	11.680	BBA	0.3040	2737.36255	133.12921	92.9899

Ethyl (2*S*, 5*R*)-1-cyano-2,5-dimethylpyrrolidine-2-carboxylate ((*S, R*)-9):

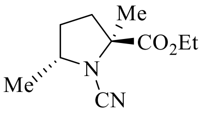

Colorless oil (46.2 mg, 40 %); R_f = 0.53 (petroleum ether/ ethyl acetate = 2:1); the enantiomeric excess was determined to be 88% by HPLC analysis on Daicel Chirapak ID-H column (hexane/isopropanol = 80/20, flow rate 1.0 mL/min, T = 30 °C), UV 220 nm, t_R(major) 11.680 min, t_R(minor) 10.943; $[\alpha]_D^{22}$ = -107.26 (c =0.96, MeOH); **^{1}H NMR (600 MHz, $CDCl_3$)** δ 4.23 (q, *J* = 7.1 Hz, 2H), 3.85 (dd, *J* = 12.8, 6.4 Hz, 1H), 2.27 (ddd, *J* = 13.0, 7.3, 5.7 Hz, 1H), 2.06-2.02 (m, 1H), 1.89 (dd, *J* = 7.9, 5.0 Hz, 1H), 1.60 (s, 3H), 1.59 – 1.55 (m, 1H), 1.32 (dd, *J* = 15.1, 6.8 Hz, 6H). **^{13}C NMR (151 MHz, $CDCl_3$)** δ 172.82, 114.38, 68.59, 61.92, 58.15, 35.83, 31.00, 23.17, 20.00, 14.17. $[M+H]^+$ Calculated for $C_{10}H_{17}N_2O_2$ 197.1285 found 197.1284.

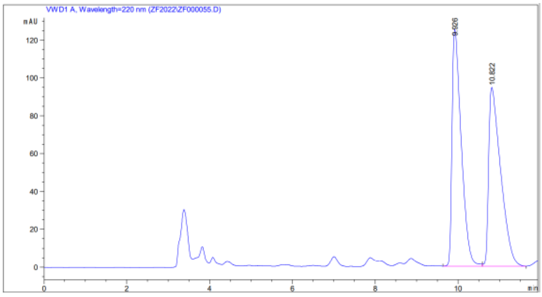

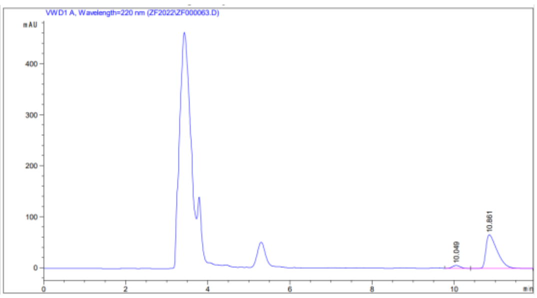

Peak #	RetTime [min]	Type	Width [min]	Area [mAU*s]	Height [mAU]	Area %
1	9.926	BV	0.2329	1936.02258	125.15704	50.4454
2	10.822	VB	0.2987	1901.83691	94.59985	49.5546

Peak #	RetTime [min]	Type	Width [min]	Area [mAU*s]	Height [mAU]	Area %
1	10.049	VV	0.2140	78.88947	5.69845	5.9159
2	10.861	VBA	0.2858	1254.61646	66.03918	94.0841

Ethyl (2*R*,5*R*)-1-cyano-2,5-dimethylpyrrolidine-2-carboxylate ((*R, R*)-9):

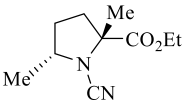

Colorless oil (35.6 mg, 39%); R_f = 0.24 (petroleum ether/ ethyl acetate = 2:1); the enantiomeric excess was determined to be 88% by HPLC analysis on Daicel Chirapak ID-H column (hexane/isopropanol = 80/20, flow rate 0.8 mL/min, T = 30 °C), UV 220 nm, t_R(major) 10.947 min, t_R(minor) 11.957; $[\alpha]_D^{22}$ = +31.65 (c =0.89, MeOH).

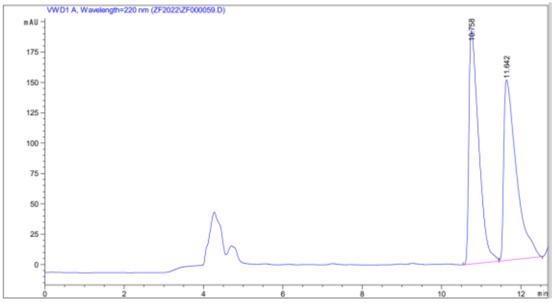

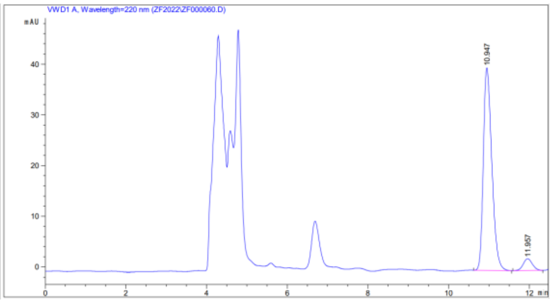

Peak #	RetTime [min]	Type	Width [min]	Area [mAU*s]	Height [mAU]	Area %
1	10.758	BV	0.2573	3275.35767	192.00891	50.0364
2	11.642	VB	0.3220	3270.59204	147.98445	49.9636

Peak #	RetTime [min]	Type	Width [min]	Area [mAU*s]	Height [mAU]	Area %
1	10.947	BB	0.2259	583.60242	39.93624	94.1017
2	11.957	BB	0.2402	36.58037	2.32756	5.8983

Ethyl (2*R*, 5*S*)-1-cyano-2,5-dimethylpyrrolidine-2-carboxylate ((*R, S*)-9):

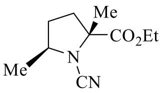

Colorless oil (26.5 mg, 32%); R_f = 0.24 (petroleum ether/ ethyl acetate = 2:1); the enantiomeric excess was determined to be 87% by HPLC analysis on Daicel Chirapak ID-H column (hexane/isopropanol = 80/20, flow rate 1.0 mL/min, T = 30 °C), UV 220 nm, t_R(major) 9.949 min, t_R(minor) 10.987; $[\alpha]_D^{22}$ = +85.77 (c =0.66, MeOH).

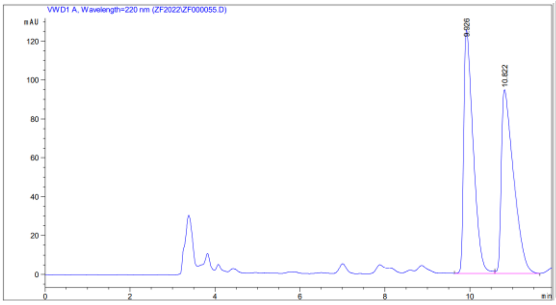

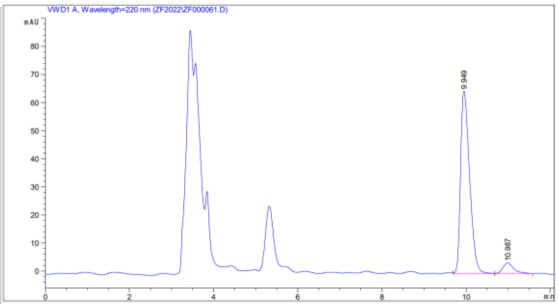

Peak #	RetTime [min]	Type	Width [min]	Area [mAU*s]	Height [mAU]	Area %
1	9.926	BV	0.2329	1936.02258	125.15704	50.4454
2	10.822	VB	0.2987	1901.83691	94.59985	49.5546

Peak #	RetTime [min]	Type	Width [min]	Area [mAU*s]	Height [mAU]	Area %
1	9.949	VV	0.2307	968.94452	65.04191	93.2867
2	10.987	VB	0.2821	69.72893	3.75779	6.7133

1.5.5 The synthesis of compounds 10

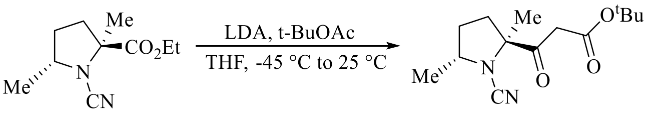

(*S, R*)-9 **(*S, R*)-10**

tert-Butyl acetate (56.8 mg, 0.49 mmol) was added dropwise over a period of 3 min to a freshly prepared solution of LDA (0.20 ml, 0.43 mmol) at -45 °C under N_2. The solution was stirred for 15 min and treated with cyanamide **(*S, R*)-9** (37.6 mg, 0.19 mmol) in 0.5 mL of dry THF. The mixture was stirred for 1 h at -45 °C and 2 h at 25 °C, quenched with H_2O (2 mL), and extracted with CH_2Cl_2 (3 mL × 3). The combined organic extracts were dried over $MgSO_4$ and concentrated to crude (*S, R*)-**10**, which was used for the next step without further purification.

Compound (*R, S*)-**10** was prepared from (*R, S*)-**9** according to the above procedure.

1.5.6 The synthesis of compounds 11

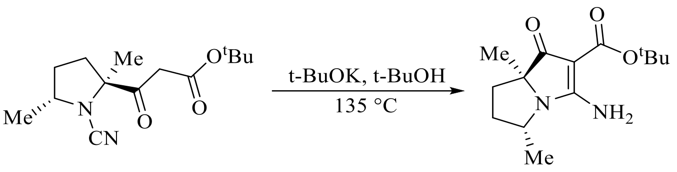

(*S, R*)-10 **(*S, R*)-11**

Compound (*S, R*)-**10** (50.5 mg. 0.19 mmol) was stirred in 2 mL of *t*-BuOH, followed by *t*-BuOH (32.0 mg, 0.285 mmol) was added under N_2 in a sealed tube submerged in a 135 °C oil bath. After

15 h, the mixture was cooled, diluted with 2 mL of H_2O, and extracted with CH_2Cl_2 (3 mL × 3). The combined organic extracts were dried over $MgSO_4$ and concentrated. Flash chromatography separation on silica gel column (95:5 CH_2Cl_2/MeOH as eluent) gave (*S, R*)-**11** (21.8 mg, 42% over 2 steps). White solid; m.p.= 247-249 °C; R_f= 0.23 (petroleum ether/ ethyl acetate = 2:1); $[\alpha]_D^{22}$ = -17.15 (c =0.83, MeOH); **^{1}H NMR (600 MHz, $CDCl_3$)** δ 3.63 (dd, *J* = 14.3, 7.1 Hz, 1H), 2.38 – 2.32 (m, 1H), 1.95 – 1.87 (m, 1H), 1.82 (dd, *J* = 12.5, 6.5 Hz, 1H), 1.70 – 1.64 (m, 1H), 1.62 (s, 2H), 1.55 (s, 9H), 1.40 (d, *J* = 6.5 Hz, 3H), 1.35 (s, 3H). **^{13}C NMR (151 MHz, $CDCl_3$)** δ 194.53, 173.28, 165.98, 88.41, 80.01, 74.79, 55.41, 35.55, 32.25, 28.59, 24.07, 22.63. $[M+H]^+$ Calculated for $C_{14}H_{23}N_2O_3$ 267.1703 found 267.1702.

Compound (*R, S*)-**11** was prepared according to the above procedure from (*R, S*)-**10**. $[\alpha]_D^{22}$ = +29.15 (c = 0.11, MeOH).

1.5.7 The synthesis of compounds (*S, R*)- and (*R, S*)-NP25302

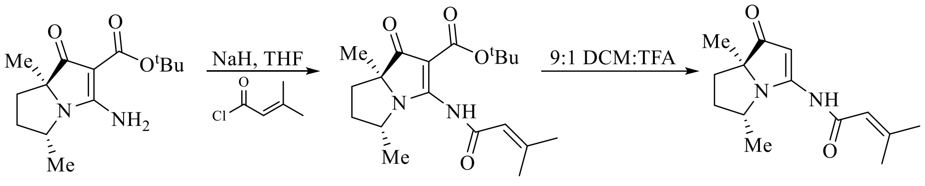

(*S, R*)-11 (*S, R*)-12 (*S, R*)-NP25302

Compound (*S, R*)-**11** (21.8 mg, 0.08 mmol) and NaH (60% dispersion in mineral oil, 40 mg, 0.64 mmol) were stirred in 2 mL of dry THF under N_2 at 25 °C for 10 min. A solution of 3,3-dimethylacryloyl chloride (14.28 mg, 0.12 mmol) was added, and the resulting mixture was stirred for 2 h, quenched with H_2O, and extracted with CH_2Cl_2 (3 mL × 3). The combined organic extracts were dried over $MgSO_4$ and concentrated to give crude (*S, R*)-**12**. This crude (*S, R*)-**12** was dissolved in 3 mL of a 9:1 CH_2Cl_2/TFA solution and stirred for 15 h at 25 °C. The mixture was neutralized with saturated sodium bicarbonate solution, saturated with NaCl, and extracted with EtOAc (2 mL × 3). The combined organic extracts were dried over $MgSO_4$ and concentrated to give crude product. Pure (*S, R*)-**NP25302** was obtained by flash chromatography separation on silica gel column (eluent: petroleum ether/ ethyl acetate/ triethylamine =100/200/2). (*R, S*)-**NP25302** was prepared from (*R, S*)-**11** (11.5 mg, 0.04 mmol) according to this procedure.

N-((5R,7aS)-5,7a-dimethyl-1-oxo-5,6,7,7a-tetrahydro-1H-pyrrolizin-3-yl)-3-methylbut-2-enamide ((*S*, *R*)-NP25302):

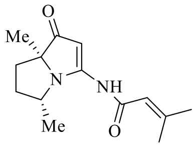

White solid (13.4 mg, 71 %); m. p. = 217-219 °C; R_f = 0.19 (petroleum ether/ ethyl acetate = 1:2); the enantiomeric excess was determined to be 89% by HPLC analysis on Daicel Chirapak ID-H column (hexane/isopropanol = 70/30, flow rate 1.0 mL/min, T = 30 °C), UV 254 nm, t_R(major) 12.533 min, t_R(minor) 11.484 min; $[\alpha]_D^{22}$ = + 62.27 (c = 0.44, CH_3OH); **^{1}H NMR (600 MHz, $CDCl_3$)** δ 8.19 (s, 1H), 5.84 (s, 1H), 5.79 (s, 1H), 3.69 – 3.58 (m, 1H), 2.22 (s, 3H), 2.18 – 2.12 (m, 1H), 1.92 (s, 3H), 1.85 – 1.79 (m, 3H), 1.38 – 1.33 (m, 6H). **^{13}C NMR (151 MHz, $CDCl_3$)** δ 204.99, 169.35, 163.96, 158.45, 117.35, 91.78, 73.54, 56.31, 34.50, 31.55, 27.67, 23.66, 23.45, 20.50. $[M+H]^+$ Calculated for $C_{14}H_{21}N_2O$ 249.1598 found 249.1597.

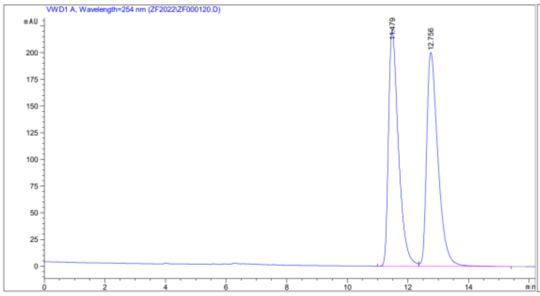

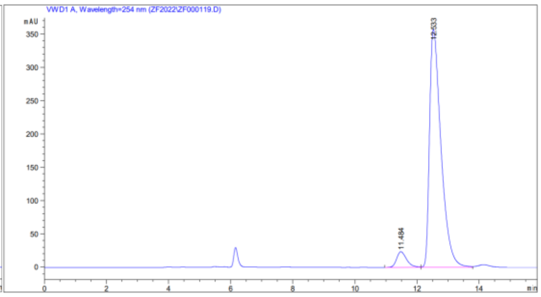

Peak #	RetTime [min]	Type	Width [min]	Area [mAU*s]	Height [mAU]	Area %
1	11.479	BV	0.3467	5060.71094	221.56030	49.4982
2	12.756	VB	0.3926	5163.31250	200.29945	50.5018

Peak #	RetTime [min]	Type	Width [min]	Area [mAU*s]	Height [mAU]	Area %
1	11.484	BV	0.3408	525.14404	23.50753	5.4134
2	12.533	VV	0.3888	9175.59375	356.99210	94.5866

N-((5S,7aR)-5,7a-dimethyl-1-oxo-5,6,7,7a-tetrahydro-1H-pyrrolizin-3-yl)-3-methylbut-2-enamide ((*R*, *S*)-NP25302):

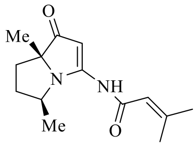

White solid (6.8 mg, 68 %); m. p. = 220-224 °C; R_f = 0.19 (petroleum ether/ ethyl acetate = 1:2); the enantiomeric excess was determined to be 91% by HPLC analysis on Daicel Chirapak ID-H column (hexane/isopropanol = 70/30, flow rate 1.0 mL/min, T = 30 °C), UV 254 nm, t_R(major) 11.457 min, t_R(minor) 12.933 min; $[\alpha]_D^{22}$ = -77.72 (c = 0.19, CH_3OH).

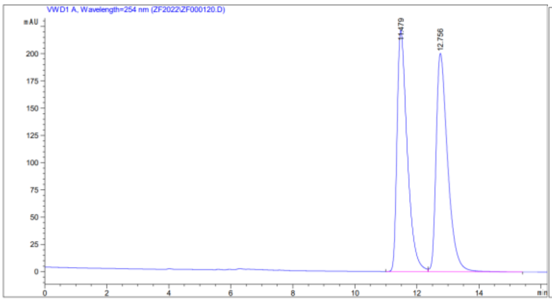

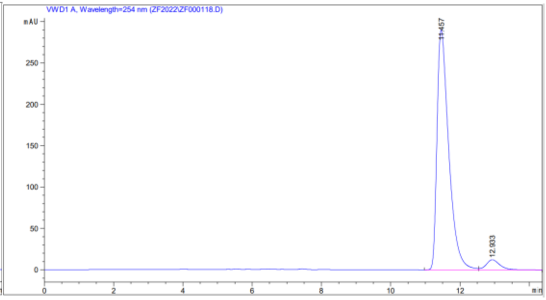

Peak #	RetTime [min]	Type	Width [min]	Area [mAU*s]	Height [mAU]	Area %	Peak #	RetTime [min]	Type	Width [min]	Area [mAU*s]	Height [mAU]	Area %
1	11.479	BV	0.3467	5060.71094	221.56030	49.4982	1	11.457	BV	0.3565	6830.98682	289.97098	95.2985
2	12.756	VB	0.3926	5163.31250	200.29945	50.5018	2	12.933	VBA	0.4155	337.00510	12.25812	4.7015

1.6 Reaction mechanism investigation

1.6.1 Control experiments[a]

Supplementary Table 8: Control experiments with modified chiral aldehyde catalysts.

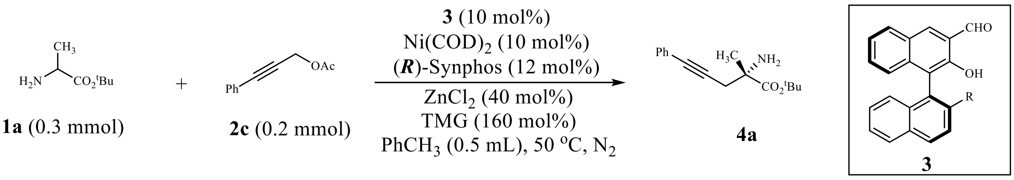

Entry	3, R	T (h)	Yield (%)[b]	ee (%)[c]
1	**3a**, OH	24	80	91
2	**3l**, OCH_3	24	trace	N.D.[d]
3	**3m**, H	24	trace	N.D.[d]

[a] Unless noted otherwise, reactions were performed with **1a** (0.30 mmol), **2c** (0.20 mmol), catalyst **3** (0.02 mmol), (***R***)-Synphos (0.024 mmol), $Ni(COD)_2$ (0.02 mmol), TMG (0.32 mmol), and $ZnCl_2$ (0.08 mmol) in toluene (0.5 mL) at 50 ºC. [b]Isolated yield. [c]Determined by chiral HPLC analysis. [d]N.D. = Not determined.

General procedure： In a nitrogen-filled glove box, an oven-dried 10 mL screw-cap reaction tube equipped with a stir bar was charged with $Ni(COD)_2$ (5.5 mg, 0.02 mmol), (***R***)-Synphos (15.3 mg, 0.024 mmol) and stirred in toluene (0.5 mL) at r.t. for about 5 min; Then, *tert*-butyl amino acid ester **1a** (0.3 mmol, 43.5 mg), propargylic acetate ester **2c** (0.2 mmol, 35.0 mg), chiral aldehyde **3** (0.02 mmol), $ZnCl_2$ (10.9 mg, 0.08 mmol) and TMG (36.8 mg, 0.32 mmol) were added. The mixture was continuously stirred at 50 °C under nitrogen atmosphere. After the reaction completed, the solvent was removed by rotary evaporation, and the residue was purified by flash chromatography separation on silica gel column (eluent: petroleum ether/ ethyl acetate/ triethylamine = 250/100/2).

1.6.2 Nonlinear effect investigation

All of these reactions were carried out under the optimal reaction conditions with the utilization of chiral aldehyde catalyst 3i and chiral ligand L1 with different ee values. The enantiomeric excess of chiral aldehydes catalysts 3i was determined by HPLC analysis on Daicel Chirapak IA-H column (hexane/isopropanol = 70/30, flow rate 1.0 mL/min, T = 30 ºC), UV 254 nm. The enantiomeric excess chiral ligand L1 was determined by HPLC analysis on Daicel Chirapak IF-H column (hexane/isopropanol = 95/5, flow rate 0.8 mL/min, T = 30 ºC), UV 254 nm.

Supplementary Table 9: Enantiopurity relationship between ligand **L1** and product **4a**

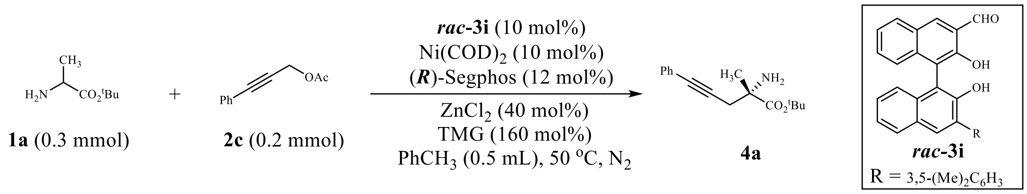

Entry	ee of L1 (%)[c]	T (h)	Yield (%)[b]	ee (%)[c]
1	0	24	40	0
2	21	24	43	20
3	40	24	46	33
4	59	24	41	55
5	78	24	49	64
6	100	24	52	81

[a] Unless noted otherwise, reactions were performed with **1** (0.30 mmol), **2** (0.20 mmol), catalyst ***rac.*-3i** (0.02 mmol), (***R***)-Segphos (0.024 mmol), $Ni(COD)_2$ (0.02 mmol), TMG (0.32 mmol), and $ZnCl_2$ (0.08 mmol) in toluene (0.5 mL) at 50 ºC. [b]Isolated yield. [c]Determined by chiral HPLC analysis.

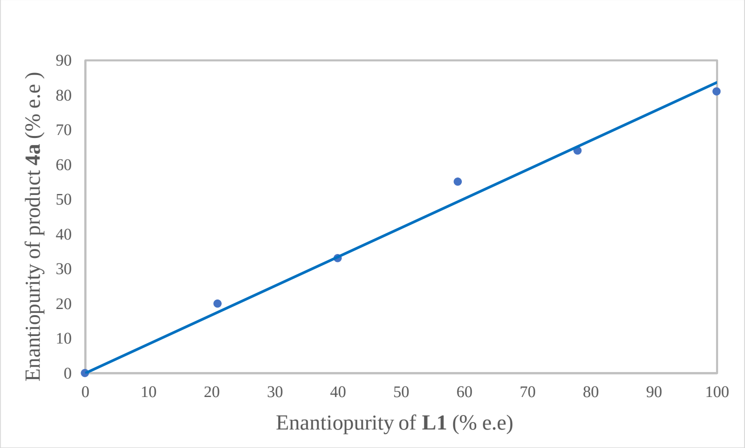

Supplementary Figure 1. The ee value relationship between **L1** and product **4a**.

Supplementary Table 10: Enantiopurity relationship between chiral aldehyde **3i** and product **4a**[a]

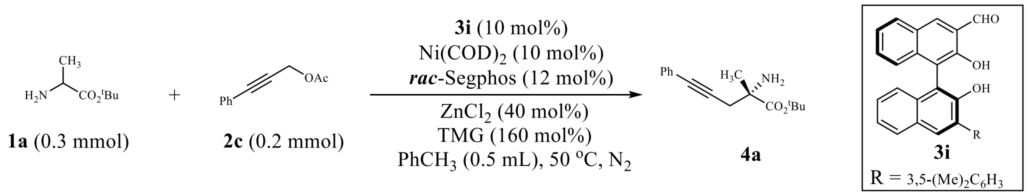

Entry	ee of 3i (%)[c]	T (h)	Yield (%)[b]	ee (%)[c]
1	0	24	40	0
2	18	24	42	4
3	39	24	41	8
4	60	24	45	20
5	80	24	41	31
6	100	24	50	43

[a] Unless noted otherwise, reactions were performed with **1** (0.30 mmol), **2** (0.20 mmol), catalyst **3i** (0.02 mmol), *rac*-Segphos (0.024 mmol), $Ni(COD)_2$ (0.02 mmol), TMG (0.32mmol), and $ZnCl_2$ (0.08 mmol) in toluene (0.5 mL) at 50 ºC. [b]Isolated yield. [c]Determined by chiral HPLC analysis.

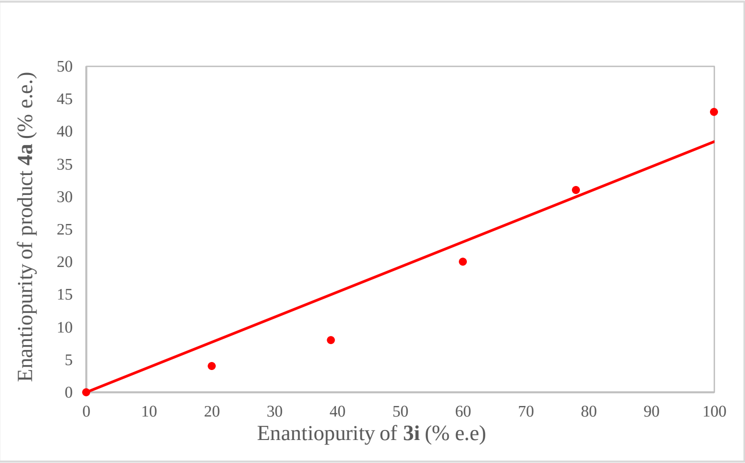

Supplementary Figure 2. The ee value relationship between **3i** and product **4a**.

1.6.3 With Schiff base as reactant

Supplementary Table 11: control experiments with Schiff base **1a-3i** as reactant[a]

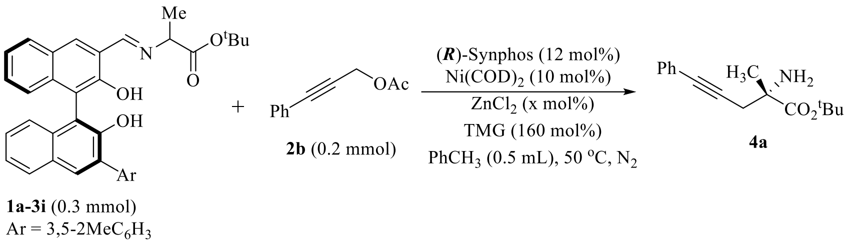

Entry	X(mol%)	T (h)	Yield (%)[b]	ee (%)[c]
1	0	24	trace	N.D.[d]
2	40	24	52	84
3	150	24	49	93

[a] Unless noted otherwise, reactions were performed with **1a-3i** (0.30 mmol), **2** (0.20 mmol), (***R***)-Synphos (0.024 mmol), Ni(COD)$_2$ (0.02 mmol), TMG (0.32 mmol), and $ZnCl_2$ (X mmol) in toluene (0.5 mL) at 50 °C. [b]Isolated yield. [c]Determined by chiral HPLC analysis. [d]N.D. = Not determined.

1.6.4 Intermediates detected by HRMS

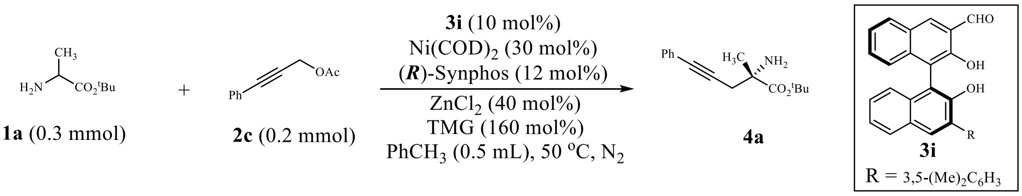

In a nitrogen-filled glove box, an oven-dried 10 mL screw-cap reaction tube equipped with a stir bar was charged with Ni(COD)$_2$ (5.5 mg, 0.02 mmol), (***R***)-Synphos (15.3 mg, 0.024 mmol) and stirred in toluene (0.5 mL) at r.t. for about 5 min; Then, tert-butyl amino acid ester **1a** (0.3 mmol, 43.5 mg), propargylic acetate ester **2c** (0.2 mmol, 35.0 mg), chiral aldehyde **3i** (25.1 mg, 0.06 mmol), $ZnCl_2$ (10.9 mg, 0.08 mmol) and TMG (36.8mg, 0.32 mmol) were added. The mixture was continuously stirred at 50 °C under nitrogen atmosphere. After 2h, a small part of the reaction

mixture was diluted with acetonitrile and submitted to HRMS detection immediately. Four key intermediates and their isotopic distributions were listed below.

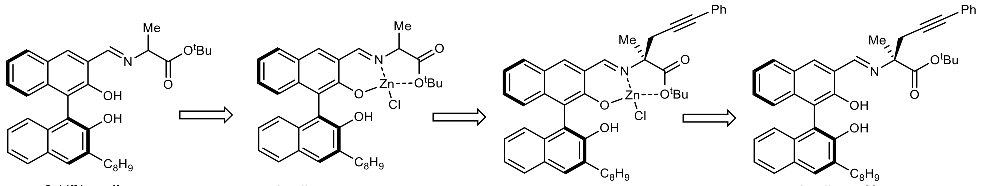

Schiff base **II** m/z = 544.2490 (M-H) | Schiff base-Zn complex **I** m/z = 642.1375 (M-H) | Schiff base-Zn complex **IV** m/z = 756.1843 (M-H) | Schiff base **V** m/z = 658.2978 (M-H)

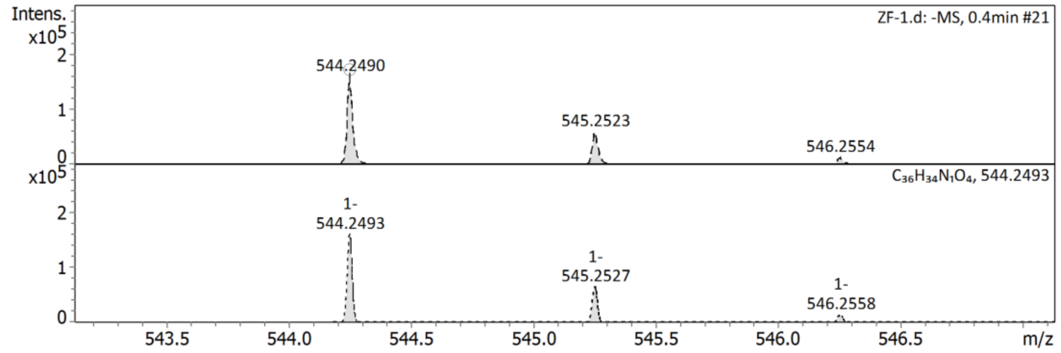

Meas. m/z	#	Sum Formula	m/z	Ion Formula	Adduct	err [ppm]	mSigma	Score	rdb	e⁻ Conf	N-Rule
544.2490	1	C36H35NO4	544.2493	C36H34NO4	M-H	0.6	14.9	100.00	20.0	even	ok

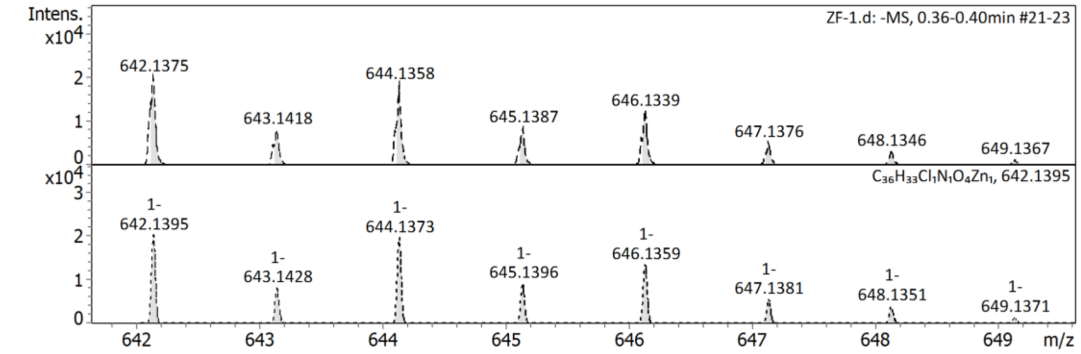

Meas. m/z	#	Sum Formula	m/z	Ion Formula	Adduct	err [ppm]	mSigma	Score	rdb	e⁻ Conf	N-Rule
642.1375	1	C36H34ClNO4Zn	642.1395	C36H33ClNO4Zn	M-H	3.2	34.5	100.00	23.0	even	ok

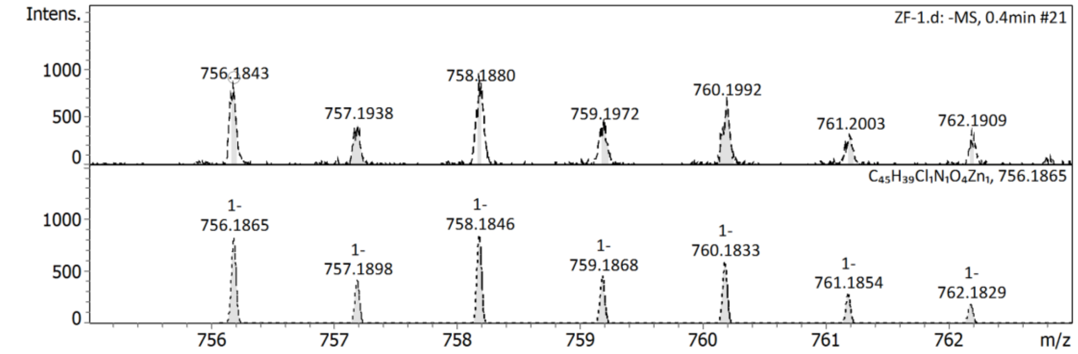

Meas. m/z	#	Sum Formula	m/z	Ion Formula	Adduct	err [ppm]	mSigma	Score	rdb	e⁻ Conf	N-Rule
756.1843	1	C45H40ClNO4Zn	756.1865	C45H39ClNO4Zn	M-H	2.9	337.1	100.00	29.0	even	ok

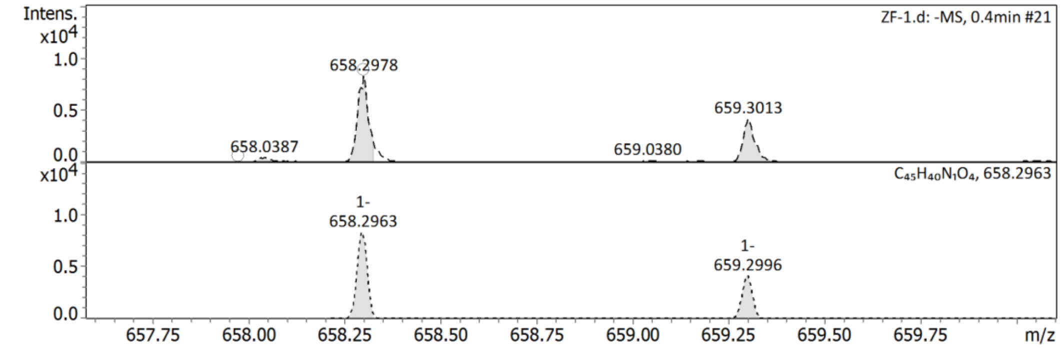

Meas. m/z	#	Sum Formula	m/z	Ion Formula	Adduct	err [ppm]	mSigma	Score	rdb	e⁻ Conf	N-Rule
658.2978	1	C45H41NO4	658.2963	C45H40NO4	M-H	-2.3	497.1	100.00	26.0	even	ok

Supplementary Figure 3. HRMS data of the key intermediates

1.7 Copies of NMR spectra

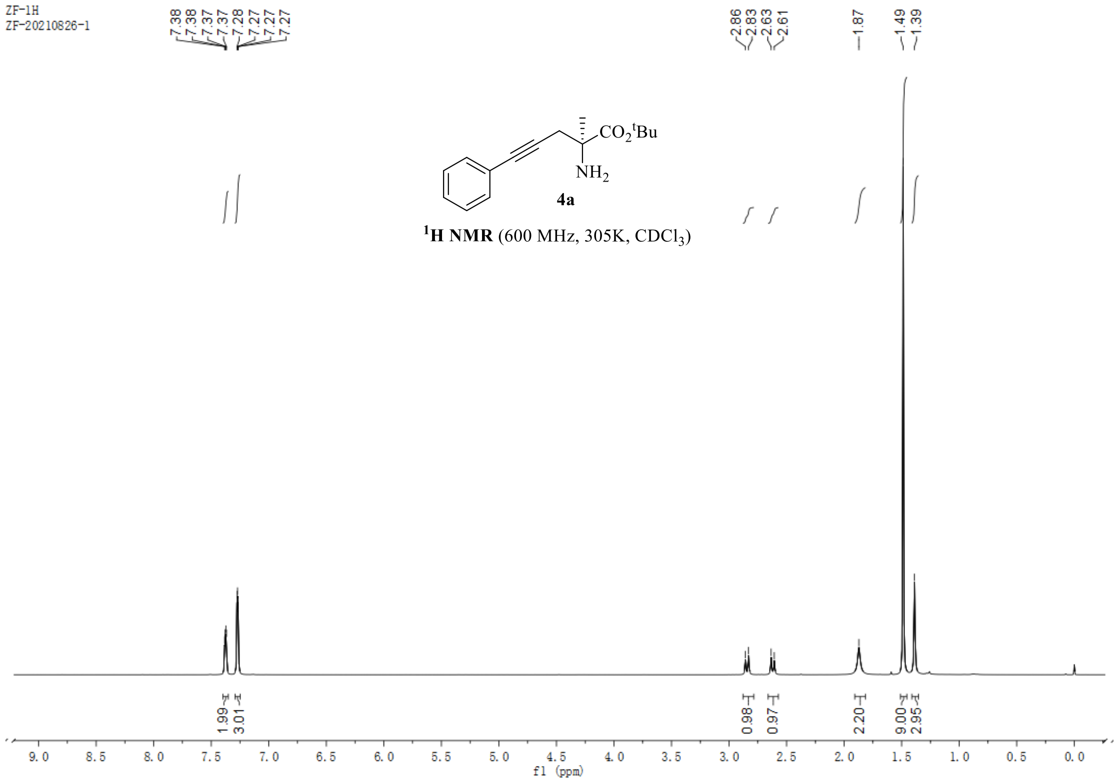

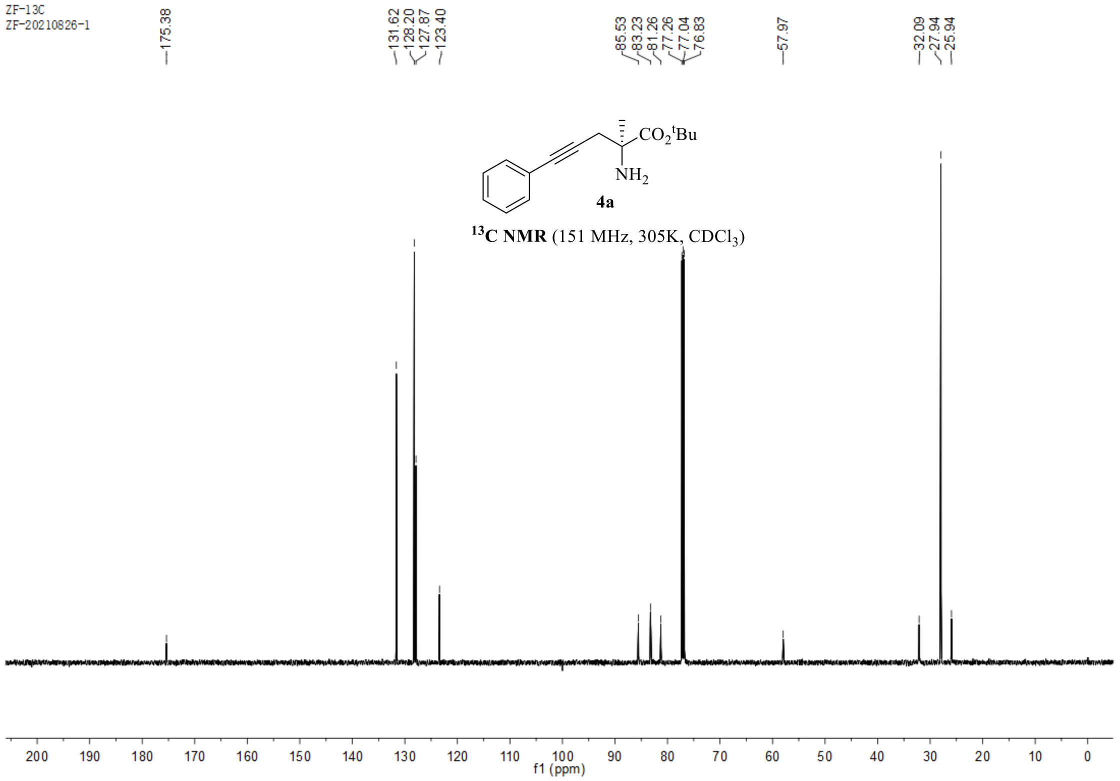

Supplementary Figure 4: NMR of compound 4a.

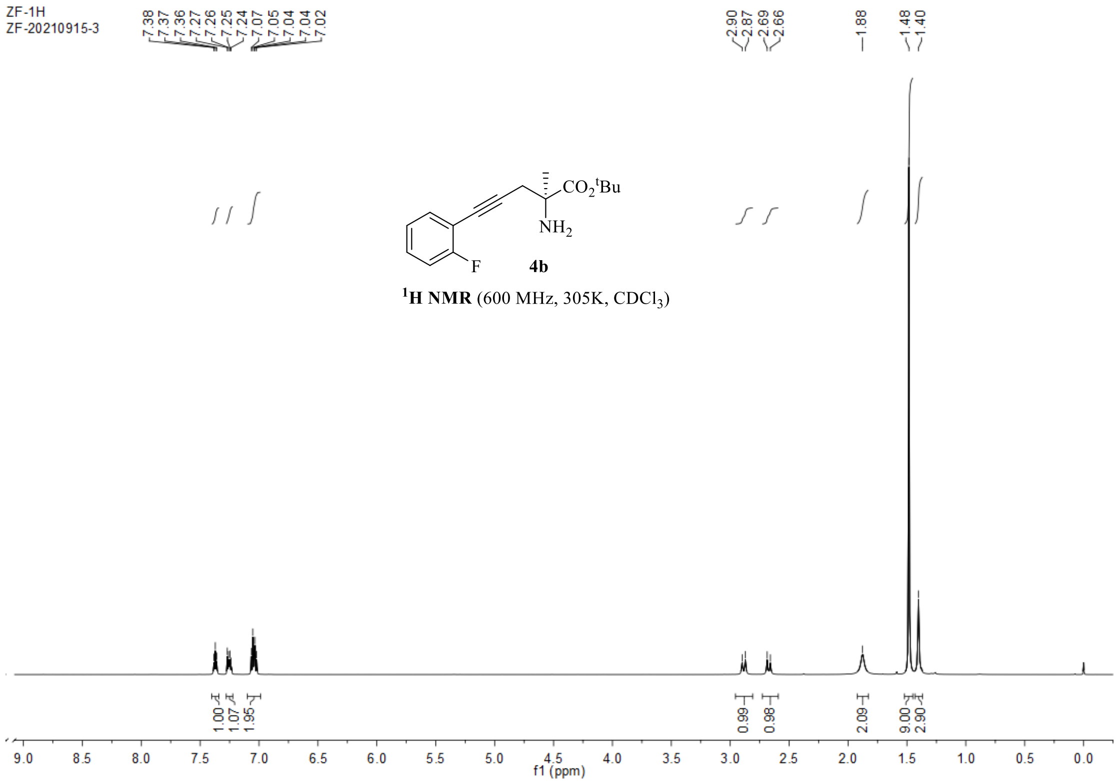
ZF-1H
ZF-20210915-3
7.38 7.37 7.36 7.27 7.26 7.25 7.24 7.07 7.05 7.04 7.04 7.02
2.90 2.87 2.69 2.66
1.88
1.48 1.40
CO2tBu
NH2
F
4b
1H NMR (600 MHz, 305K, CDCl3)
1.00 1.07 1.95
0.99 0.98
2.09
9.00 2.90
f1 (ppm)

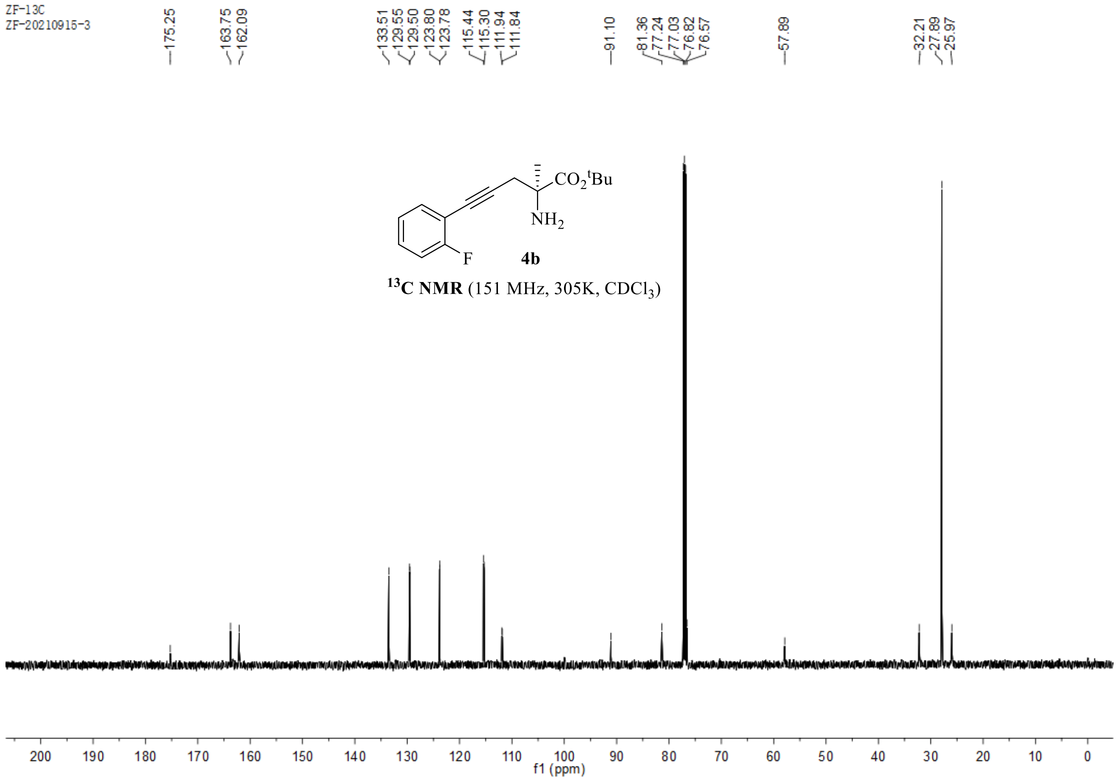
ZF-13C
ZF-20210915-3
175.25
163.75 162.09
133.51 129.55 129.50 123.80 123.78 115.44 115.30 111.94 111.84
91.10
81.36 77.24 77.03 76.82 76.57
57.89
32.21 27.89 25.97
CO2tBu
NH2
F
4b
13C NMR (151 MHz, 305K, CDCl3)
f1 (ppm)

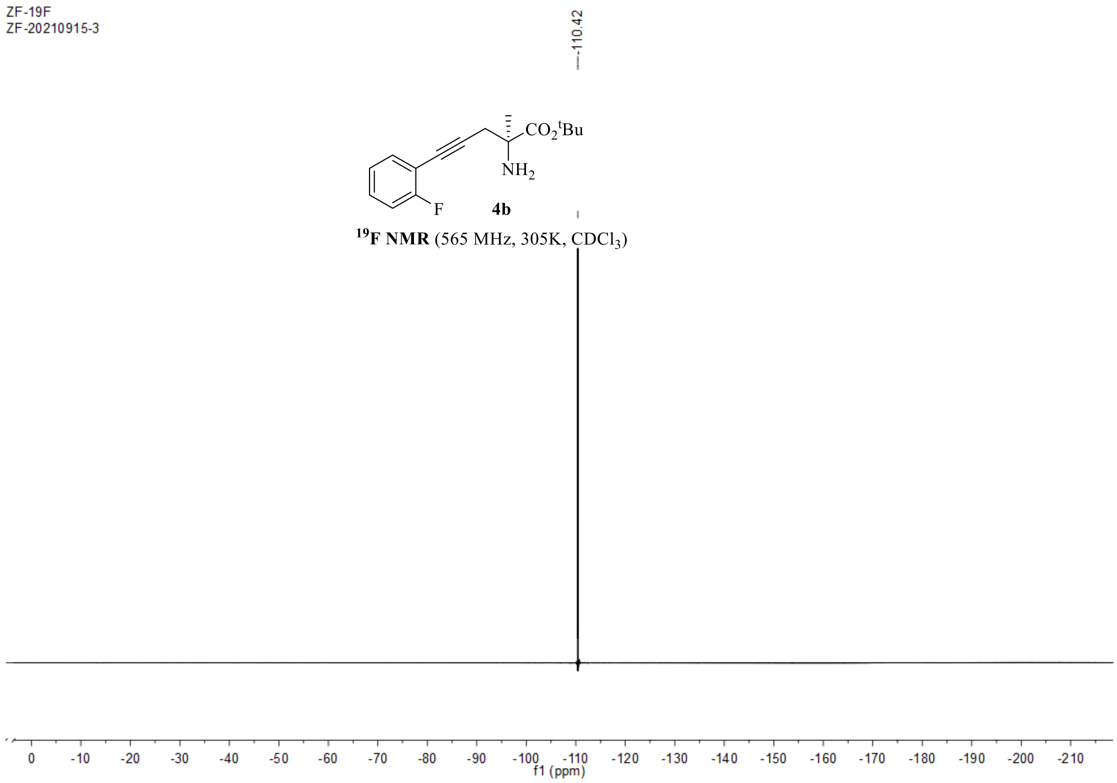

Supplementary Figure 5: NMR of compound 4b.

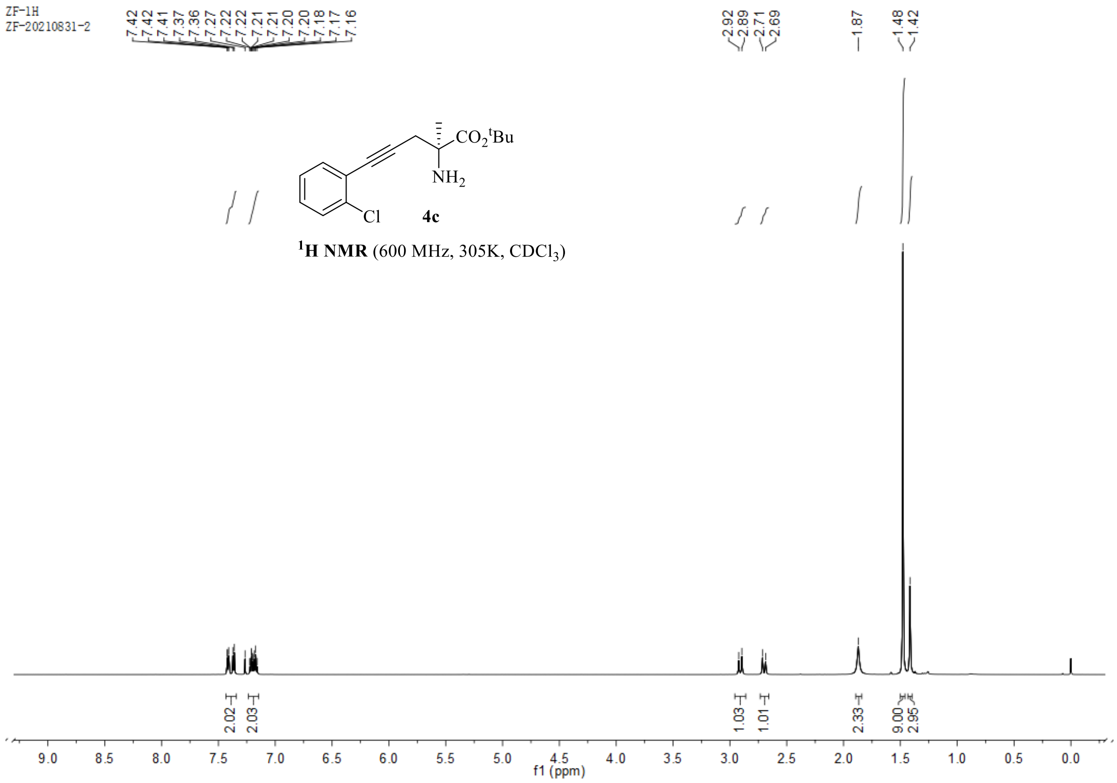

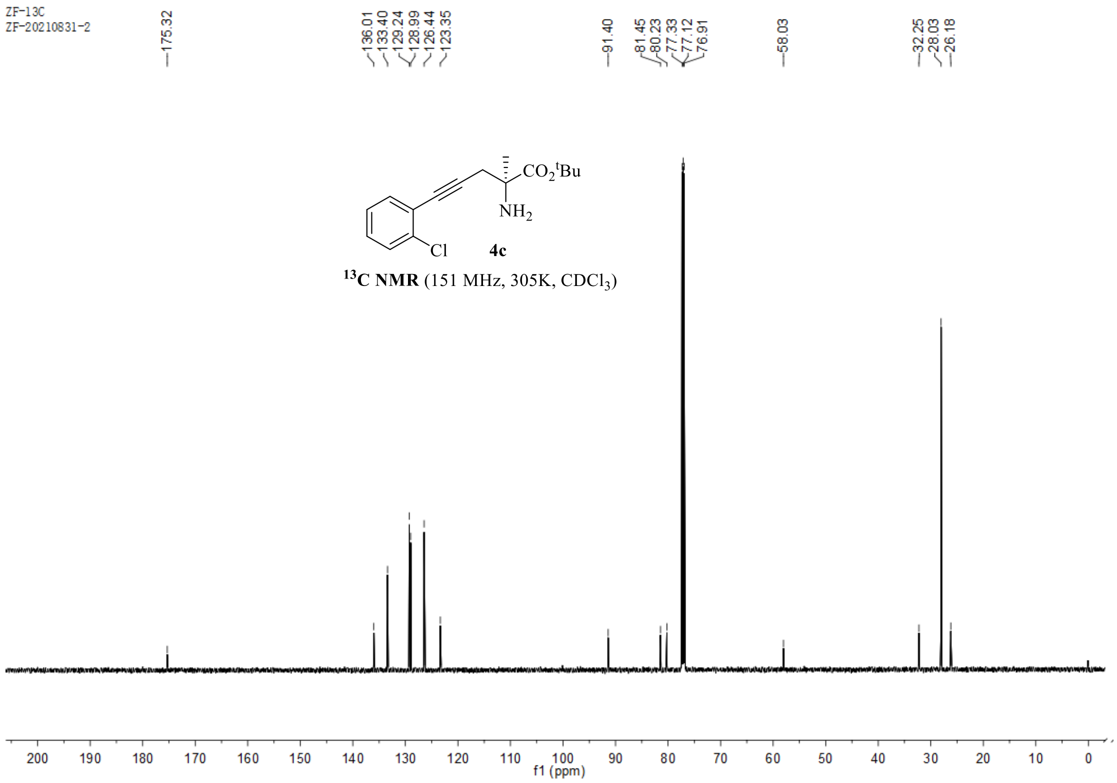

Supplementary Figure 6: NMR of compound 4c.

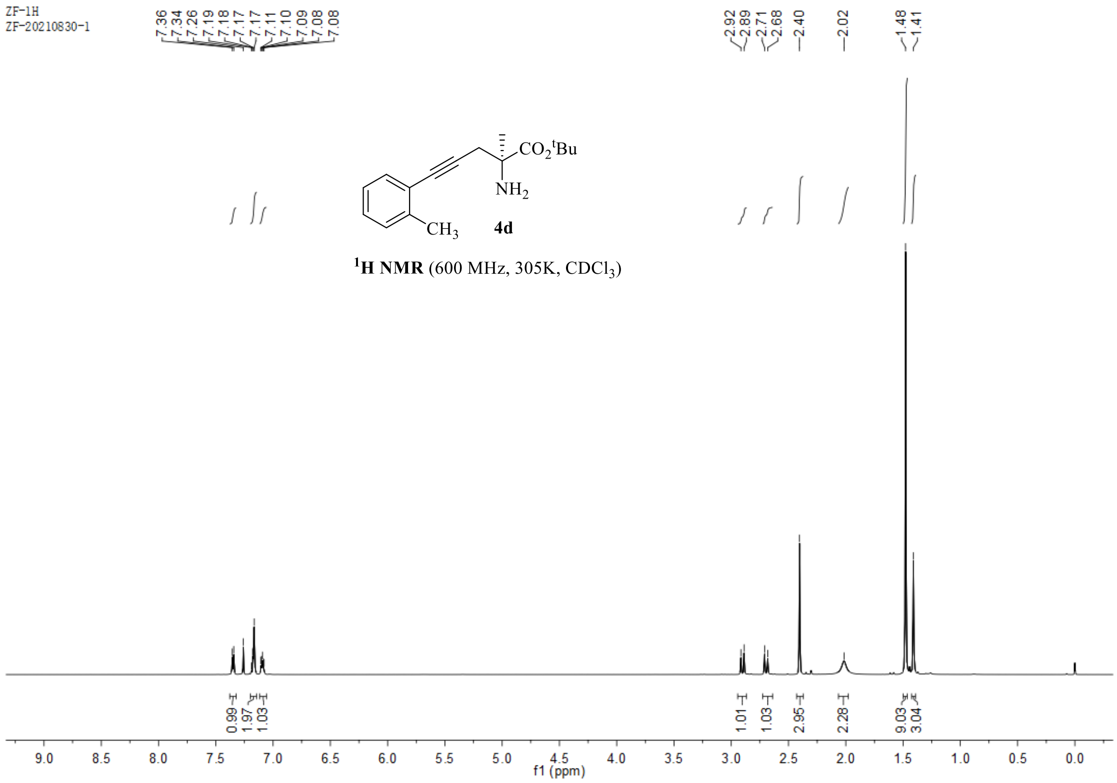

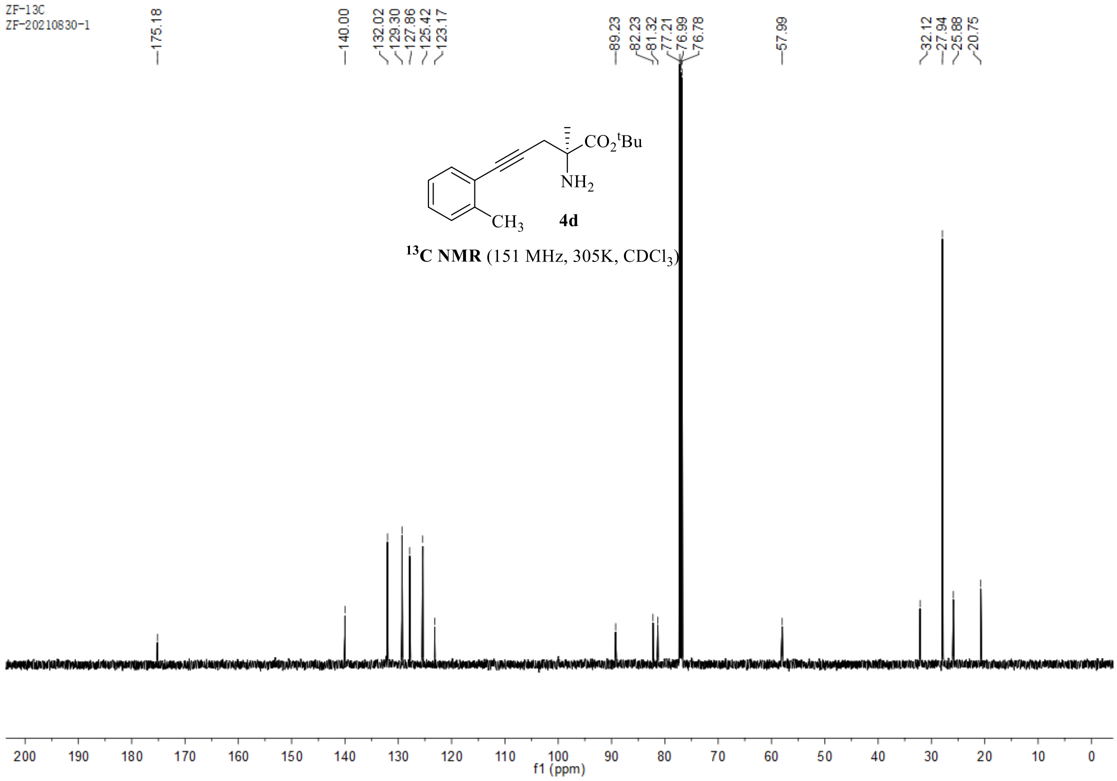

Supplementary Figure 7: NMR of compound 4d.

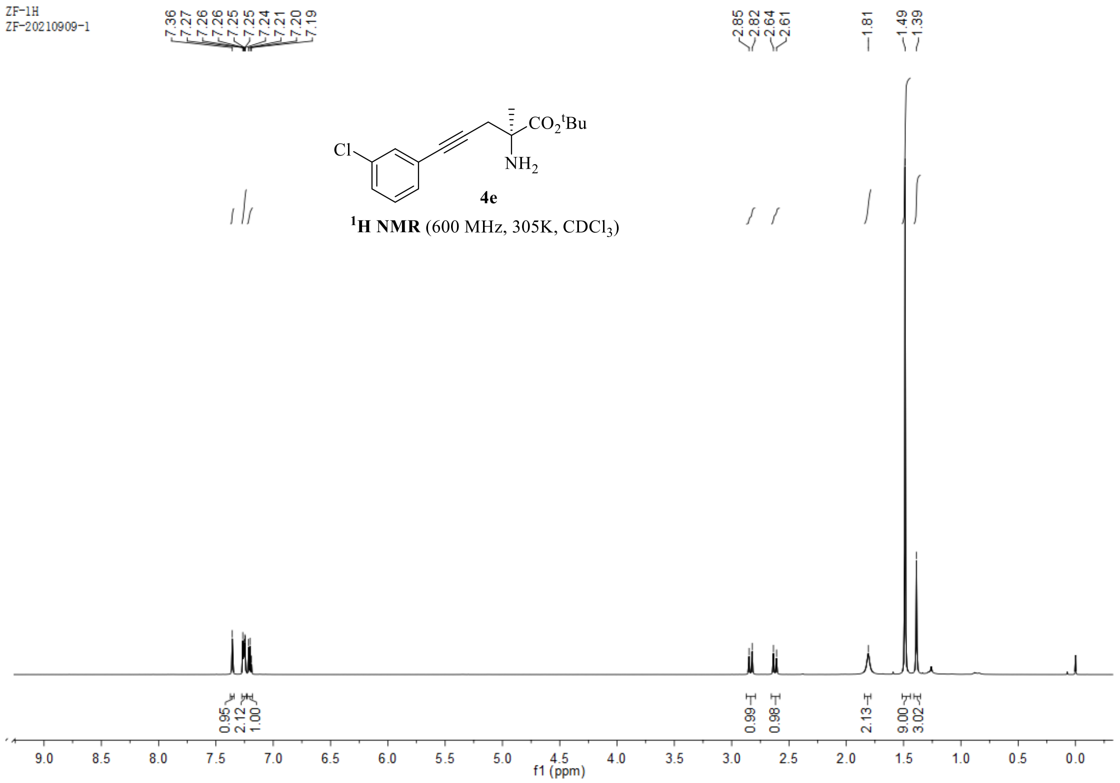

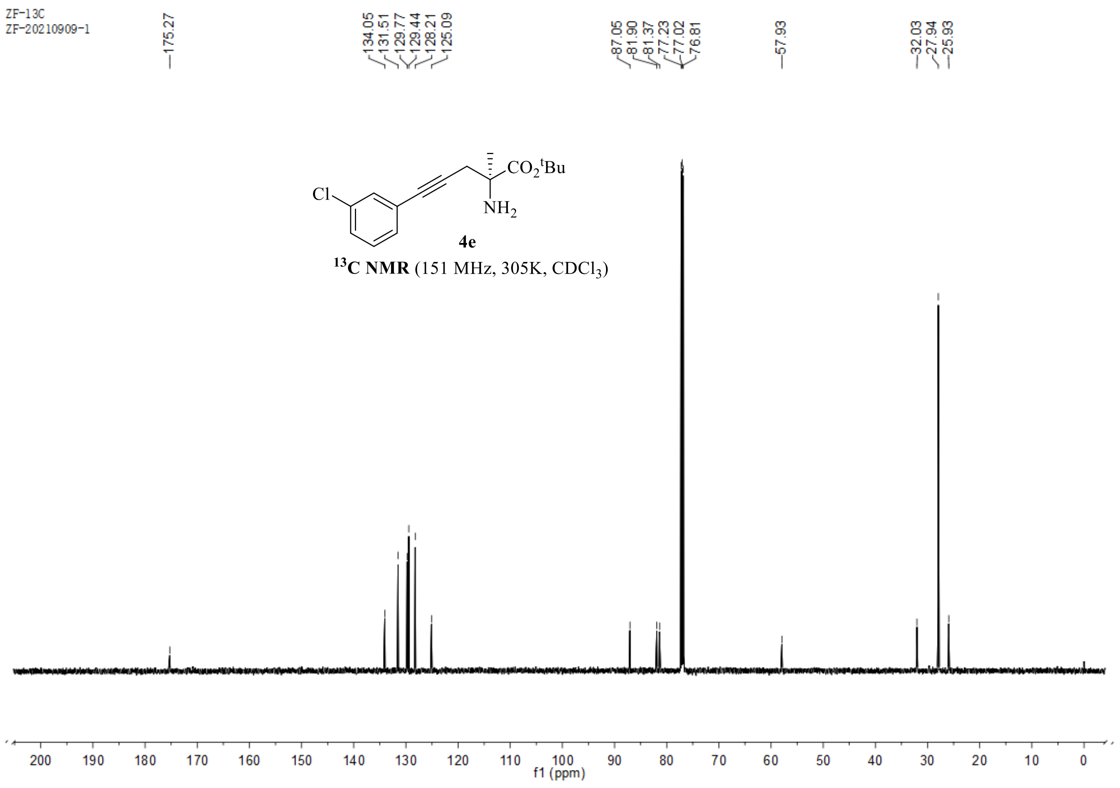

Supplementary Figure 8: NMR of compound 4e.

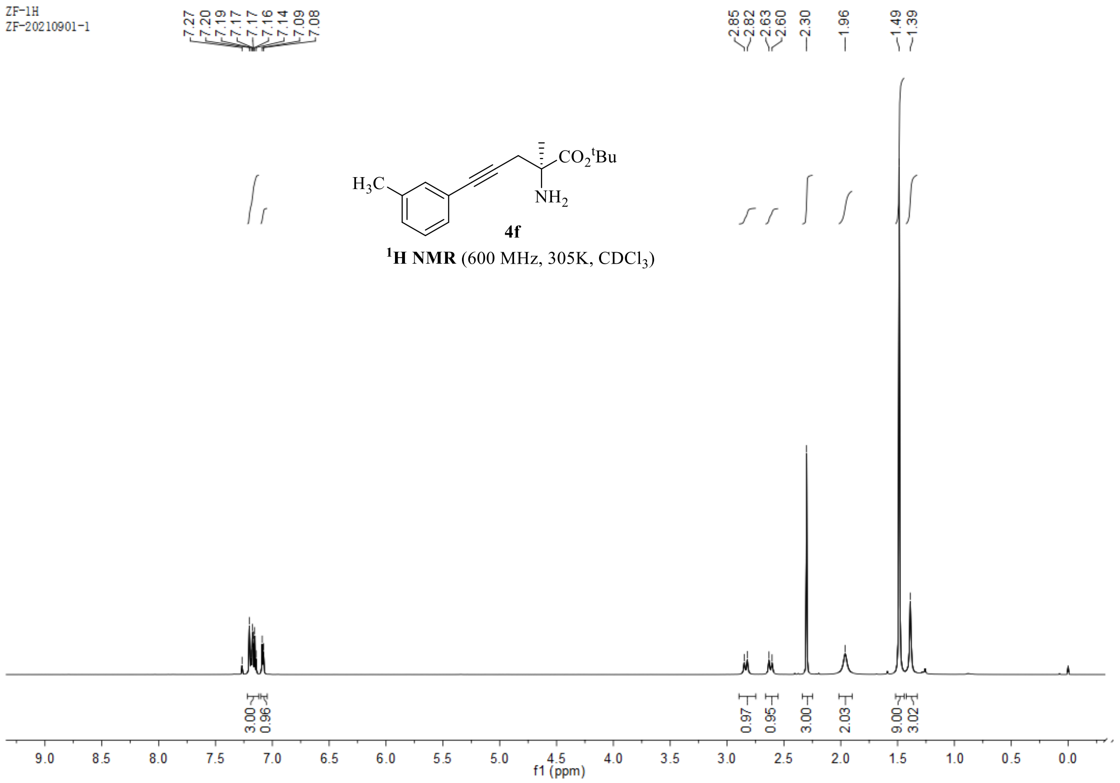

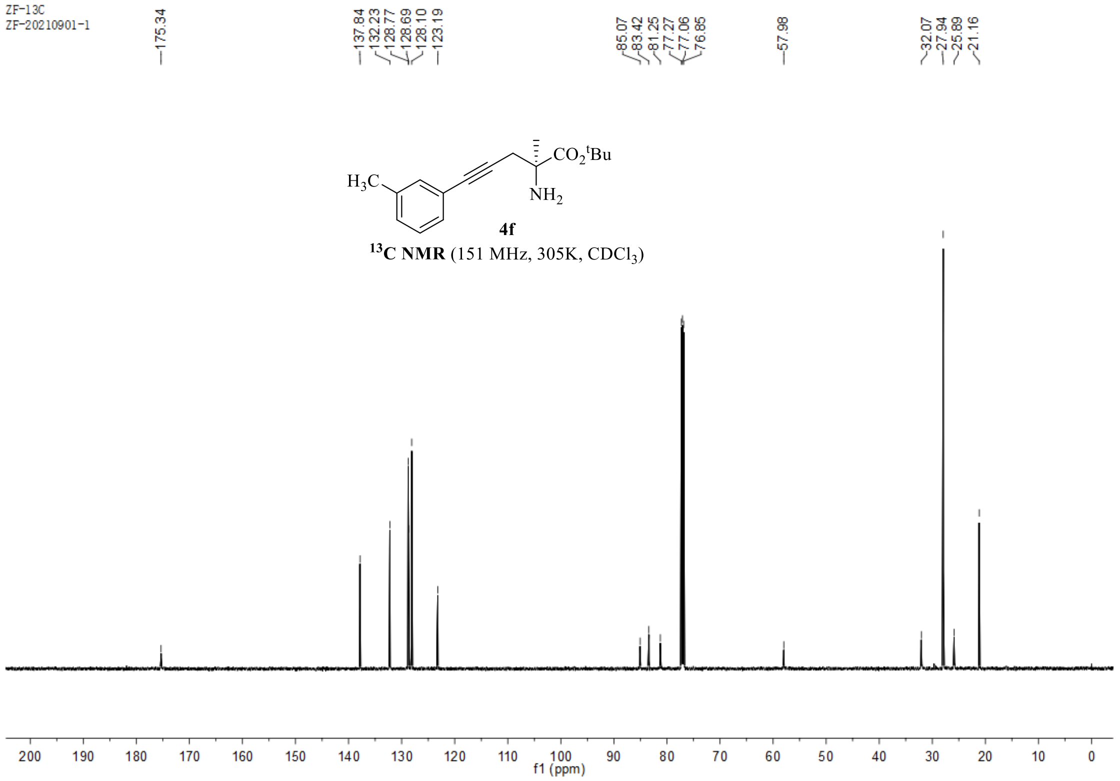

Supplementary Figure 9: NMR of compound 4f.

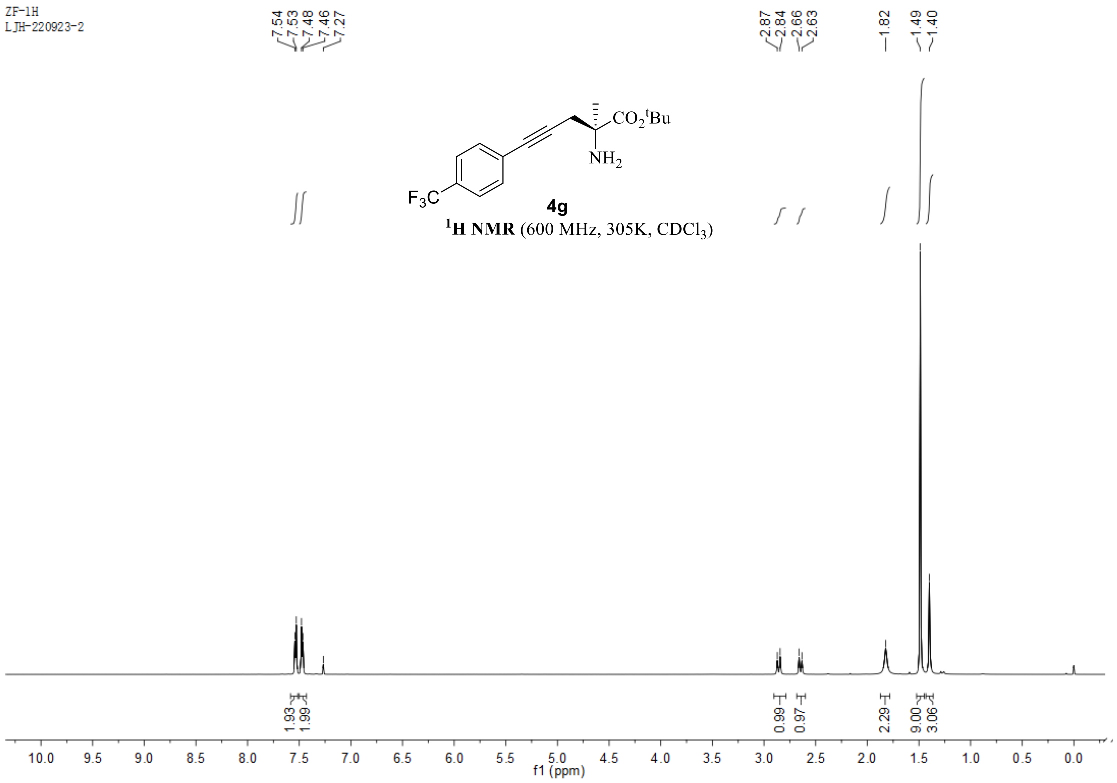
ZF-1H
LJH-220923-2
7.54
7.53
7.48
7.46
7.27
2.87
2.84
2.66
2.63
1.82
1.49
1.40
CO2tBu
NH2
F3C
4g
1H NMR (600 MHz, 305K, CDCl3)
1.93
1.99
0.99
0.97
2.29
9.00
3.06
10.0 9.5 9.0 8.5 8.0 7.5 7.0 6.5 6.0 5.5 5.0 4.5 4.0 3.5 3.0 2.5 2.0 1.5 1.0 0.5 0.0
f1 (ppm)

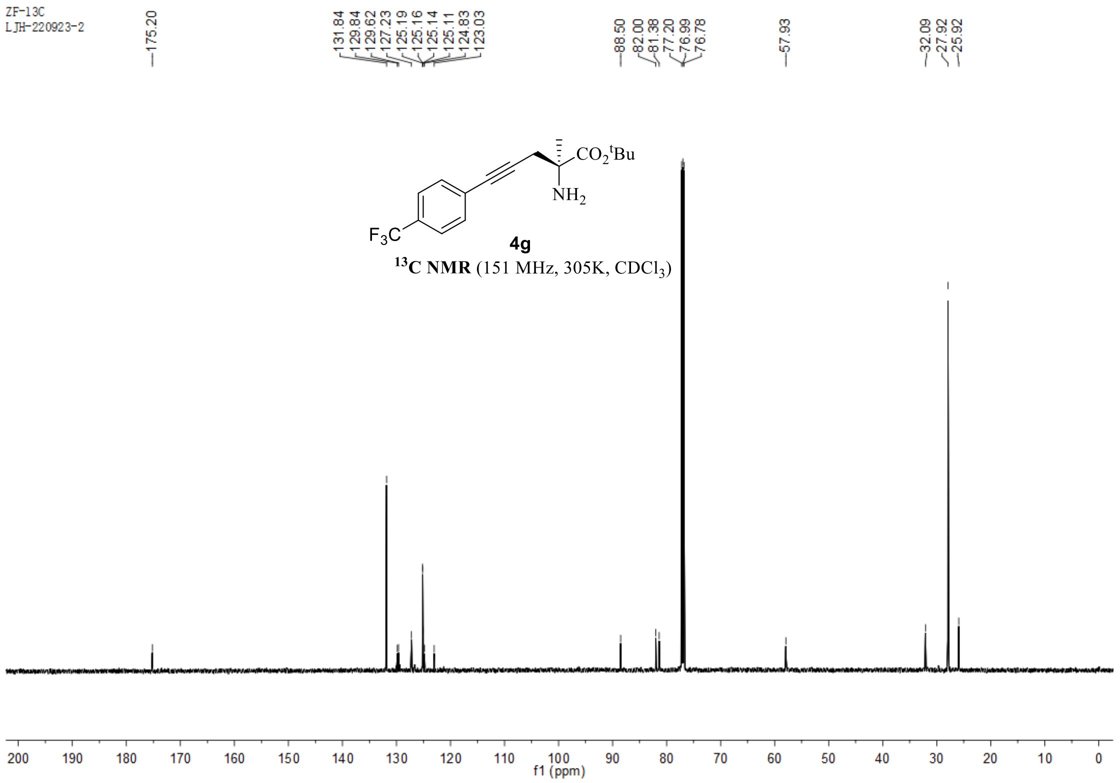
ZF-13C
LJH-220923-2
175.20
131.84
129.84
129.62
127.23
125.19
125.16
125.14
125.11
124.83
123.03
88.50
82.00
81.38
77.20
76.99
76.78
57.93
32.09
27.92
25.92
CO2tBu
NH2
F3C
4g
13C NMR (151 MHz, 305K, CDCl3)
200 190 180 170 160 150 140 130 120 110 100 90 80 70 60 50 40 30 20 10 0
f1 (ppm)

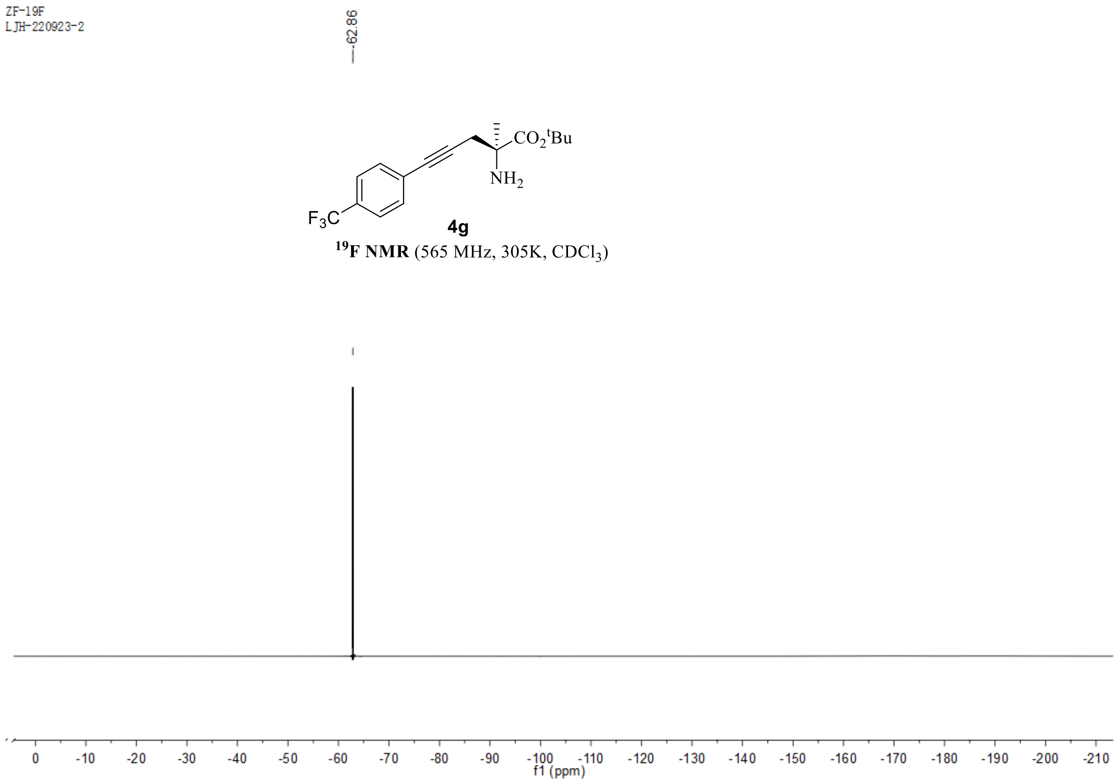

Supplementary Figure 10: NMR of compound 4g.

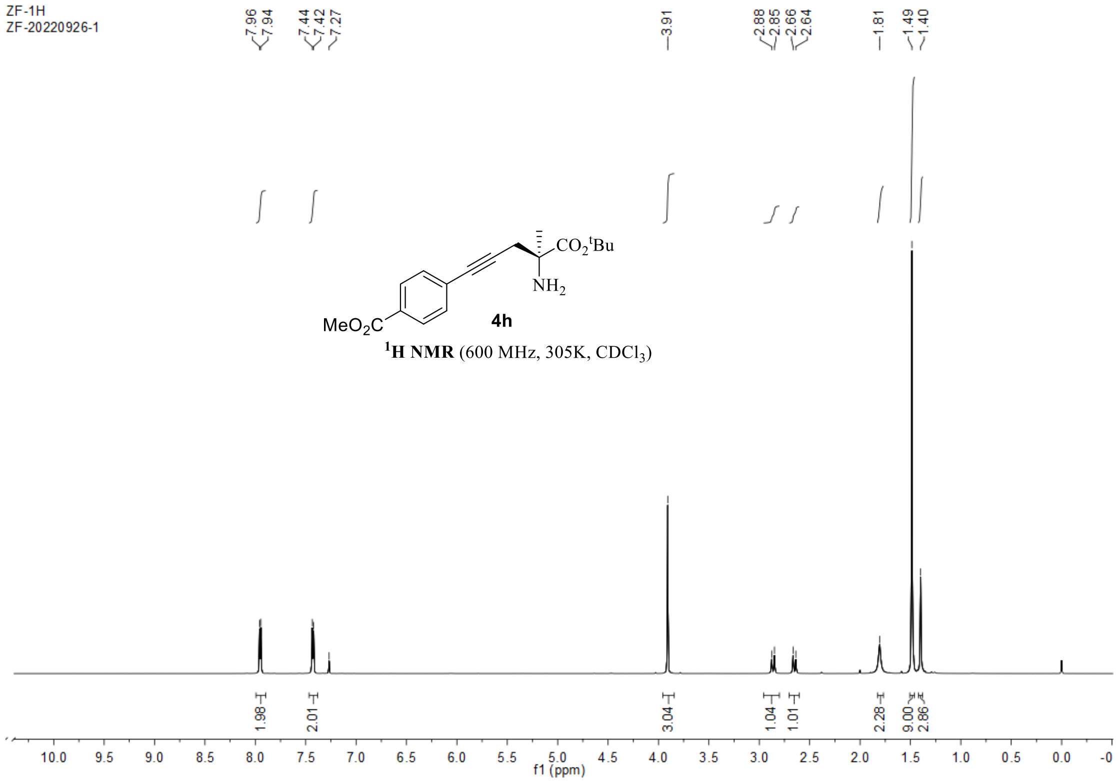

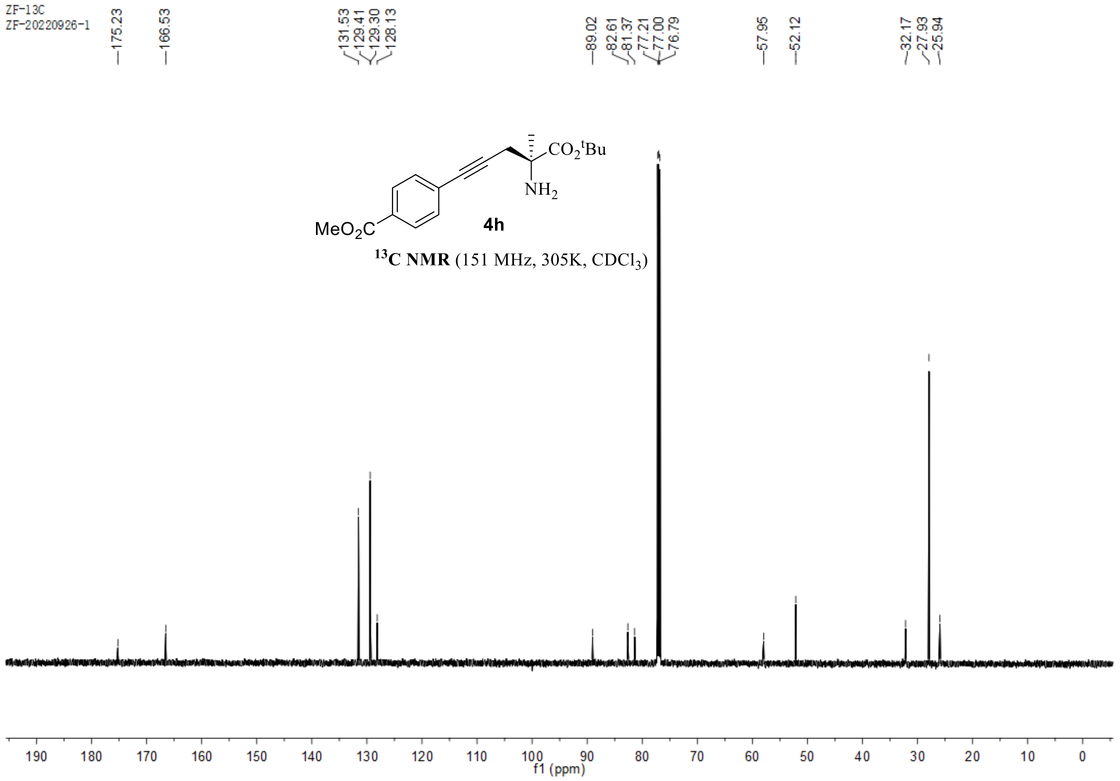

Supplementary Figure 11: NMR of compound 4h.

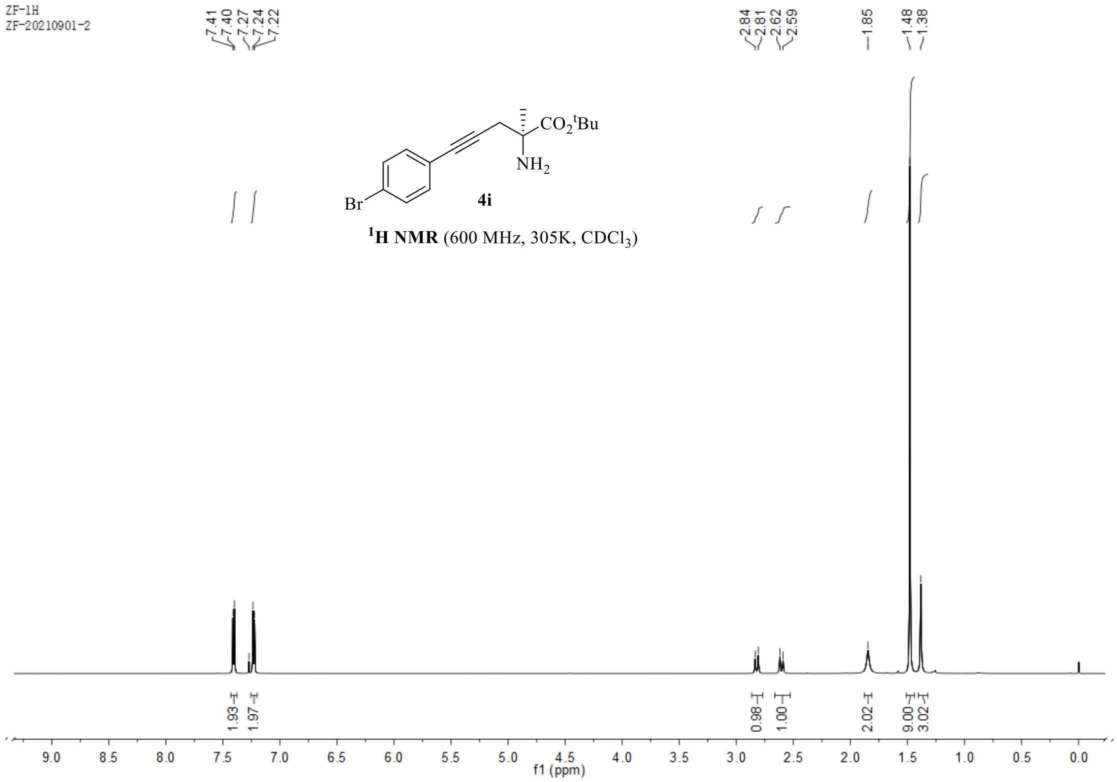

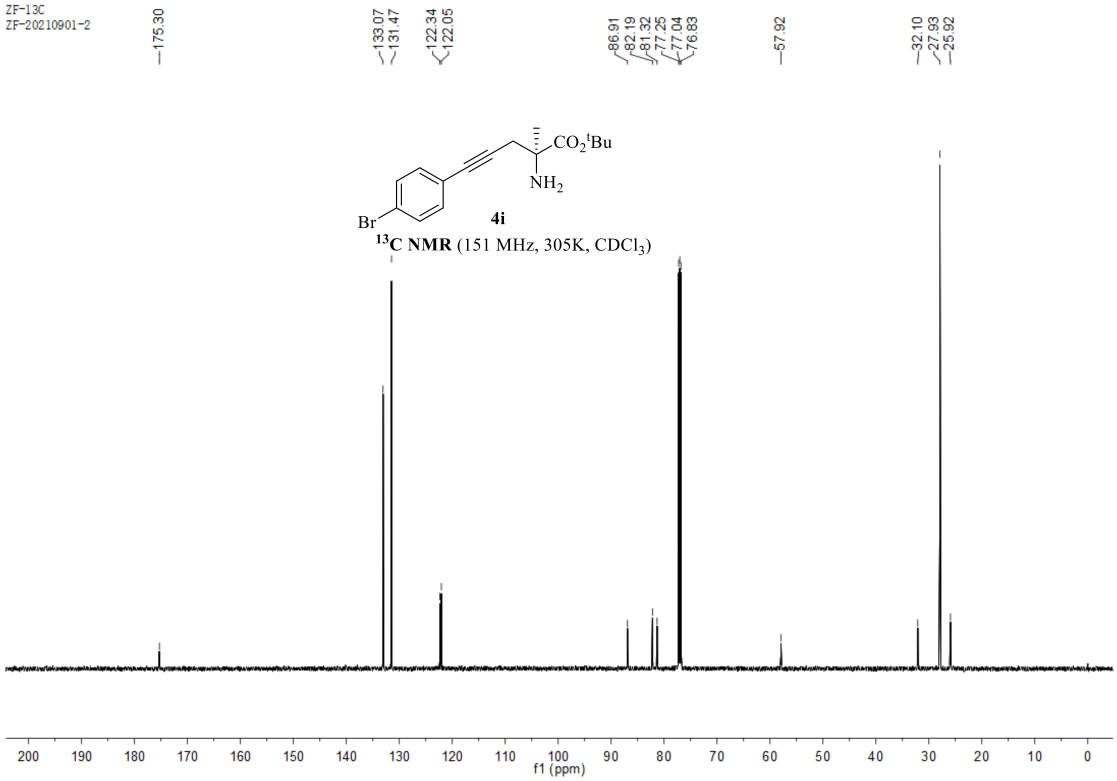

Supplementary Figure 12: NMR of compound 4i.

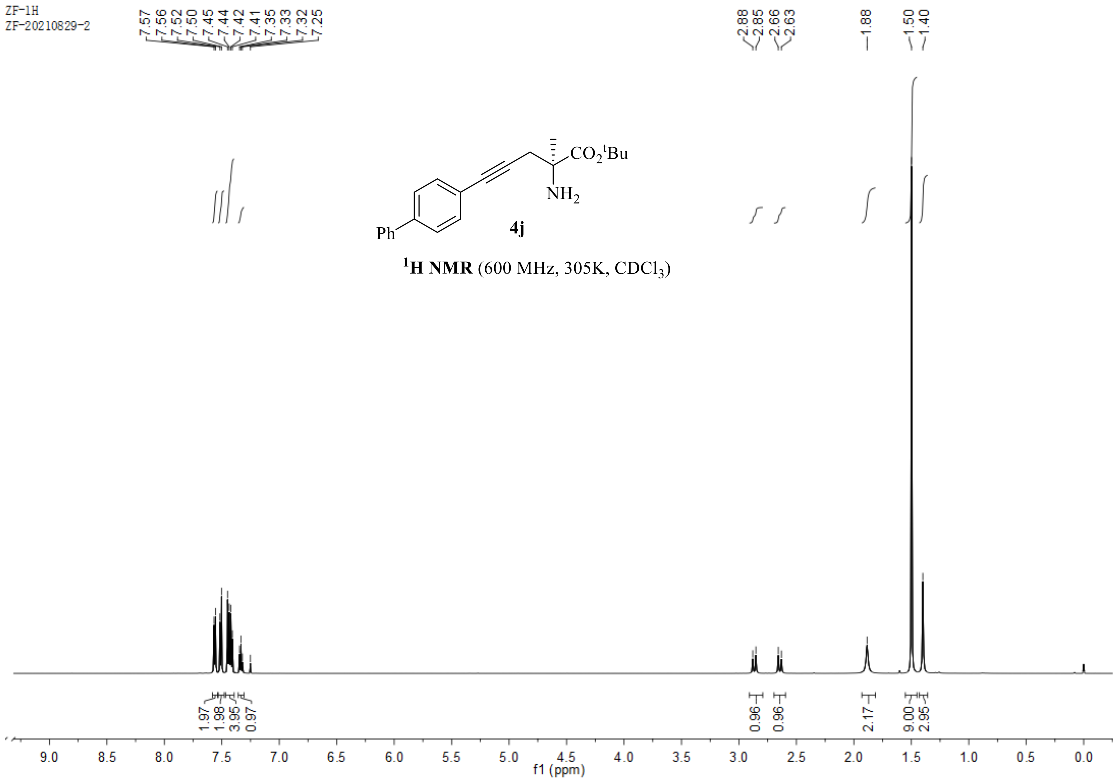

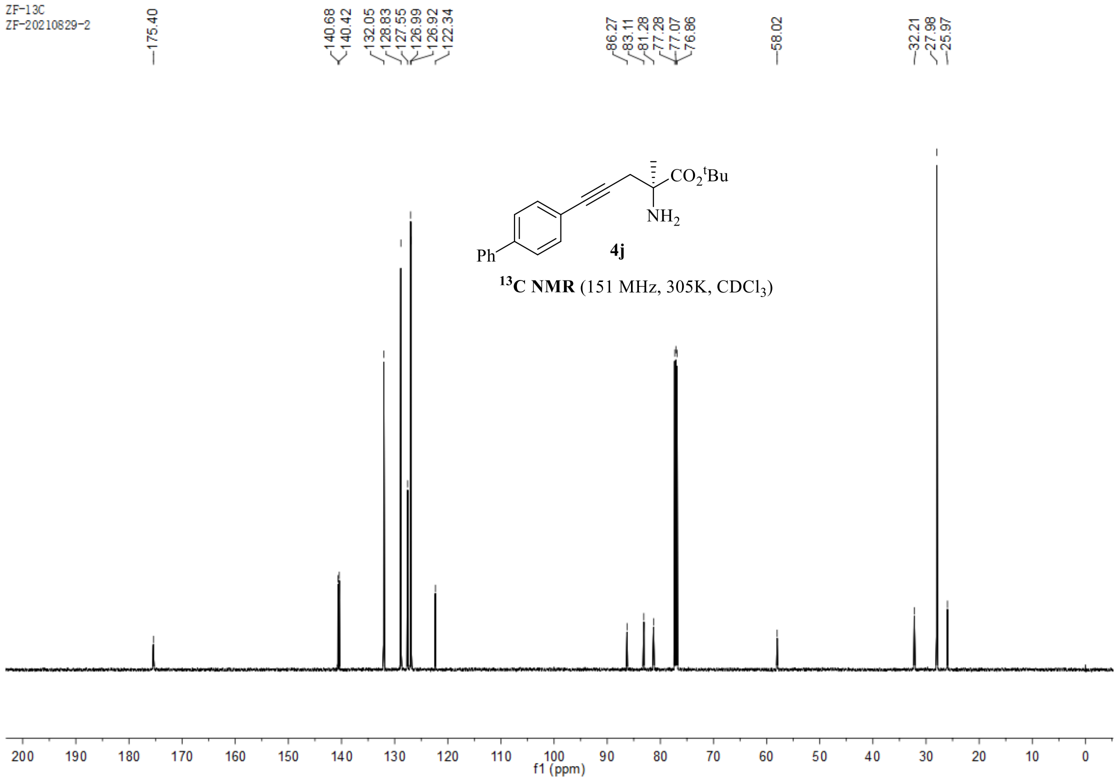

Supplementary Figure 13: NMR of compound 4j.

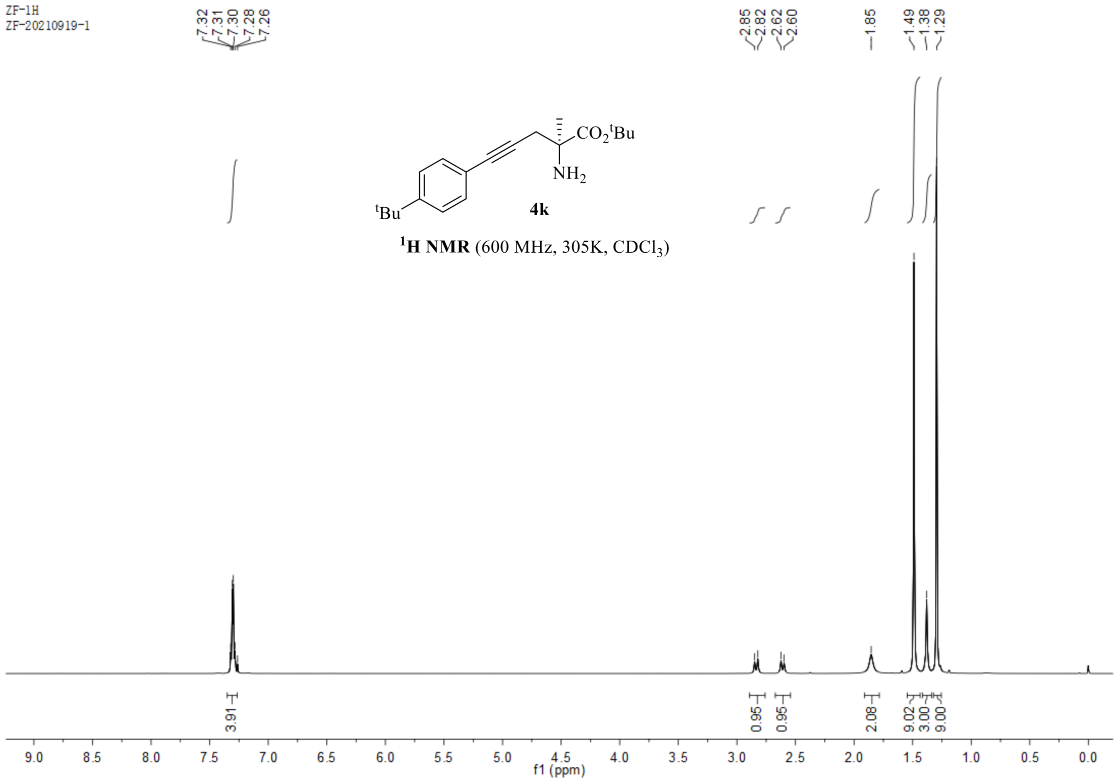

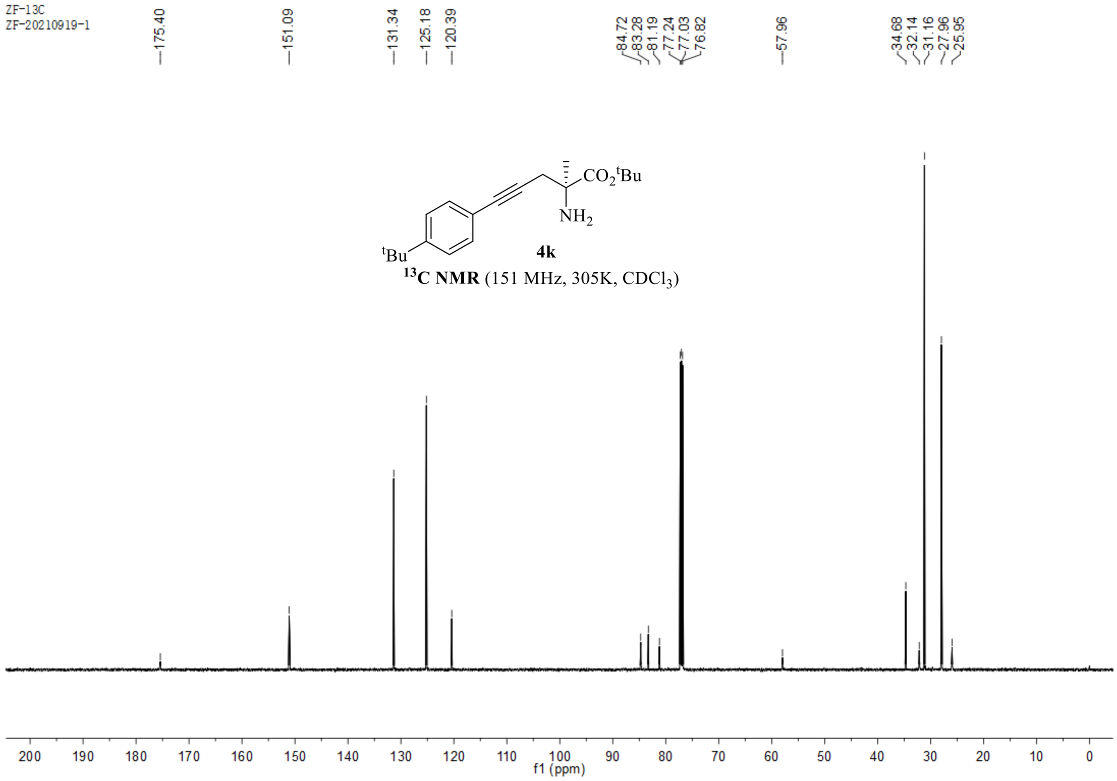

Supplementary Figure 14: NMR of compound 4k.

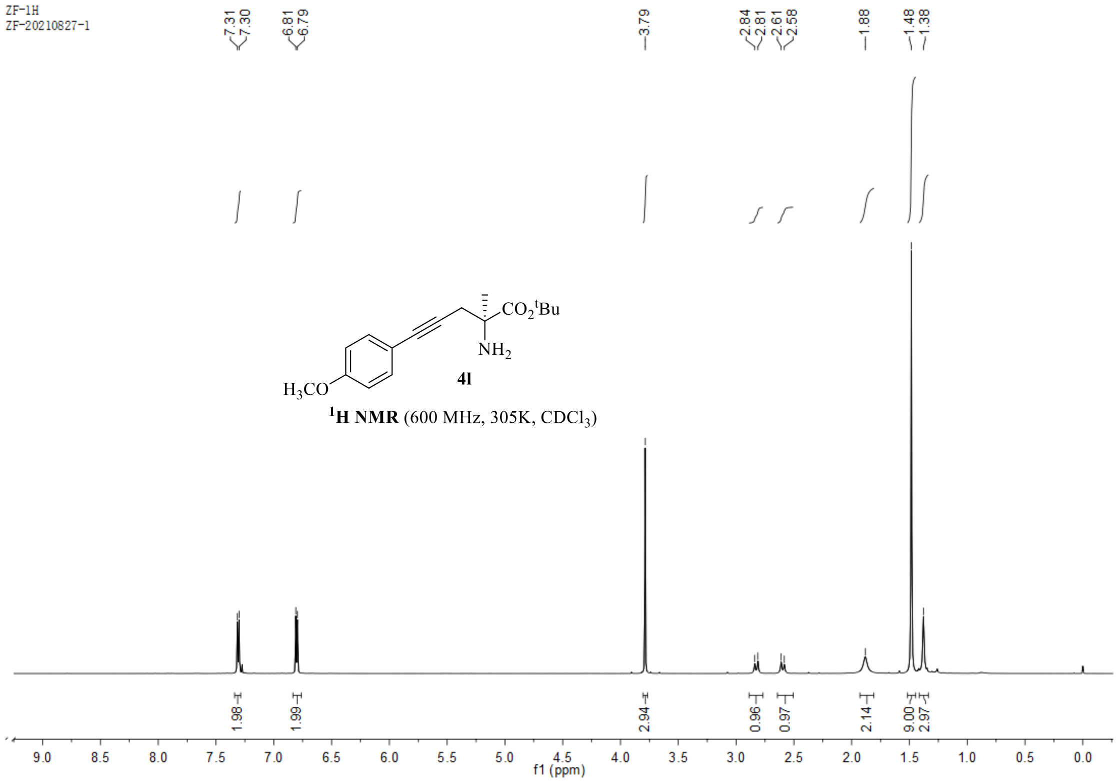

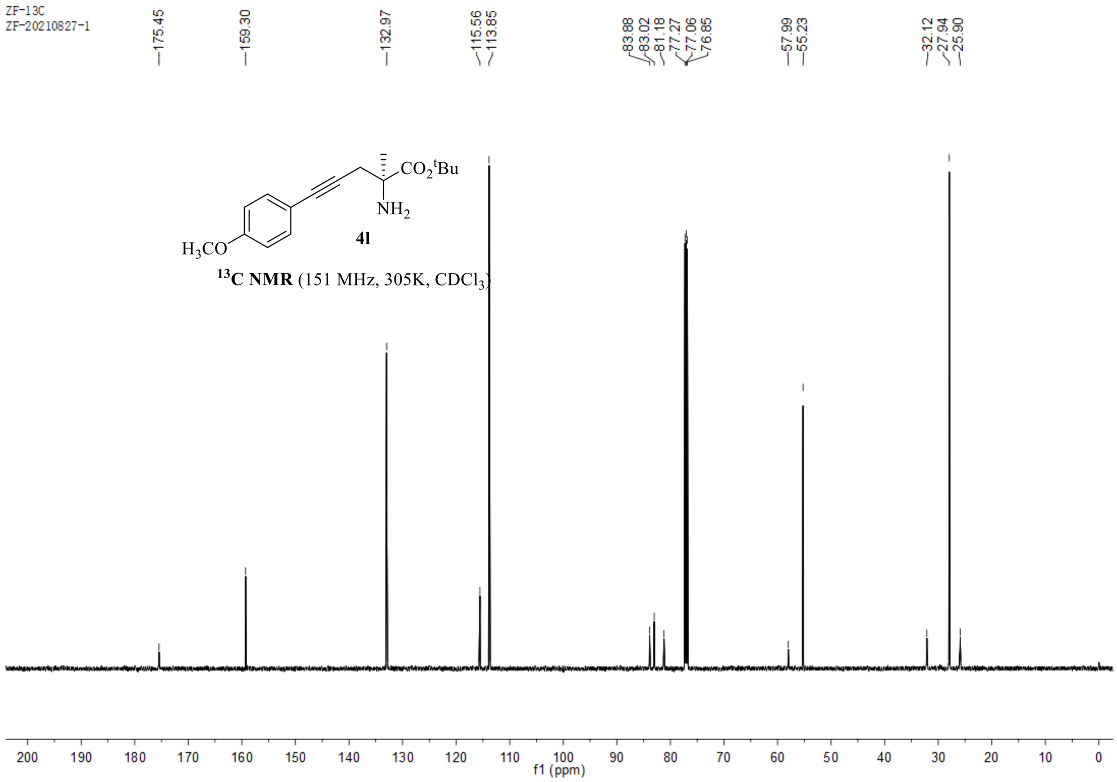

Supplementary Figure 15: NMR of compound 4l.

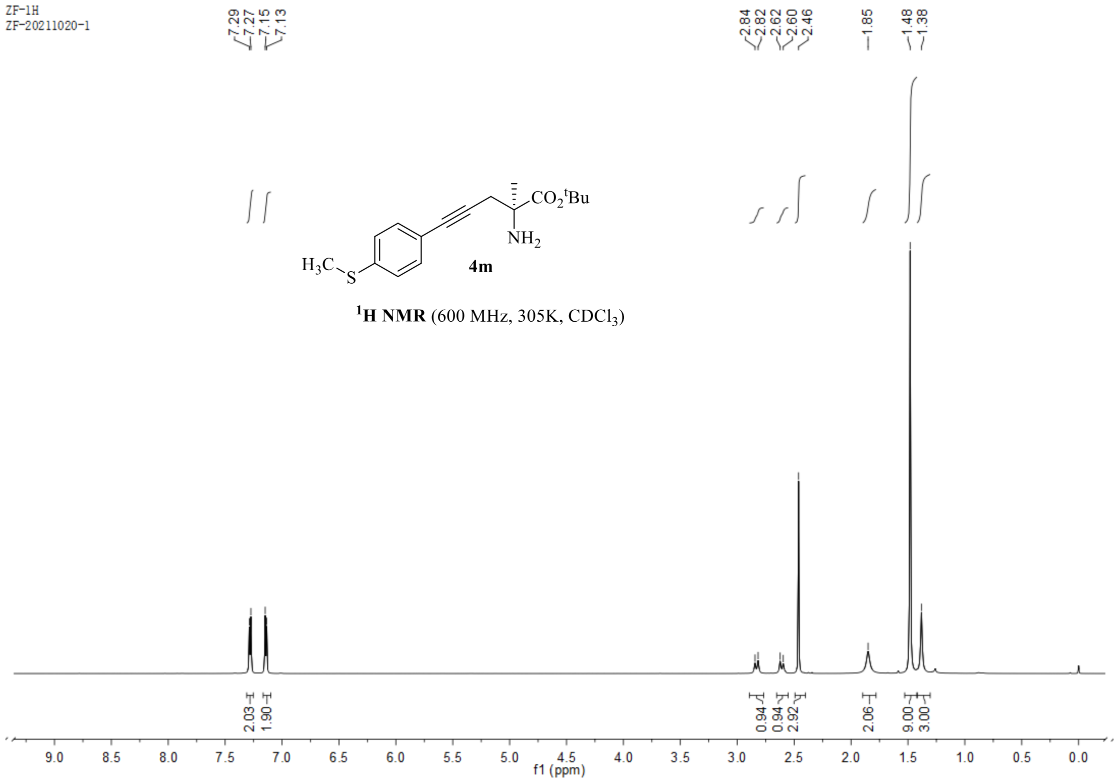

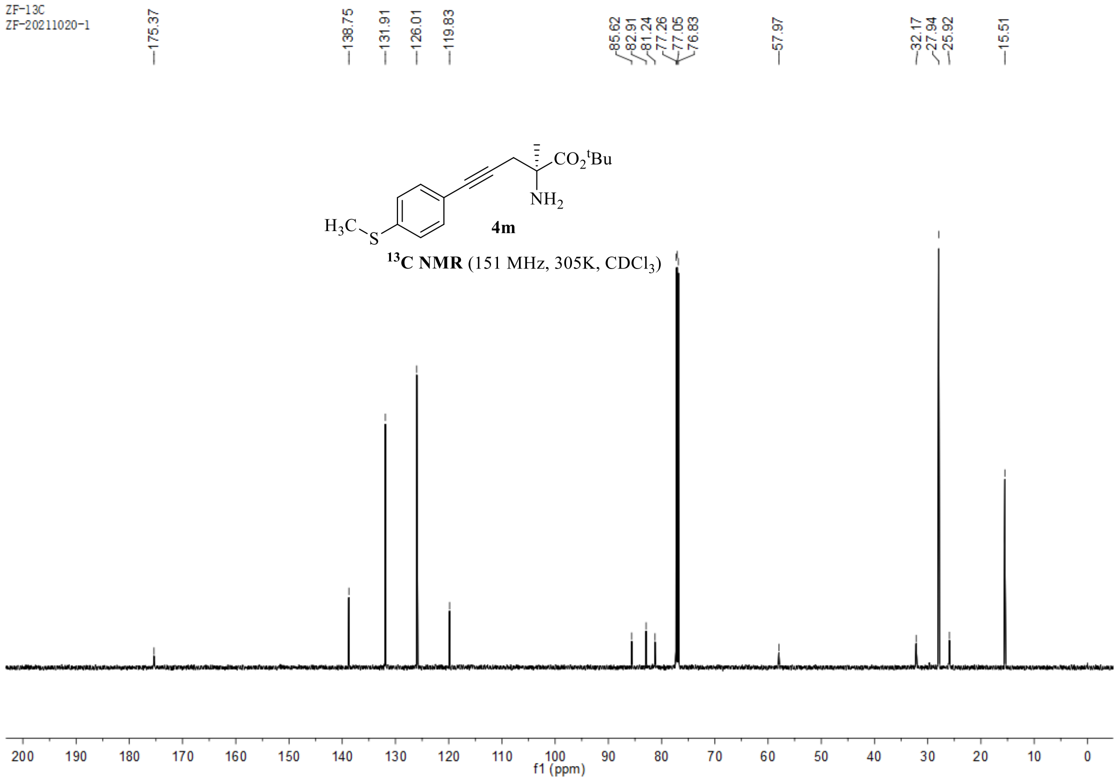

Supplementary Figure 16: NMR of compound 4m.

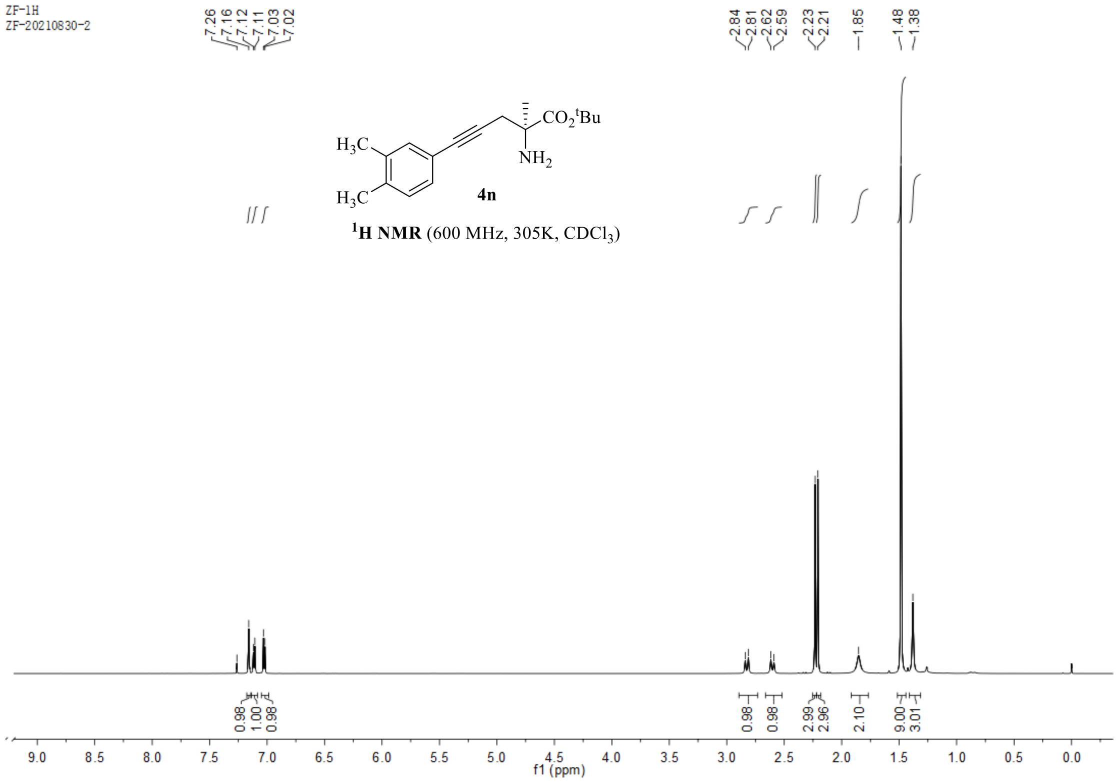

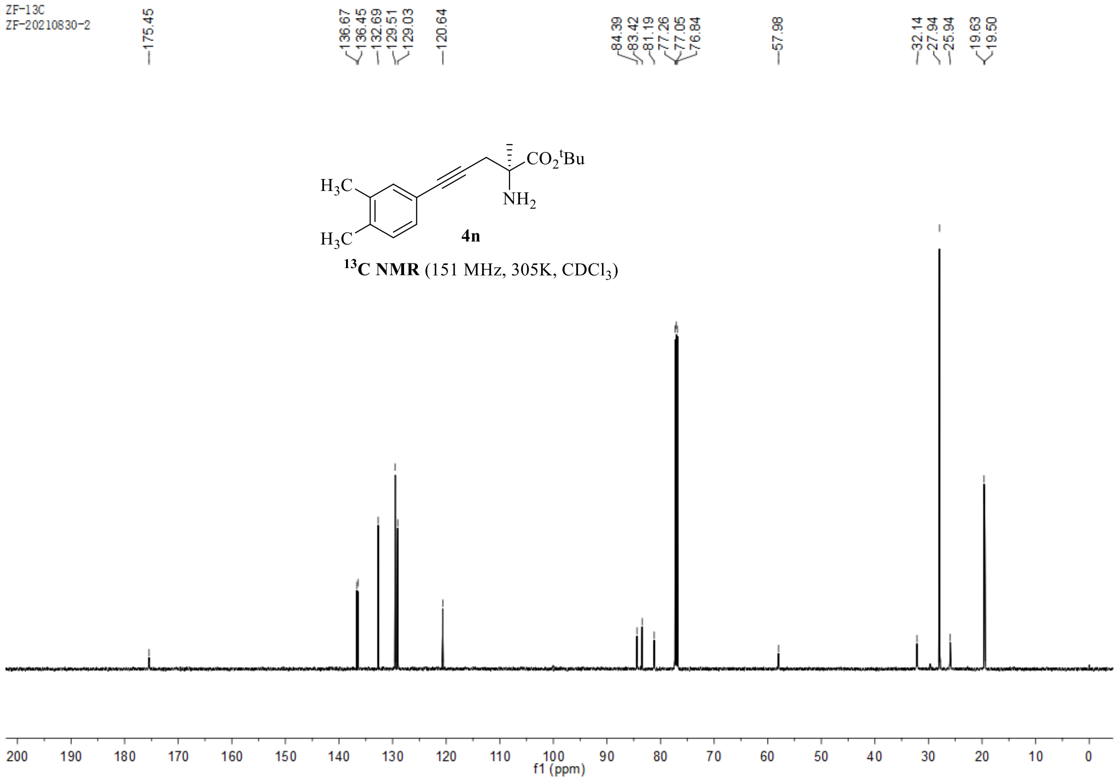

Supplementary Figure 17: NMR of compound 4n.

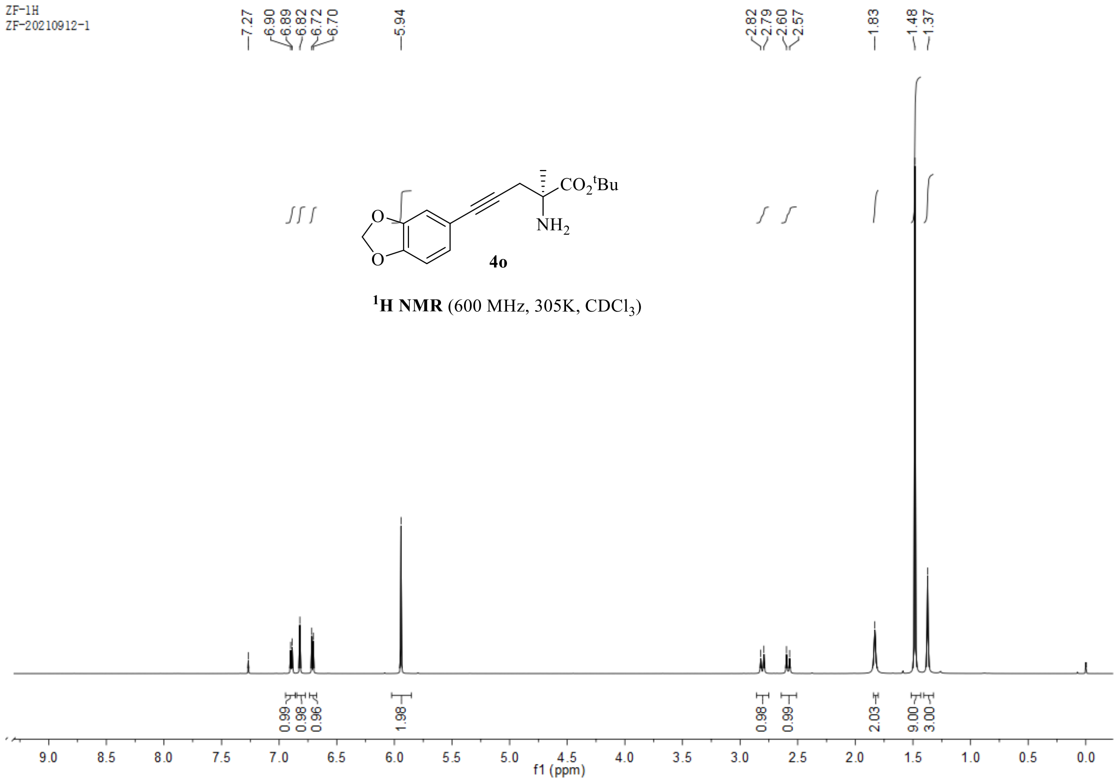

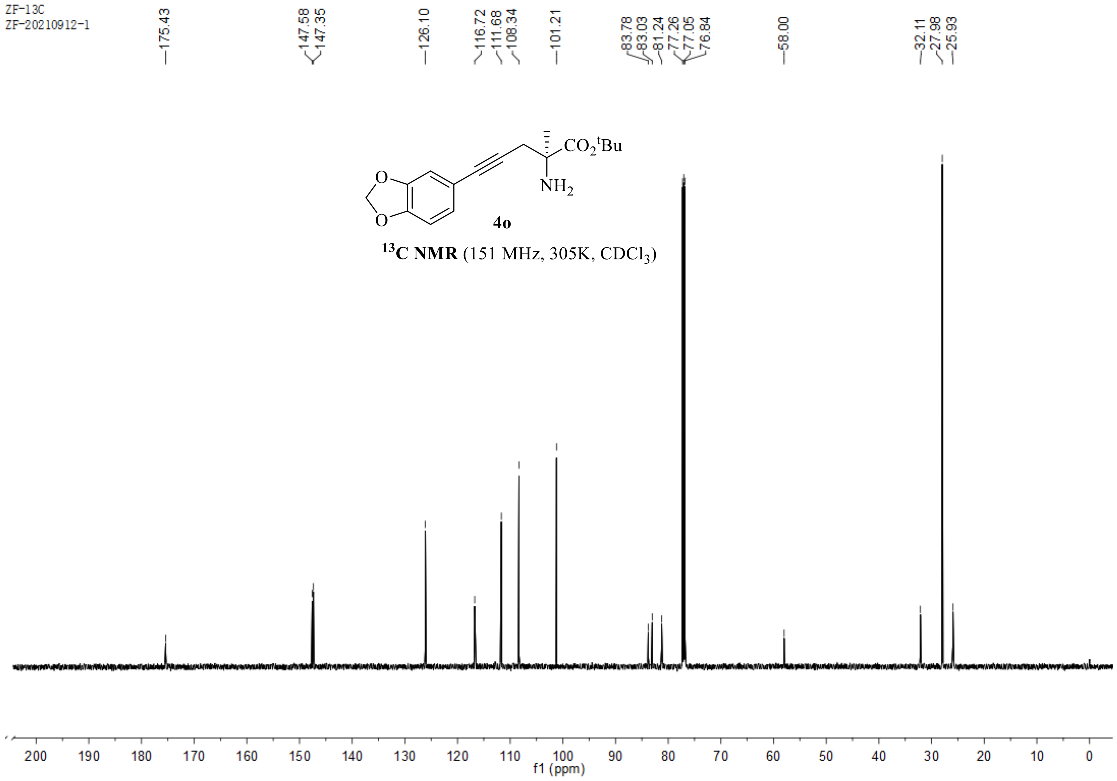

Supplementary Figure 18: NMR of compound 4o.

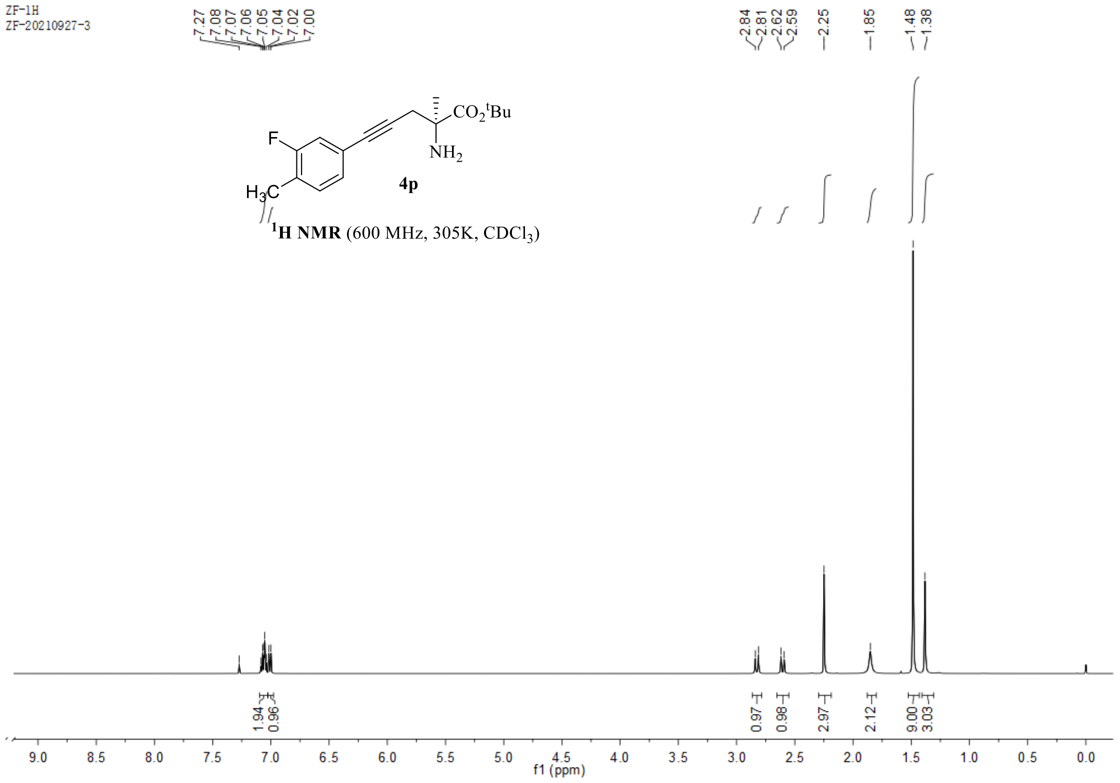
ZF-1H
ZF-20210927-3
7.27
7.08
7.07
7.06
7.05
7.04
7.02
7.00
2.84
2.81
2.62
2.59
2.25
1.85
1.48
1.38
$CO_2{}^tBu$
F
NH_2
H_3C
4p
^{1}H NMR (600 MHz, 305K, $CDCl_3$)
1.94
0.96
0.97
0.98
2.97
2.12
9.00
3.03
f1 (ppm)

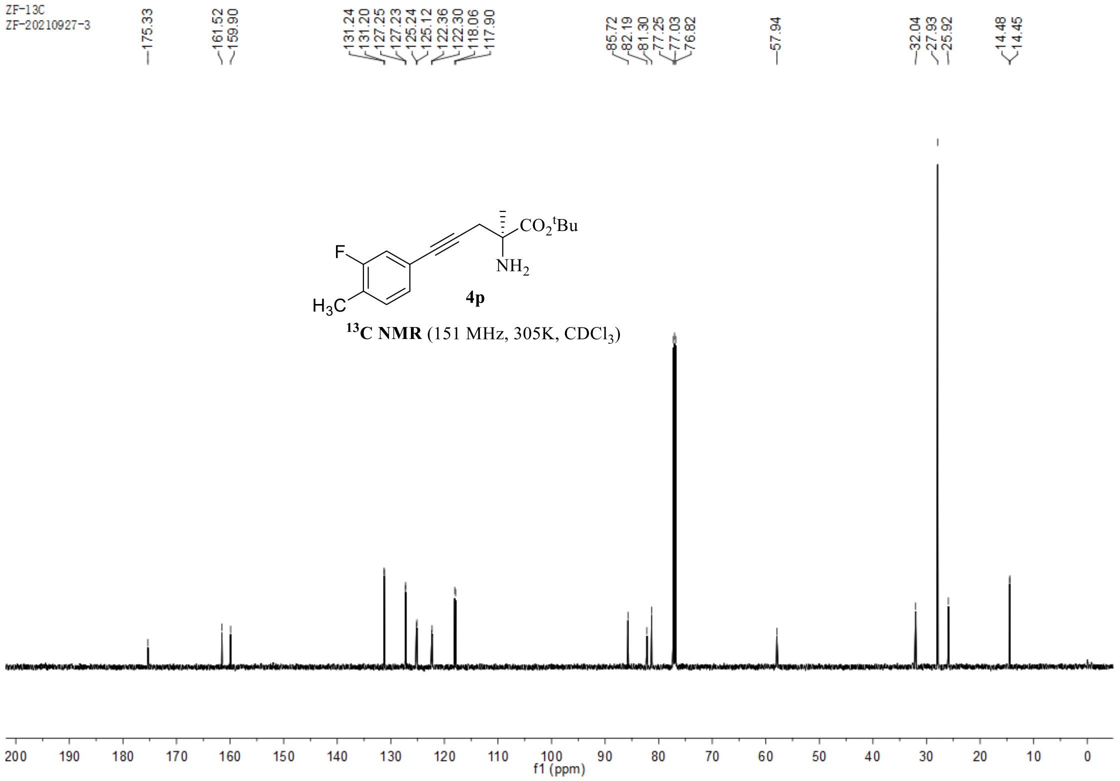
ZF-13C
ZF-20210927-3
175.33
161.52
159.90
131.24
131.20
127.25
127.23
125.24
125.12
122.36
122.30
118.06
117.90
85.72
82.19
81.30
77.25
77.03
76.82
57.94
32.04
27.93
25.92
14.48
14.45
$CO_2{}^tBu$
F
NH_2
H_3C
4p
^{13}C NMR (151 MHz, 305K, $CDCl_3$)
f1 (ppm)

ZF-19F
ZF-20210927-3

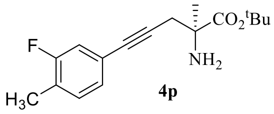

4p

^{19}F NMR (565 MHz, 305K, $CDCl_3$)

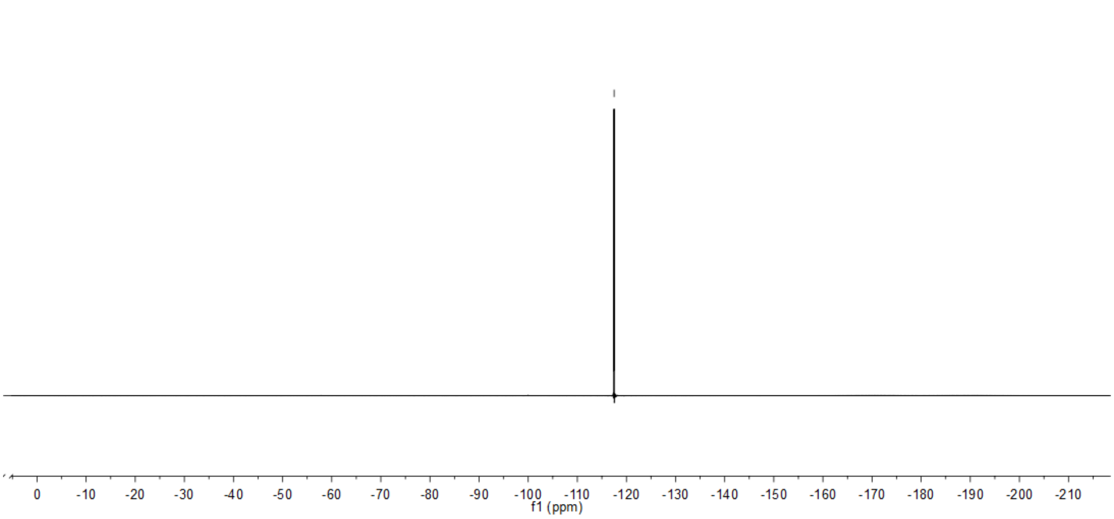

Supplementary Figure 19: NMR of compound 4p.

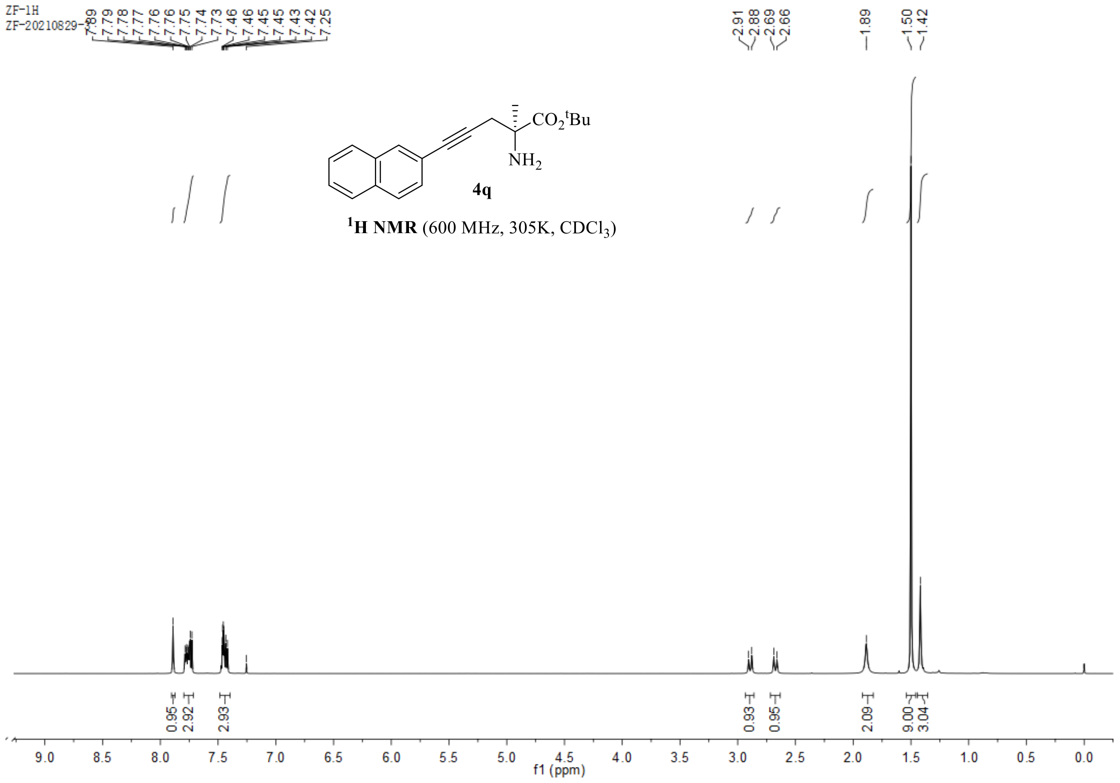

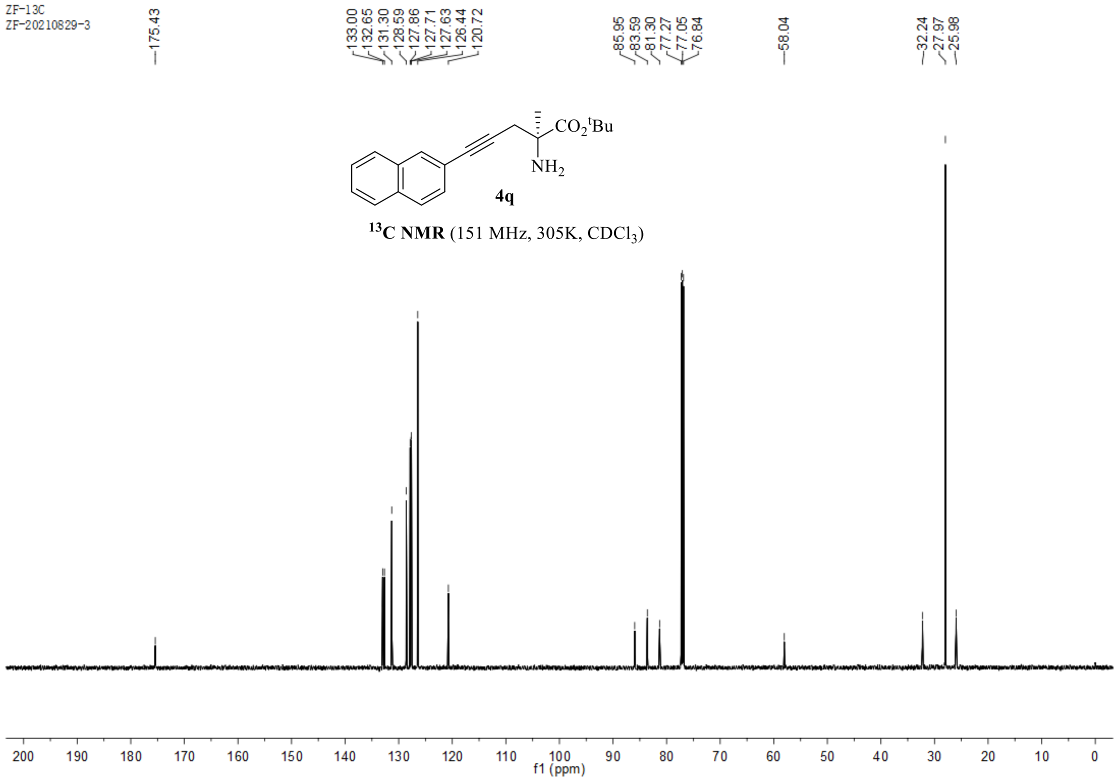

Supplementary Figure 20: NMR of compound 4q.

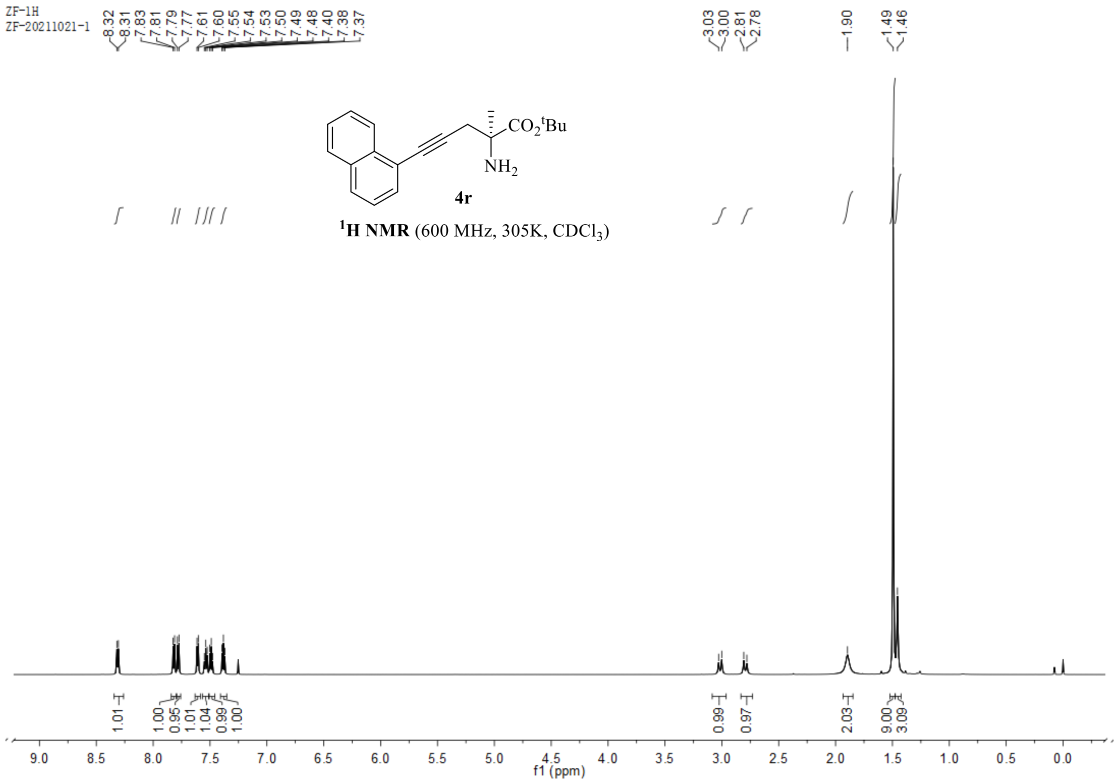

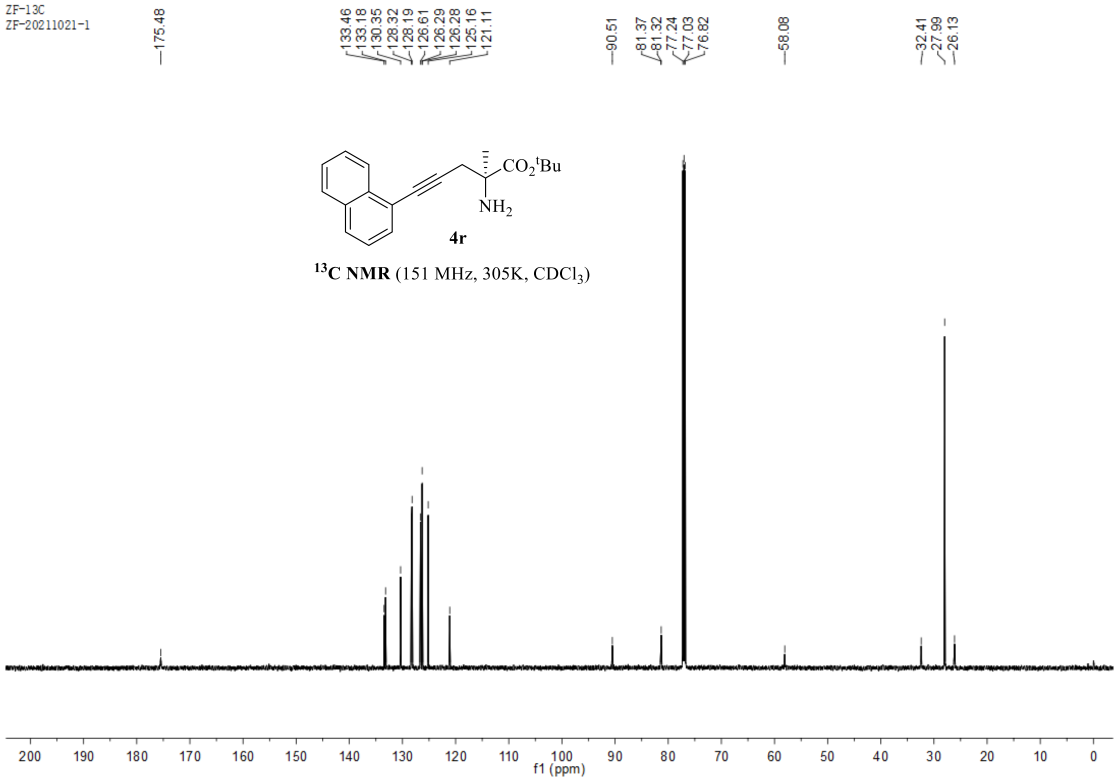

Supplementary Figure 21: NMR of compound 4r.

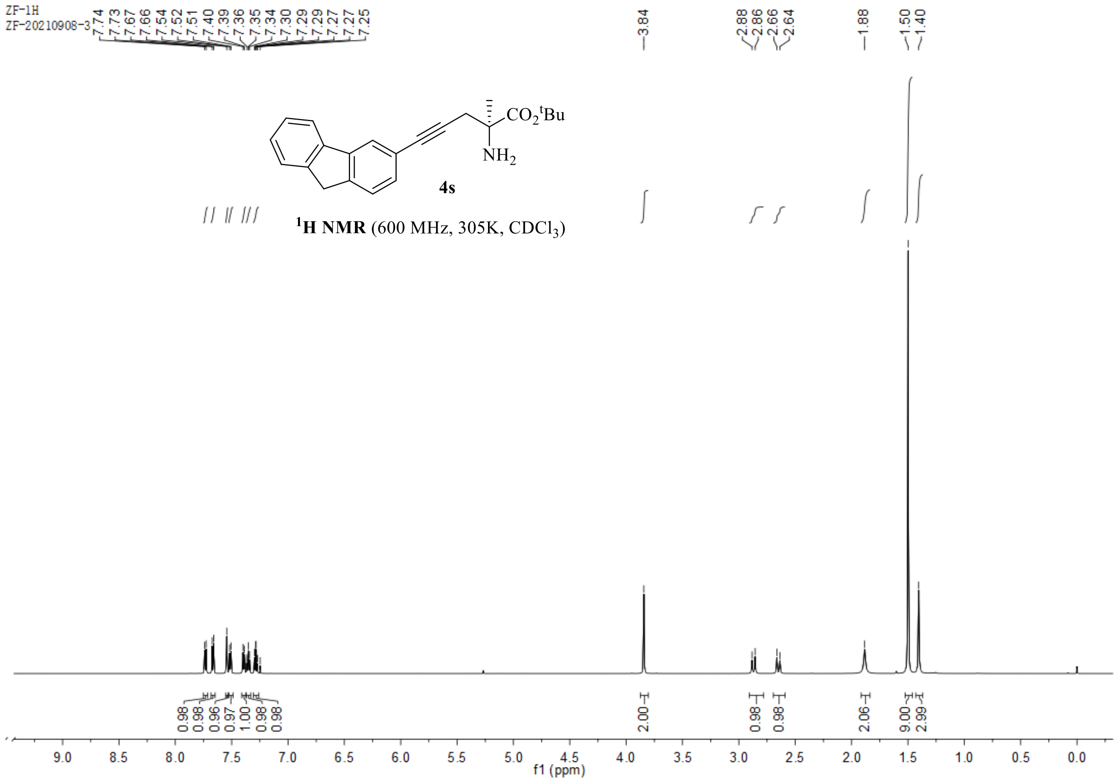

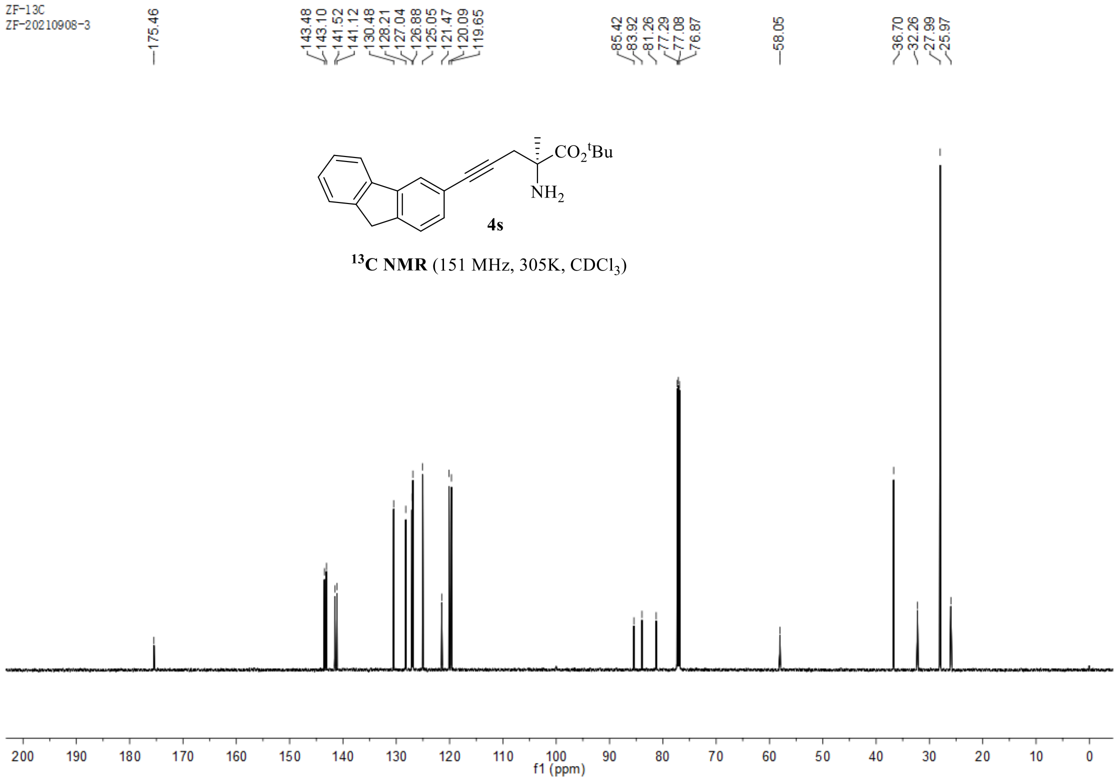

Supplementary Figure 22: NMR of compound 4s.

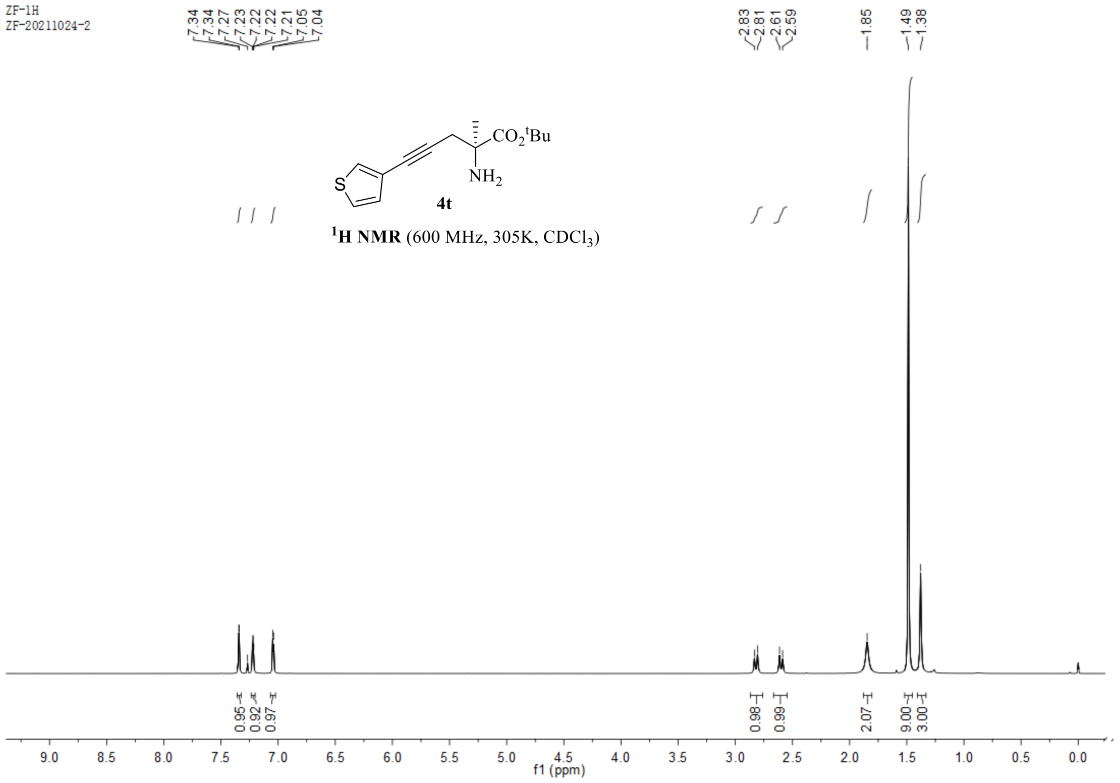

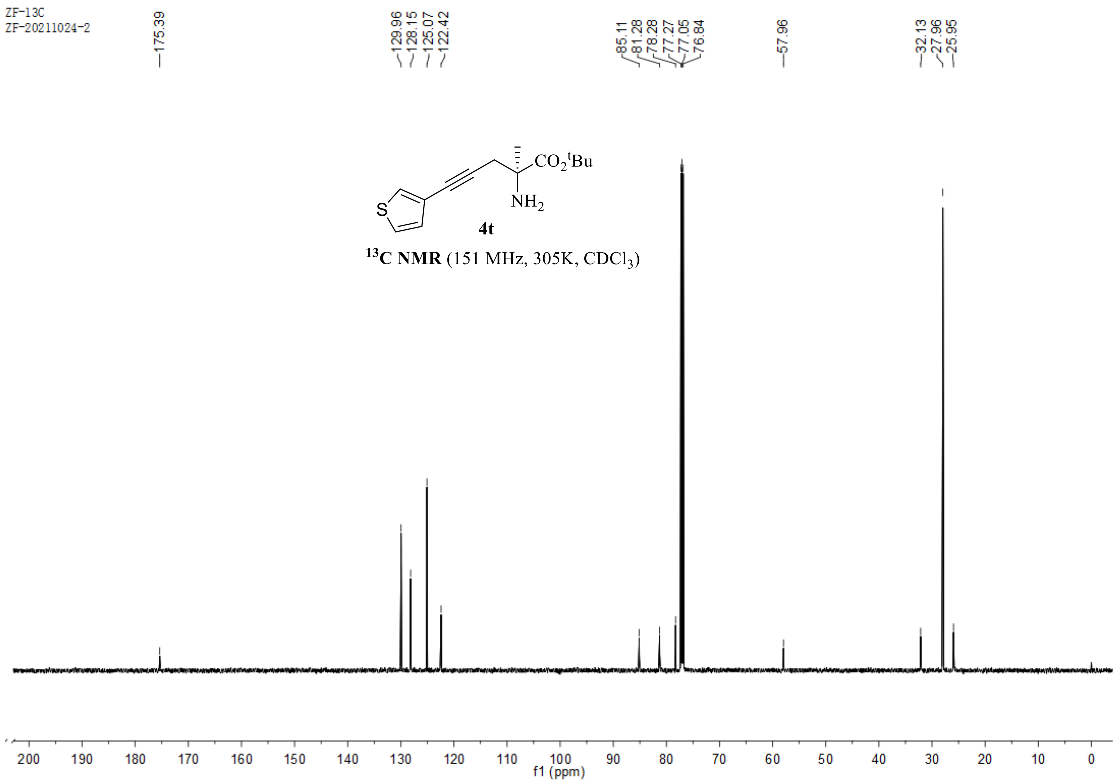

Supplementary Figure 23: NMR of compound 4t.

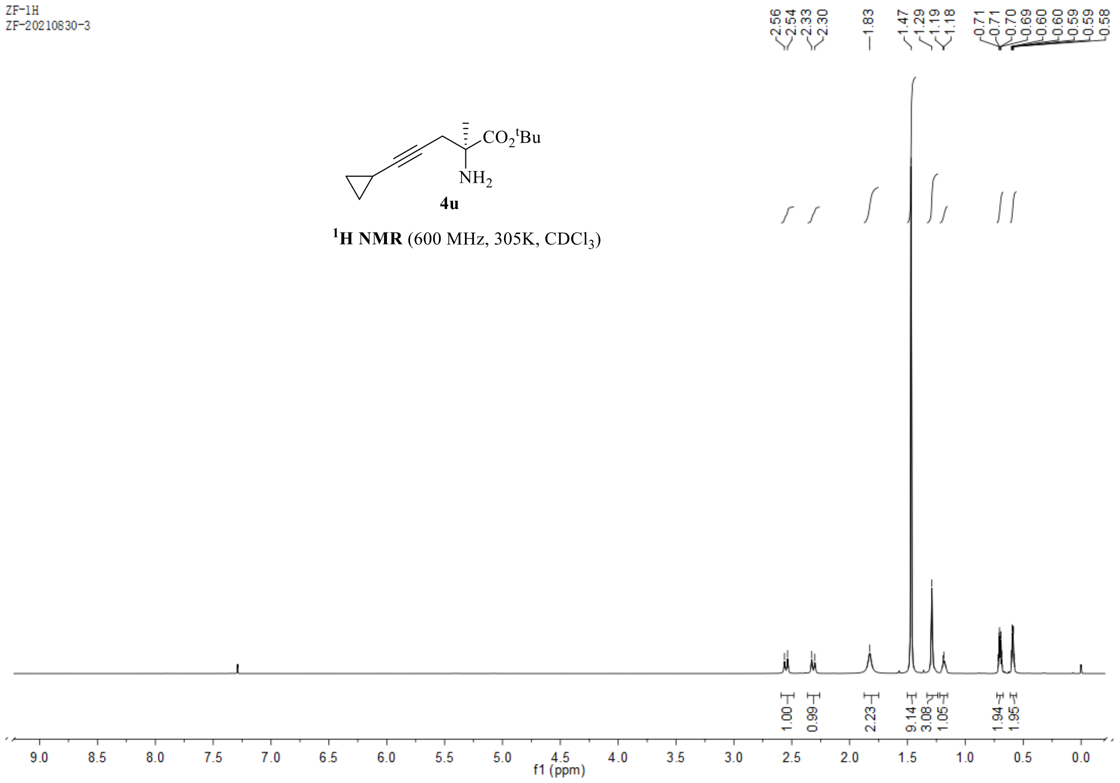

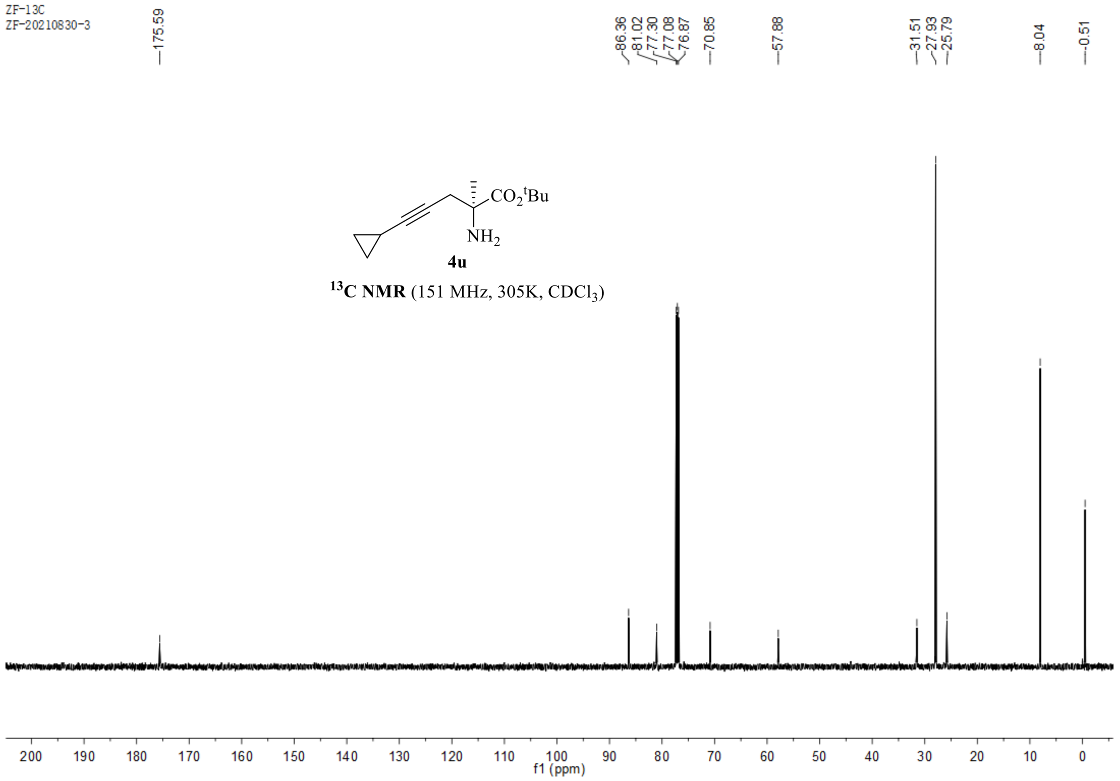

Supplementary Figure 24: NMR of compound 4u.

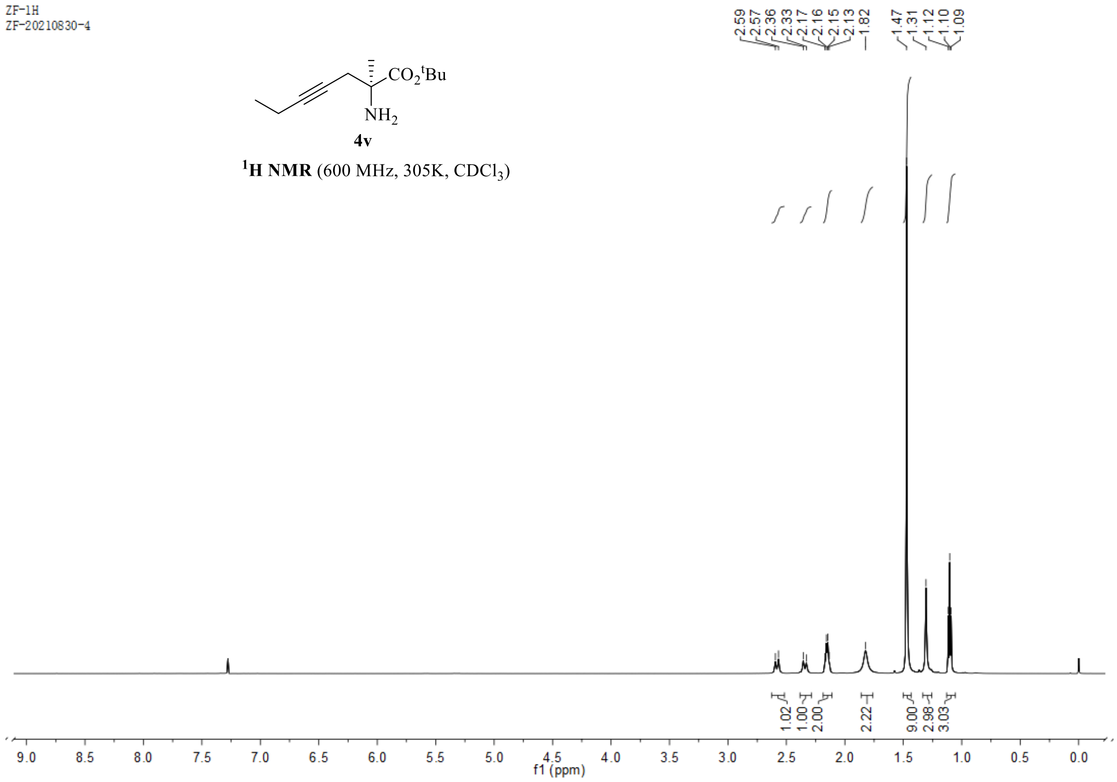

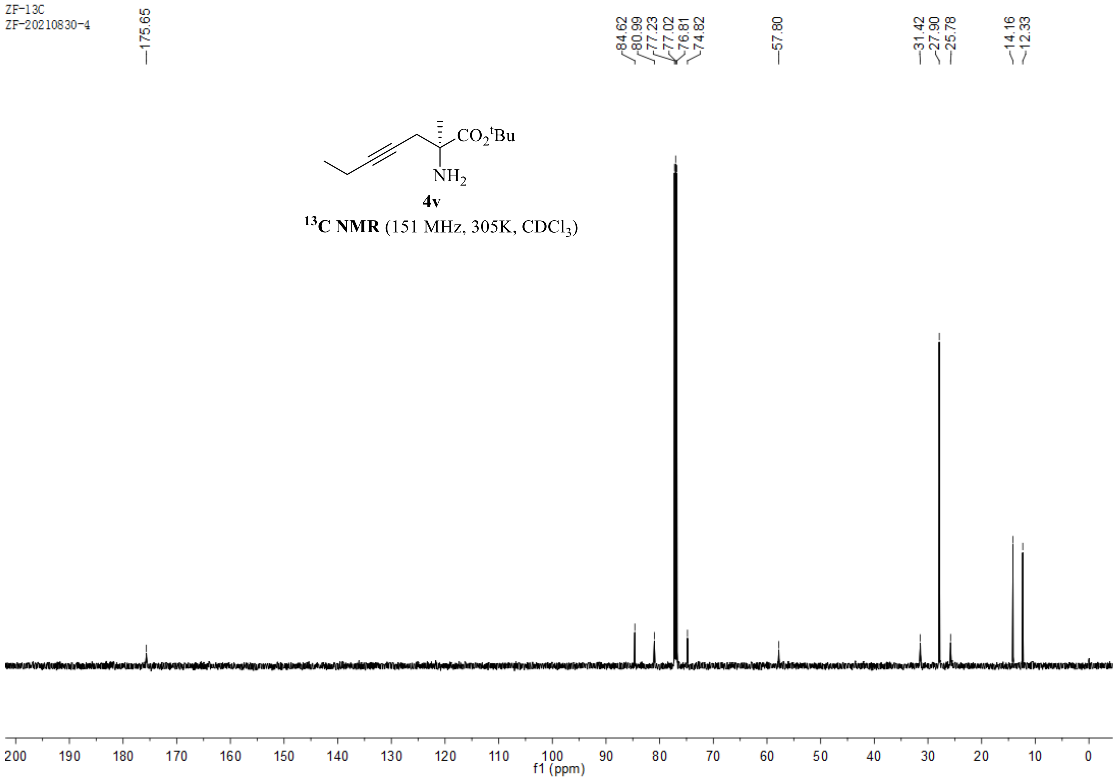

Supplementary Figure 25: NMR of compound 4v.

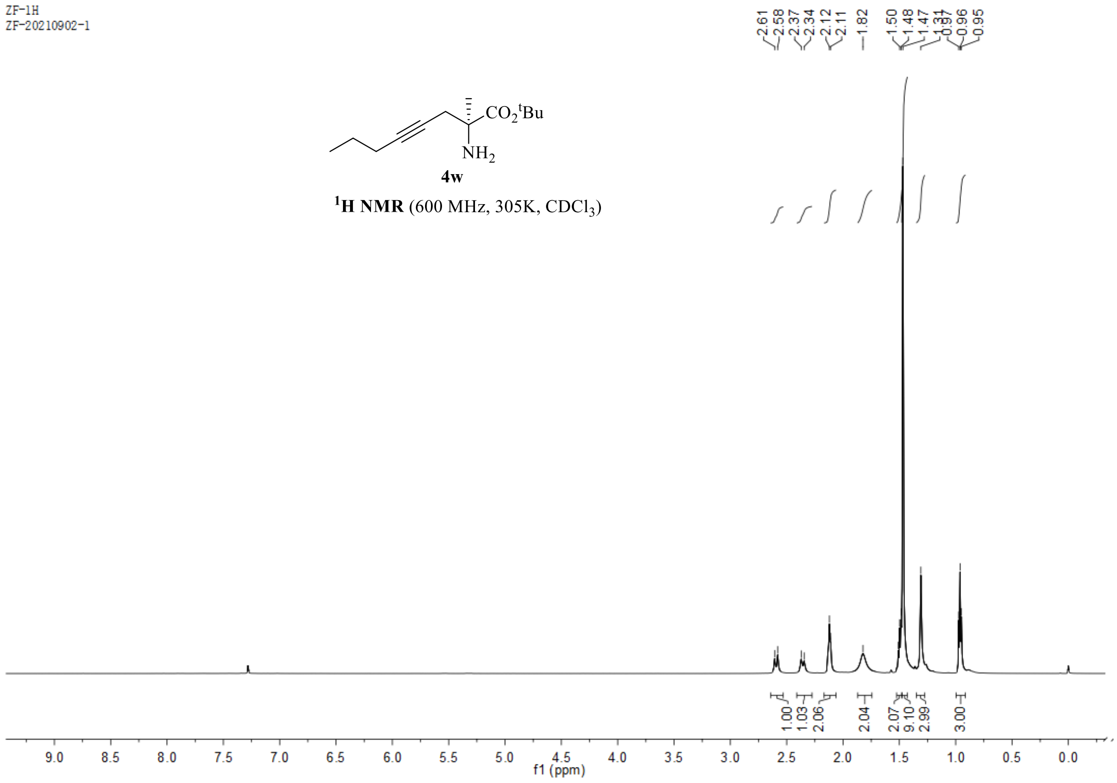

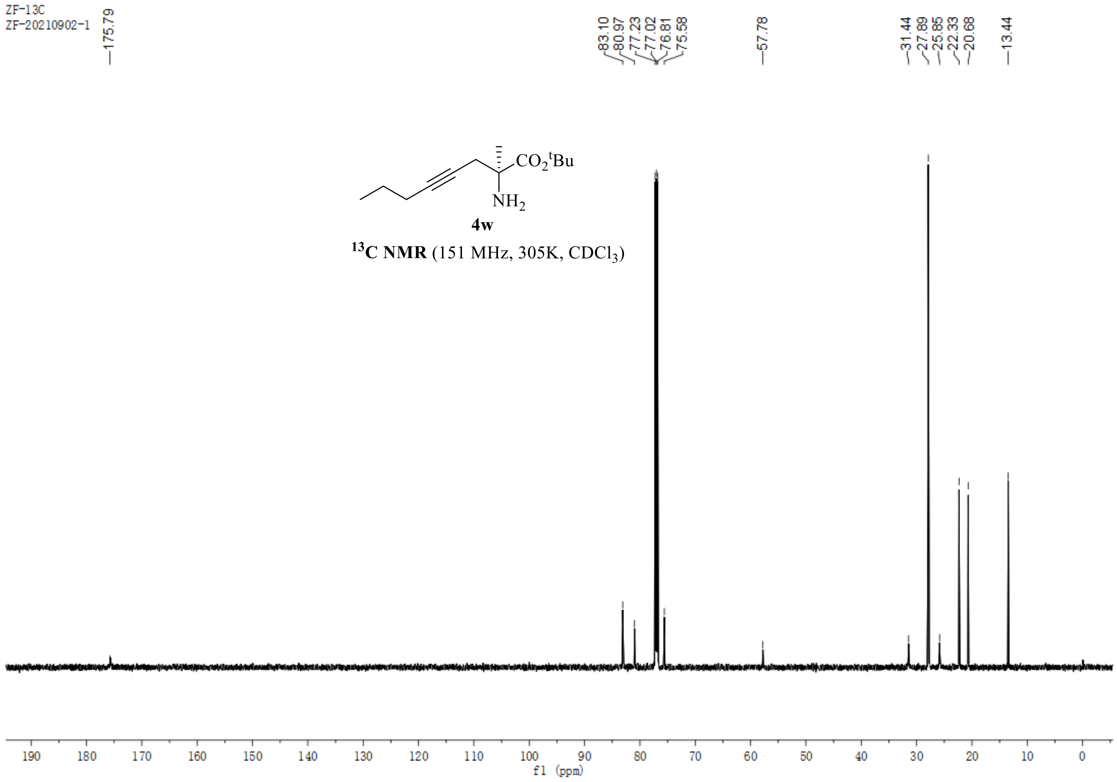

Supplementary Figure 26: NMR of compound 4w.

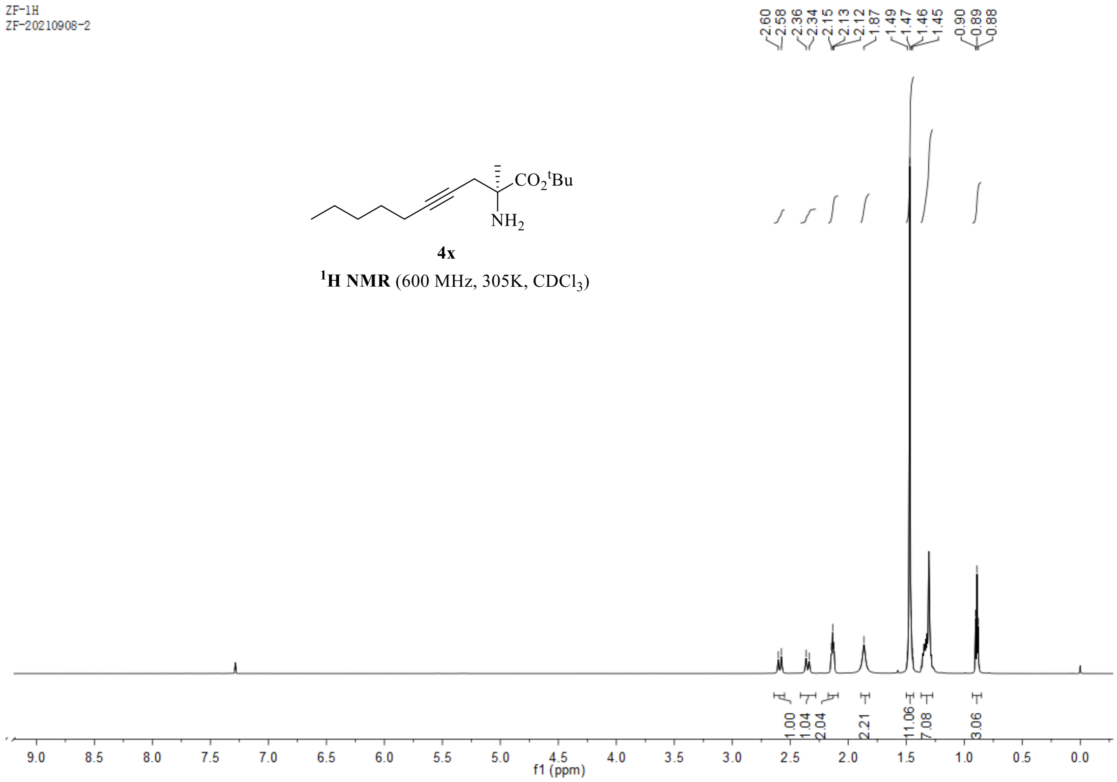

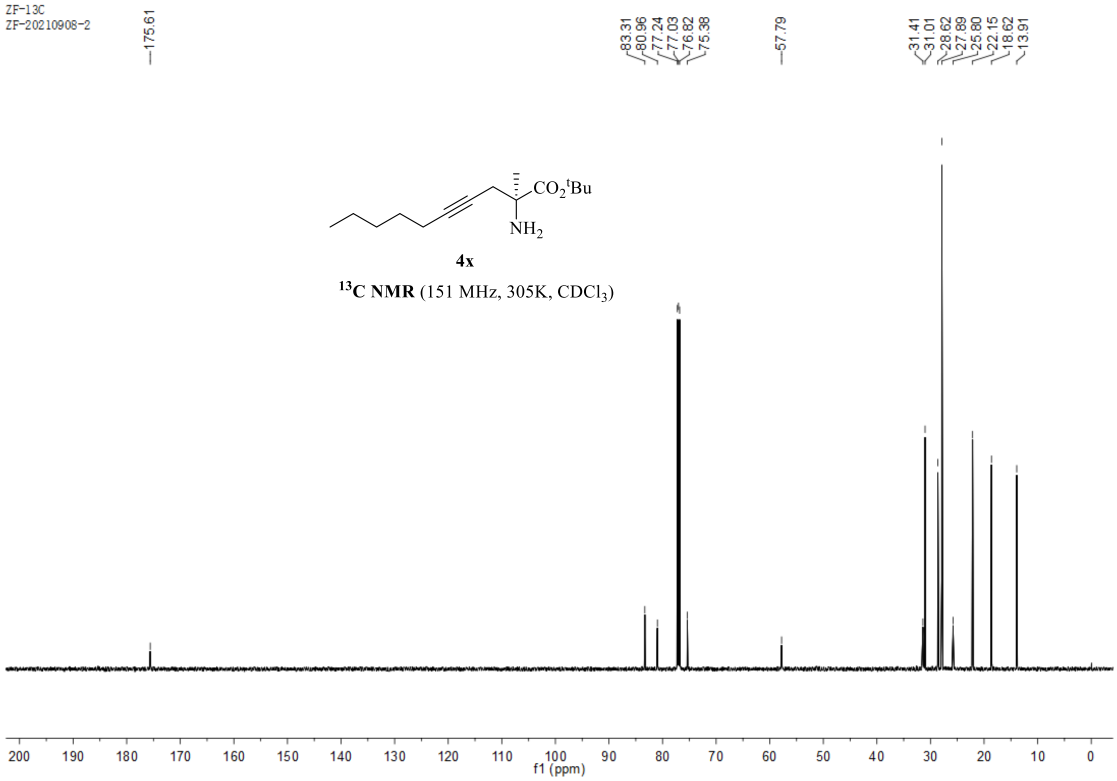

Supplementary Figure 27: NMR of compound 4x.

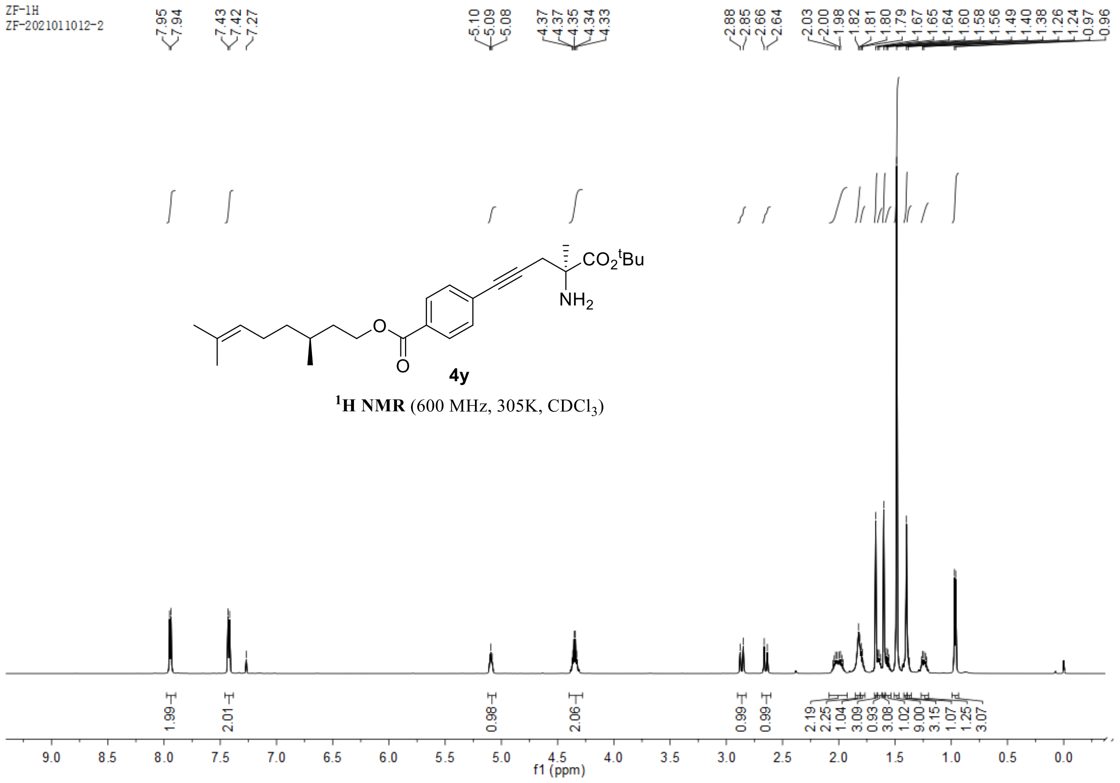

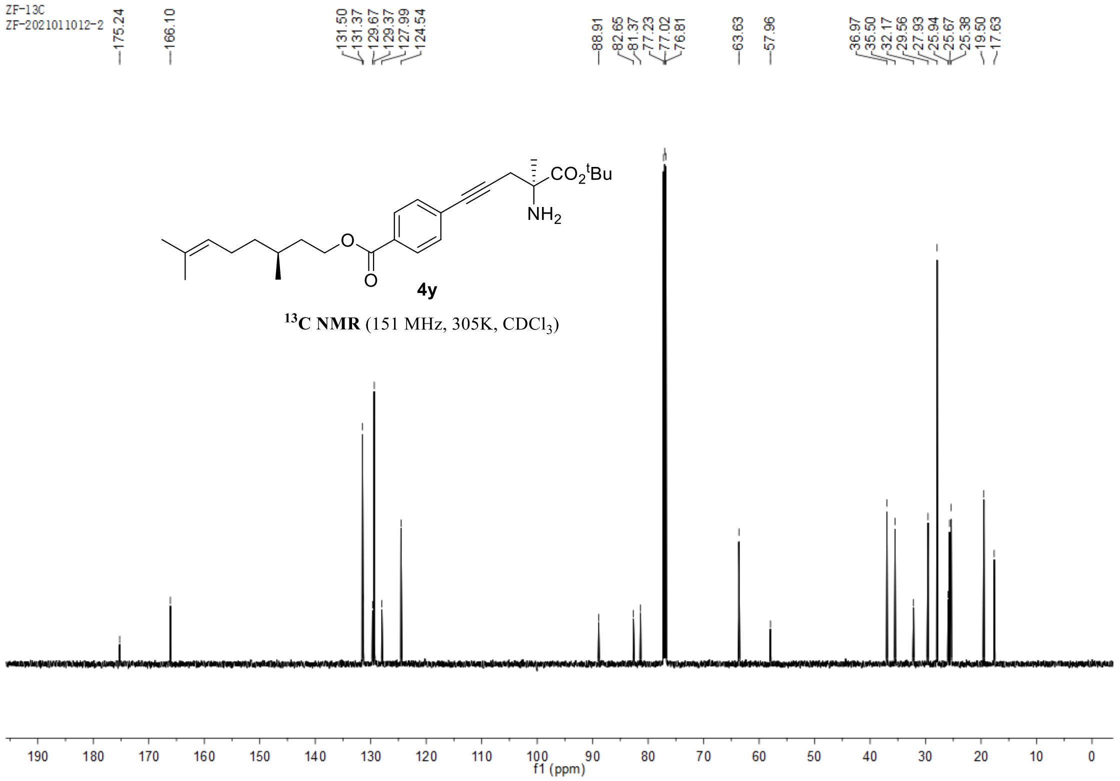

Supplementary Figure 28: NMR of compound 4y.

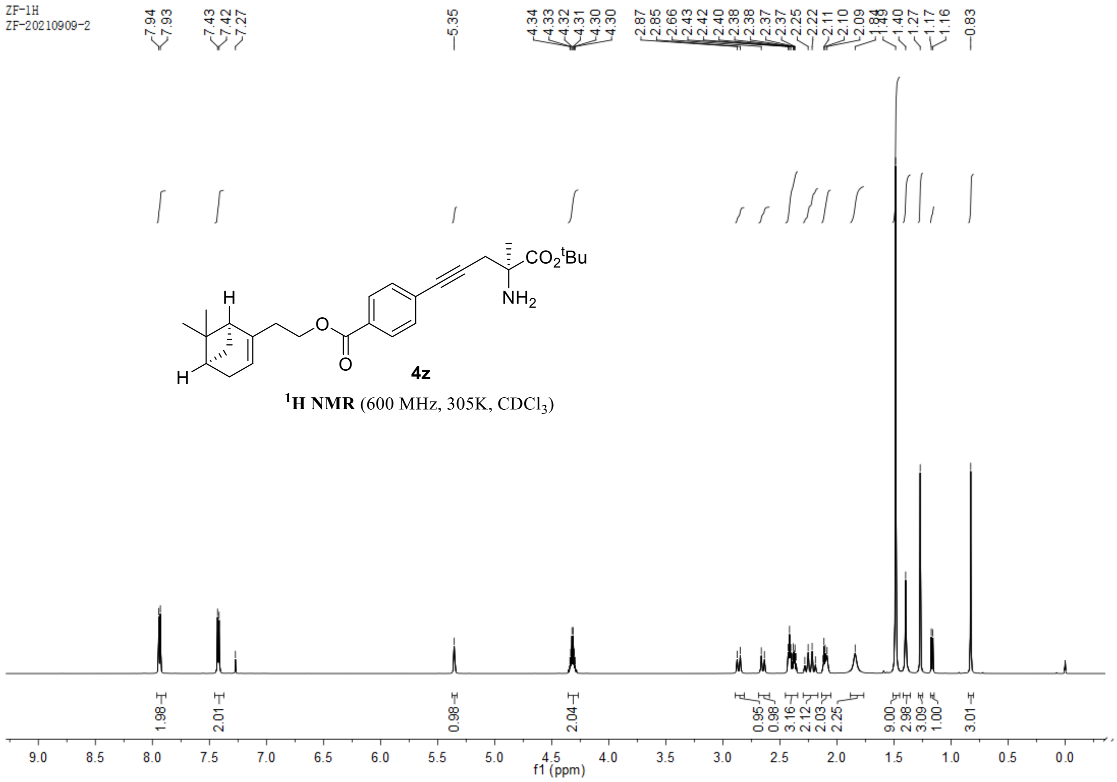

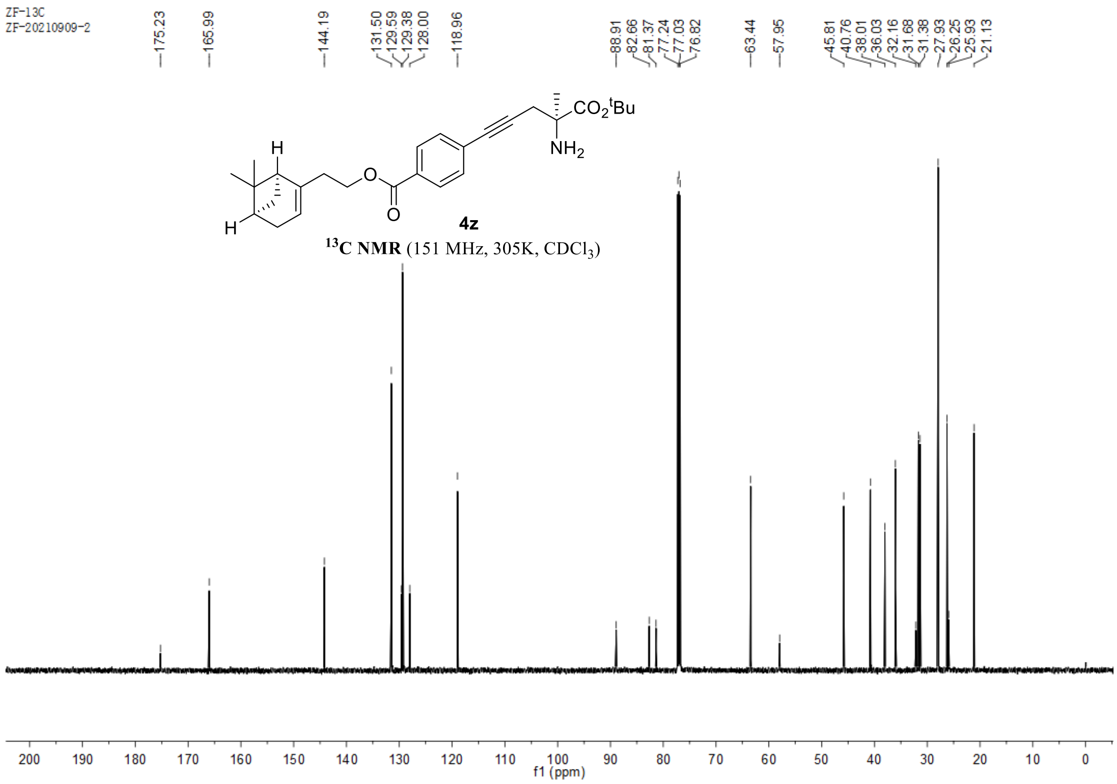

Supplementary Figure 29: NMR of compound 4z.

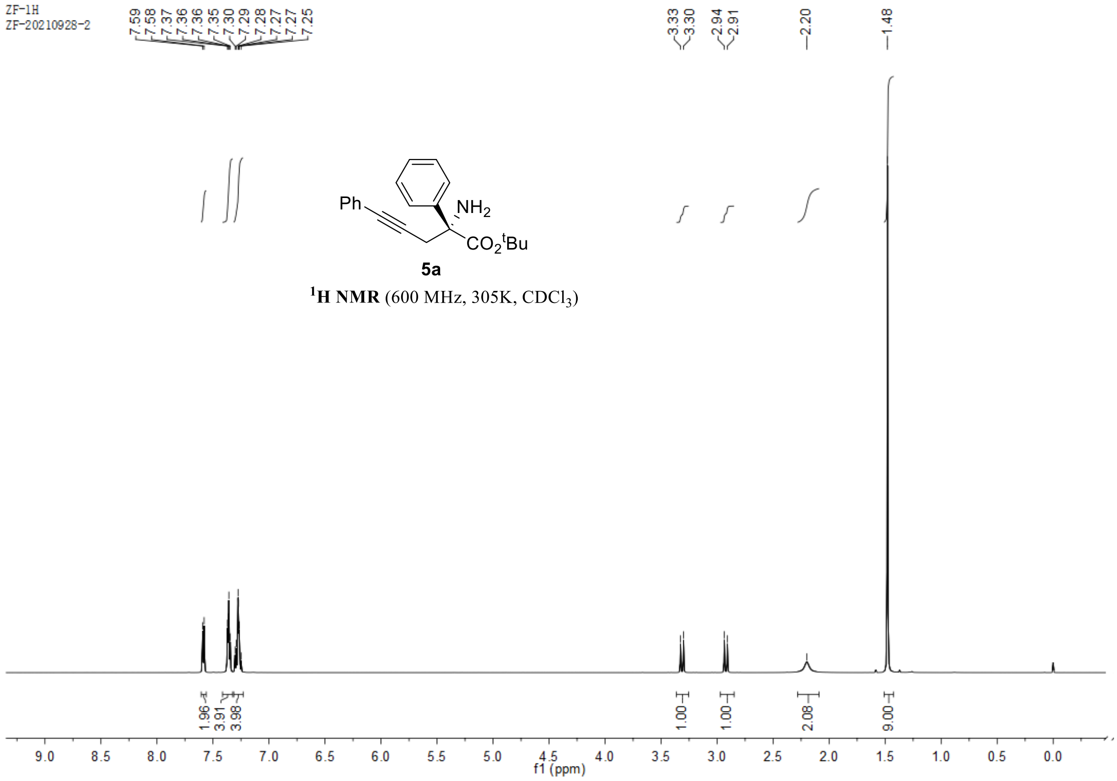

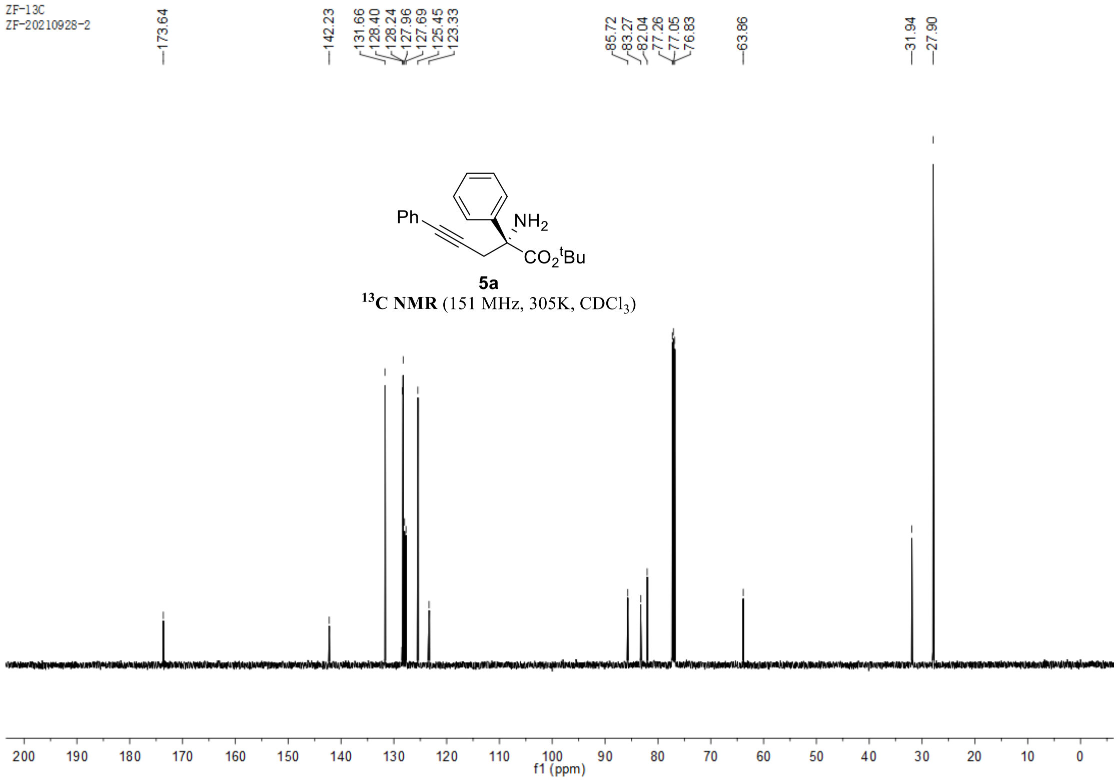

Supplementary Figure 30: NMR of compound 5a.

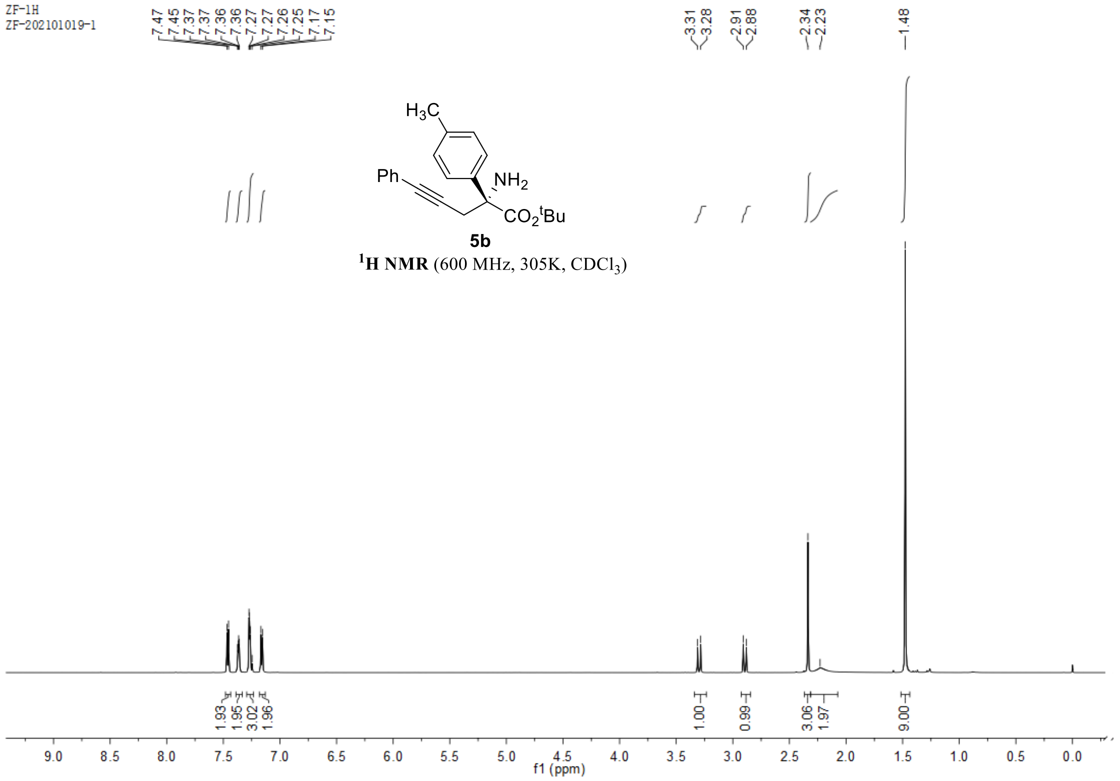

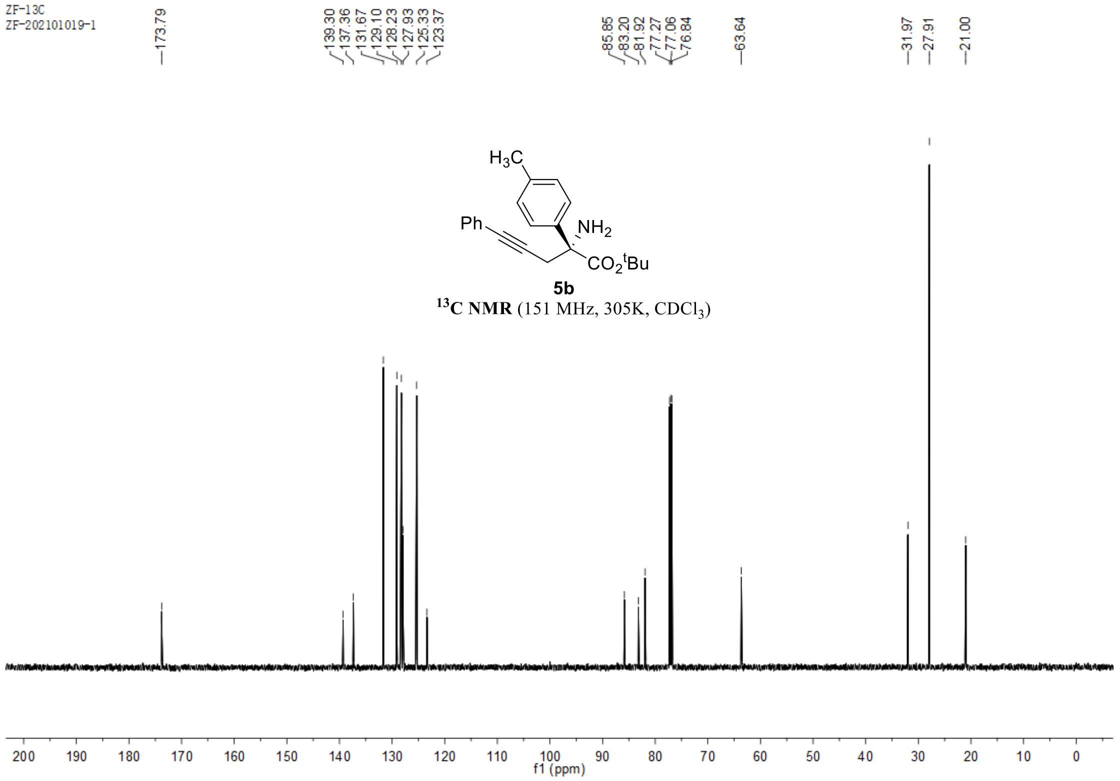

Supplementary Figure 31: NMR of compound 5b.

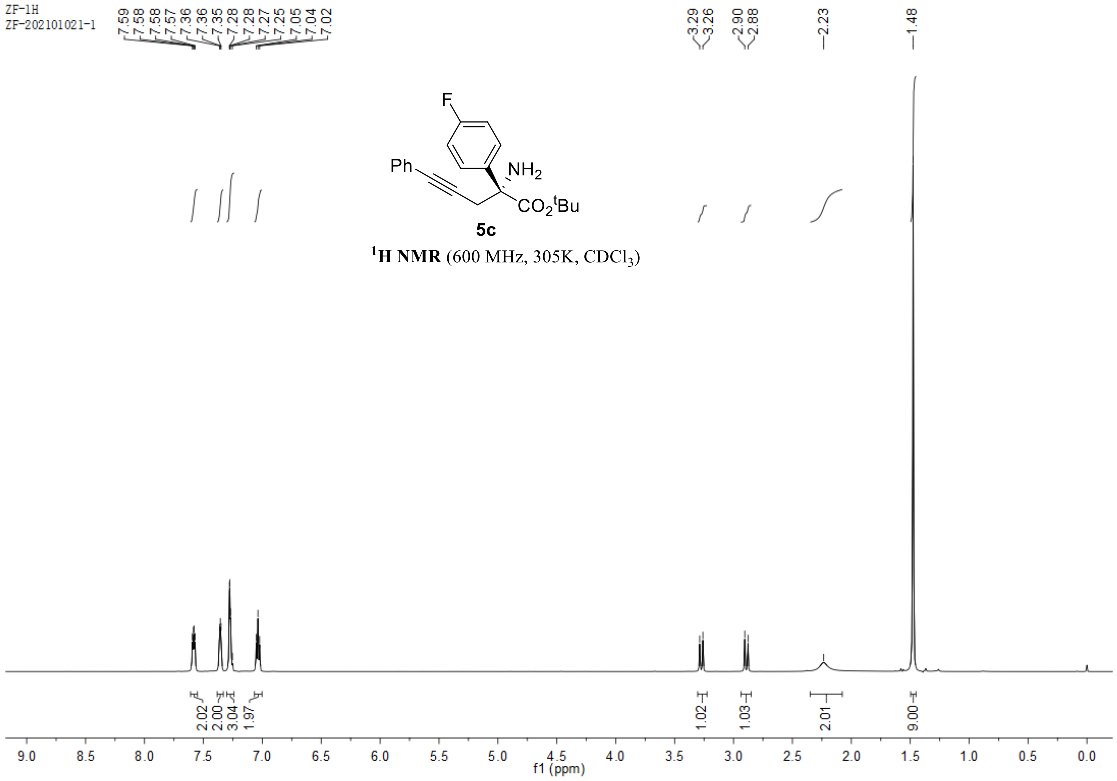

5c

^{1}H NMR (600 MHz, 305K, $CDCl_3$)

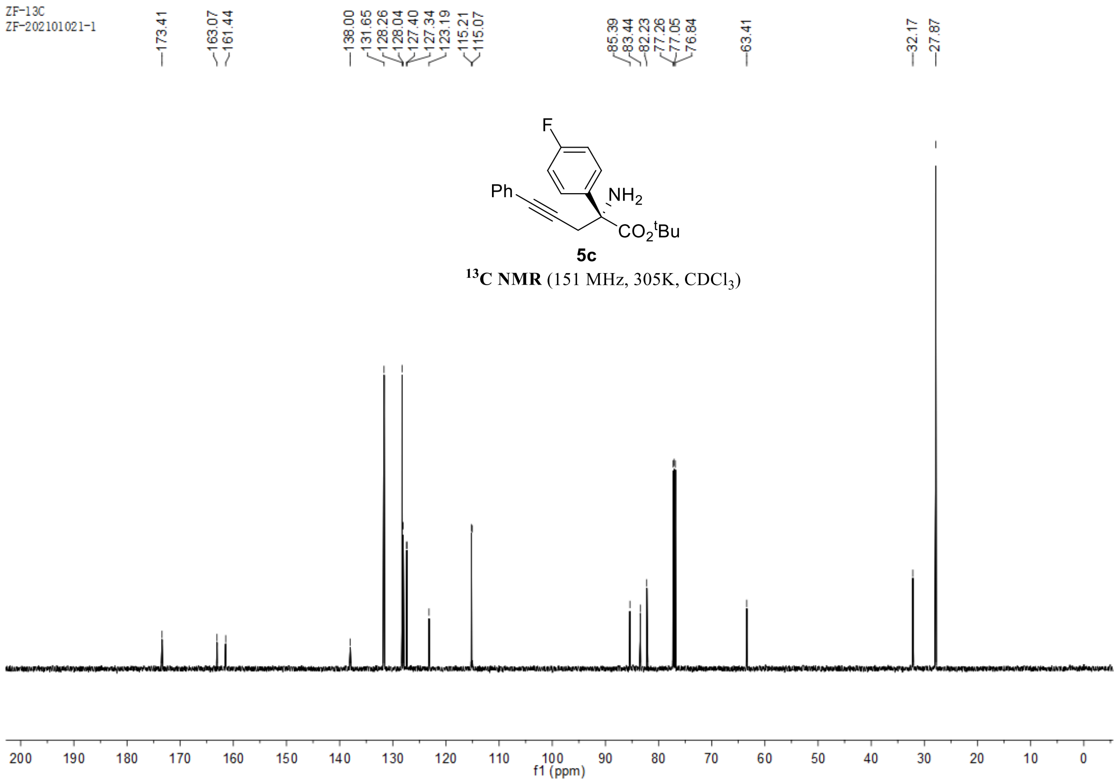

5c

^{13}C NMR (151 MHz, 305K, $CDCl_3$)

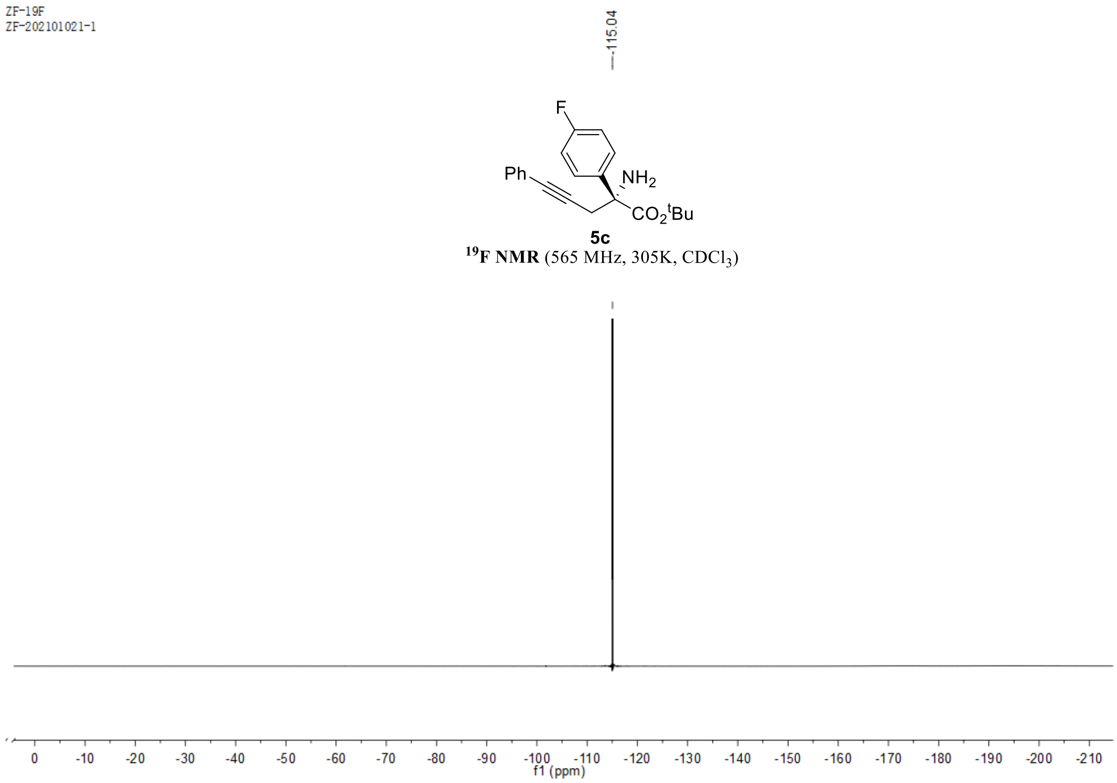

Supplementary Figure 32: NMR of compound 5c.

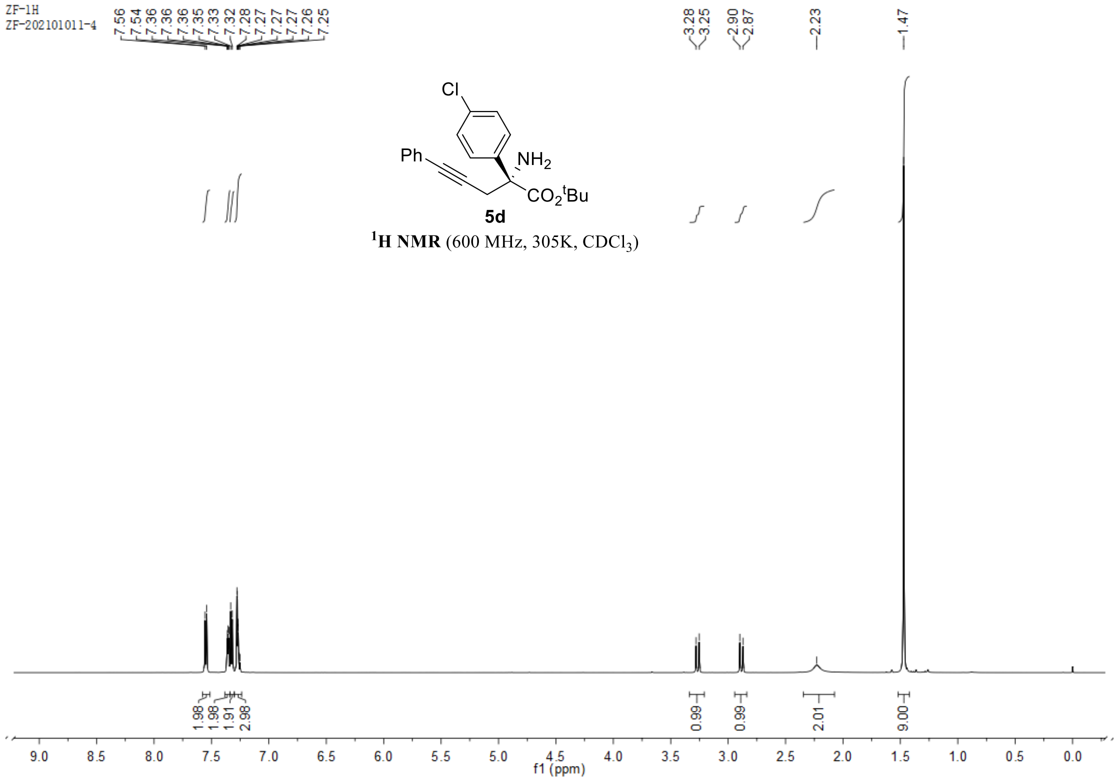

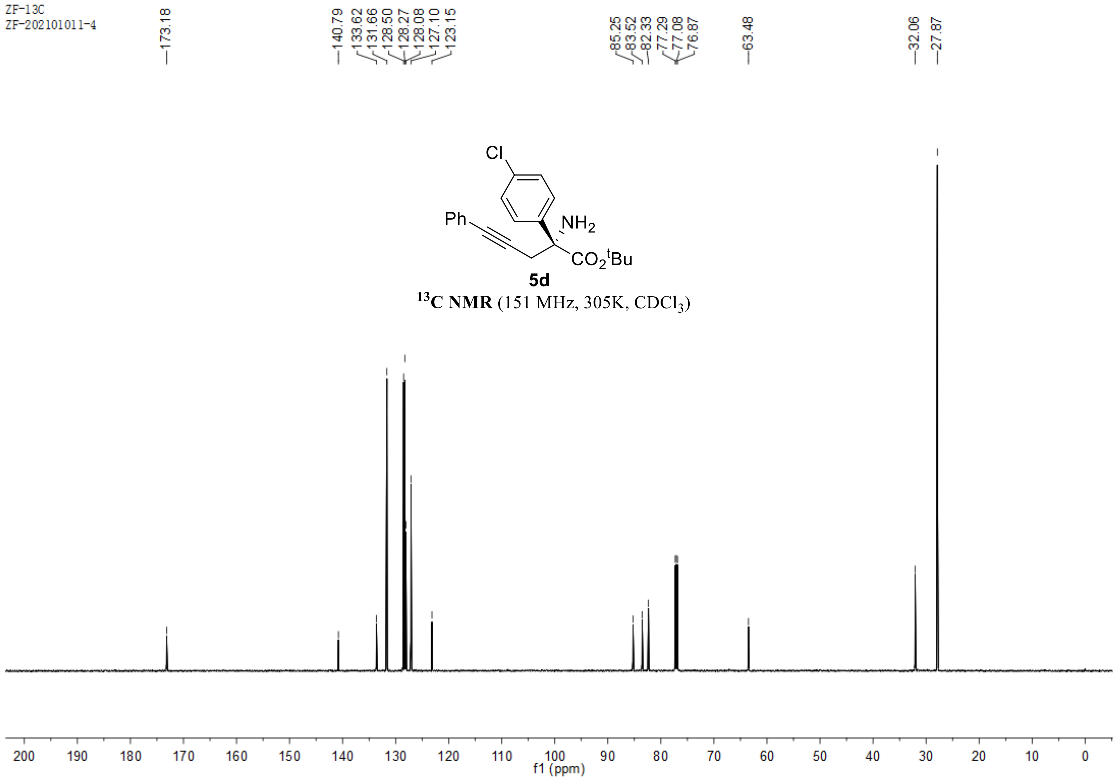

Supplementary Figure 33: NMR of compound 5d.

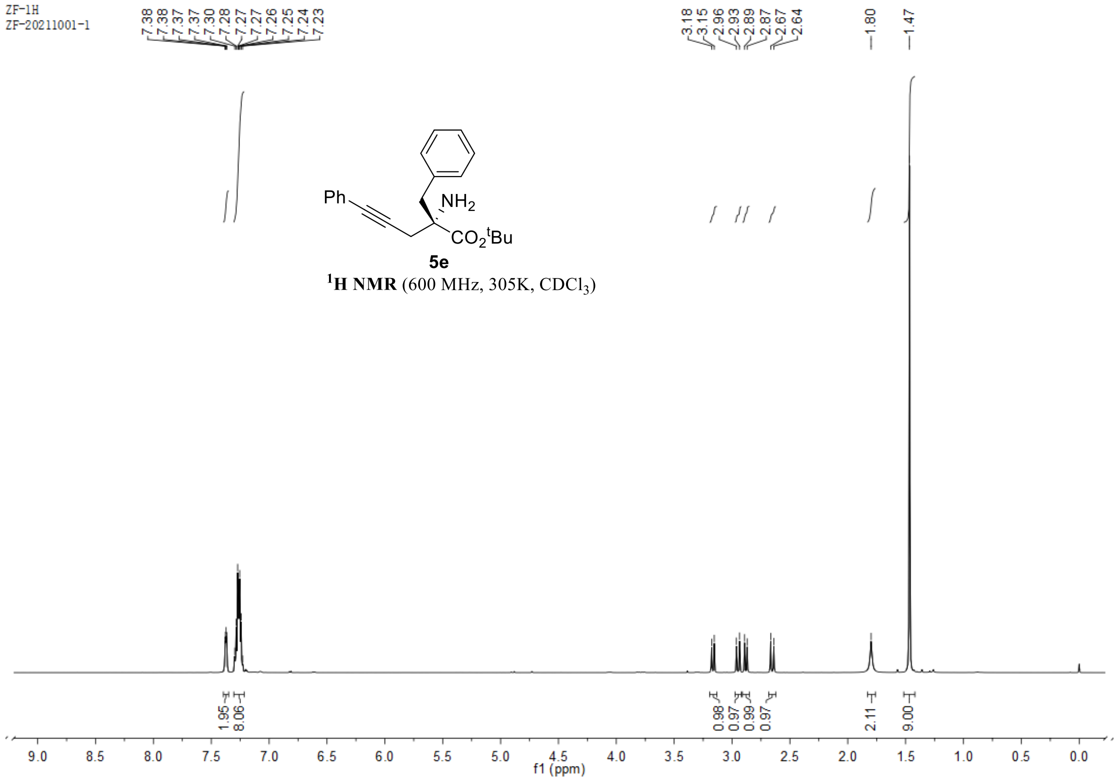

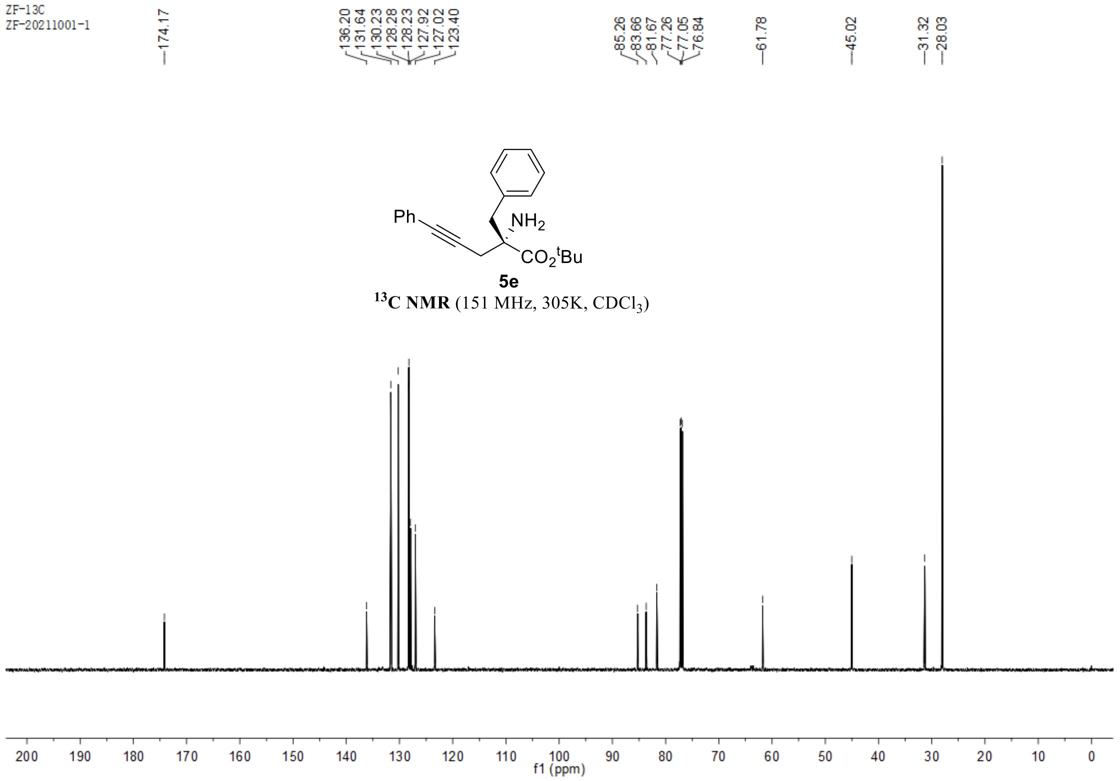

Supplementary Figure 34: NMR of compound 5e.

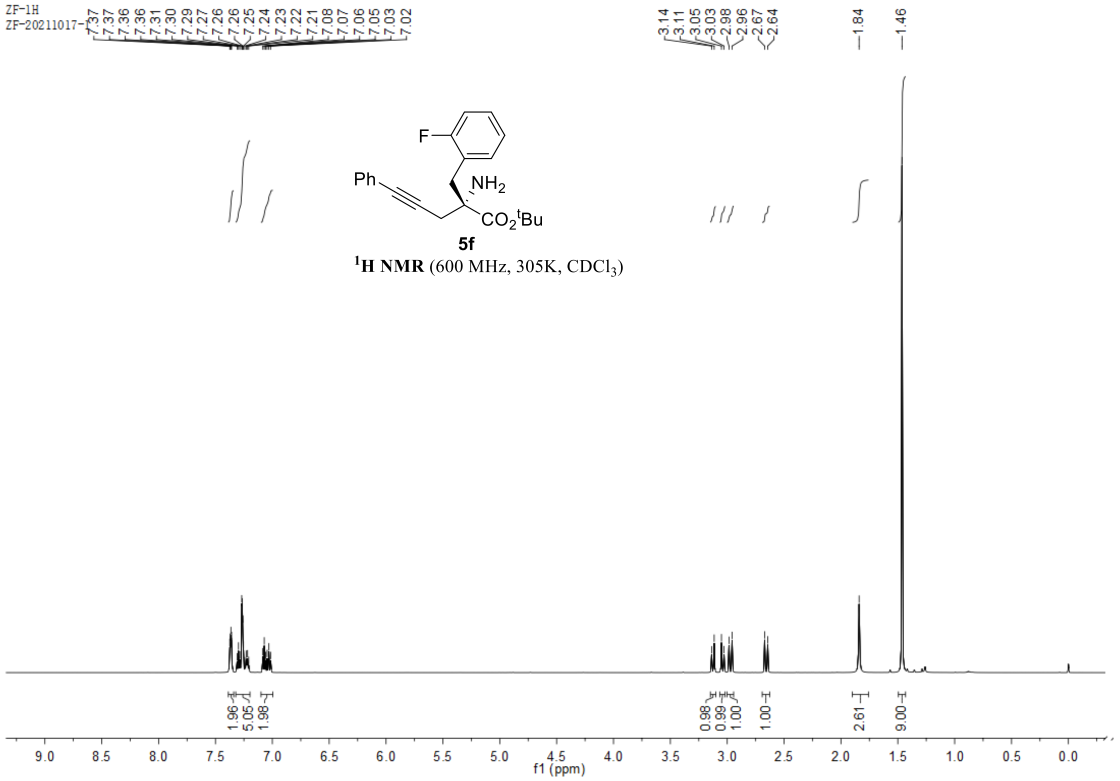
ZF-1H
ZF-20211017-1
7.37 7.37 7.36 7.31 7.30 7.29 7.27 7.26 7.25 7.24 7.23 7.22 7.21 7.08 7.07 7.06 7.05 7.03 7.02
3.14 3.11 3.05 3.03 2.98 2.96 2.67 2.64
1.84
1.46
F
Ph
NH2
CO2tBu
5f
1H NMR (600 MHz, 305K, CDCl3)
1.96 5.05 1.98
0.98 0.99 1.00 1.00
2.61
9.00
9.0 8.5 8.0 7.5 7.0 6.5 6.0 5.5 5.0 4.5 4.0 3.5 3.0 2.5 2.0 1.5 1.0 0.5 0.0
f1 (ppm)

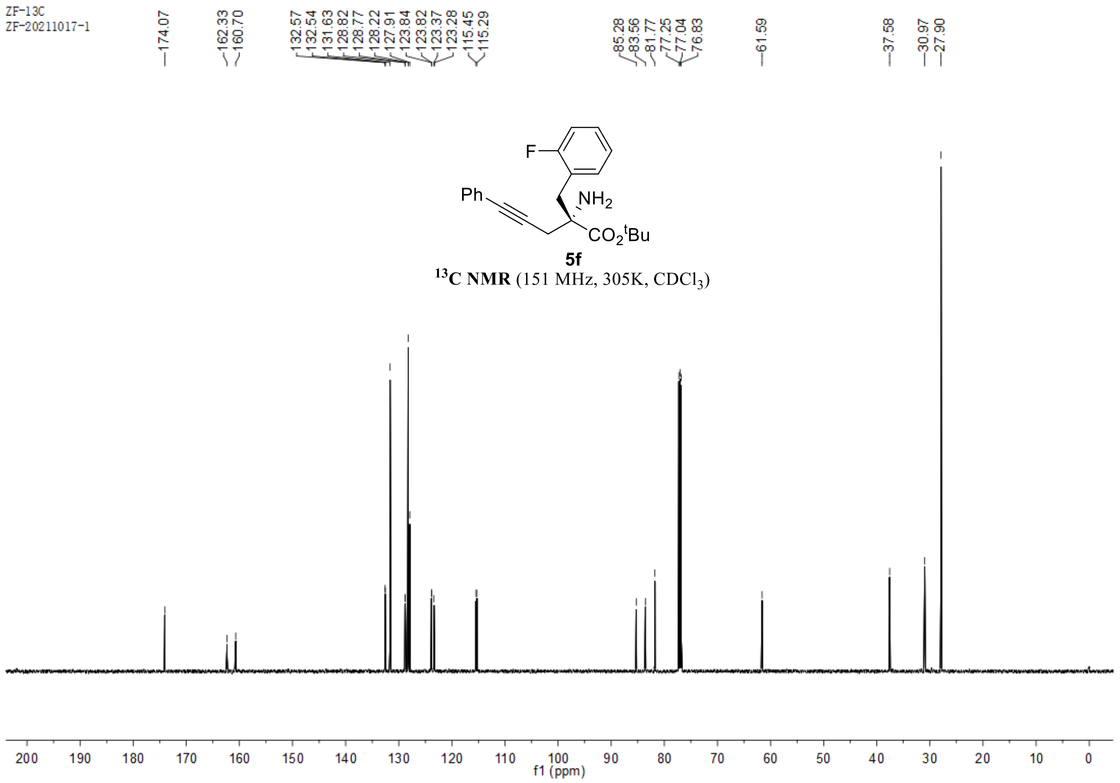
ZF-13C
ZF-20211017-1
174.07
162.33 160.70
132.57 132.54 131.63 128.82 128.77 128.22 127.91 123.84 123.82 123.37 123.28 115.45 115.29
85.28 83.56 81.77 77.25 77.04 76.83
61.59
37.58
30.97 27.90
F
Ph
NH2
CO2tBu
5f
13C NMR (151 MHz, 305K, CDCl3)
200 190 180 170 160 150 140 130 120 110 100 90 80 70 60 50 40 30 20 10 0
f1 (ppm)

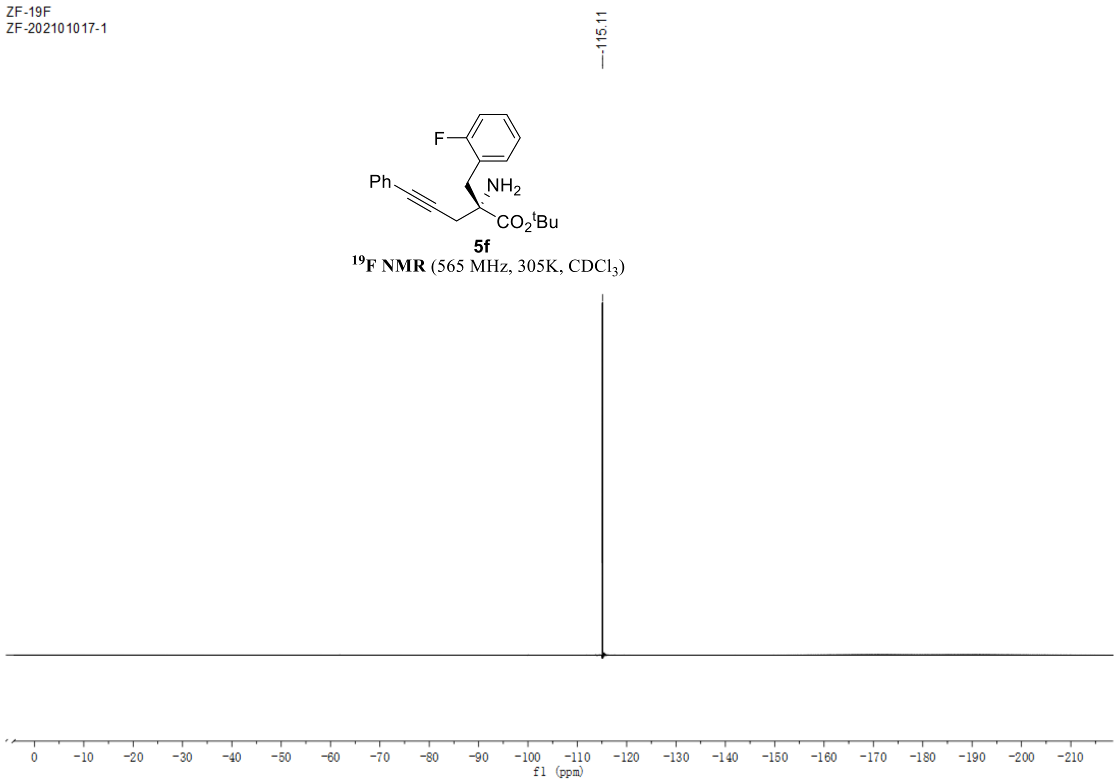

Supplementary Figure 35: NMR of compound 5f.

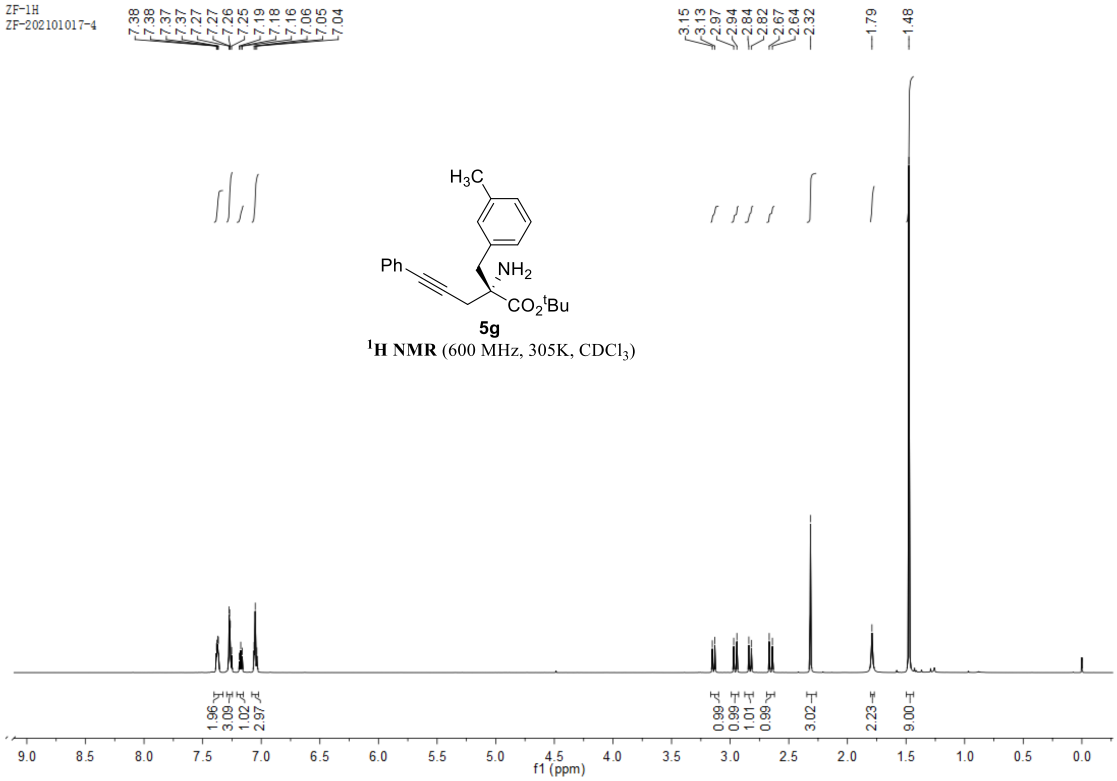

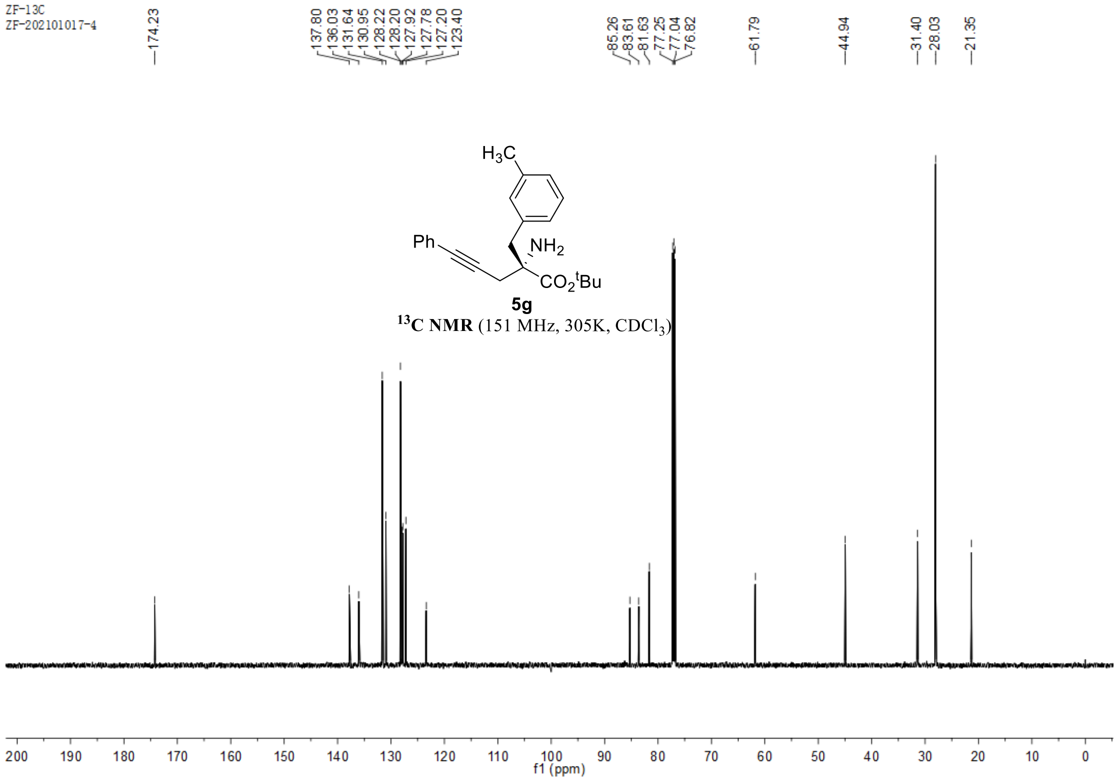

Supplementary Figure 36: NMR of compound 5g.

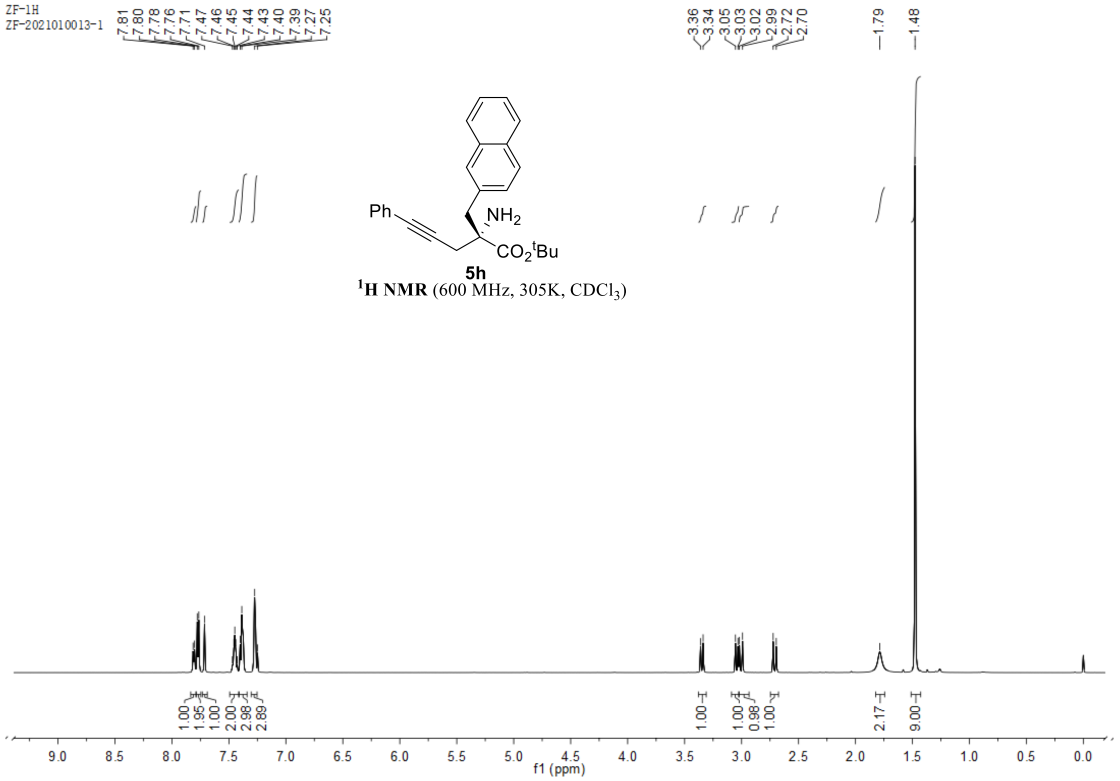

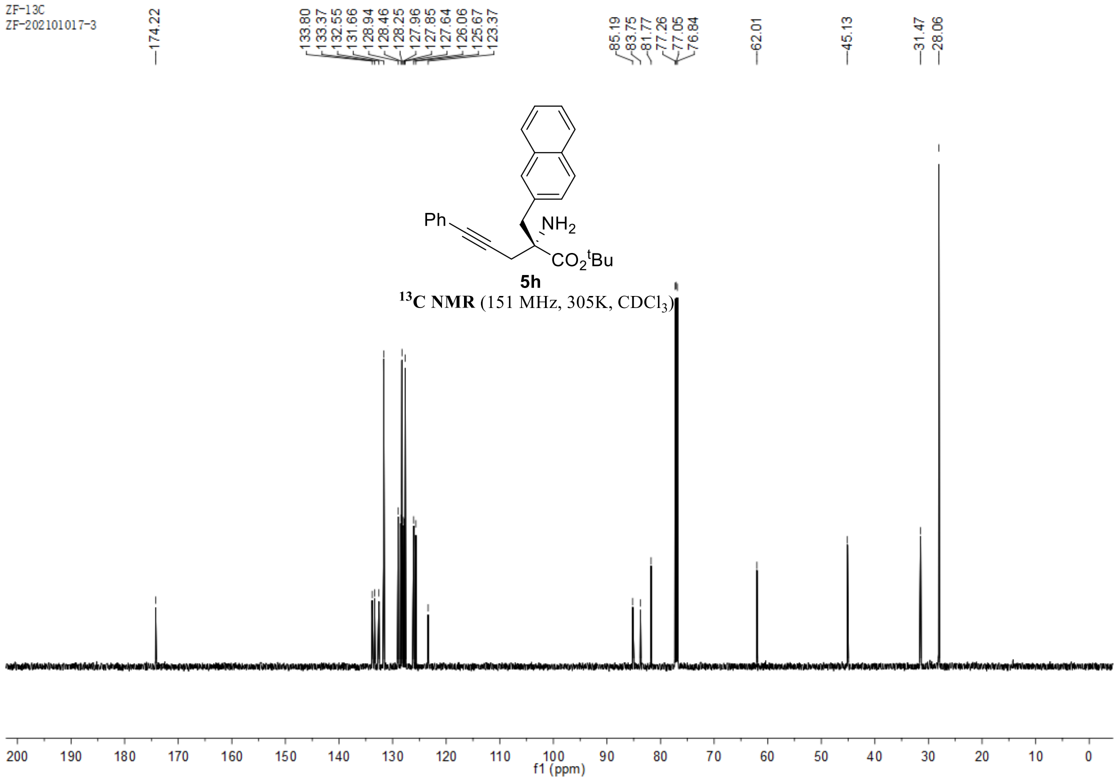

Supplementary Figure 37: NMR of compound 5h.

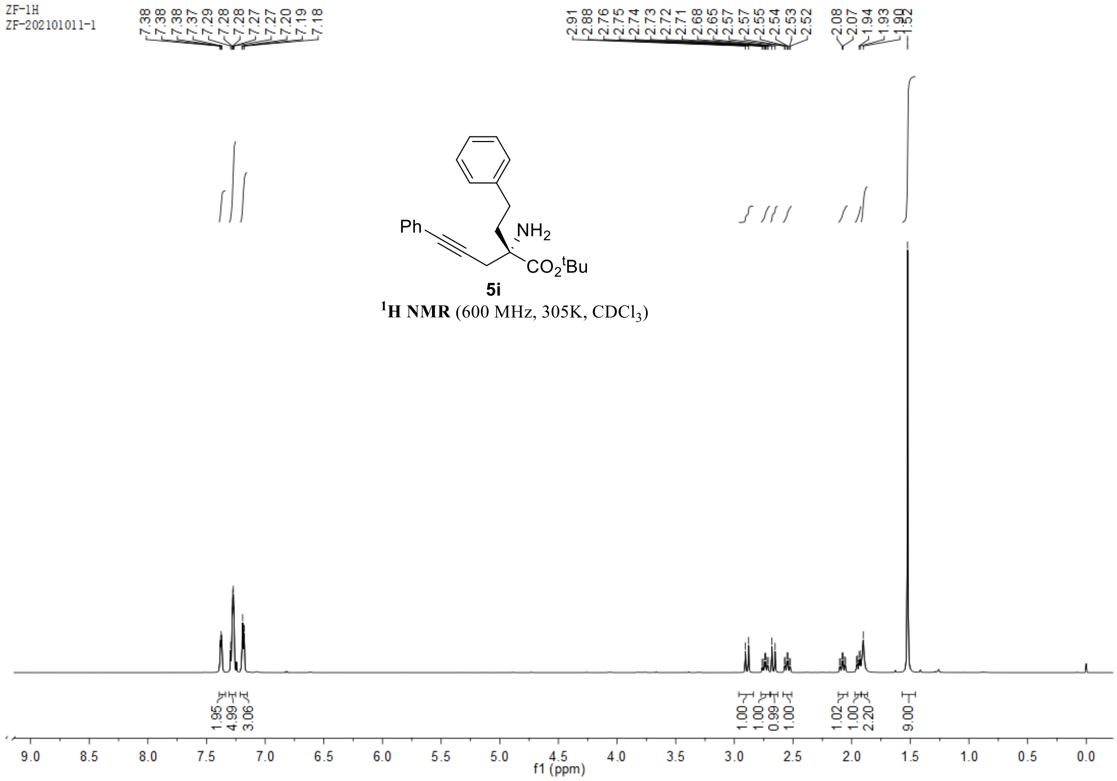

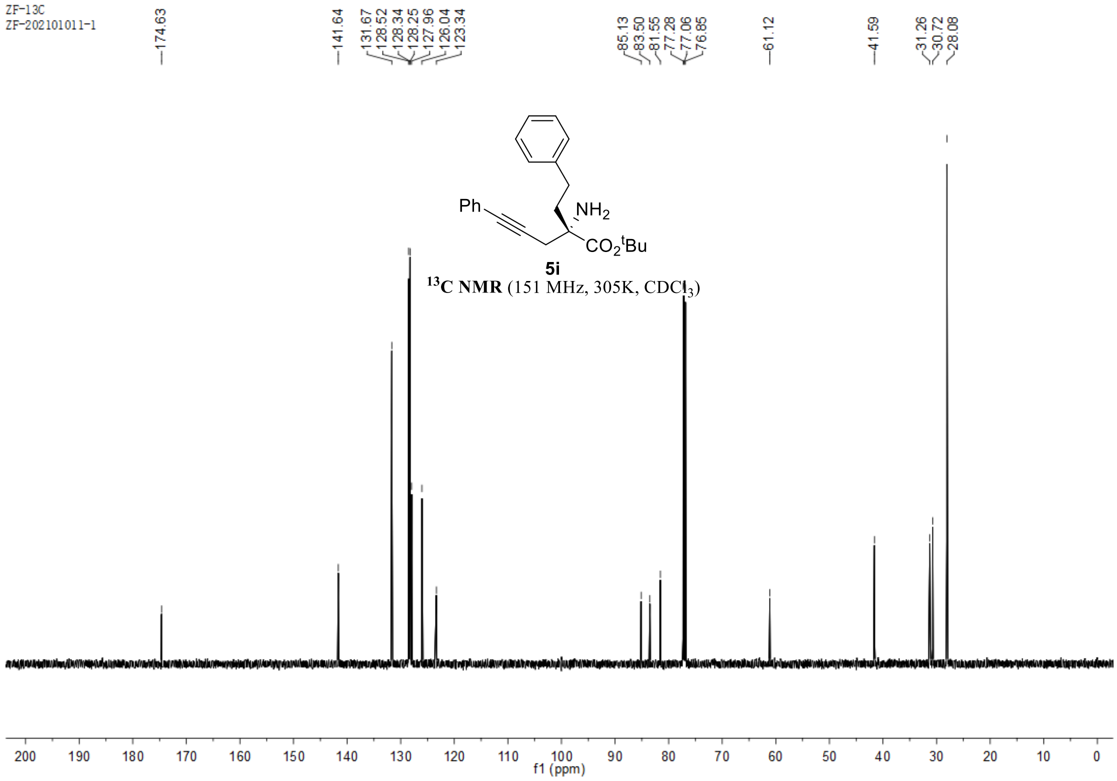

Supplementary Figure 38: NMR of compound 5i.

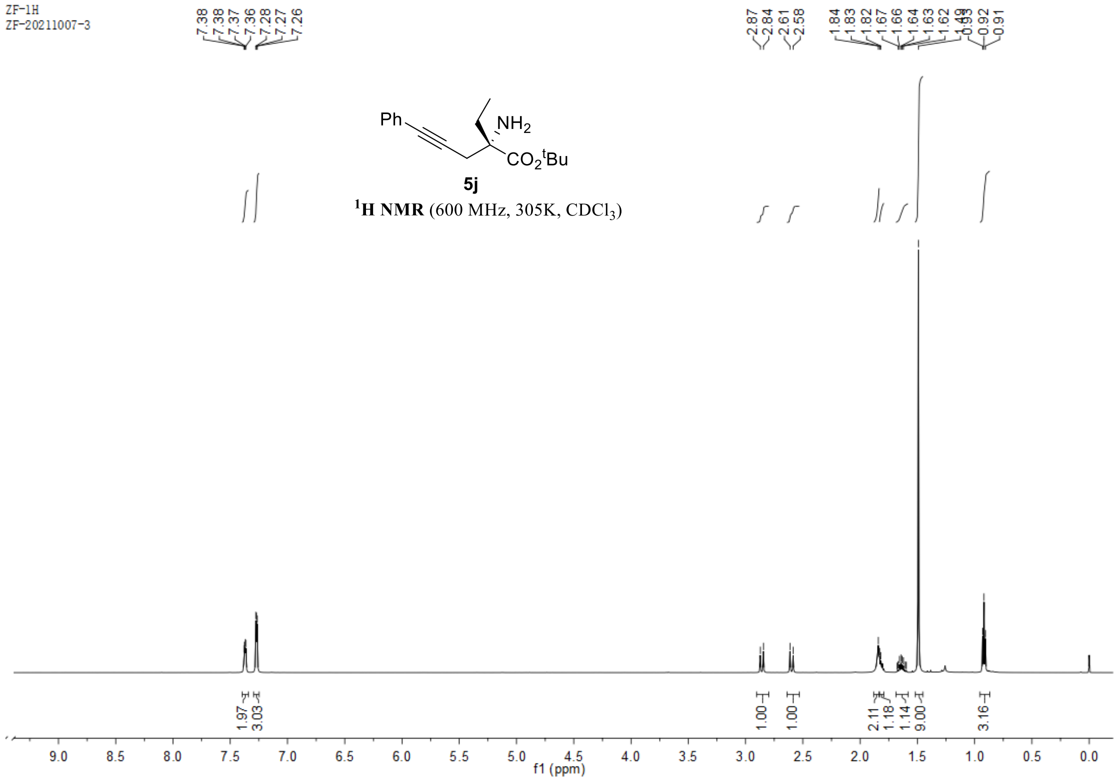

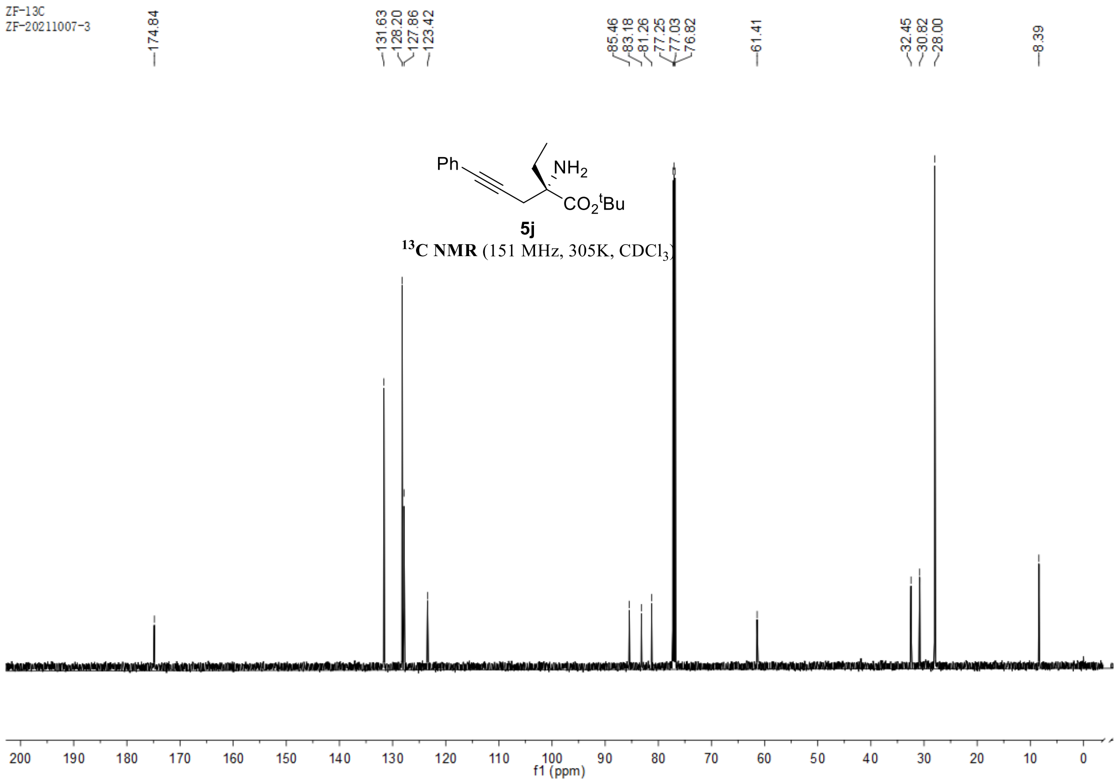

Supplementary Figure 39: NMR of compound 5j.

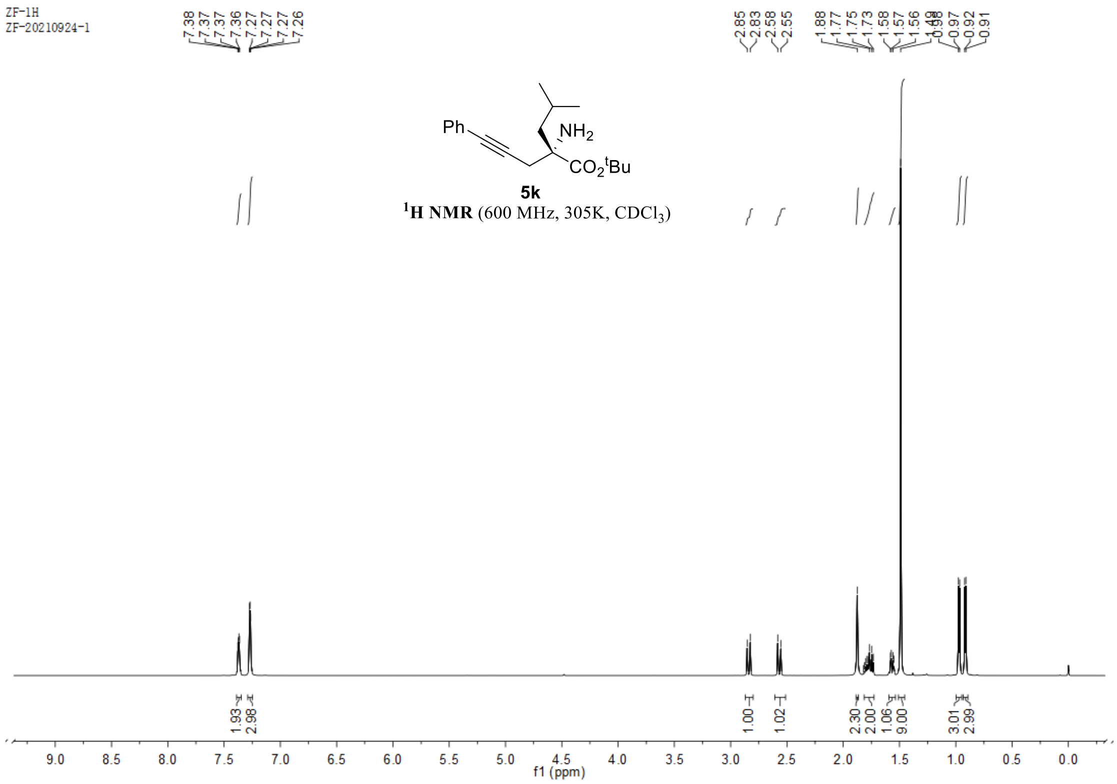

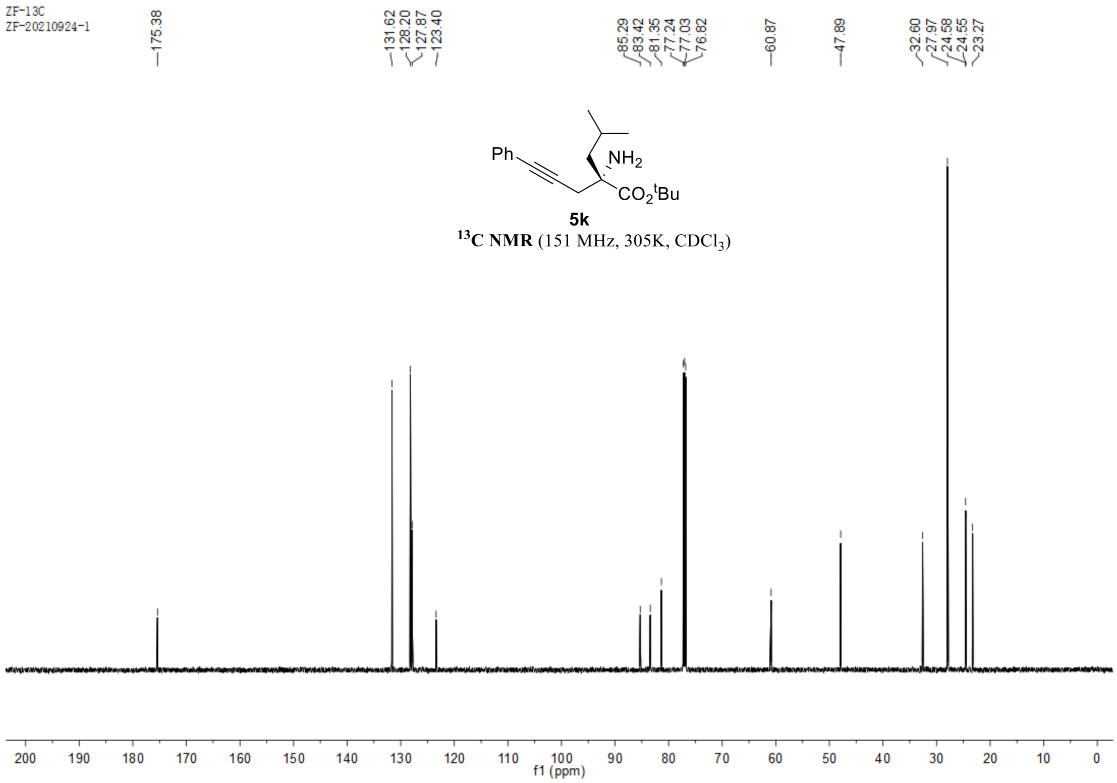

Supplementary Figure 40: NMR of compound 5k.

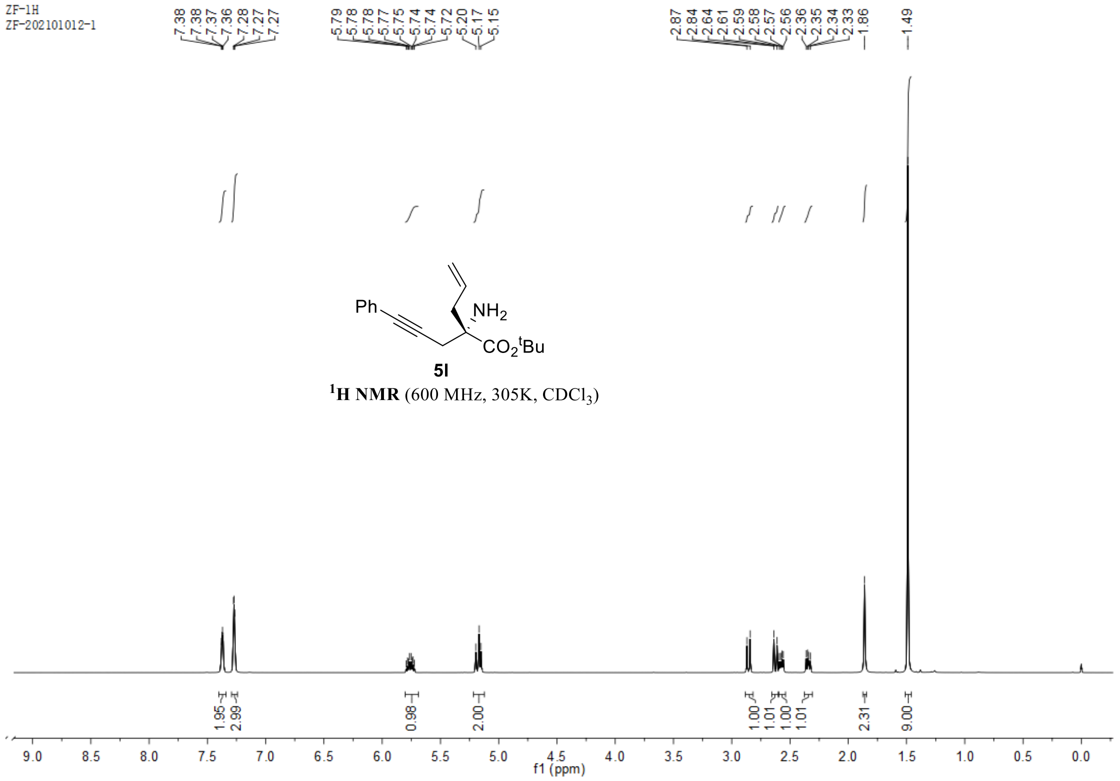

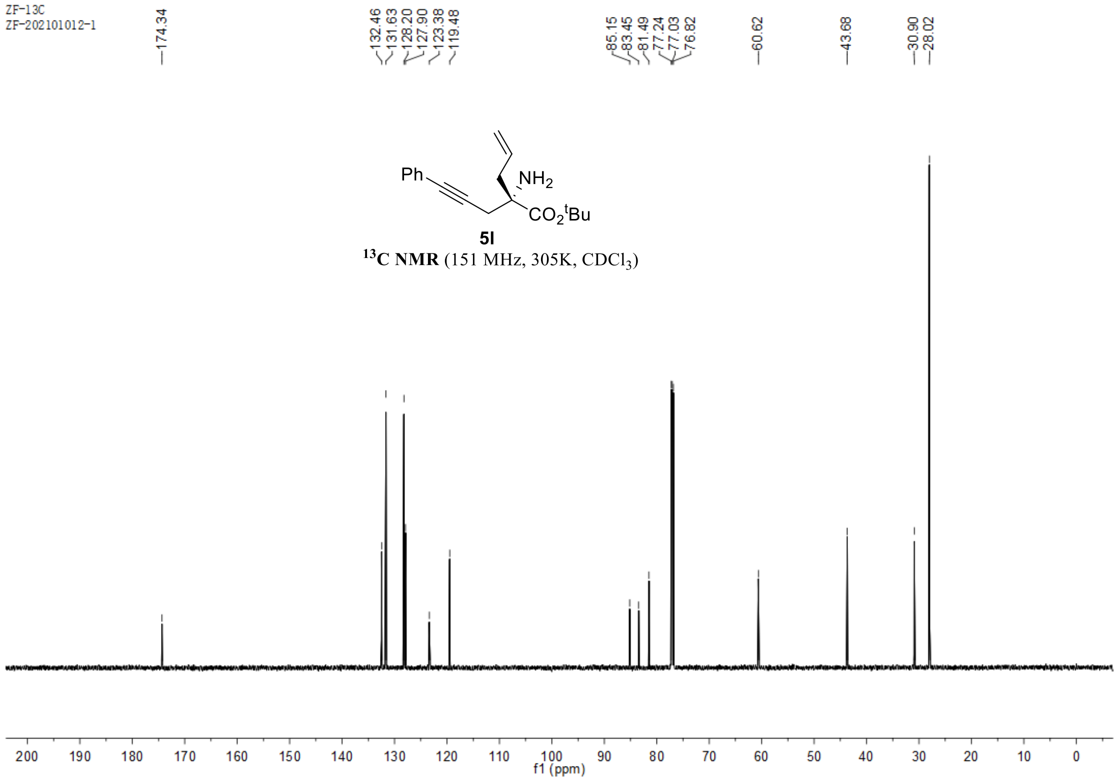

Supplementary Figure 41: NMR of compound 5l.

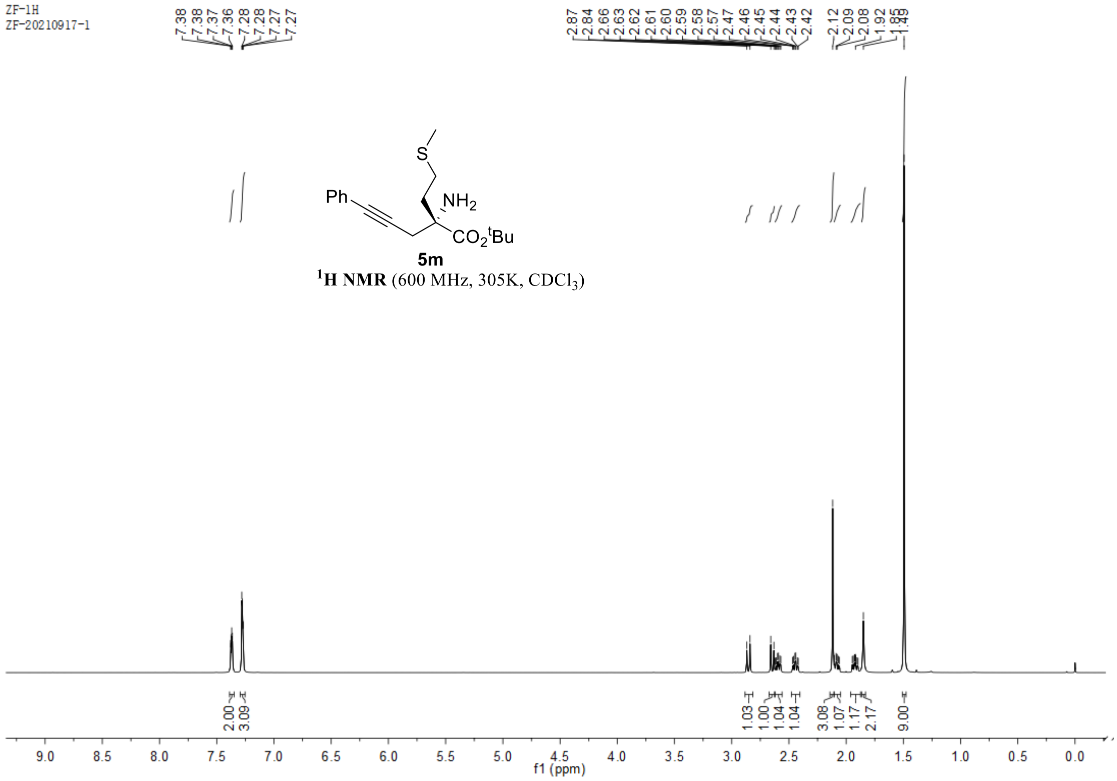

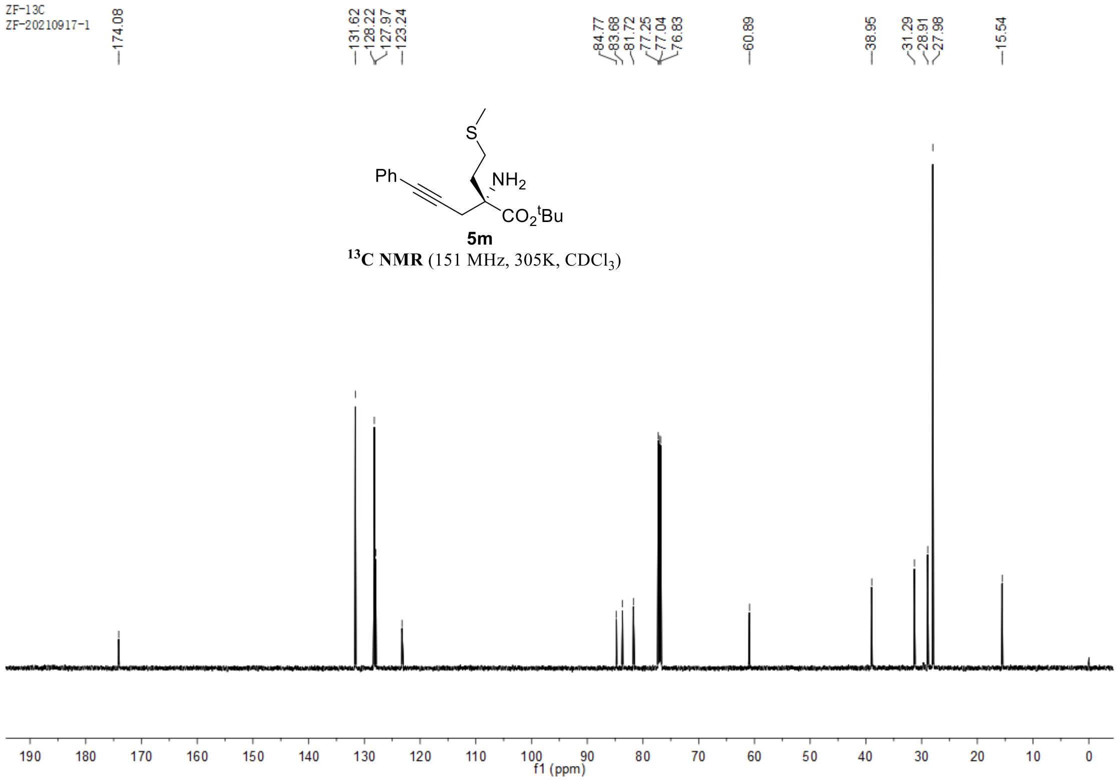

Supplementary Figure 42: NMR of compound 5m.

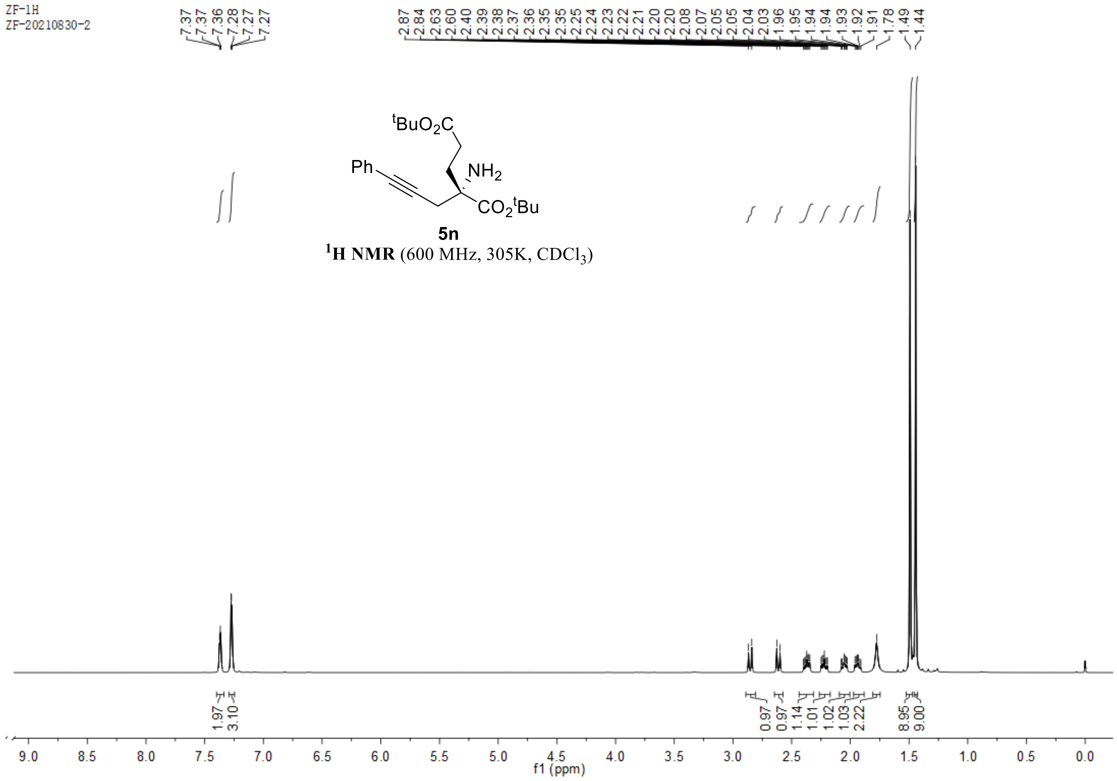

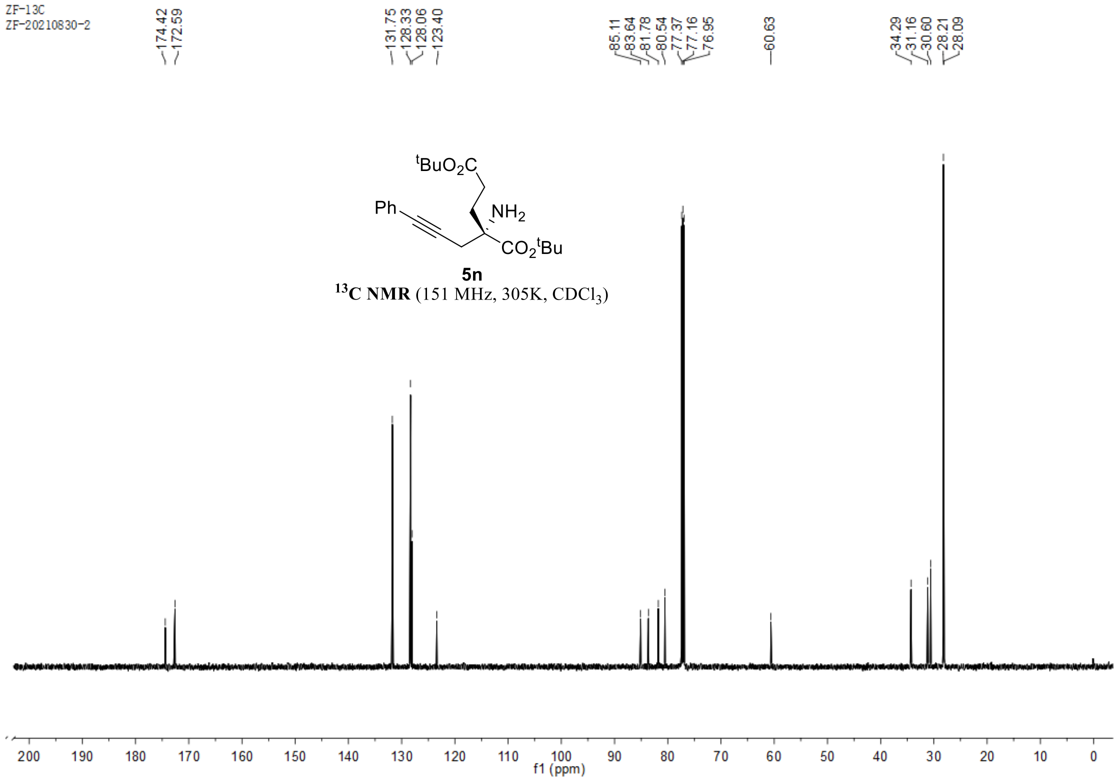

Supplementary Figure 43: NMR of compound 5n.

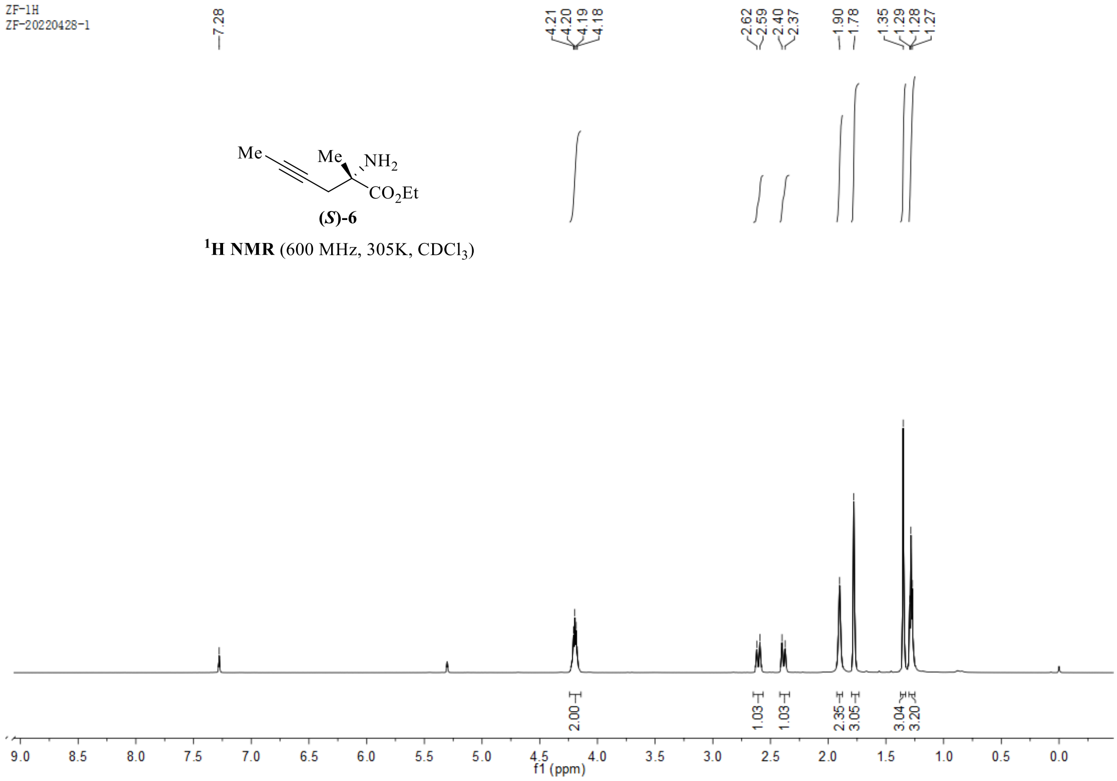

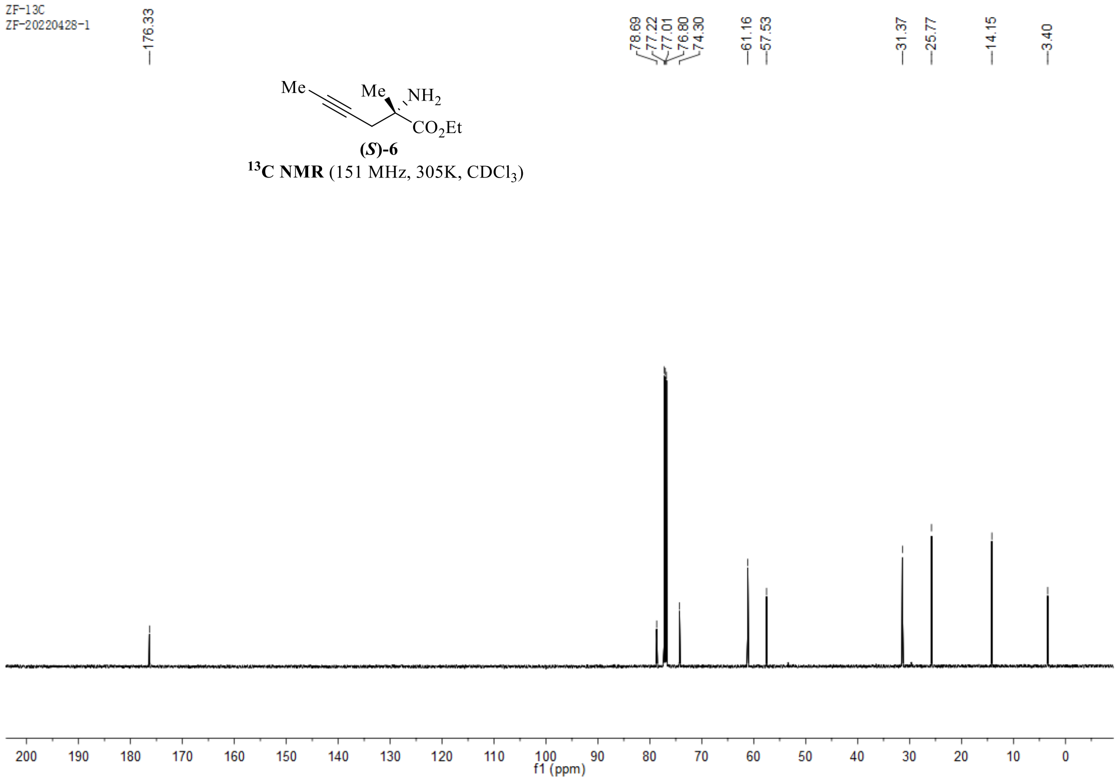

Supplementary Figure 44: NMR of compound (*S*)-6

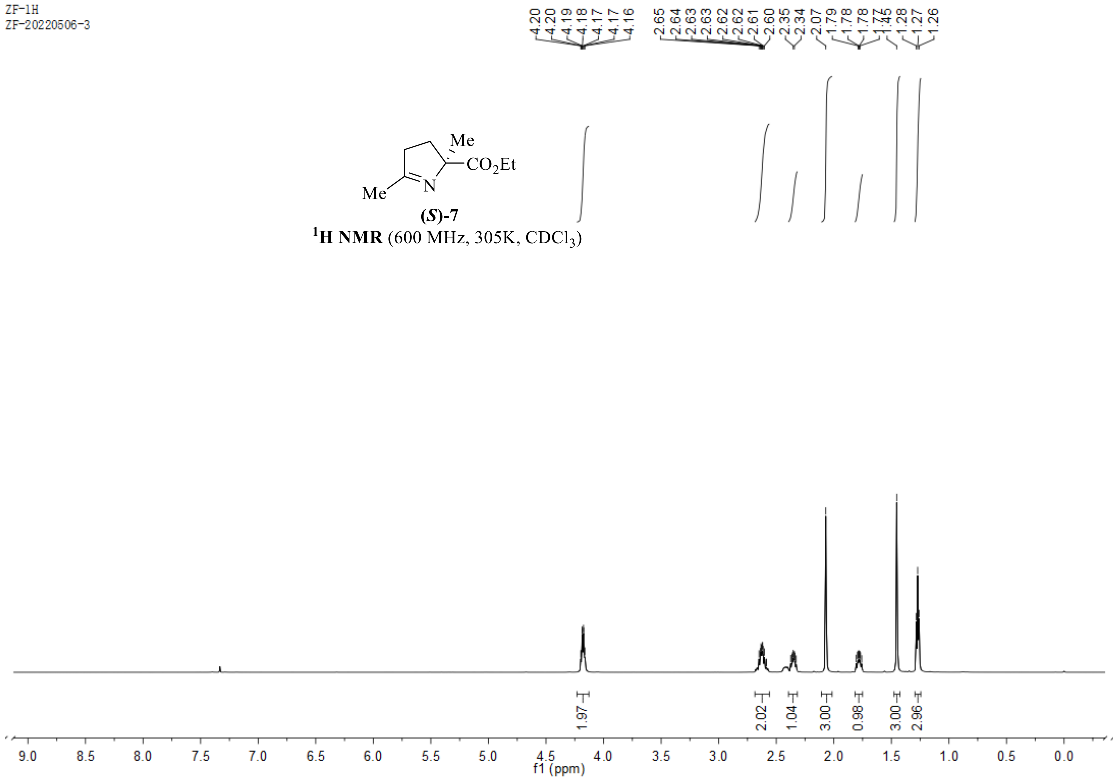

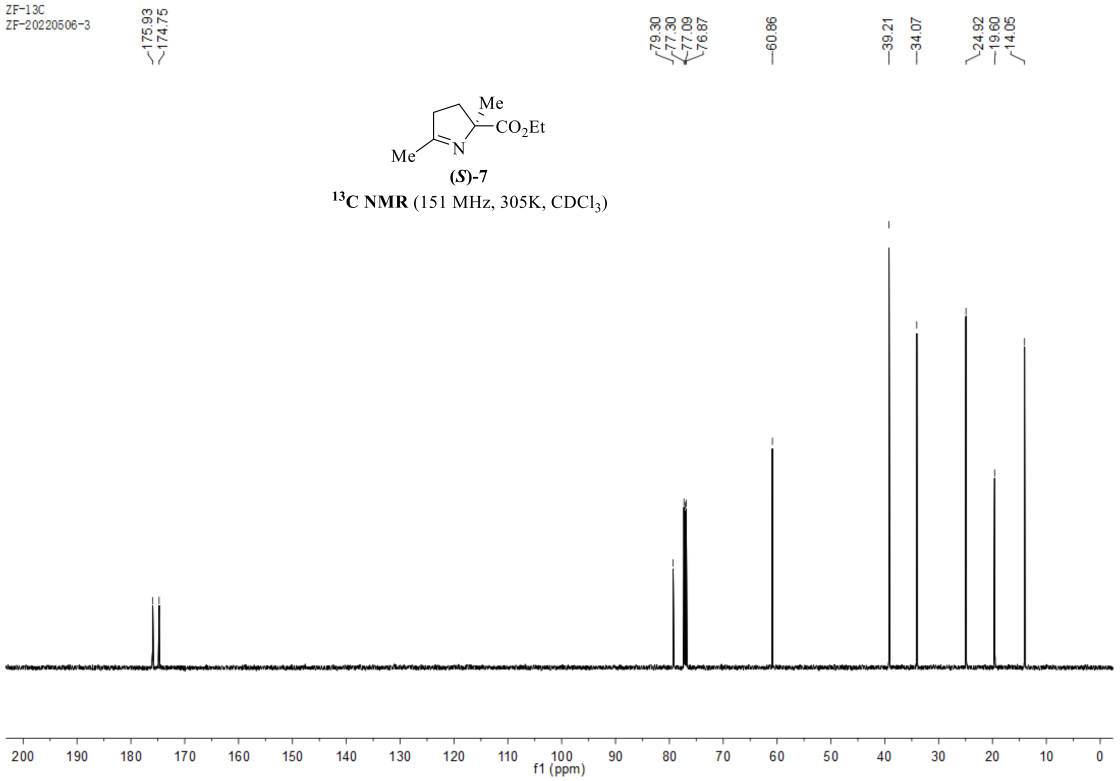

Supplementary Figure 45: NMR of compound (*S*)-7

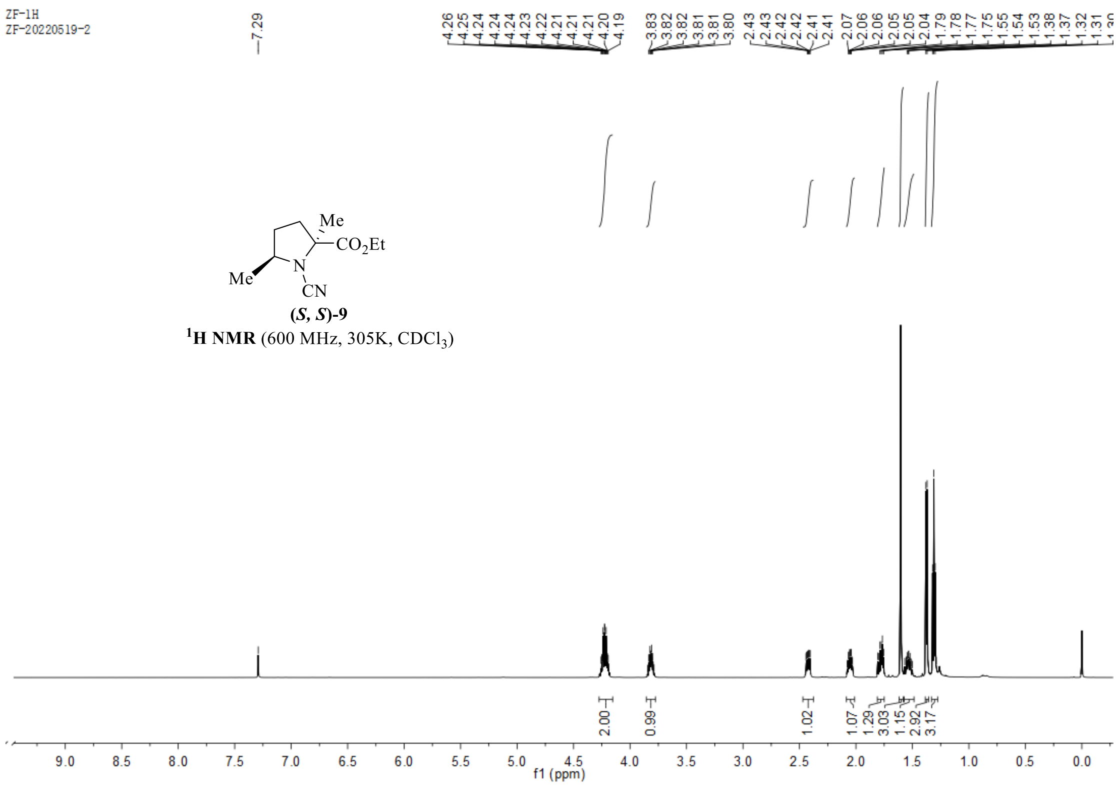

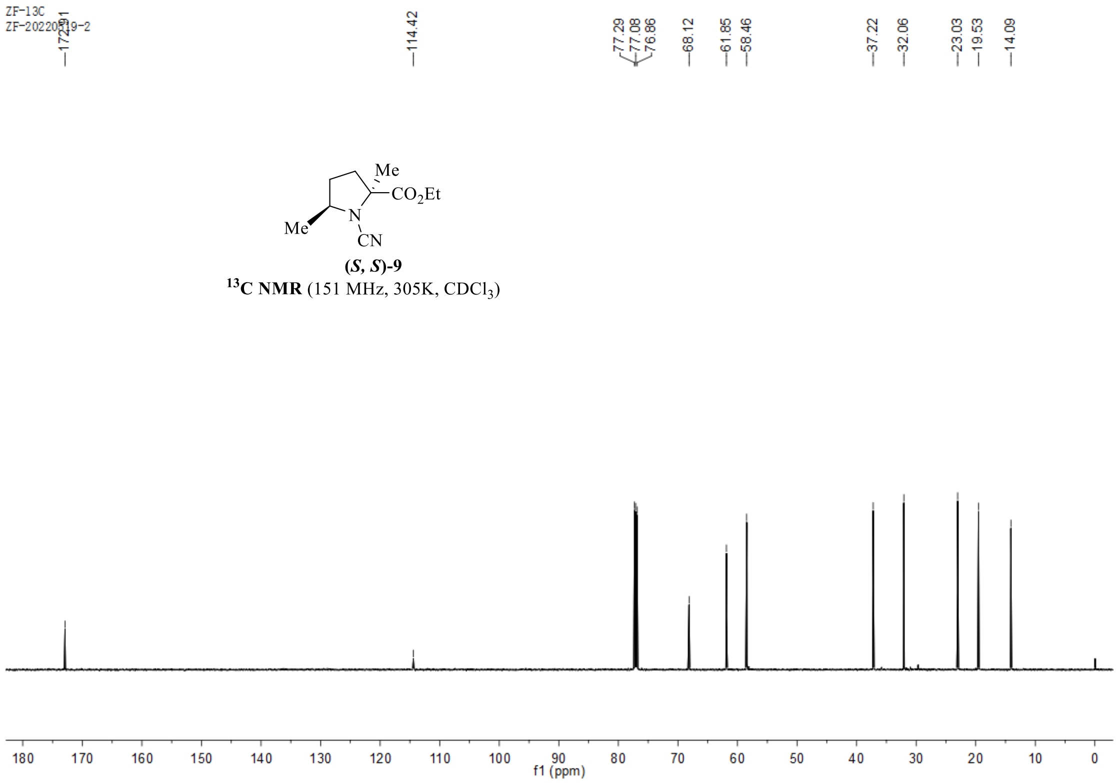

Supplementary Figure 46: NMR of compound (*S, S*)-9

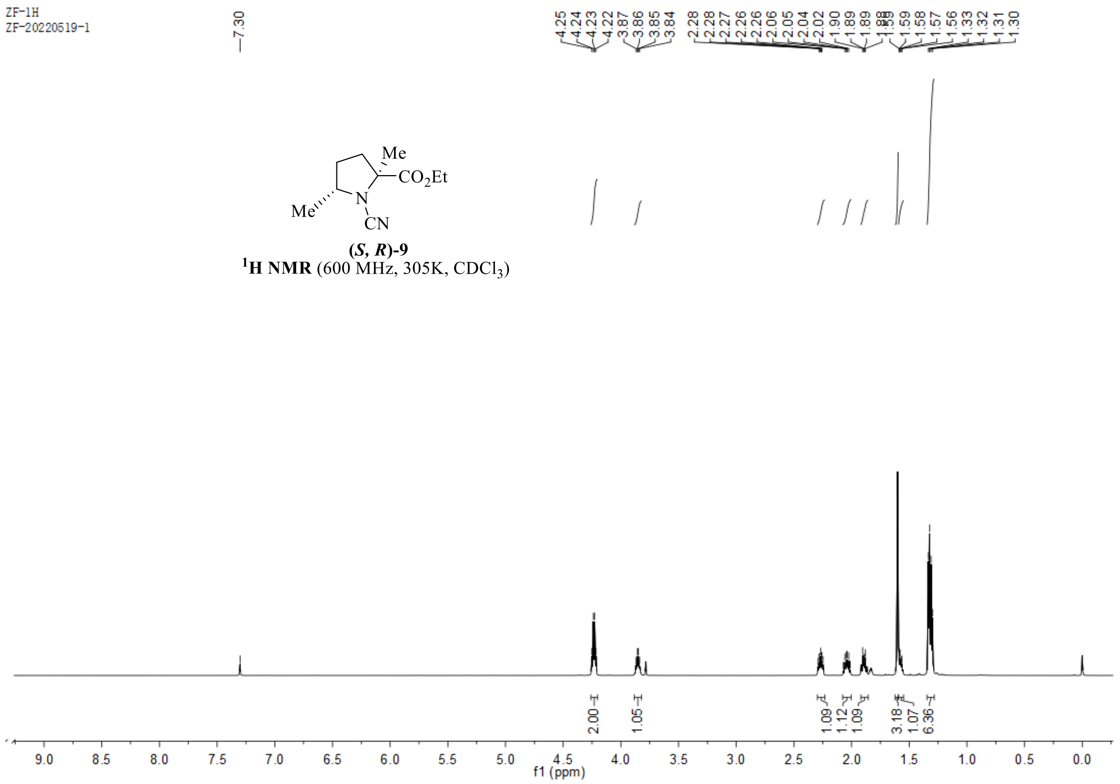

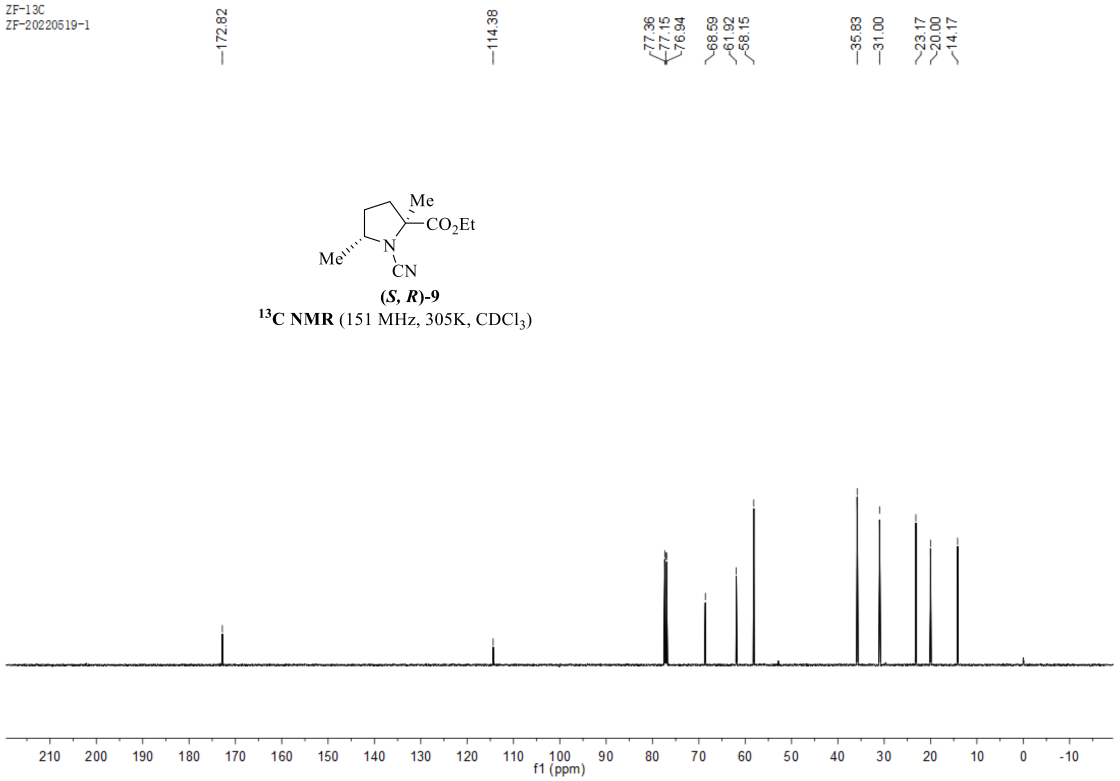

Supplementary Figure 47: NMR of compound (*S, R*)-9

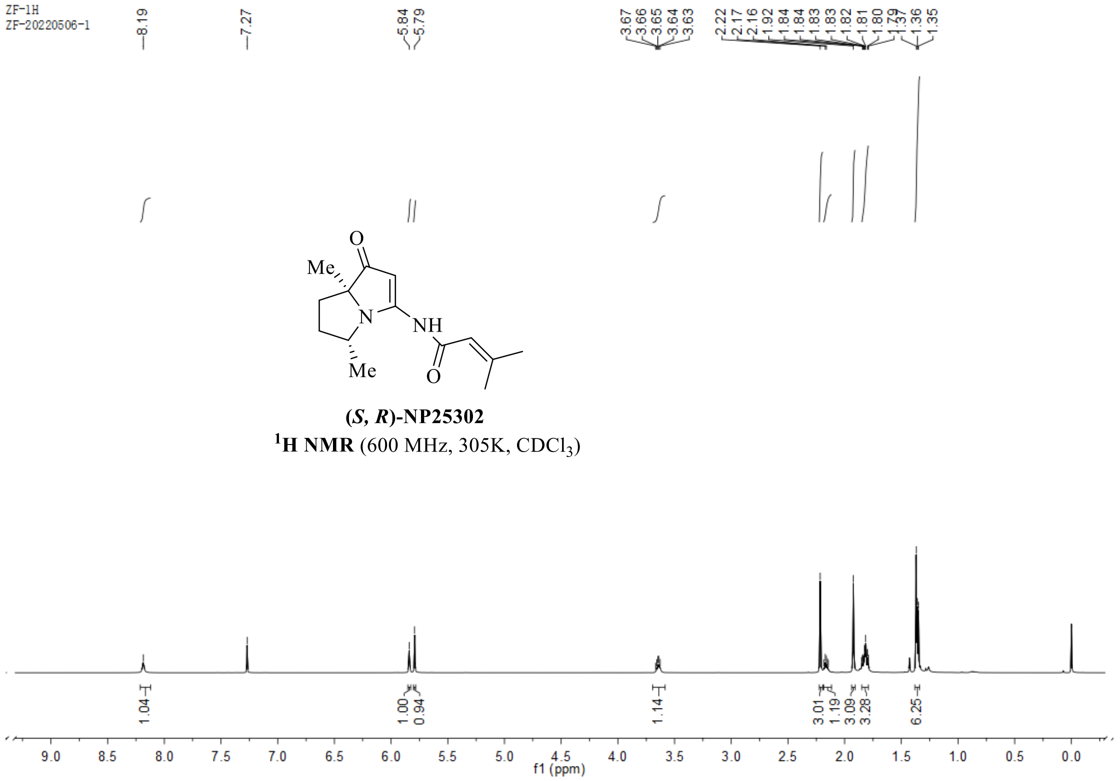

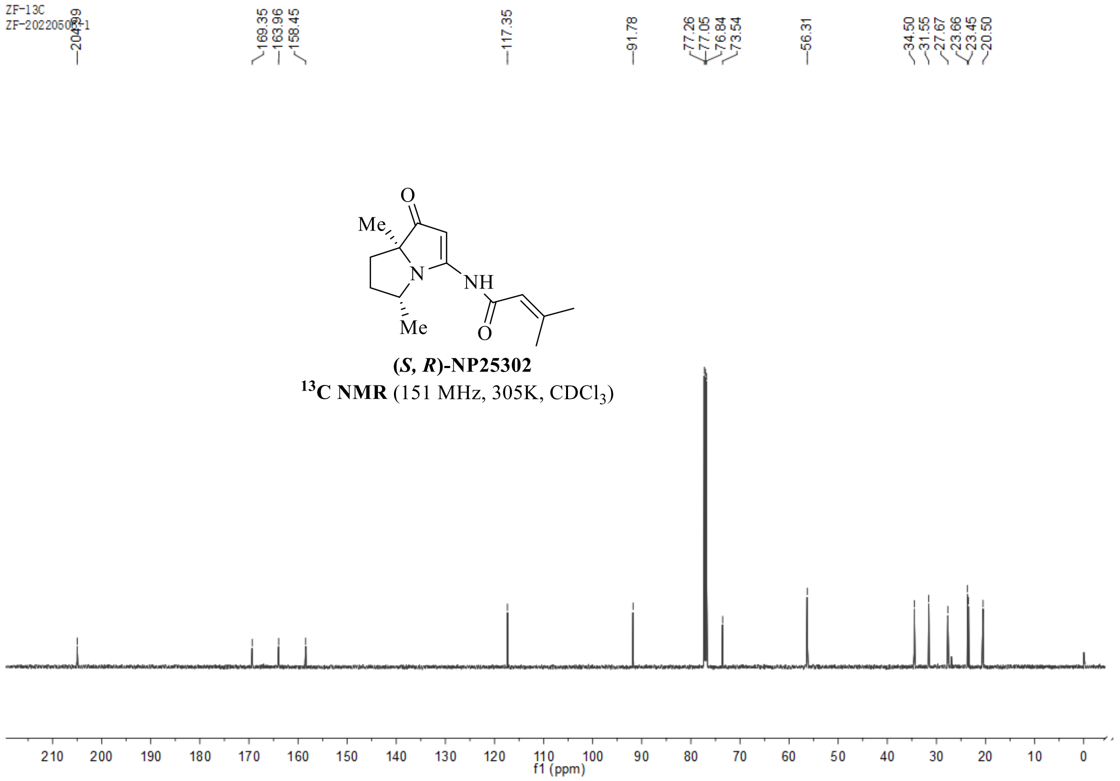

Supplementary Figure 48: NMR of compound (*S, R*)-NP25302

2. Supplementary References

[1] Kartic, M.; Hasina, D. H.; Krishanu, S., *Org. Lett*. **2020**, *22*, 7443-7449.

[2] Chen, L.; Luo, M.-J.; Zhu, F.; Wen, W.; Guo, Q.-X., *J. Am. Chem. Soc.* **2018**, *141*, 9774-9780.

[3] Trujillo, C; Sanchez, S., G.; Karpaviciene, L.; Jahn, U.; Cikotiene, L.; Rulisek, L., *Chem. Eur. J.* **2014**, *33*, 10360-10370.

[4] Peng, L.-P.; He, Z.-Z.; Xu, X.-H.; Guo, C., *Angew. Chem. Int. Ed.* **2020**, *59*, 14270-14274.
